# Effect of turmeric products on knee osteoarthritis: a systematic review and network meta-analysis

**DOI:** 10.1186/s12906-025-05045-z

**Published:** 2025-07-29

**Authors:** Han Su Wai, Thanika Pathomwichaiwat, Thanarat Suansanae, Surakit Nathisuwan, Wipharak Rattanavipanon

**Affiliations:** 1 Clinical Pharmacy Division, Department of Pharmacy, Faculty of Pharmacy, Mahidol University, 447 Sri-ayutthaya Road, Ratchathewi, Bangkok, 10400 Thailand; 2 Department of Pharmaceutical Botany, Faculty of Pharmacy, Mahidol University, Bangkok, 10400 Thailand; 3 Center for Meta-Analysis and Evidence Synthesis of Traditional and Alternative Therapies (META), Bangkok, Thailand

**Keywords:** Turmeric, Knee osteoarthritis, Systematic review, Network meta-analysis, Curcuminoid

## Abstract

**Background:**

Turmeric has traditionally been used to treat various inflammatory conditions, including knee osteoarthritis (OA). There are multiple turmeric preparations available. However, the comparative effectiveness of these products remains unknown. This study aimed to assess the comparative effectiveness of turmeric products for knee OA outcomes by conducting a systematic review and network meta-analysis of randomized, controlled trials (RCTs).

**Methods:**

PubMed, EMBASE, SCOPUS, and ClinicalTrials.gov databases were searched up to August 2024, identifying RCTs that compared turmeric preparations and/or active comparators versus placebo. The primary outcome measured pain reduction, using the Western Ontario and McMaster Universities Osteoarthritis Index (WOMAC), while secondary outcomes evaluated pain using other tools. Mean differences (MDs) were pooled using a random-effects model, and the concept of minimum clinically important difference (MCID) was considered.

**Results:**

Seventeen studies were included. All turmeric preparations significantly reduced WOMAC pain. The mean differences (MD, 95% CI) for WOMAC pain reduction were as follows: − 4.01 (–6.22, − 1.80) for conventional curcuminoid preparations (CT) plus active drug comparators (AC, defined as NSAIDs and acetaminophen), − 3.33 (–5.26, − 1.39) for AC, − 3.17 (–5.50, − 0.83) for CT, and − 2.47 (–3.27, − 1.67) for bioavailability-enhanced curcuminoid preparations (BE). The BE preparation also demonstrated a 30% reduction in WOMAC pain compared to placebo, reaching the MCID threshold. The BE + AC combination led to a 70% reduction in VAS pain compared to AC alone.

**Conclusions:**

All turmeric preparations appear to be effective in reducing knee OA pain when used as monotherapy compared to placebo. However, the certainty of evidence remains low, indicating a need for further research.

**PROSPERO registration number:**

CRD42023464749.

**Clinical trial number:**

not applicable.

**Supplementary Information:**

The online version contains supplementary material available at 10.1186/s12906-025-05045-z.

## Background

Osteoarthritis (OA) is a chronic and progressive joint disease that causes chronic pain, limited mobility and disability [1, 2]. The goals of knee OA treatment are to provide pain relief, improve joint function, and enhance patients’ quality of life [3]. Current pharmacological therapies include analgesics, corticosteroids, and non-steroidal anti-inflammatory drugs (NSAIDs). These agents have well-known serious adverse drug reactions with prolonged use [4]. Significant attempts have been made to find safer alternatives or add-on therapies. Among these alternatives from natural products, turmeric has recently gained significant popularity.

Turmeric (*Curcuma longa* L., synonym: *C. domestica* Valeton), a perennial rhizomatous plant, has been used traditionally for centuries in various disorders [5]. Curcuminoids (2–5% by weight, primarily curcumin) and polysaccharides are the key bioactive substances that have been shown to possess important medicinal properties [6, 7]. Since curcumin, the major bioactive compound of turmeric, is relatively stable and can withstand an acidic environment in the stomach, the common route of turmeric administration is therefore via the oral route [8]. However, due to the poor water solubility and low bioavailability of curcuminoids, bioavailability-enhanced formulations have been developed using various processing techniques, such as adding additives like piperine or formulating curcumin into nanoparticles or emulsions [9]. Furthermore, a polysaccharide-rich fraction can be obtained using hydrophilic solvents [10]. Consequently, a variety of turmeric preparations with different bioactive compositions are available.

Turmeric has been evaluated as a treatment for knee osteoarthritis (OA) in various randomized controlled trials (RCTs) due to its well-recognized anti-inflammatory property [10, 11]. Studies in humans showed that curcumin and its metabolites were detectable in plasma following oral ingestion, suggesting systemic absorption [12, 13]. Studies in animal models showed distribution of curcumin into synovial fluid which suggested that curcumin can potentially reach the site of action [14]. In addition, studies in both animal models and humans showed clear changes in various biomarkers suggesting that curcumin can penetrate the synovial compartment and modulate inflammatory responses in the joint [15].

There were previous systematic reviews and meta-analyses attempting to address this issue [16, 17]. However, variations in turmeric preparations, including differences in phytochemical content, dosages and bioavailability-enhancing techniques, are present across studies making it difficult to reach a reliable conclusion [10, 18]. Moreover, these studies use different outcome measures, complicating efforts to summarize the effect size of the intervention [6]. A more recent Bayesian network meta-analysis (NMA) by Zhao et al. (2024) assessed the efficacy and safety of curcumin—either as a monotherapy or in combination with other agents—but grouped all curcumin interventions together without distinguishing specific formulations based on their phytochemical content, or enhancement strategies [19]. In contrast, our study addresses these limitations by conducting an NMA that classifies turmeric interventions according to their phytochemical composition and processing techniques, allowing for a more detailed comparison of the efficacy and safety of distinct turmeric-based therapies in patients with knee OA.

## Methods

This study was conducted in accordance with the Preferred Reporting Items for Systematic Reviews and Meta-Analyses Extension for Network Meta-Analysis (PRISMA-NMA) standards (see Appendix **A** for checklist) [20]. The protocol was pre-registered on PROSPERO (CRD42023464749).

### Search strategy and study selection

An extensive literature search was performed across MEDLINE (via PubMed), EMBASE, SCOPUS, and ClinicalTrials.gov databases, identifying relevant studies up to August 2024. Search terms included Medical Subject Headings (MeSH) such as turmeric, “curcuma longa”, “curcuma domestica”, curcumin, curcuminoid, osteoarthritis, “degenerative arthritis” and other synonymous words. The name of the species was checked on the World Flora Online (WFO) database [21]. Relevant studies were retrieved in full texts and reference lists of those studies were reviewed. Efforts were made to contact corresponding authors whenever clarification regarding study details was needed. No language restrictions were applied.

All RCTs in humans meeting the following criteria were included: (1) conducted in adults aged *≥* 18 years with a clinical or radiological diagnosis of symptomatic primary knee OA; (2) compared the efficacy of turmeric preparations, used either alone or combined with standard treatment, against either a placebo or active comparators; (3) measured patient-reported outcomes (improvement in pain, stiffness, and function) as outcomes. RCTs with two or more intervention arms were included provided that data for the turmeric-only intervention arm were available in comparison with a placebo or an active comparator. In line with the American College of Rheumatology 2019 guidelines, glucosamine and chondroitin were not included as part of the standard treatment comparator [4]. The study selection process was independently conducted by two investigators (H.W. and W.R.).

### Data extraction and risk of bias assessment

Study characteristics (year, study design, country, and treatment duration), patient characteristics (age, sex, body mass index, duration of knee pain, and baseline pain intensity), and regimen characteristics (types of turmeric interventions, comparators, and dose), and outcomes of interest were extracted. If data were reported unclearly, corresponding authors were contacted. Intention-to-treat analysis data were used whenever available. The Cochrane Collaboration’s ROB 2.0 tool was utilized for assessing the risk of bias in the included RCTs [22].

### Classification of interventions

Turmeric interventions were classified into three groups based on phytochemical composition and processing techniques. These included conventional curcuminoid preparations (CT), bioavailability-enhanced curcuminoid preparations (BE), and polysaccharide preparations (PLS), as described in Table 1. Active drug comparators (AC) included standard pharmacological treatment, including NSAIDs and analgesics. Placebo (P) was set as the reference treatment for our NMA.

**Table 1:** Description of included interventions

Group	Characteristics	Interventions
CT	Extraction using hydroalcoholic solution, concentrating curcuminoids	Curcuminoids capsules, *C. longa* extract (Haridra), *C. domestica* extract
BE	Enhanced bioavailability via additives (such as piperine), using matrices or particle size reduction techniques	BCM-95^®^, CuraMed^®^ (BCM-95), SinaCurcumin^®^, SinaCurcumin™, Curcugen^®^, Theracurmin^®^, Curene^®^, C3 complex^®^, Longvida^®^, FLEXOFYTOL^®^
PLS	Polysaccharides-rich extract or fraction in combination with turmeric volatile oils; contains low amount of curcuminoid content	Turmacin™, Turmacin Plus
AC	Standard pharmacological treatments used for knee OA	NSAIDs, acetaminophen
P	Placebo treatments	Inert substances resembling turmeric interventions, containing no active curcuminoid or polysaccharide ingredients

**Abbreviation**: AC, active drug comparator; BE, bioavailability-enhanced curcuminoid preparations; CT, conventional curcuminoid preparations; P, placebo; PLS, polysaccharide preparations

### Outcomes of interest

Change in the Western Ontario and McMaster Universities Osteoarthritis Index (WOMAC) pain subscale from baseline was used as the primary outcome. Secondary outcomes included the change from baseline of each subscale of WOMAC, changes in other pain scores (e.g. visual analog scale (VAS)). Adverse events (AE) associated with turmeric preparations were also reported.

### Unit standardization

The extracted efficacy outcomes were continuous and presented on different scales. Consequently, these data were converted to a common scale to enable the calculation of pooled estimates in mean difference (MD). In our study, VAS scores were converted to a 0–100 scale. The WOMAC pain subscale (5 questions) was converted to a 0–20 scale, stiffness (2 questions) to 0–8, and function (17 questions) to 0–68 in each respective study, employing the rescaling equation [23], as described in Appendix **B** (see Additional file 1).

### Certainty of evidence

Evaluation of the certainty of evidence was performed by two independent investigators (H.W. and W.R.) following the GRADE guidance [24]. Each outcome was graded using this approach, and the overall certainty of evidence was rated as high, moderate, low, or very low, using the GRADEpro^®^ GDT software online version [25].

### Data synthesis and statistical methods

A pairwise meta-analysis was conducted to integrate the treatment effects of turmeric products and control groups. Mean differences (MD) with the 95% confidence interval (CI) were pooled using a random-effects model [26]. Cochrane Q test and I^2^ statistics were used to assess heterogeneity [27, 28]. A network meta-analysis was performed to compare each intervention to a common comparator (placebo, P) by pooling the estimated MDs across studies. The global inconsistency test evaluated inconsistency in the entire network. A transitivity assessment was conducted to validate the distribution of potential clinical and methodological variations. Potential effect modifiers were evaluated for each respective outcome. The surface under the cumulative ranking curve (SUCRA) was used to rank turmeric interventions for each outcome. Subgroup analyses were prespecified based on the body mass index (BMI), treatment duration (follow-up period), dose, and ethnicity. Sensitivity analyses were pre-specified to evaluate the robustness of the findings including the impact of the follow-up period, risk of bias, and small study effects, the latter of which was examined using adjusted funnel plots. All analyses were conducted using STATA statistical software, version 14.0. Statistical significance was considered achieving a *p*-value less than 0.05.

To assess clinical significance, the minimum clinically important difference (MCID) concept was considered [29]. In our study, the MCID threshold was pre-specified as a 20% change from baseline for all WOMAC subscore measures. This threshold was chosen based on a systematic review conducted by an OMERACT Rasch Working Group Systematic Review and Critique [30]. For MCID cut-point of the VAS, a 30% change from baseline was chosen based on the outcomes in chronic pain trials [31]. After calculating the effect sizes along with 95% CI, percent change from baseline was calculated for each head-to-head trial to assess if they met the MCID threshold.

## Results

### Study selection

**Fig. 1:**
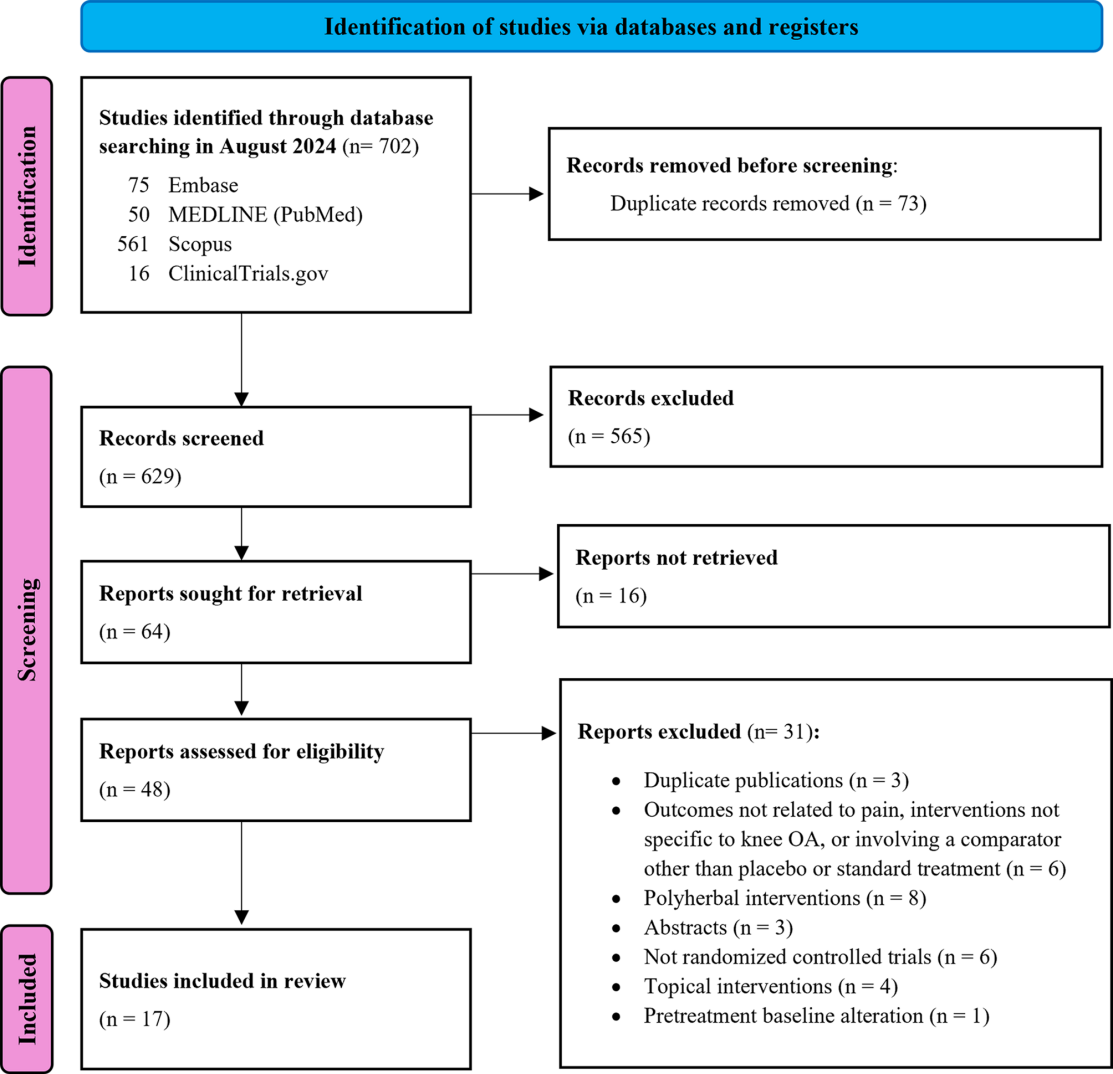
PRISMA flow diagram of study selection

Initially, 702 potentially eligible studies were identified. After the title and abstract screening, 64 records remained. Only 48 studies were available for full-text assessment. Thirty-one studies were excluded for not meeting our study criteria. Finally, 17 studies were included in our analysis. The PRISMA flow chart of literature screening is illustrated in Fig. 1. The search strategy is demonstrated in Appendix **C** (see Additional file 1).

### Characteristics and risk of bias of all included studies

The 17 included RCTs originated mostly from Asia. Participants were adult patients, with the majority being female. Eight studies compared various turmeric interventions with active drug comparators (diclofenac, ibuprofen, and acetaminophen) [32–39] with three studies using diclofenac in both treatment and comparator arms [32, 36, 39] (Table 2) and Appendix **D** (see Additional file 1). Details of intervention used in each study duration of knee OA, baseline pain intensity and other baseline characteristics are described in in Appendix **D** (see Additional file 1). The risk of bias using ROB 2.0 suggested that 35%, 41%, and 24% of the studies had low risk, some concerns, and high risk of bias, respectively. (see Appendix **E**, Additional file 1)

**Table 2:** Characteristics of all included studies

Authors	Treatment		Sample size (male/female)		Age, years, mean *±* SD		BMI (kg/m^2^), mean *±* SD	
	Intervention	Control	Intervention	Control	Intervention	Control	Intervention	Control
Shep, et al. 2019 [37]	BE	AC	70 (45/25)	69 (48/21)	53.09 *±* 4.17	52.14 *±* 3.76	NA	NA
Singhal, et al. 2021 [38]	BE	AC	73 (20/53)	71 (17/54)	53.1 *±* 10.9	50.8 *±* 9.9	NA	NA
Haroyan, et al. 2018 [45]	BE	P	66 (6/60)	68 (3/65)	54.65 *±* 8.84	56.04 *±* 8.55	28.33 *±* 3.6	28.81 *±* 3.36
Atabaki, et al. 2020 [32]	BE + AC	AC	15 (0/15)	15 (0/15)	49.13 *±* 5.8^*^	48.26 *±* 5.11^*^	22 *±* 1.39^*^	21.9 *±* 1.51^*^
Hashemzadeh, et al. 2020 [46]	BE	P	36 (7/29)	35 (4/31)	54.11 *±* 5.8	56.54 *±* 5.77	NA	NA
Lopresti, et al. 2021 [47]	BE	P	51 (27/24)	50 (24/26)	59.59 *±* 6.57^*^	57.92 *±* 6.22^*^	28.93 *±* 4.64^*^	28.82 *±* 4.24^*^
Nakagawa, et al. 2014 [48]	BE	P	18 (4/14)	23 (5/18)	71.9 *±* 5.3	66.1 *±* 7.2	25.1 *±* 2.7	24.8 *±* 2.3
Panda, et al. 2018 [49]	BE	P	25 (NA)	25 (NA)	55.2 *±* 8.58	53.12 *±* 8.25	25.44 *±* 2.75	24.92 *±* 1.92
Panahi, et al. 2014 [50]	BE	P	19 (5/14)	21 (4/17)	57.32 *±* 8.78	57.57 *±* 9.05	28.75 *±* 3.17	29.64 *±* 4.46
Gupte, et al. 2019 [33]	BE	AC	17 (6/11)	25 (2/23)	57 *±* 7.5	54 *±* 8	NA	NA
Henrotin, et al. 2019 [51]	BE (high dose), BE (low dose)	P	49 (10/39), 47 (7/40)	45 (11/34)	60.9 *±* 9.78, 61.4 *±* 7.49	63.3 *±* 7.69	29.4 *±* 4.87, 30.4 *±* 5.23	29.4 *±* 5.2
Pinsornsak and Niempoog 2012 [36]	CT + AC	AC	38 (NA)	37 (NA)	NA	NA	NA	NA
Srivastava, et al. 2016 [39]	CT + AC	AC	78 (25/53)	82 (32/50)	50.23 *±* 8.08	50.27 *±* 8.63	28.32 *±* 5.06	27.4 *±* 5.76
Kuptniratsaikul, et al. 2009 [35]	CT	AC	52 (11/41)	55 (10/45)	61.4 *±* 8.7	60 *±* 8.4	26.4 *±* 3.7	26.8 *±* 4.8
Kuptniratsaikul, et al. 2014 [34]	CT	AC	171 (14/157)	160 (21/139)	60.3 *±* 6.8	60.9 *±* 6.9	26.5 *±* 3.7	26.6 *±* 4
Madhu, et al. 2013 [52]	PLS	P	30 (13/17)	30 (13/17)	56.63 *±* 10.58	56.77 *±* 9.98	27.01 *±* 4.6	27.97 *±* 4.21
Wang, et al. 2020 [53]	PLS	P	36 (18/18)	34 (13/21)	61.3 *±* 8.5	62.4 *±* 8.8	29.9 *±* 6.3	30.6 *±* 7.2

**Abbreviation**: AC, active drug comparator; BE, bioavailability-enhanced curcuminoid preparations; CT, conventional curcuminoid preparations; NA, not available; P, placebo; PLS, polysaccharide preparations

^*^ converted from standard error of the mean

### Pairwise meta-analysis

Seven studies (*N* = 913) were included in the pairwise meta-analysis of WOMAC pain. The analysis showed that BE significantly reduced WOMAC pain compared to placebo with a pooled MD (95% CI) of − 2.47 (–3.25, − 1.68). Regarding the secondary outcomes, none of the interventions showed any significant improvement on WOMAC stiffness compared to placebo. For WOMAC function, significant improvement was observed with BE compared to placebo, with a pooled MD (95%CI) of − 9.62 (–12.47, − 6.76). For VAS pain, both BE and PLS significantly reduced pain compared to the placebo, with a pooled MD (95% CI) of − 16.77 (–20.94, − 12.60) and − 26.55 (–36.53, − 16.57), respectively. Other comparisons, except AC vs. BE, obtained statistically significant results. Further details are presented in Appendix **F** (see Additional file 1).

### Network meta-analysis

**Fig. 2:**
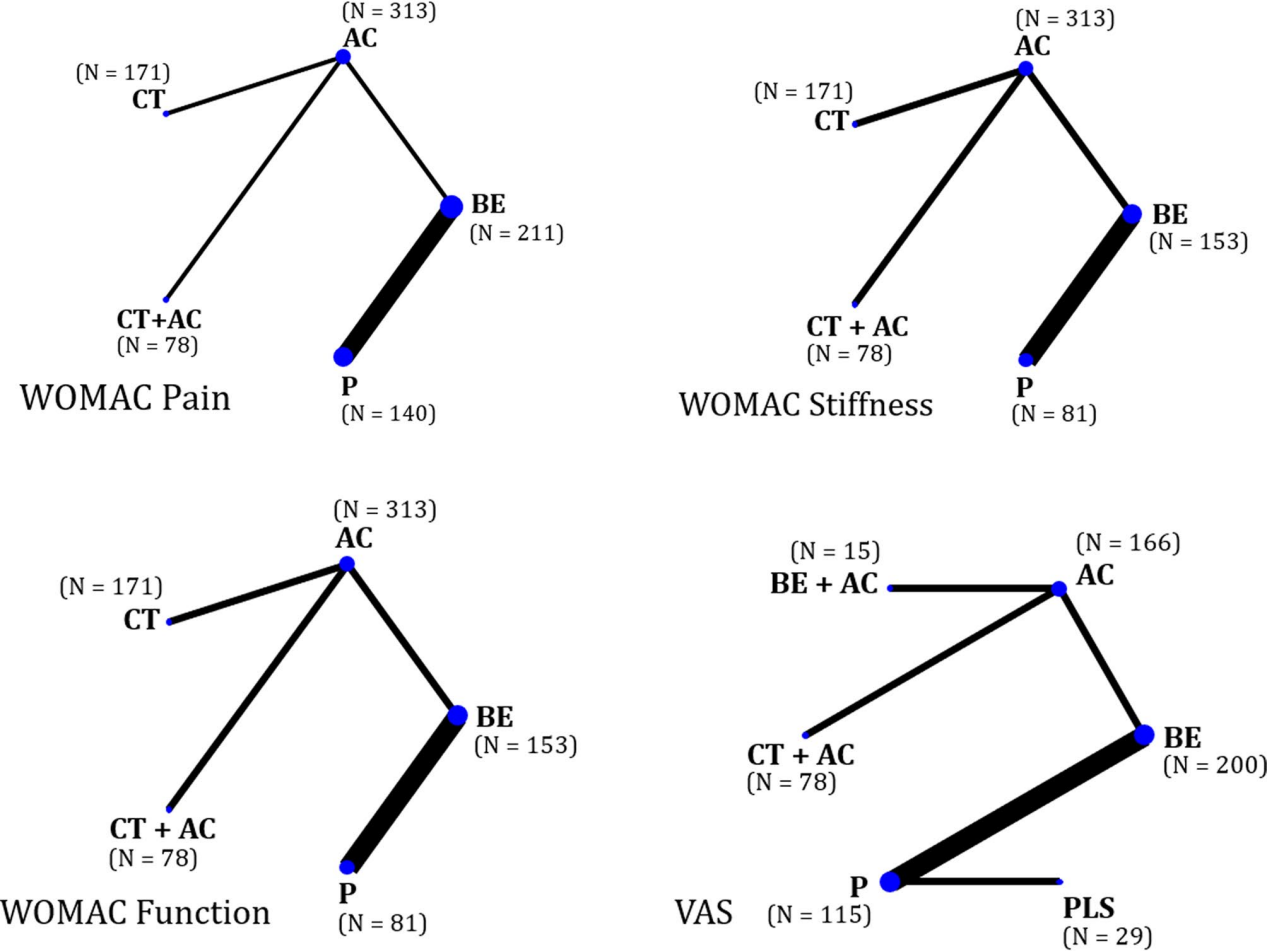
Network maps of WOMAC Pain, Stiffness, Function, and VAS outcomes. Abbreviation: AC, active drug comparator; BE, bioavailability-enhanced curcuminoid preparations; BE + AC, bioavailability-enhanced curcuminoid preparations + active drug comparator; CT, conventional curcuminoid preparations; CT + AC, conventional curcuminoid preparations + active drug comparator; P, placebo; PLS, polysaccharide preparations

The network maps for WOMAC pain, stiffness, and function outcomes included five interventions (CT, BE, CT + AC, AC, P) while the VAS outcome network map involved six interventions (BE, BE + AC, CT + AC, PLS, AC, P) (Fig. 2). The pooled estimates of all outcomes were based on the inconsistency model due to the detection of global inconsistency. (see Appendix **G**, Additional file 1)

### Primary outcome

**Fig. 3:**
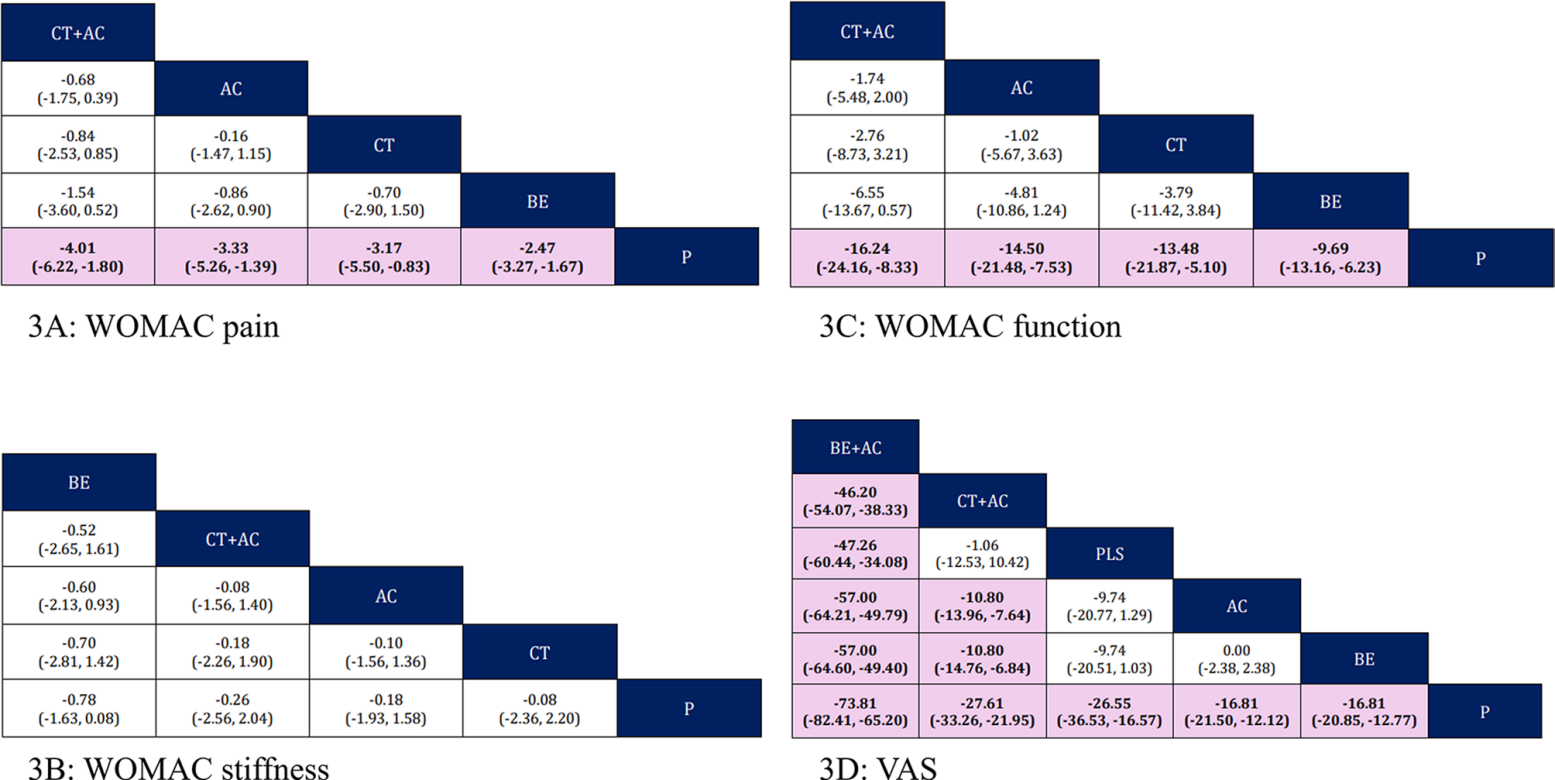
League table for network-estimated mean differences (95% confidence intervals) among interventions. Treatments are arranged according to their SUCRA ranking. For each outcome, comparisons should be interpreted from the column to the row, with the intervention in the row serving as the reference for the comparison. Abbreviation: AC, active drug comparator; BE, bioavailability-enhanced curcuminoid preparations; BE+AC, bioavailability-enhanced curcuminoid preparations + active drug comparator; CT, conventional curcuminoid preparations; CT + AC, conventional curcuminoid preparations + active drug comparator; P, placebo; PLS, polysaccharide preparations

Seven studies (*N* = 913) were included in the network meta-analysis of WOMAC pain. All interventions significantly reduced WOMAC pain compared to placebo, with an MD (95% CI) of − 4.01 (–6.22, − 1.80) for CT + AC, − 3.33 (–5.26, − 1.39) for AC, − 3.17 (–5.50, − 0.83) for CT, and − 2.47 (–3.27, − 1.67) for BE. No significant difference was found among different interventions. The SUCRA ranking of WOMAC pain outcome among interventions was CT + AC > AC > CT > BE > P (Fig. 3). For the MCID evaluation, the % change from baseline for BE vs. P was 29.69%, exceeding the MCID cut-point (Table 3). Regarding the transitivity assessment, most of the effect modifiers did not show extreme variations that would suggest a significant violation of transitivity, except for the baseline pain intensity, which was significantly higher in the CT + AC vs. AC comparison than in other comparisons. (see Appendix **K**, Additional file 1)

**Table 3:** Percentage change from baseline in WOMAC pain, function, and VAS scores

Outcome	Comparison	% Change from baseline
WOMAC pain	**BE vs. P**	**29.695**
	CT vs. AC	1.495
	CT + AC vs. AC	4.475
WOMAC function	**BE vs. P**	**54.424**
	CT vs. AC	4.242
	CT + AC vs. AC	4.875
VAS	BE vs. P	28.530
	**BE + AC vs. AC**	**69.555**
	BE vs. AC	0.000
	CT + AC vs. AC	13.846
	**PLS vs. P**	**41.484**

**Abbreviation**: AC, active drug comparator; BE, bioavailability-enhanced curcuminoid preparations; BE + AC, bioavailability-enhanced curcuminoid preparations + active drug comparator; CT, conventional curcuminoid preparations; CT + AC, conventional curcuminoid preparations + active drug comparator; P, placebo; PLS, polysaccharide preparations

### Secondary outcomes

For WOMAC stiffness, none of the interventions showed any significant improvement compared to placebo and among other interventions. For WOMAC function, significant improvement was seen with all interventions compared to placebo. However, no significant difference was found in other comparisons. The SUCRA ranking of WOMAC function outcome among interventions was CT + AC > AC > CT > BE > P. For VAS pain, all interventions significantly improved VAS compared to placebo. Additionally, the combination of turmeric preparation and active drug (BE + AC, and CT + AC) was found to significantly reduce VAS compared to either AC or BE alone. All effect sizes and the ranking can be seen in the Fig. 3. For the MCID evaluation of WOMAC function, the % change from baseline for BE vs. P exceeded the MCID cut-point. In addition, PLS vs. P, and BE + AC vs. AC exceeded the MCID cut-point for VAS pain (Table 3). The details of the SUCRA ranking, adjusted funnel plot, and transitivity assessment of all outcomes are shown in Appendix **I**-**K** (see Additional file 1).

### Safety outcome

Safety outcomes of each intervention were descriptively tabulated in the Appendix **L**, Additional file 1. The PLS group had the highest percentage of AEs (30.30%), with 18.18% originating from miscellaneous symptoms. GI symptoms were the second most common AE. Among GI symptoms, BE preparation reported the highest incidence (6.54%), followed by PLS (6.06%).

### Certainty of evidence

The assessment of the certainty of direct estimates resulted in moderate certainty for most outcomes, due to serious imprecision alone. The indirect estimate yielded low-certainty evidence for all outcomes, attributed to very serious imprecision. Moreover, the network estimate indicated low and very low certainty for all statistically significant outcomes, largely due to serious imprecision and incoherence. Details of the certainty of evidence are illustrated in Appendix **M** (see Additional file 1).

### Subgroup analyses

A subgroup analysis was planned to evaluate the impact of dose on efficacy outcomes, given the variability in turmeric preparation doses, particularly in BE group. Of the 7 included studies, 5 utilized BE preparations, and 2 employed CT preparations. Human pharmacokinetic data were available for 6 studies, with detected variety of analytes varying such as curcumin only, curcumin + derivatives, curcuminoids only, curcuminoids + derivatives, complicating direct comparisons and limiting further specific analyses on dose. Additional analyses were planned for BMI (limited by missing data in 2 studies, with the non-obese group constituting only 3.58% of the total population), duration of treatment (limited by including only 1 study which exceeded 12-week cut-point for long-term effects), and ethnicity (restricted by Asian predominance).

### Sensitivity analyses

Sensitivity analyses were prespecified and conducted based on the follow-up period of more than 28 days, studies with high risk of bias, and small studies effect. Finally, the results of all sensitivity analyses were consistent with the main analysis (see Appendix **N**, Additional file 1). Additionally, a sensitivity analysis assessed the impact of baseline pain intensity differences by excluding the highest baseline intensity comparison. As a result, the CT + AC vs. AC comparison was excluded for WOMAC pain and function, and the BE + AC vs. AC comparison for VAS, causing SUCRA rankings to shift, with the previously second-ranked interventions becoming top-ranked. For WOMAC stiffness, excluding CT + AC did not alter SUCRA ranking, as BE remained first. Detailed results are presented in Appendix **N** (see Additional file 1).

## Discussion

Our study is the first to employ an NMA to evaluate the efficacy and safety of diverse turmeric preparations for knee OA. This issue is important because different products possess different phytochemical compositions and also different processing techniques which result in different pharmacodynamics and pharmacokinetics properties of bioactive compounds and products. By classifying interventions based on both factors, it could provide evidence-based insights into differences in efficacy and safety of different turmeric preparations on knee OA.

Our study suggested that all turmeric interventions significantly relieved pain and improved function compared to placebo. Among all interventions, BE was the most reliable, having the highest number of head-to-head comparisons with placebo, which resulted in the greatest accuracy of both direct and indirect evidence for this intervention. Furthermore, BE preparations also provided both pain relief and improved function at the magnitude that clearly exceeded the MCID, compared to placebo. A considerable amount of experimental studies corroborate our findings that turmeric may provide benefits in knee OA. Several bioactive substances in turmeric, especially curcuminoids, have been shown to provide comprehensive anti-inflammatory and antioxidant properties. Through mechanisms such as inhibiting cytokines and suppressing NFκβ activation, curcumin effectively mitigates inflammation in OA joints [6]. Curcuminoids also modulate signaling pathways like Toll-like receptor 4 (TLR4), leading to reduced expression of matrix-degrading enzymes, pro-inflammatory factors, and inhibiting cyclooxygenase 2 (COX-2) and lipoxygenase (LOX) [40, 41]. Additionally, it exhibits antioxidant effectiveness by scavenging free oxygen species and activating the Nrf2 pathway [42]. This multifaceted action addresses both inflammation and oxidative damage. In addition, the tumerosaccharide-containing preparations are known to reduce inflammation by attenuating interleukin-1 beta (IL-1β) activity, inhibiting the release of proinflammatory cytokines, and inflammatory mediators like nitric oxide and prostaglandin E2 [43]. Among the various polysaccharides found in the polar extract of turmeric, ukonans (A, B, C, D) are identified as one group of polysaccharides [10].

Despite being composed mostly of indirect evidence, the NMA also suggested that turmeric interventions used as an add-on therapy to an active comparator outperformed both turmeric monotherapy and the active comparator alone in pain reduction and functional improvement, according to SUCRA ranking. Specifically, CT + AC ranked first for WOMAC pain, and BE + AC followed by CT + AC ranked top for VAS pain reduction. In terms of functional improvement, CT + AC combination therapy also secured the top rank in SUCRA. These findings are consistent with the previous meta-analysis [17]. The synergistic effect likely arises from the complementary mechanisms of action between turmeric’s bioactive compounds, especially curcumin, and NSAIDs. Curcumin downregulates COX-2 mRNA, while NSAIDs like diclofenac inhibit COX-2 receptor [44].

The comparative efficacy of various turmeric preparations was performed during the step of SUCRA ranking. However, the comparison between BE preparations versus CT was still inconclusive. Although BE formulations are designed to enhance curcumin bioavailability ranging from almost 7 to 20 times that of unformulated curcumin, a lack of comprehensive pharmacokinetic data limits our ability to determine the exact dose of curcumin absorbed into the bloodstream. In some BE studies included in our analysis, participants received lower doses compared to CT studies, potentially resulting in similar or even lower curcumin absorption, consequently, leaving the efficacy comparison inconclusive. Additionally, the top-ranking preparations in the SUCRA displayed higher baseline pain levels, suggesting that baseline conditions may have influenced the results rather than the efficacy of the formulations themselves. Therefore, while BE is theoretically expected to outperform CT, the current evidence is still insufficient to draw definitive conclusions.

Regarding safety, turmeric has been widely used as a food ingredient since ancient times and is generally considered to have a wide safety margin. However, we noticed an appreciable incidence of AEs with PLS and BE preparations, particularly as GI symptoms. Interestingly, the reliability of AE incidence was still limited due to the variability in AE definitions among the included studies and the spontaneous reporting nature, which is prone to missing data. However, the AEs appear to be mild and do not lead to serious consequences. Long-term monitoring may be required to better understand the safety profiles of various turmeric preparations since knee OA typically requires long-term treatment.

Regarding transitivity considerations, a sensitivity analysis of baseline intensity shows high baseline intensity biases ranking for pain and function outcomes, especially for CT + AC and BE + AC combinations. For WOMAC stiffness, excluding the highest baseline comparison did not affect BE’s top ranking, indicating stability. However, the primary effect estimates remained consistent, and the network remained connected, supporting the robustness of the findings. Future studies should account for variations in baseline pain intensity between trials as a potential confounding factor to avoid biased results.

Our study has several limitations. First, the detection of global inconsistency and the prevalence of indirect comparisons necessitated the use of an inconsistency model. However, the reliability of consistency test might be limited in the case of open-geometry network map and we were unable to explore further with other statistical techniques (loop-specific or node-splitting technique) in this setting. Furthermore, the predominance of single studies with small sample sizes resulted in wide confidence intervals. Further high-quality, head-to-head, clinical studies are needed to confirm these findings although the NMA results were aligned with those of the pairwise meta-analysis. Second, attempts to conduct a subgroup analysis on turmeric doses were hindered by high variability in doses, particularly among bioavailability-enhanced (BE) preparations. Future research should focus on dose standardization and comprehensive pharmacokinetic studies to better understand the relationship between administered doses, plasma concentrations, and therapeutic outcomes. Third, limited data availability also restricted comprehensive subgroup analyses, and the inability to analyze adverse events further detracts from the findings. Fourth, transitivity assessment suggested that baseline intensity and treatment duration may have influenced the observed effects, indicating a need for careful interpretation of the results. Finally, a significant limitation is the calculation of the minimum clinically important difference (MCID), which can only be evaluated when direct head-to-head trials are available. Unfortunately, for several comparisons in our analysis, direct head-to-head data were lacking, allowing MCID calculations for only a few pairs. BE is the only preparation with head-to-head data that allows MCID calculation against placebo, ensuring clinical significance.

## Conclusions

All turmeric preparations have been shown to significantly reduce pain and improve function in knee OA patients, compared with placebo. These preparations showed promising potential when used in combination with active drugs, leading to larger pain reduction and functional improvement, compared to monotherapy. However, the certainty of evidence was low to very low for most network estimates, requiring cautious interpretation. Future head-to-head, well-designed, long-term studies are necessary to ensure the long-term efficacy and safety of turmeric interventions for knee OA. Additionally, the relationship between dosage used and observed effects remains a critical area for future investigations.

## Supplementary Information

Below is the link to the electronic supplementary material.

Supplementary Material 1

Supplementary Material 2

## Data Availability

Data is provided within the manuscript or supplementary information files.

## Abbreviations

AC: Active drug comparators
BE: Bioavailability-enhanced curcuminoid preparations
CT: Conventional curcuminoid preparations
MCID: Minimum clinically important difference
NMA: Network meta-analysis
OA: Osteoarthritis
P: Placebo
PLS: Polysaccharide preparations
PRISMA-NMA: Preferred Reporting Items for Systematic Reviews and Meta-Analyses Extension for Network Meta-analysis
SUCRA: Surface under the cumulative ranking curve
VAS: Visual analog scale
WOMAC: Western Ontario and McMaster Universities Osteoarthritis Index
