# Supplementary material for: Effect of turmeric products on knee osteoarthritis: a systematic review and network meta-analysis
Source: BMC Complement Med Ther. 2025 Jul 29;25:292. doi: 10.1186/s12906-025-05045-z (PMC12309109; doi:10.1186/s12906-025-05045-z)
Supplement: Supplementary file 1 — Supplementary Material 1 [file 12906_2025_5045_MOESM1_ESM.pdf]

Supplementary Material

Online Supplementary Content

Contents

Appendix A 8

PRISMA NMA checklist 8

eTable A1 PRISMA NMA Checklist of Items to Include When Reporting A Systematic Review Involving a Network Meta-analysis 8

Appendix B 12

Unit standardization 12

eTable B1 Different units standardized to Likert units for WOMAC pain 12

eTable B2 Different units standardized to Likert units for WOMAC stiffness 14

eTable B3 Different units standardized to Likert units for WOMAC function 15

eTable B4 Different units standardized to 0–100 units for VAS 16

Appendix C 17

Search algorithms 17

eTable C1 PICO domains and search terms 17

eTable C2 Search algorithms 18

Appendix D 20

Additional characteristics of all included studies 20

eTable D1 Regimen characteristics of included studies 20

eTable D2 Baseline and follow-up characteristics of all included studies 24

eTable D3 Extracted data of outcomes of interest 27

eTable D4 Detailed characteristics of curcuminoid preparations included in the analysis 30

Appendix E 31

Risk of bias assessment 31

eFigure E Risk of bias graph 31

eTable E Summarized risk of bias of included studies using ROB2.0 32

Appendix F 33

Results of meta-analyses of direct comparisons of treatment options 33

eFigure F1 Mean difference (and 95% CI) for WOMAC pain 34

eFigure F2 Mean difference (and 95% CI) for WOMAC stiffness 35

eFigure F3 Mean difference (and 95% CI) for WOMAC function 36

eFigure F4 Mean difference (and 95% CI) for VAS 37

Appendix G 39

Assessment of global inconsistency for each outcome network in main analysis 39

eTable G Assessment of global inconsistency in networks using the ‘design-by-treatment’ interaction model 39

Appendix H 40

Interval plots 40

eFigure H1 Interval plot of WOMAC pain 40

eFigure H2 Interval plot of WOMAC stiffness 41

eFigure H3 Interval plot of WOMAC function 42

eFigure H4 Interval plot of VAS 43

Appendix I 44

SUCRA ranking for WOMAC Pain, Stiffness, Function, and VAS 44

eFigure I1 SUCRA ranking among interventions on WOMAC pain 44

eFigure I2 SUCRA ranking among interventions on WOMAC stiffness 45

eFigure I3 SUCRA ranking among interventions on WOMAC function 46

eFigure I4 SUCRA ranking among interventions on VAS 47

Appendix J 48

Adjusted funnel plots 48

eFigure J1 Adjusted funnel plot for WOMAC pain 48

eFigure J2 Adjusted funnel plot for WOMAC stiffness 49

eFigure J3 Adjusted funnel plot for WOMAC function 50

eFigure J4 Adjusted funnel plot for VAS 51

Appendix K 52

Transitivity assessment tables 52

eTable K1 Descriptive table for the transitivity assessment of potential effect modifiers on WOMAC pain outcome 52

eTable K2 Descriptive table for the transitivity assessment of potential effect modifiers on WOMAC stiffness outcome 53

eTable K3 Descriptive table for the transitivity assessment of potential effect modifiers on WOMAC function outcome 54

eTable K4 Descriptive table for the transitivity assessment of potential effect modifiers on VAS outcome 55

Appendix L 56

Adverse events 56

eTable L Frequency of adverse events across different treatment groups 56

Appendix M 58

Quality of evidence 58

eTable M Grading 58

Appendix N 61

Sensitivity analyses 61

eFigure N1 Sensitivity analysis of follow-up period for WOMAC pain 61

eTable N1 Descriptive table for transitivity assessment of sensitivity analysis on follow-up period for WOMAC pain 62

eFigure N2 Sensitivity analysis of follow-up period for WOMAC stiffness 63

eTable N2 Descriptive table for transitivity assessment of sensitivity analysis on follow-up period for WOMAC stiffness 64

eFigure N3 Sensitivity analysis of follow-up period for WOMAC function....65
eTable N3 Descriptive table for transitivity assessment of sensitivity analysis on follow-up period for WOMAC function....66
eFigure N4 Sensitivity analysis of critical risk of bias for WOMAC pain....67
eTable N4 Descriptive table for transitivity assessment of sensitivity analysis on critical risk of bias for WOMAC pain....68
eFigure N5 Sensitivity analysis of critical risk of bias for WOMAC stiffness....69
eTable N5 Descriptive table for transitivity assessment of sensitivity analysis on critical risk of bias for WOMAC stiffness....70
eFigure N6 Sensitivity analysis of critical risk of bias for WOMAC function....71
eTable N6 Descriptive table for transitivity assessment of sensitivity analysis on critical risk of bias for WOMAC function....72
eFigure N7 Sensitivity analysis of critical risk of bias for VAS....73
eTable N7 Descriptive table for transitivity assessment of sensitivity analysis on critical risk of bias for VAS....74
eFigure N8 Sensitivity analysis of small-study effects for WOMAC pain....75
eTable N8 Descriptive table for transitivity assessment of sensitivity analysis on small-study effects for WOMAC pain....76
eFigure N9 Sensitivity analysis of small-study effects for WOMAC stiffness....77
eTable N9 Descriptive table for transitivity assessment of sensitivity analysis on small-study effects for WOMAC stiffness....78
eFigure N10 Sensitivity analysis of small-study effects for WOMAC function....79
eTable N10 Descriptive table for transitivity assessment of sensitivity analysis on small-study effects for WOMAC function....80
eFigure N11 Sensitivity analysis of small-study effects for VAS....81
eTable N11 Descriptive table for transitivity assessment of sensitivity analysis on small-study effects for VAS....82

eFigure N12 Adjusted funnel plot of small-study effects for WOMAC pain 83
eFigure N13 Adjusted funnel plot of small-study effects for WOMAC stiffness 84
eFigure N14 Adjusted funnel plot of small-study effects for WOMAC function 85
eFigure N15 Adjusted funnel plot of small-study effects for VAS 86
Appendix O 87
References 87

Appendix A

PRISMA NMA checklist

eTable A1 PRISMA NMA Checklist of Items to Include When Reporting A Systematic Review Involving a Network Meta-analysis

Section/Topic	Item #	Checklist Item	Reported on Page #
**TITLE**			
Title	1	Identify the report as a systematic review *incorporating a network meta-analysis (or related form of meta-analysis).*	1
**ABSTRACT**			
Structured summary	2	Provide a structured summary including, as applicable:<br>**Background:** main objectives<br>**Methods:** data sources; study eligibility criteria, participants, and interventions; study appraisal; and *synthesis methods, such as network meta-analysis.*<br>**Results:** number of studies and participants identified; summary estimates with corresponding confidence/credible intervals; *treatment rankings may also be discussed. Authors may choose to summarize pairwise comparisons against a chosen treatment included in their analyses for brevity.*<br>**Discussion/Conclusions:** limitations; conclusions and implications of findings.<br>**Other:** primary source of funding; systematic review registration number with registry name.	2
**INTRODUCTION**			
Rationale	3	Describe the rationale for the review in the context of what is already known, *including mention of why a network meta-analysis has been conducted.*	4-5
Objectives	4	Provide an explicit statement of questions being addressed, with reference to participants, interventions, comparisons, outcomes, and study design (PICOS).	5
**METHODS**			

Protocol and registration	5	Indicate whether a review protocol exists and if and where it can be accessed (e.g., Web address); and, if available, provide registration information, including registration number.	2,5
Eligibility criteria	6	Specify study characteristics (e.g., PICOS, length of follow-up) and report characteristics (e.g., years considered, language, publication status) used as criteria for eligibility, giving rationale. *Clearly describe eligible treatments included in the treatment network, and note whether any have been clustered or merged into the same node (with justification).*	6-7
Information sources	7	Describe all information sources (e.g., databases with dates of coverage, contact with study authors to identify additional studies) in the search and date last searched.	5
Search	8	Present full electronic search strategy for at least one database, including any limits used, such that it could be repeated.	10
Study selection	9	State the process for selecting studies (i.e., screening, eligibility, included in systematic review, and, if applicable, included in the meta-analysis).	5
Data collection process	10	Describe method of data extraction from reports (e.g., piloted forms, independently, in duplicate) and any processes for obtaining and confirming data from investigators.	6
Data items	11	List and define all variables for which data were sought (e.g., PICOS, funding sources) and any assumptions and simplifications made.	10
**Geometry of the network**	**S1**	Describe methods used to explore the geometry of the treatment network under study and potential biases related to it. This should include how the evidence base has been graphically summarized for presentation, and what characteristics were compiled and used to describe the evidence base to readers.	8
Risk of bias within individual studies	12	Describe methods used for assessing risk of bias of individual studies (including specification of whether this was done at the study or outcome level), and how this information is to be used in any data synthesis.	6
Summary measures	13	State the principal summary measures (e.g., risk ratio, difference in means). *Also describe the use of additional summary measures assessed, such as treatment rankings and surface under the cumulative ranking curve (SUCRA) values, as well as modified approaches used to present summary findings from meta-analyses.*	9
Planned methods of analysis	14	Describe the methods of handling data and combining results of studies for each network meta-analysis. This should include, but not be limited to:<br>• *Handling of multi-arm trials;*<br>• *Selection of variance structure;*<br>• *Selection of prior distributions in Bayesian analyses; and*<br>• *Assessment of model fit.*	6,8-9
**Assessment of Inconsistency**	**S2**	Describe the statistical methods used to evaluate the agreement of direct and indirect evidence in the treatment network(s) studied. Describe efforts taken to address its presence when found.	9

Risk of bias across studies	15	Specify any assessment of risk of bias that may affect the cumulative evidence (e.g., publication bias, selective reporting within studies).	9
Additional analyses	16	Describe methods of additional analyses if done, indicating which were pre-specified. This may include, but not be limited to, the following:<br>• Sensitivity or subgroup analyses;<br>• Meta-regression analyses;<br>• *Alternative formulations of the treatment network; and*<br>• *Use of alternative prior distributions for Bayesian analyses (if applicable).*	9
**RESULTS†**			
Study selection	17	Give numbers of studies screened, assessed for eligibility, and included in the review, with reasons for exclusions at each stage, ideally with a flow diagram.	11
**Presentation of network structure**	**S3**	Provide a network graph of the included studies to enable visualization of the geometry of the treatment network.	14
**Summary of network geometry**	**S4**	Provide a brief overview of characteristics of the treatment network. This may include commentary on the abundance of trials and randomized patients for the different interventions and pairwise comparisons in the network, gaps of evidence in the treatment network, and potential biases reflected by the network structure.	11-12
Study characteristics	18	For each study, present characteristics for which data were extracted (e.g., study size, PICOS, follow-up period) and provide the citations.	32-33
Risk of bias within studies	19	Present data on risk of bias of each study and, if available, any outcome level assessment.	12
Results of individual studies	20	For all outcomes considered (benefits or harms), present, for each study: 1) simple summary data for each intervention group, and 2) effect estimates and confidence intervals. *Modified approaches may be needed to deal with information from larger networks.*	12
Synthesis of results	21	Present results of each meta-analysis done, including confidence/credible intervals. *In larger networks, authors may focus on comparisons versus a particular comparator (e.g. placebo or standard care), with full findings presented in an appendix. League tables and forest plots may be considered to summarize pairwise comparisons.* If additional summary measures were explored (such as treatment rankings), these should also be presented.	15

**Exploration for inconsistency**	**S5**	Describe results from investigations of inconsistency. This may include such information as measures of model fit to compare consistency and inconsistency models, *P* values from statistical tests, or summary of inconsistency estimates from different parts of the treatment network.	12-13
Risk of bias across studies	22	Present results of any assessment of risk of bias across studies for the evidence base being studied.	12
Results of additional analyses	23	Give results of additional analyses, if done (e.g., sensitivity or subgroup analyses, meta-regression analyses, *alternative network geometries studied, alternative choice of prior distributions for Bayesian analyses,* and so forth).	18
**DISCUSSION**			
Summary of evidence	24	Summarize the main findings, including the strength of evidence for each main outcome; consider their relevance to key groups (e.g., healthcare providers, users, and policy-makers).	18
Limitations	25	Discuss limitations at study and outcome level (e.g., risk of bias), and at review level (e.g., incomplete retrieval of identified research, reporting bias). *Comment on the validity of the assumptions, such as transitivity and consistency. Comment on any concerns regarding network geometry (e.g., avoidance of certain comparisons).*	20
Conclusions	26	Provide a general interpretation of the results in the context of other evidence, and implications for future research.	21
**FUNDING**			
Funding	27	Describe sources of funding for the systematic review and other support (e.g., supply of data); role of funders for the systematic review. This should also include information regarding whether funding has been received from manufacturers of treatments in the network and/or whether some of the authors are content experts with professional conflicts of interest that could affect use of treatments in the network.	23

Appendix B

Unit standardization

In standardization outcome scales across diverse trials, established conversion formulas were employed to rescale mean responses and SDs from the original instrument (B) to the units of the more familiar instrument (A). The converted mean estimates were derived by adjusting the original mean and SD using the ranges of the respective instruments [1].

mA = (mB – LB) (RA/RB) + LA; RA = UA – LA; RB = UB – LB

eTable B1 Different units standardized to Likert units for WOMAC pain

Author, year	Treatment	n	mB	SDB	UB	LB	UA	LA	RA	RB	mA	SDA
Singhal, et al. 2021 [2]	BE	73	8.78	4.87	20	0	20	0	20	20	8.78	4.87
Singhal, et al. 2021 [2]	AC	71	7.92	4.13	20	0	20	0	20	20	7.92	4.13
Haroyan, et al. 2018 [3]	BE	58	3.84	2.88	20	0	20	0	20	20	3.84	2.88
Haroyan, et al. 2018 [3]	P	59	5.22	3.58	20	0	20	0	20	20	5.22	3.58
Hashemzadeh, et al. 2020 [4]	BE	36	14.53	7.18	50	5	20	0	20	45	4.24	3.19
Hashemzadeh, et al. 2020 [4]	P	35	21.23	7.11	50	5	20	0	20	45	7.21	3.16
Panda, et al. 2018 [5]	BE	25	4.28	1.54	20	0	20	0	20	20	4.28	1.54
Panda, et al. 2018 [5]	P	25	6.96	1.43	20	0	20	0	20	20	6.96	1.43
Panahi, et al. 2014 [6]	BE	19	6.1	2.9	20	0	20	0	20	20	6.10	2.90
Panahi, et al. 2014 [6]	P	21	9.4	3.4	20	0	20	0	20	20	9.40	3.40
Srivastava, et al. 2016 [7]	CT+AC	78	9.48	1.50	20	0	20	0	20	20	9.48	1.50
Srivastava, et al. 2016 [7]	AC	82	10.16	1.45	20	0	20	0	20	20	10.16	1.45
Kuptniratsaikul, et al. 2014 [8]	CT	171	3.25	2.11	10	0	20	0	20	10	6.50	4.22
Kuptniratsaikul, et al. 2014 [8]	AC	160	3.17	1.98	10	0	20	0	20	10	6.34	3.96

Abbreviation: A, the desired instrument (Likert) with the range 0–20 for WOMAC pain; B, the original instrument; LA, lower limit of instrument A; LB, lower limit of instrument B; mA, the standardized mean (Likert); mB, the original mean; RA, range of desired

instrument (Likert) 0–20; RB, range of original instrument; SDA, the standardized standard deviation (Likert); SDB, the original standard deviation; UA, upper limit of instrument A; UB, lower limit of instrument B

eTable B2 Different units standardized to Likert units for WOMAC stiffness

**Author, year**	**Treatment**	**n**	**mB**	**SDB**	**UB**	**LB**	**UA**	**LA**	**RA**	**RB**	**mA**	**SDA**
Singhal, et al. 2021 [2]	BE	73	3.01	2.05	8	0	8	0	8	8	3.01	2.05
Singhal, et al. 2021 [2]	AC	71	3.61	1.77	8	0	8	0	8	8	3.61	1.77
Hashemzadeh, et al. 2020 [4]	BE	36	2.64	1.93	20	2	8	0	8	18	0.28	0.86
Hashemzadeh, et al. 2020 [4]	P	35	2.94	2.04	20	2	8	0	8	18	0.42	0.91
Panda, et al. 2018 [5]	BE	25	2.12	0.97	8	0	8	0	8	8	2.12	0.97
Panda, et al. 2018 [5]	P	25	3.76	1.09	8	0	8	0	8	8	3.76	1.09
Panahi, et al. 2014 [6]	BE	19	0.15	0.5	8	0	8	0	8	8	0.15	0.50
Panahi, et al. 2014 [6]	P	21	0.76	0.9	8	0	8	0	8	8	0.76	0.90
Srivastava, et al. 2016 [7]	CT + AC	78	4.08	1.50	8	0	8	0	8	8	4.08	1.50
Srivastava, et al. 2016 [7]	AC	82	4.16	1.63	8	0	8	0	8	8	4.16	1.63
Kuptniratsaikul, et al. 2014 [8]	CT	171	3.28	2.38	10	0	8	0	8	10	2.62	1.90
Kuptniratsaikul, et al. 2014 [8]	AC	160	3.16	2.36	10	0	8	0	8	10	2.53	1.89

Abbreviation: A, the desired instrument (Likert) with the range 0–8 for WOMAC stiffness; B, the original instrument; LA, lower limit of instrument A; LB, lower limit of instrument B; mA, the standardized mean (Likert); mB, the original mean; RA, range of desired instrument (Likert) 0–8; RB, range of original instrument; SDA, the standardized standard deviation (Likert); SDB, the original standard deviation; UA, upper limit of instrument A; UB, lower limit of instrument B

eTable B3 Different units standardized to Likert units for WOMAC function

Author, year	Treatment	n	mB	SDB	UB	LB	UA	LA	RA	RB	mA	SDA
Singhal, et al. 2021 [2]	BE	73	31.22	16.23	68	0	68	0	68	68	31.22	16.23
Singhal, et al. 2021 [2]	AC	71	26.41	13.82	68	0	68	0	68	68	26.41	13.82
Hashemzadeh, et al. 2020 [4]	BE	36	42.61	16.7	170	17	68	0	68	153	11.38	7.42
Hashemzadeh, et al. 2020 [4]	P	35	69.51	27.56	170	17	68	0	68	153	23.34	12.25
Panda, et al. 2018 [5]	BE	25	12.04	3.12	68	0	68	0	68	68	12.04	3.12
Panda, et al. 2018 [5]	P	25	20.04	3.77	68	0	68	0	68	68	20.04	3.77
Panahi, et al. 2014 [6]	BE	19	18.7	10.3	68	0	68	0	68	68	18.70	10.30
Panahi, et al. 2014 [6]	P	21	30.4	9.4	68	0	68	0	68	68	30.40	9.40
Srivastava, et al. 2016 [7]	CT + AC	78	32.14	3.53	68	0	68	0	68	68	32.14	3.53
Srivastava, et al. 2016 [7]	AC	82	33.88	4.53	68	0	68	0	68	68	33.88	4.53
Kuptniratsaikul, et al. 2014 [8]	CT	171	3.41	2.09	10	0	68	0	68	10	23.19	14.21
Kuptniratsaikul, et al. 2014 [8]	AC	160	3.26	2.05	10	0	68	0	68	10	22.17	13.94

Abbreviation: A, the desired instrument (Likert) with the range 0–68 for WOMAC function; B, the original instrument; LA, lower limit of instrument A; LB, lower limit of instrument B; mA, the standardized mean (Likert); mB, the original mean; RA, range of desired instrument (Likert) 0–68; RB, range of original instrument; SDA, the standardized standard deviation (Likert); SDB, the original standard deviation; UA, upper limit of instrument A; UB, lower limit of instrument B

eTable B4 Different units standardized to 0–100 units for VAS

Author, year	Treatment	n	mB	SDB	UB	LB	UA	LA	RA	RB	mA	SDA
Shep, et al. 2019 [9]	BE	70	2.2	0.81	10	0	100	0	100	10	22	8.10
Shep, et al. 2019 [9]	AC	69	2.2	0.61	10	0	100	0	100	10	22	6.10
Atabaki, et al. 2020 [10]	BE + AC	15	3.4	1.05	10	0	100	0	100	10	34	10.46
Atabaki, et al. 2020 [10]	AC	15	9.1	0.97	10	0	100	0	100	10	91	9.68
Panda, et al. 2018 [5]	BE	25	27.26	11.95	100	0	100	0	100	100	27.26	11.95
Panda, et al. 2018 [5]	P	25	44.83	4.27	100	0	100	0	100	100	44.83	4.27
Panahi, et al. 2014 [6]	BE	19	37	17	100	0	100	0	100	100	37	17.00
Panahi, et al. 2014 [6]	P	21	57	14	100	0	100	0	100	100	57	14.00
*Henrotin, et al. 2019 [11]	BE	86	37.43	23.95	100	0	100	0	100	100	37.43	23.95
Henrotin, et al. 2019 [11]	P	40	47.95	27.29	100	0	100	0	100	100	47.95	27.29
Srivastava, et al. 2016 [7]	CT + AC	78	4.03	0.71	10	0	100	0	100	10	40.3	7.07
Srivastava, et al. 2016 [7]	AC	82	5.11	1.27	10	0	100	0	100	10	51.1	12.68
Madhu, et al. 2013 [12]	PLS	29	19.48	17.84	100	0	100	0	100	100	19.48	17.84
Madhu, et al. 2013 [12]	P	29	46.03	20.84	100	0	100	0	100	100	46.03	20.84

Abbreviation: A, the desired instrument with the range 0–100 for VAS; B, the original instrument; LA, lower limit of instrument A; LB, lower limit of instrument B; mA, the standardized mean; mB, the original mean; RA, range of desired instrument 0–100; RB, range of original instrument; SDA, the standardized standard deviation; SDB, the original standard deviation; UA, upper limit of instrument A; UB, lower limit of instrument B

*Means and standard deviations of two intervention groups combined into a single intervention group

Appendix C

Search algorithms

The PICO format was used during the searching step.

eTable C1 PICO domains and search terms

**Domain**	**Search terms**
P	Knee osteoarthritis
I	Any turmeric preparations
C	Active control (Standard pharmacological treatment)<br>Placebo
O	Pain reduction<br>Stiffness<br>Function<br>Adverse events

eTable C2 Search algorithms

Database	Step	Search algorithm	Items found
**PubMed**	#1	(osteoarthritis) OR (OA)	137,383
	#2	((((turmeric) OR (curcumin)) OR (curcuma)) OR (turmer*)) OR (curcum*)	26,004
	#3	(Degenerative arthritis) OR (Degenerative joint disease)	120,395
	#4	((osteoarthritis) OR (OA)) OR ((Degenerative arthritis) OR (Degenerative joint disease))	142,894
	#5	(((((turmeric) OR (curcumin)) OR (curcuma)) OR (turmer*)) OR (curcum*)) AND (((osteoarthritis) OR (OA)) OR ((Degenerative arthritis) OR (Degenerative joint disease)))	332
	#6	(((((turmeric) OR (curcumin)) OR (curcuma)) OR (turmer*)) OR (curcum*)) AND (((osteoarthritis) OR (OA)) OR ((Degenerative arthritis) OR (Degenerative joint disease))) Filters: Clinical Trial	**48**
		New records found after updating search from Oct 2023 to Aug 2024	**2**
**Embase**	#1	turmeric OR curcumin OR (curcuma AND longa) OR (curcuma AND longa AND extract) OR (turmeric AND oil) OR turmerin	44,101
	#2	'osteoarthritis'/exp OR osteoarthritis OR oa OR 'degenerative joint disease' OR 'degenerative arthritis'	233,235
	#3	#1 AND #2	815
	#4	#1 AND #2 AND [randomized controlled trial]/lim	**71**
		New records found after updating search from Oct 2023 to Aug 2024	**4**
**Scopus**	#1	(ALL (osteoarthritis) OR ALL (OA) OR ALL (degenerative AND arthritis) OR ALL (degenerative AND joint AND disease))	715,386
	#2	(ALL (turmeric) OR ALL (curcumin) OR ALL (curcuma) OR ALL (turmer*) OR ALL (curcum*))	293,810
	#3	((ALL (osteoarthritis) OR ALL (oa) OR ALL (degenerative AND arthritis) OR ALL (degenerative AND joint AND disease))) AND ((ALL (turmeric) OR ALL (curcumin) OR ALL (curcuma) OR ALL (turmer*) OR ALL (curcum*)))	14,747

eTable C2 Search algorithms (cont.)

**Database**	**Step**	**Search algorithm**	**Items found**
	#4	((ALL (osteoarthritis) OR ALL (oa) OR ALL (degenerative AND arthritis) OR ALL (degenerative AND joint AND disease))) AND ((ALL (turmeric) OR ALL (curcumin) OR ALL (curcuma) OR ALL (turmer*) OR ALL (curcum*))) AND (EXCLUDE (DOCTYPE , "re") OR EXCLUDE (DOCTYPE , "sh") OR EXCLUDE (DOCTYPE , "ed") OR EXCLUDE (DOCTYPE , "no") OR EXCLUDE (DOCTYPE , "le") OR EXCLUDE (DOCTYPE , "tb") OR EXCLUDE (DOCTYPE , "er") OR EXCLUDE (DOCTYPE , "dp")) AND (EXCLUDE (EXACTKEYWORD , "nonhuman") OR EXCLUDE (EXACTKEYWORD , "animals") OR EXCLUDE (EXACTKEYWORD , "animal") OR LIMIT-TO (EXACTKEYWORD , "osteoarthritis"))	**477**
		New records found after updating search from Oct 2023 to Aug 2024	**84**
**ClinicalTrials.gov**	#1	Condition/disease: Osteoarthritis; Other terms: OA OR Degenerative Arthritis OR Degenerative Joint Disease; Intervention/treatment: Turmeric OR Curcumin OR Curcuminoids OR Curcuma longa OR Curcuma domestica extracts	**9**
		New records found after updating search from Oct 2023 to Aug 2024	**7**

Appendix D

Additional characteristics of all included studies

eTable D1 Regimen characteristics of included studies

Author, year	Location	Intervention used	Group	Dose/capsule (mg)	Dose/day (mg)	Regimen	Add-on dose/day (mg)	Add-on name
Shep, et al. 2019 [9]	India	BCM-95®	BE	500	1,500	1 Capsule TID		
		Diclofenac	AC	50	100	1 Tablet BID		
Singhal, et al. 2021 [2]	India	BCM-95®	BE	500	1,000	1 Capsule BID		
		Paracetamol	AC	650	1,950	1 Capsule TID		
Haroyan, et al. 2018 [3]	Armenia	CuraMed® (BCM-95)	BE	500	1,500	1 Capsule TID		
		Placebo	P	500	1,500	1 Capsule TID		
Atabaki, et al. 2020 [10]	Iran	Sinacurcumin® + Diclofenac	BE + AC	80	80	1 Capsule OD	50	Diclofenac
		Placebo + Diclofenac	P + AC	-	-	-	50	Diclofenac

eTable D1 Regimen characteristics of included studies (cont.)

Author, year	Location	Intervention used	Group	Dose/capsule (mg)	Dose/day (mg)	Regimen	Add-on dose/day (mg)	Add-on name
Hashemzadeh, et al. 2020 [4]	Iran	SinaCurcumin™	BE	40	80	1 Capsules BID		
		Placebo	P	-	-	-		
Lopresti, et al. 2021 [13]	Australia	Curcugen®	BE	500	1,000	1 capsule BID		
		Placebo	P	-	-	1 capsule BID		
Nakagawa, et al. 2014 [14]	Japan	Theracurmin®	BE	30	180	3 capsules BID		
		Placebo	P	-	-	3 capsules BID		
Panda, et al. 2018 [5]	India	Curene®	BE	500	500	1 Capsule OD		
		Placebo	P	-	-	1 Capsule OD		
Panahi, et al. 2014 [6]	Iran	C3 complex®	BE	505	1,515	1 Capsule TID		
		Placebo	P	-	-	-		

eTable D1 Regimen characteristics of included studies (cont.)

**Author, year**	**Location**	**Intervention used**	**Group**	**Dose/capsule (mg)**	**Dose/day (mg)**	**Regimen**	**Add-on dose/day (mg)**	**Add-on name**
Gupte, et al. 2019 [15]	India	Longvida®	BE	400	800	1 Capsule BID		
		Ibuprofen	AC	400	400	1 Capsule OD		
Henrotin, et al. 2019 [11]	Belgium	FLEXOFYTOL®	BE (high)	46.67	280.02	2 Capsules TID		
		FLEXOFYTOL® + Placebo	BE (low) + P	46.67	186.68	2 Capsules BID + 2 Capsule OD		
		Placebo	P	-	-	2 Capsules TID		
Pinsornsak and Niempoog 2012 [16]	Thailand	Curcuminoids capsules + Diclofenac	CT + AC	250	1,000	2 Capsules BID	75	Diclofenac
		Placebo + Diclofenac	P + AC	-	-	2 Capsules BID	75	Diclofenac
Srivastava, et al. 2016 [7]	India	*C. longa* extract registered as ‘Haridra’ + Diclofenac	CT + AC	500	1,000	1 Capsule BID	100	Diclofenac
		Placebo + Diclofenac	P + AC	-	-	-	100	Diclofenac

eTable D1 Regimen characteristics of included studies (cont.)

**Author, year**	**Location**	**Intervention used**	**Group**	**Dose/capsule (mg)**	**Dose/day (mg)**	**Regimen**	**Add-on dose/day (mg)**	**Add-on name**
Kuptniratsaikul, et al. 2009 [17]	Thailand	*C. domestica* extract	CT	500	2,000	1 Capsule QID		
		Ibuprofen	AC	400	800	1 Capsule BID		
Kuptniratsaikul, et al. 2014 [8]	Thailand	*C. domestica* extract	CT	250	1,500	2 Capsules TID		
		Ibuprofen	AC	200	1,200	2 Capsules TID		
Madhu, et al. 2013 [12]	India	Turmacin	PLS	500	1,000	1 Capsule BID		
		Placebo	P	400	800	1 Capsule BID		
Wang, et al. 2020 [18]	Australia	Turmacin Plus	PLS	500	1,000	2 Capsules OD		
		Placebo	P	-	-	2 Capsules OD		

Abbreviation: A, active drug comparator; BE, bioavailability-enhanced curcuminoid preparations; CT, conventional curcuminoid preparations; P, placebo; PLS, polysaccharide preparations

eTable D2 Baseline and follow-up characteristics of all included studies

**Author, year**	**Duration of knee OA/pain, months, mean ± SD**		**Baseline pain intensity measured by a pain measure, mean ± SD**		**Follow-up duration (days)**
	**Intervention**	**Control**	**Intervention**	**Control**	
**Shep, et al. 2019 [9]**	7.4 ±3.53	7.45 ±3.15	78.40 ±6.3[a]	78.10 ±7.3[a]	28
**Singhal, et al. 2021 [2]**	NA	NA	56.30 ±20.50[b]	50.20 ±19.50[b]	42**
**Haroyan, et al. 2018 [3]**	NA	NA	28.94 ±13.20[b]	33.37 ±15.21[b]	84**
**Atabaki, et al. 2020 [10]**	53.52 ±30.21***	56.4 ±30.21***	79.30 ±15.10[a]	84.60 ±19.36[a]	90**
**Hashemzadeh, et al. 2020 [4]**	21.69 ±9.94	24.8 ±8.52	33.44 ±14.29[b]	34.00 ±14.66[b]	42
**Lopresti, et al. 2021 [13]**	NA	NA	6.22 ±1.43[c]	5.80 ±1.56[c]	56**
**Nakagawa, et al. 2014 [14]**	NA	NA	52 ±24[a]	42 ±25[a]	56
**Panda, et al. 2018 [5]**	NA	NA	52.37 ±6.41[a]	52.79 ±4.47[a]	60
**Panahi, et al. 2014 [6]**	NA	NA	66.32 ±14.22[a]	59.05 ±17.29[a]	42**

eTable D2 Baseline and follow-up characteristics of all included studies (cont.)

**Author, year**	**Duration of knee OA/pain, months, mean ± SD**		**Baseline pain intensity measured by a pain measure, mean ± SD**		**Follow-up duration (days)**
	**Intervention**	**Control**	**Intervention**	**Control**	
**Gupte, et al. 2019 [15]**	NA	NA	80[a,d]	85[a,d]	90
**Henrotin, et al. 2019 [11]**	88.92 ±97.52[***], 79.2 ±56.052[***]	91.2 ±111.6[***]	62.9 ±13.8[a], 63.3 ±15.8[a]	59.9 ±12.3[a]	90
**Pinsornsak and Niempoog 2012 [16]**	NA	NA	55 ±NA[a]	53.1 ±NA[a]	90[**]
**Srivastava, et al. 2016 [7]**	NA	NA	79.40 ±11.48[a]	76.60 ±12.68[a]	120
**Kuptniratsaikul, et al. 2009 [17]**	19.1 ±19.6	22.3 ±26.4	5.3 ±2.3[c]	5.0 ±1.9[c]	42[**]
**Kuptniratsaikul, et al. 2014 [8]**	51.3 ±53.4	52 ±51.7	50.88 ±17.28[b]	49.92 ±16.32[b]	28[**]

eTable D2 Baseline and follow-up characteristics of all included studies (cont.)

**Author, year**	**Duration of knee OA/pain, months, mean ± SD**		**Baseline pain intensity measured by a pain measure, mean ± SD**		**Follow-up duration (days)**
	**Intervention**	**Control**	**Intervention**	**Control**	
**Madhu, et al. 2013 [12]**	NA	NA	66.5 ±21.06[a]	61.5 ±13.71[a]	42
**Wang, et al. 2020 [18]**	NA	NA	55.6 ±16.1[a]	54.4 ±17.8[a]	84**

Abbreviation: AC, active drug comparator; BE, bioavailability-enhanced curcuminoid preparations; CT, conventional curcuminoid preparations; NA, not available; P, placebo; PLS, polysaccharide preparations, ** converted from week, *** converted from year, [a] pain measured on a VAS scale (score standardized to 0–100), [b] pain measured on a WOMAC scale (total score standardized to 0–96), [c] pain measured on an NRS scale (0–10), [d] estimated data obtained from graphical presentation

For each outcome of interest except the adverse events, the final mean (the longest reported follow-up) was used. Data were extracted in mean ± standard deviation (SD). In instances where data were not presented in SDs, SDs were computed from the reported standard errors (SEs) or confidence intervals (CIs) as provided.

eTable D3 Extracted data of outcomes of interest

Author, year	Treatment	n	Final mean	Final SD
**WOMAC pain**				
Singhal, et al. 2021 [2]	BE	73	8.78	4.87*
	AC	71	7.92	4.13*
Haroyan, et al. 2018 [3]	BE	58	3.84	2.88
	P	59	5.22	3.58
Hashemzadeh, et al. 2020 [4]	BE	36	14.53	7.18
	P	35	21.23	7.11
Panda, et al. 2018 [5]	BE	25	4.28	1.54
	P	25	6.96	1.43
Panahi, et al. 2014 [6]	BE	19	6.1	2.9
	P	21	9.4	3.4
Srivastava, et al. 2016 [7]	CT+AC	78	9.48	1.50*
	AC	82	10.16	1.45*
Kuptniratsaikul, et al. 2014 [8]	CT	171	3.25	2.11
	AC	160	3.17	1.98
**WOMAC stiffness**				
Singhal, et al. 2021 [2]	BE	73	3.01	2.05*
	AC	71	3.61	1.77*
Hashemzadeh, et al. 2020 [4]	BE	36	2.64	1.93
	P	35	2.94	2.04
Panda, et al. 2018 [5]	BE	25	2.12	0.97
	P	25	3.76	1.09
Panahi, et al. 2014 [6]	BE	19	0.15	0.5
	P	21	0.76	0.9
Srivastava, et al. 2016 [7]	CT + AC	78	4.08	1.50*
	AC	82	4.16	1.63*
Kuptniratsaikul, et al. 2014 [8]	CT	171	3.28	2.38*
	AC	160	3.16	2.36*

eTable D3 Extracted data of outcomes of interest (cont.)

Author, year	Treatment	n	Final mean	Final SD
**WOMAC function**				
Singhal, et al. 2021 [2]	BE	73	31.22	16.23*
	AC	71	26.41	13.82*
Hashemzadeh, et al. 2020 [4]	BE	36	42.61	16.7
	P	35	69.51	27.56
Panda, et al. 2018 [5]	BE	25	12.04	3.12
	P	25	20.04	3.77
Panahi, et al. 2014 [6]	BE	19	18.7	10.3
	P	21	30.4	9.4
Srivastava, et al. 2016 [7]	CT + AC	78	32.14	3.53*
	AC	82	33.88	4.53*
Kuptniratsaikul, et al. 2014 [8]	CT	171	3.41	2.09*
	AC	160	3.26	2.05*
**VAS**				
Shep, et al. 2019 [9]	BE	70	2.2	0.81
	AC	69	2.2	0.61
Atabaki, et al. 2020 [10]	BE + AC	15	3.4	1.05*
	AC	15	9.1	0.97*
Panda, et al. 2018 [5]	BE	25	27.26	11.95
	P	25	44.83	4.27
Panahi, et al. 2014 [6]	BE	19	37**	17
	P	21	57**	14
Henrotin, et al. 2019 [11]	BE	86	37.43	23.95
	P	40	47.95	27.29
Srivastava, et al. 2016 [7]	CT + AC	78	4.03	0.71*
	AC	82	5.11	1.27*
Madhu, et al. 2013 [12]	PLS	29	19.48	17.84
	P	29	46.03	20.84

Abbreviation: AC, active drug comparator; BE, bioavailability-enhanced curcuminoid preparations; CT, conventional curcuminoid preparations; P, placebo; PLS, polysaccharide preparations; SD, standard deviation; *, Converted from standard error; **, estimated data obtained from graphical presentation

To avoid duplication of control arms in the NMA, the high-dose and low-dose groups from the Henrotin et al. (2019) study were combined into a single intervention group. The combined mean and standard deviation were calculated using the following formulas as stated in the Cochrane Handbook, chapter 6:

Combined sample size = $N_1 + N_2$

Combined mean = $\dfrac{N_1 M_1 + N_2 M_2}{N_1 + N_2}$

Combined standard deviation = $\sqrt{\dfrac{(N_1 - 1)SD_1^2 + (N_2 - 1)SD_2^2 + \dfrac{N_1 N_2}{N_1 + N_2}\left(M_1^2 + M_2^2 - 2M_1 M_2\right)}{N_1 + N_2 - 1}}$

Where N_1 is the sample size of the high-dose arm (48), M_1 is the mean of the high-dose arm (37.63), and SD_1 is the standard deviation of the high-dose arm (25.63). N_2 is the sample size of the low-dose arm (38), M_2 is the mean of the low-dose arm (37.18), and SD_2 is the standard deviation of the low-dose arm (21.98).

eTable D4 Detailed characteristics of curcuminoid preparations included in the analysis

**Author, year**	**Intervention**	**Technique to enhance bioavailability**	**Dose taken per day (mg)**	**Increased absorption (fold)***
Shep, et al. 2019 [9]	BCM-95®	Addition of turmeric oil	1,500	6.93
Singhal, et al. 2021 [2]			1,000	
Haroyan, et al. 2018 [3]	CuraMed® (BCM-95)		1,500	
Panahi, et al. 2014 [6]	C3 complex®	Addition of piperine	1,515	20
Panda, et al. 2018 [5]	Curene®	Formulated with proprietary Aquasome technology – for enhancing bioavailability of curcuminoids	500	NA
Henrotin, et al. 2019 [11]	FLEXOFYTOL®	Reduction in sample size (emulsifier, polysorbate 80)	280.02, 186.68	NA
Atabaki, et al. 2020 [10]	Sinacurcumin®	Reduction in particle size (nanomicelle formulation)	80	NA
Hashemzadeh, et al. 2020 [4]	SinaCurcumin™			
Srivastava, et al. 2016 [7]	*C. longa* extract registered as ‘Haridra’	None	1,000	1
Kuptniratsaikul, et al. 2014 [8]	*C. domestica* extract	None	1,500	1

* Data from the literature review [19]

Appendix E

Risk of bias assessment

eFigure E Risk of bias graph

The review authors' judgment about each risk of bias item are presented as percentages across all included studies, indicating the distribution of low, some concerns, and high risk of bias.

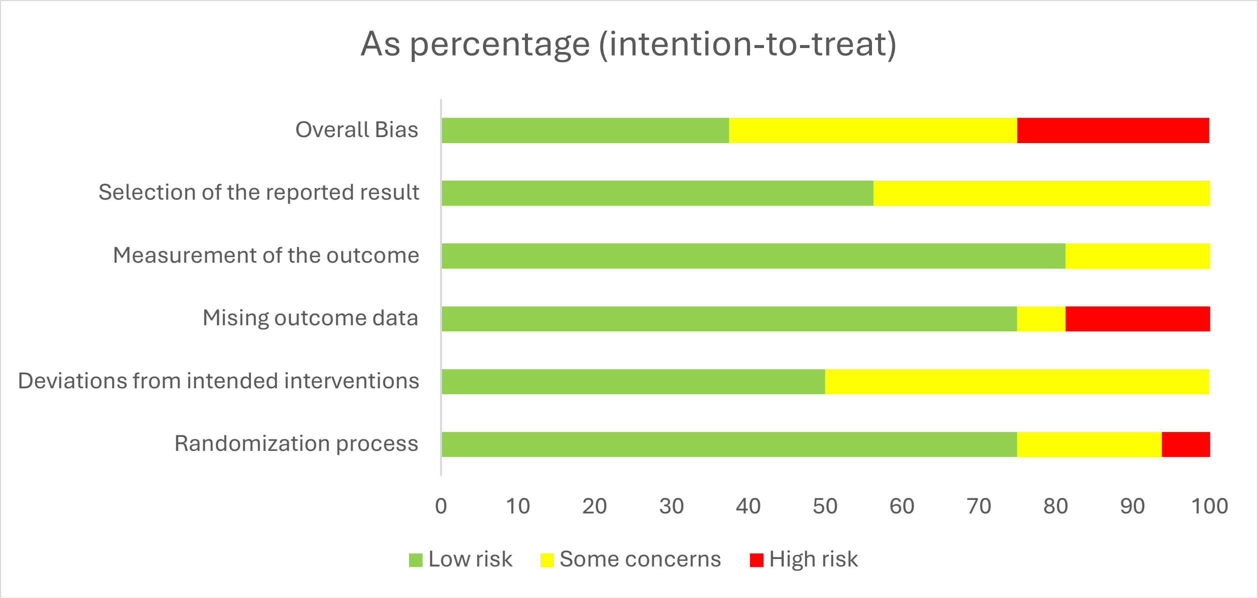

eTable E Summarized risk of bias of included studies using ROB2.0

We adhered to the recommended approach for assessing the risk of bias in studies included in Cochrane reviews. This approach encompasses six specific domains, namely the randomization process, intended intervention, missing outcome data, measurement of outcome, reported results, and overall risk of bias. Each domain consists of one or more specific entries in a 'Risk of Bias' table. The tool involves assigning a judgment regarding the risk of bias for each entry. This is accomplished by answering a pre-specified question about the adequacy of the study in relation to the entry, resulting in a judgment of low risk of bias, some concerns, or high risk of bias.

Intention-to-treat

Unique ID	Study ID	D1	D2	D3	D4	D5	Overall
1	Shep, D., et al. 2019	+	!	+	!	!	!
2	Singhal, S., et al. 2021	+	!	+	!	+	!
3	Haroyan, A., et al. 2018	+	+	+	+	+	+
4	Atabaki, M., et al. 2020	+	+	+	+	+	+
5	Hashemzadeh, K., et al. 2020	+	+	+	+	+	+
6	Lopresti, A.L., et al. 2021	+	+	+	+	+	+
7	Nakagawa, Y., et al. 2014	!	!	-	+	!	-
8	Panda, S.K., et al. 2018	+	+	!	+	+	!
9	Panahi, Y., et al. 2014	!	!	-	+	!	-
10	Gupte, P.A., et al. 2019	+	!	-	+	!	-
11	Henrotin, Y., et al. 2019	+	+	+	+	+	+
12	Pinsornsak, P., et al. 2012	!	!	+	+	!	!
13	Srivastava, S., et al. 2016	+	+	+	+	!	!
14	Kuptniratsaikul, V. et al., 2009	+	!	+	!	!	!
15	Kuptniratsaikul, V. et al., 2014	+	!	!	+	+	!
16	Madhu, K., et al. 2013	-	!	+	+	+	-
17	Wang, Z., et al. 2020	+	+	+	+	+	+

\+ Low risk
! Some concerns
\- High risk

D1 Randomisation process
D2 Deviations from the intended interventions
D3 Missing outcome data
D4 Measurement of the outcome
D5 Selection of the reported result

Appendix F

Results of meta-analyses of direct comparisons of treatment options

While eight RCTs reported WOMAC stiffness and WOMAC function outcomes [2-8, 18], Haroyan et al. 2018, and Wang et al. 2020 reported mean change data only, precluding their integration [3, 18]. For WOMAC stiffness and function outcomes, six studies (N = 796) were included in the analysis.

eFigure F1 Mean difference (and 95% CI) for WOMAC pain

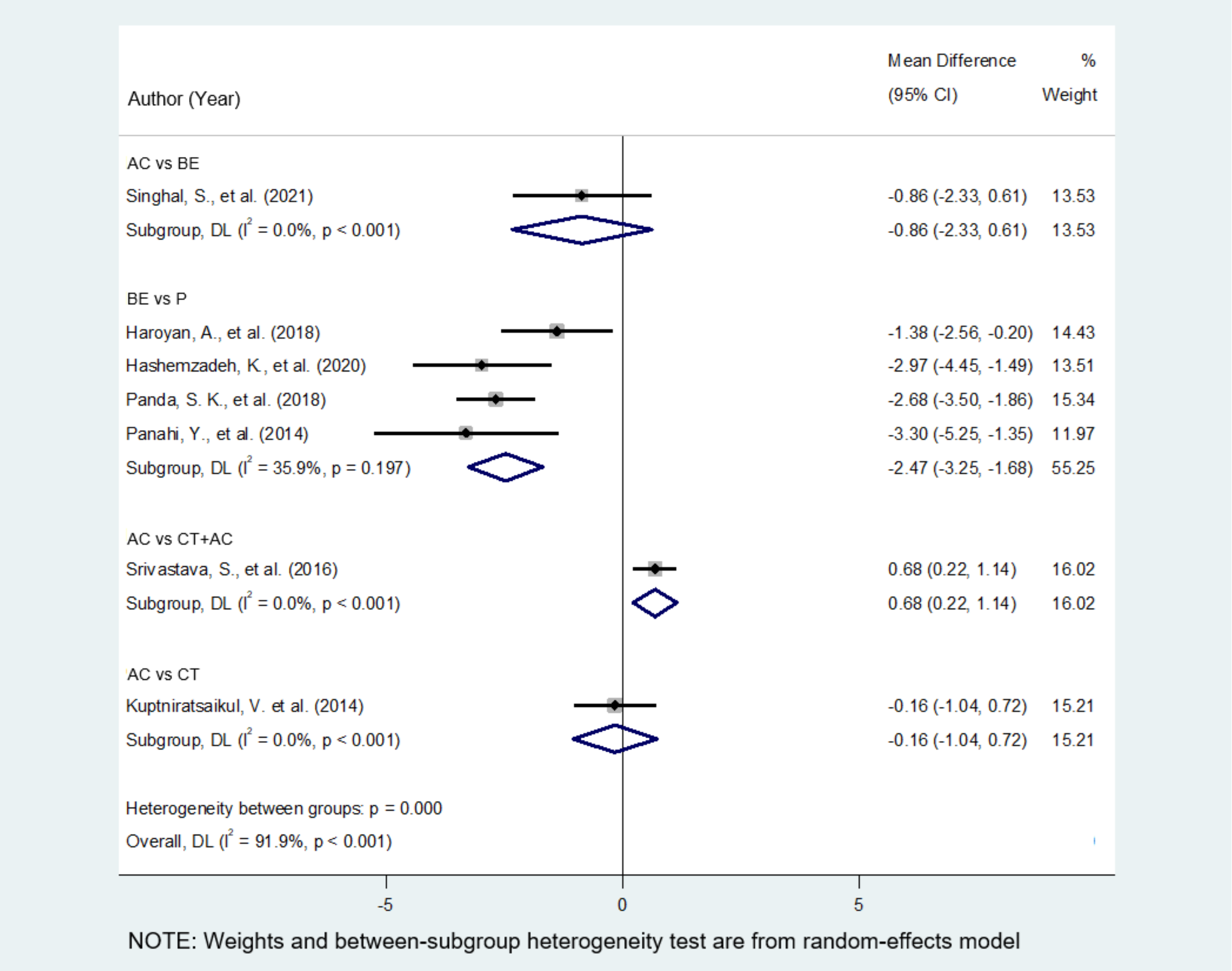

Abbreviation: CI, confidence interval; AC, active drug comparator; BE, bioavailability-enhanced curcuminoid preparations; CT, conventional curcuminoid preparations; CT + AC, conventional curcuminoid preparations + active drug comparator; P, placebo

eFigure F2 Mean difference (and 95% CI) for WOMAC stiffness

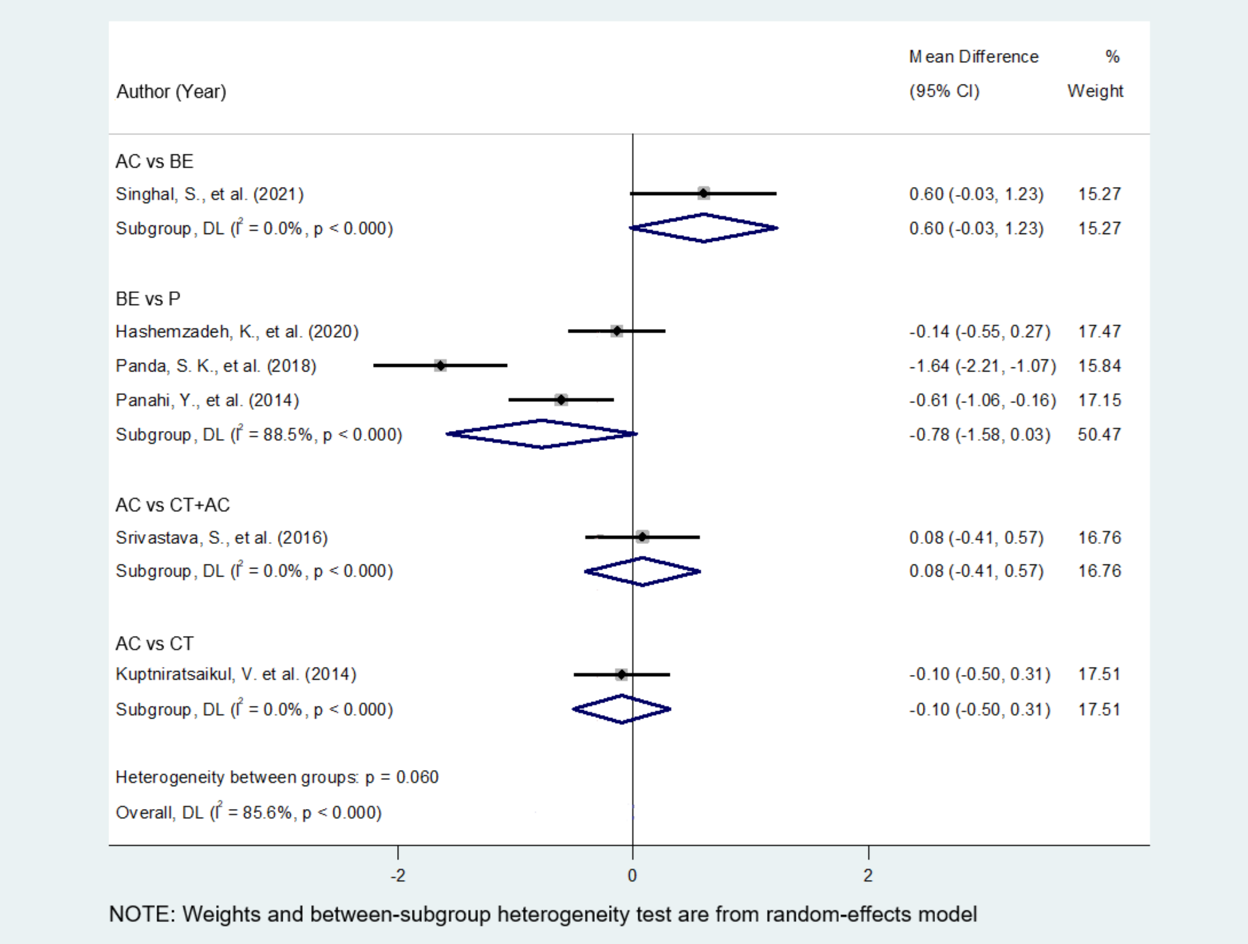

Abbreviation: CI, confidence interval; AC, active drug comparator; BE, bioavailability-enhanced curcuminoid preparations; CT, conventional curcuminoid preparations; CT + AC, conventional curcuminoid preparations + active drug comparator; P, placebo

eFigure F3 Mean difference (and 95% CI) for WOMAC function

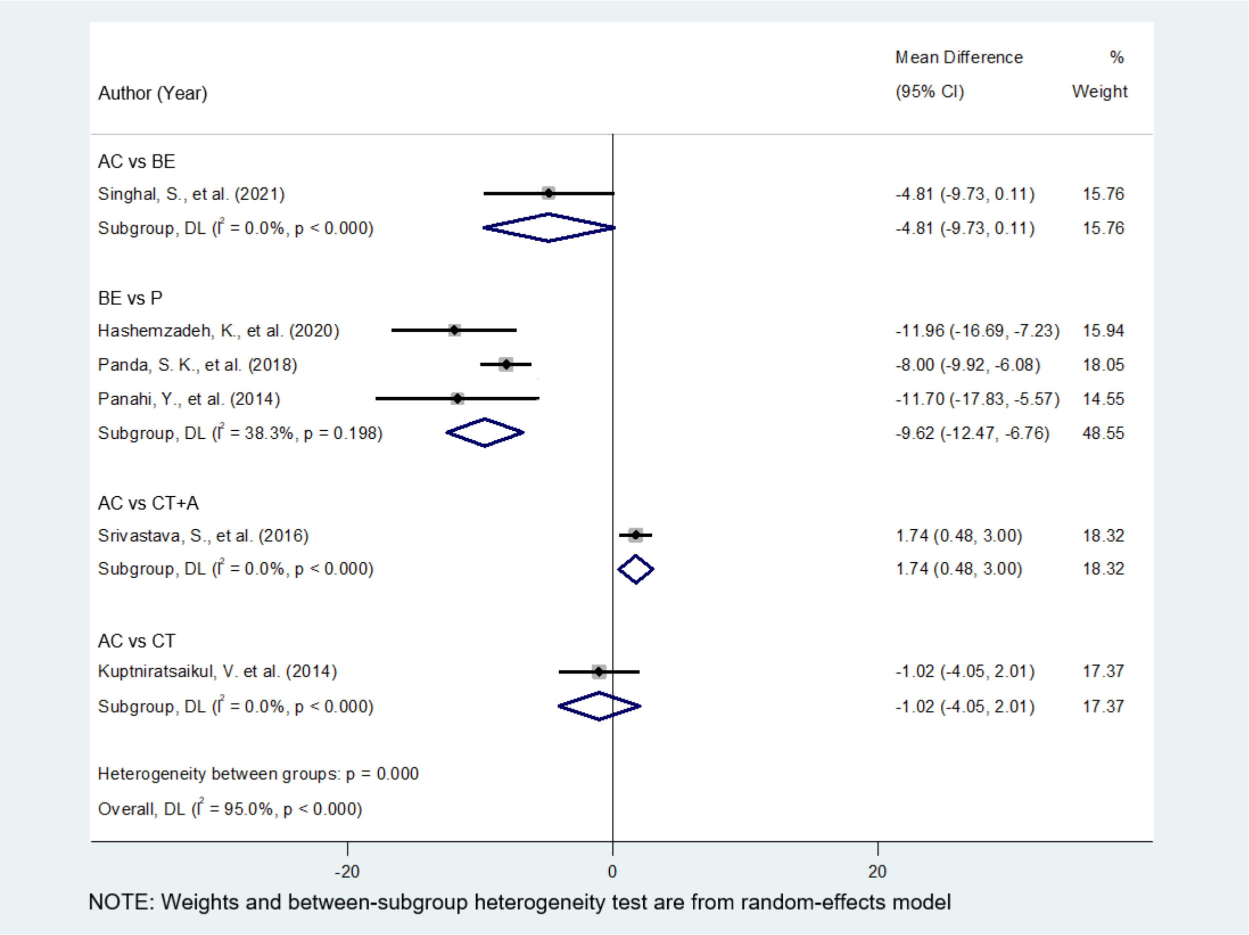

Abbreviation: CI, confidence interval; AC, active drug comparator; BE, bioavailability-enhanced curcuminoid preparations; CT, conventional curcuminoid preparations; CT + AC, conventional curcuminoid preparations + active drug comparator; P, placebo

Eleven RCTs reported VAS outcomes [5-7, 9-12, 14-16, 18]. Nakagawa et al. 2014 and Wang et al. 2020 reported mean change data only, so they were not integrated for analysis [14, 18]. Among the remaining nine RCTs, two studies were excluded: Gupte et al. 2019 reported the data graphically, and Pinsornsak et al. 2012 reported the data in mean only without SD [15, 16]. Finally, seven studies (*N* = 603) were included for VAS pain outcome. Subgroups were formed according to their intervention group and control group: A vs. BE, BE vs. P, A vs. CT + A, P vs. PLS, and A vs. BE + A.

eFigure F4 Mean difference (and 95% CI) for VAS

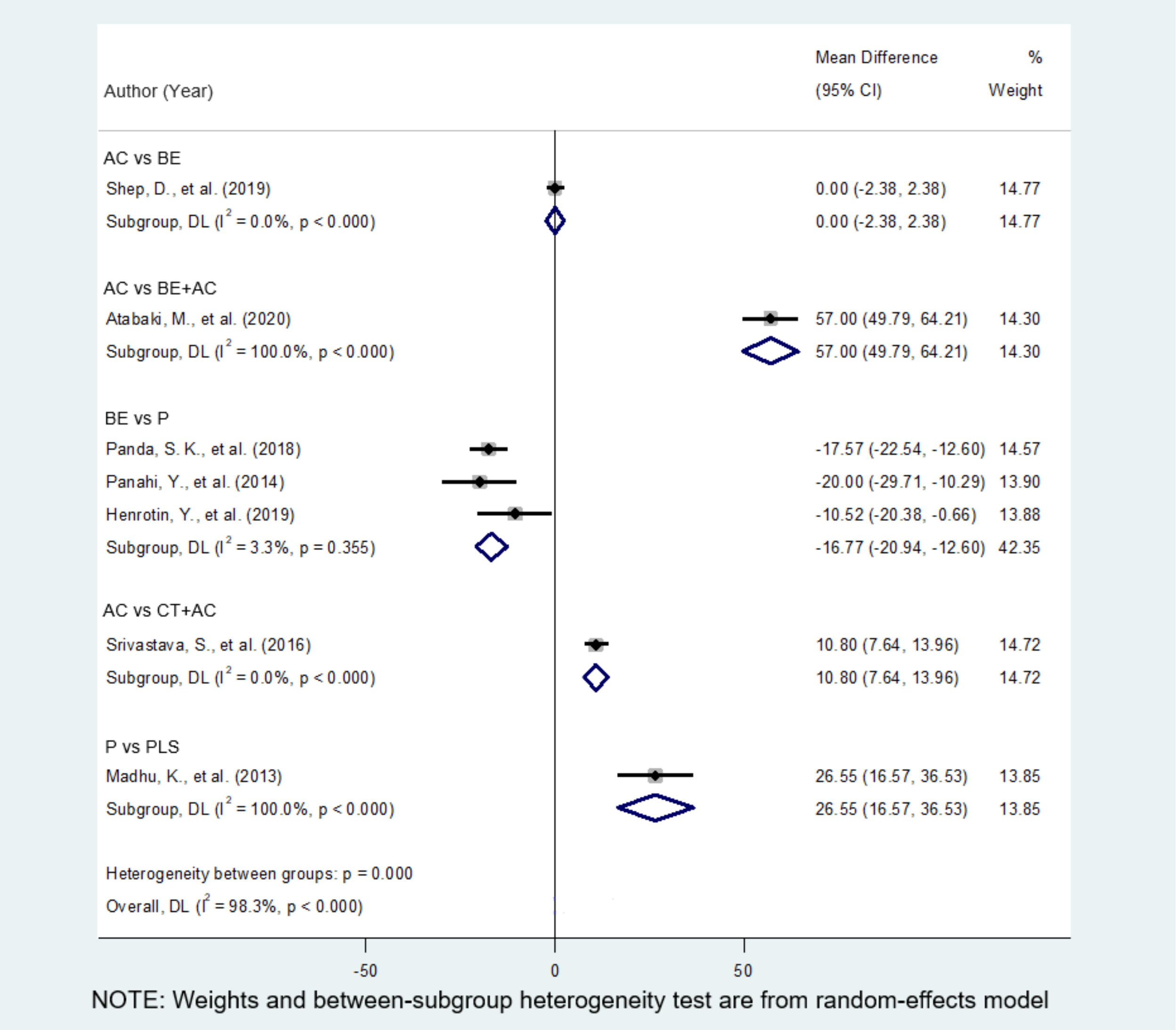

Abbreviation: CI, confidence interval; AC, active drug comparator; BE, bioavailability-enhanced curcuminoid preparations; BE + AC, bioavailability-enhanced curcuminoid preparations + active

drug comparator; CT + AC, conventional curcuminoid preparations + active drug comparator; P, placebo; PLS, polysaccharide preparations

Appendix G

Assessment of global inconsistency for each outcome network in main analysis

eTable G Assessment of global inconsistency in networks using the ‘design-by-treatment’ interaction model

**Network outcome**	**Chi-square**	***p*-value for test of global inconsistency**
WOMAC pain	36.59	0.0000
WOMAC stiffness	3.17	0.0748
WOMAC function	30.11	0.0000
VAS pain	27.16	0.0000

Appendix H

Interval plots

eFigure H1 Interval plot of WOMAC pain

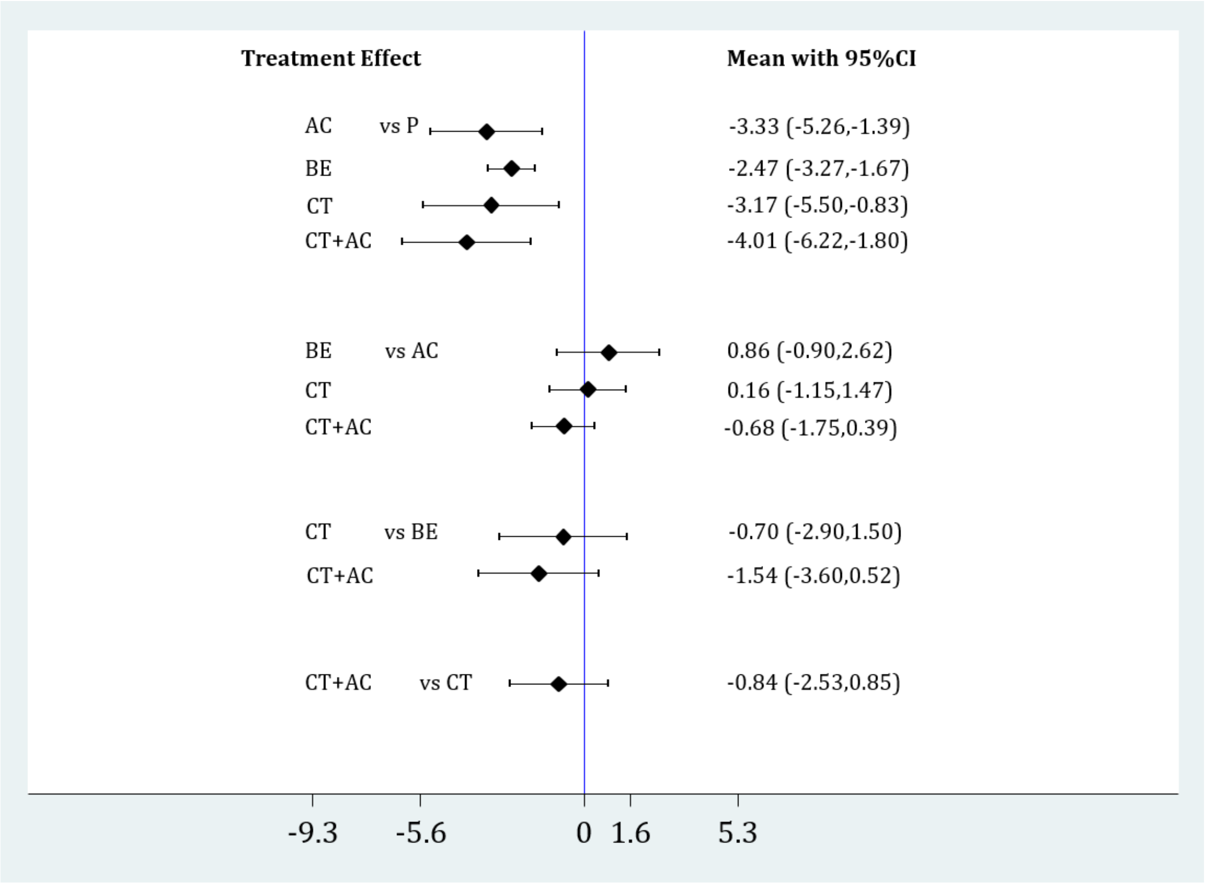

(chi-square for global consistency = 36.59, p = 0.0000)

Abbreviation: CI, confidence interval; AC, active drug comparator; BE, bioavailability-enhanced curcuminoid preparations; CT, conventional curcuminoid preparations; CT + AC, conventional curcuminoid preparations + active drug comparator; P, placebo

eFigure H2 Interval plot of WOMAC stiffness

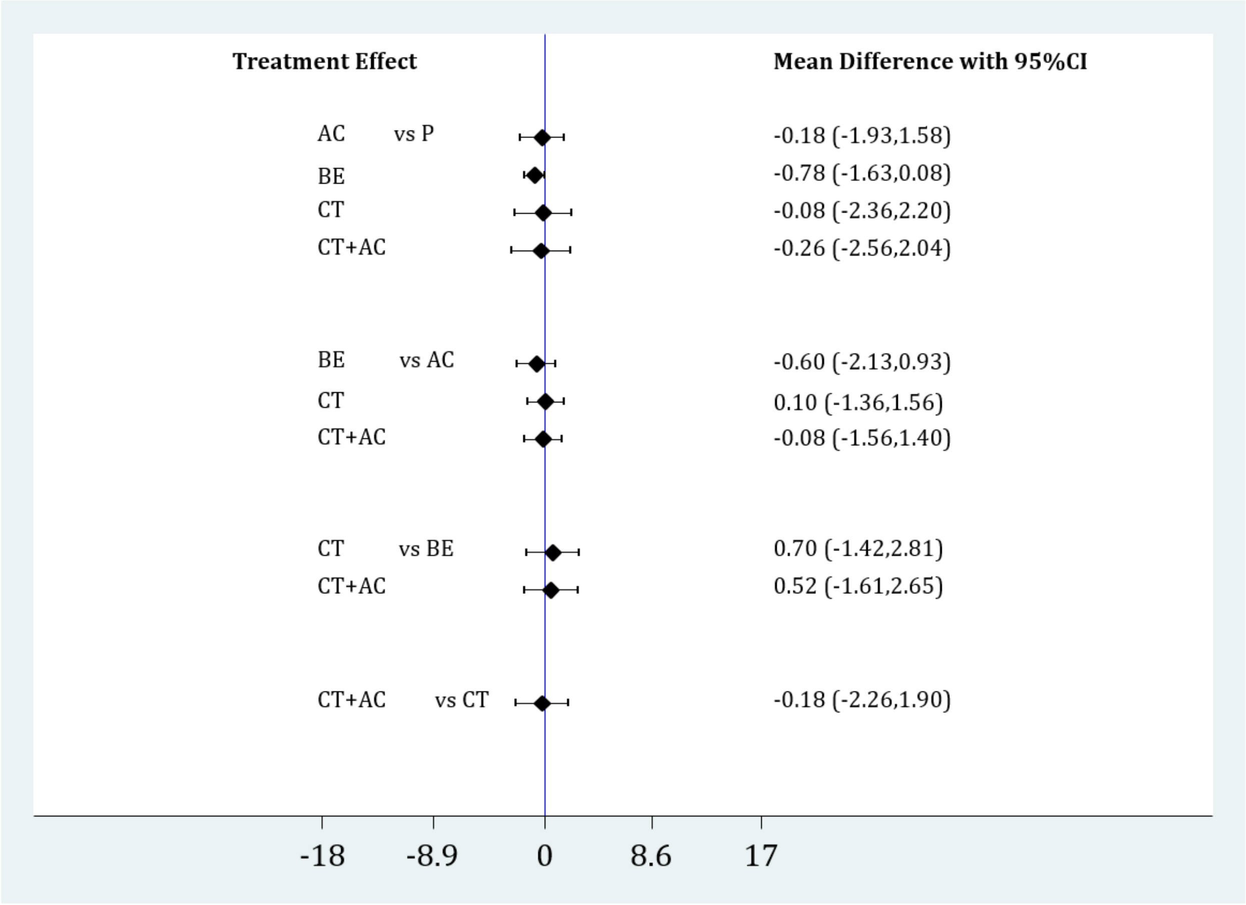

(chi-square for global consistency = 3.17, *p* = 0.0748)

Abbreviation: CI, confidence interval; AC, active drug comparator; BE, bioavailability-enhanced curcuminoid preparations; CT, conventional curcuminoid preparations; CT + AC, conventional curcuminoid preparations + active drug comparator; P, placebo

eFigure H3 Interval plot of WOMAC function

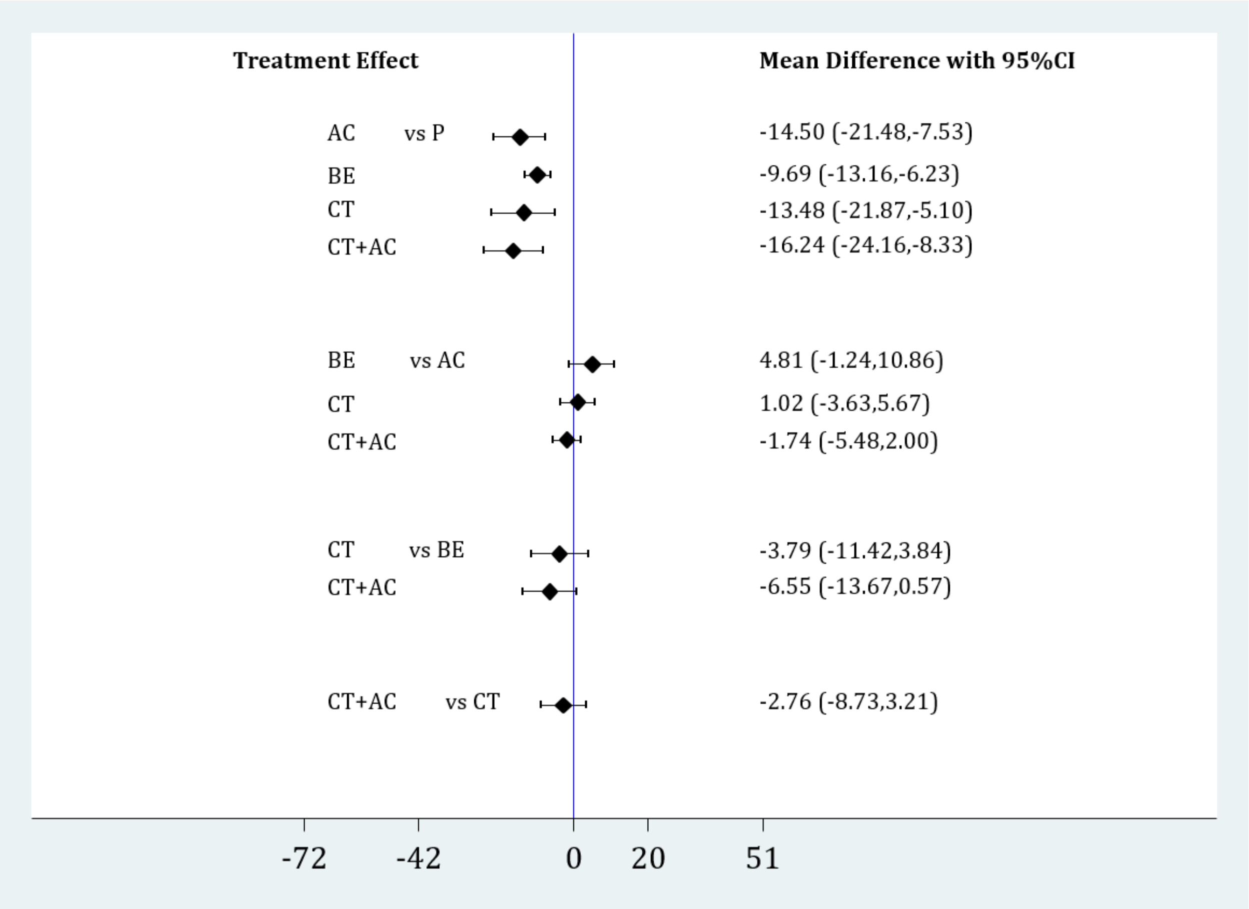

(chi-square for global consistency = 30.11, p = 0.0000)

Abbreviation: CI, confidence interval; AC, active drug comparator; BE, bioavailability-enhanced curcuminoid preparations; CT, conventional curcuminoid preparations; CT + AC, conventional curcuminoid preparations + active drug comparator; P, placebo

eFigure H4 Interval plot of VAS

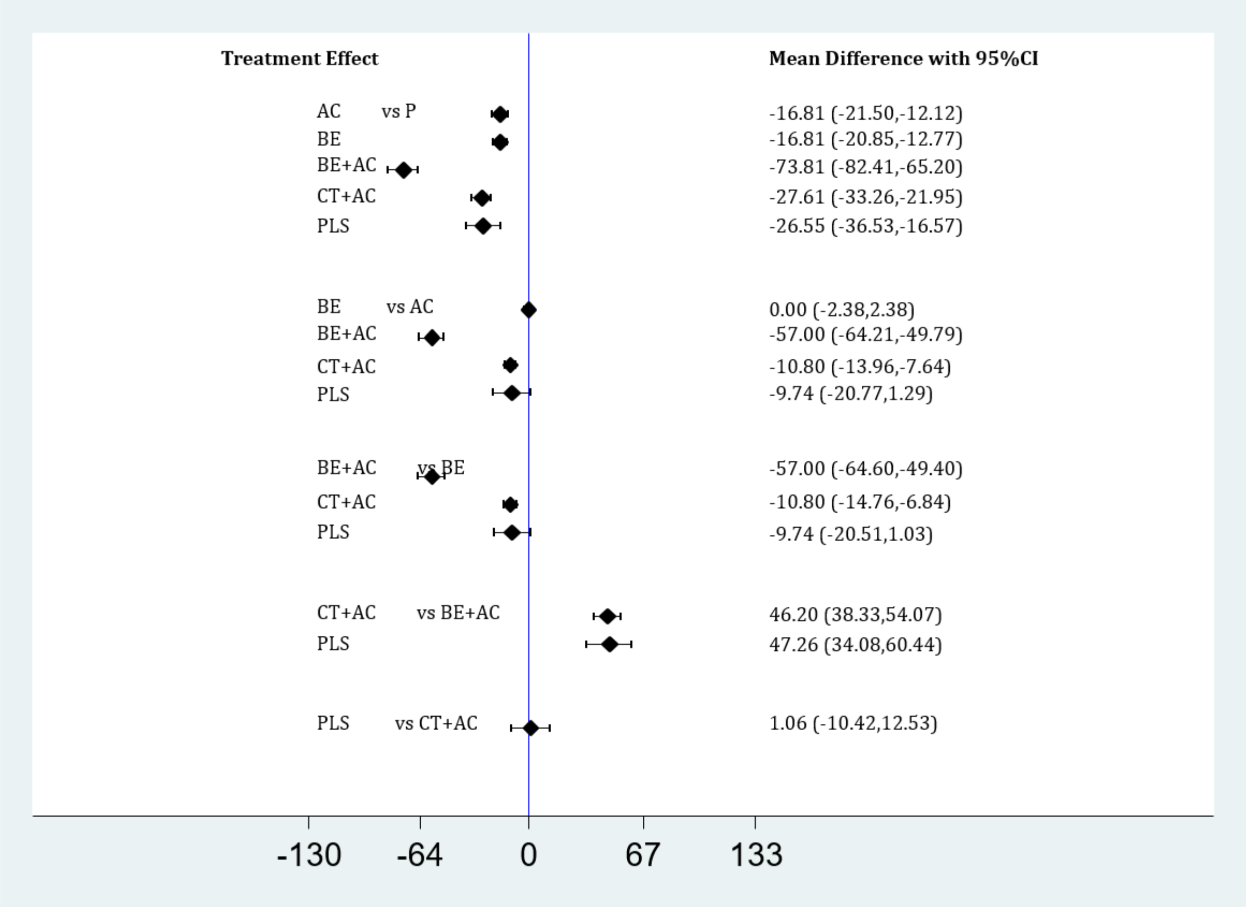

(chi-square for global consistency = 27.16, p = 0.0000)

Abbreviation: CI, confidence interval; AC, active drug comparator; BE, bioavailability-enhanced curcuminoid preparations; BE + AC, bioavailability-enhanced curcuminoid preparations + active drug comparator; CT + AC, conventional curcuminoid preparations + active drug comparator; P, placebo; PLS, polysaccharide preparations

Appendix I

SUCRA ranking for WOMAC Pain, Stiffness, Function, and VAS

eFigure I1 SUCRA ranking among interventions on WOMAC pain

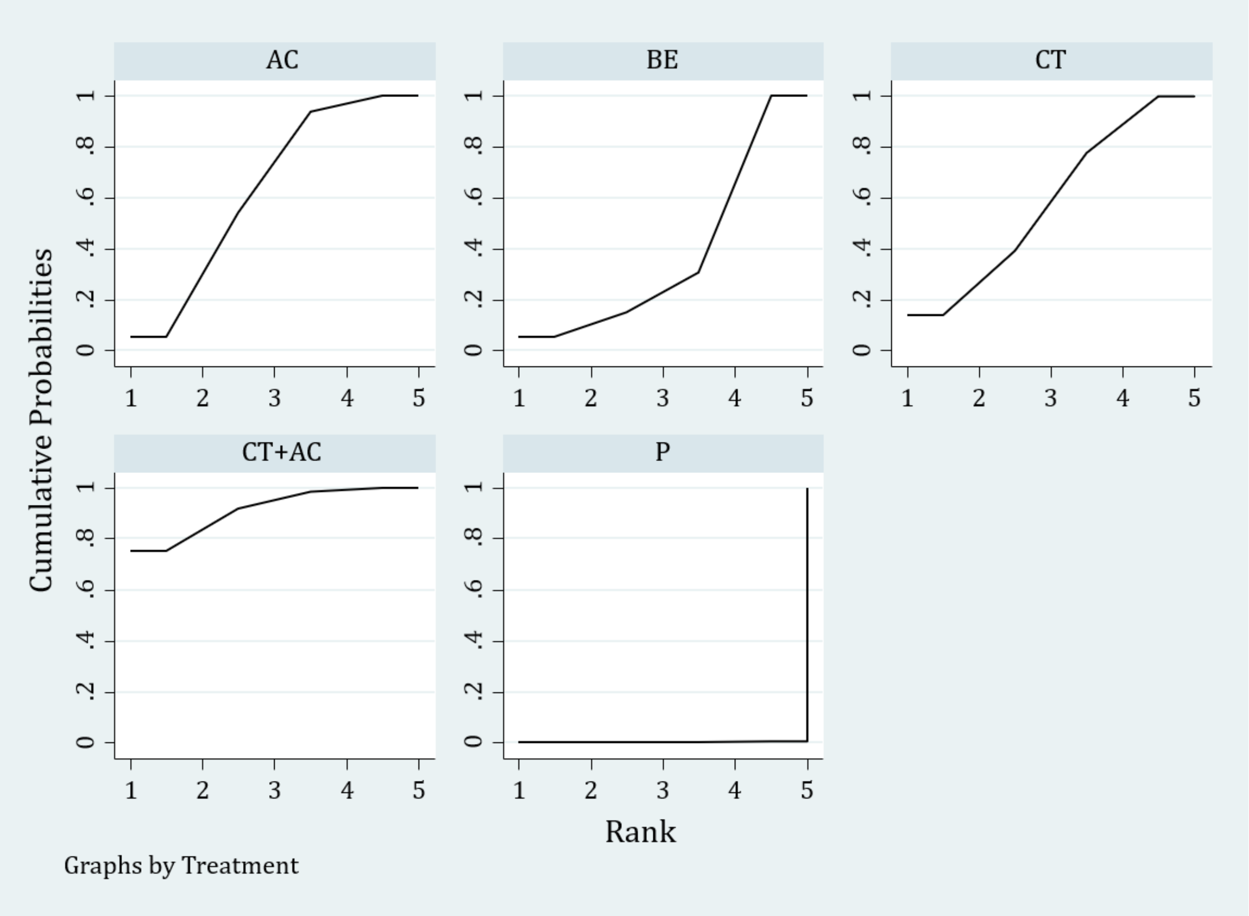

Abbreviation: AC, active drug comparator; BE, bioavailability-enhanced curcuminoid preparations; CT, conventional curcuminoid preparations; CT + AC, conventional curcuminoid preparations + active drug comparator; P, placebo; SUCRA, surface under the cumulative ranking

eFigure I2 SUCRA ranking among interventions on WOMAC stiffness

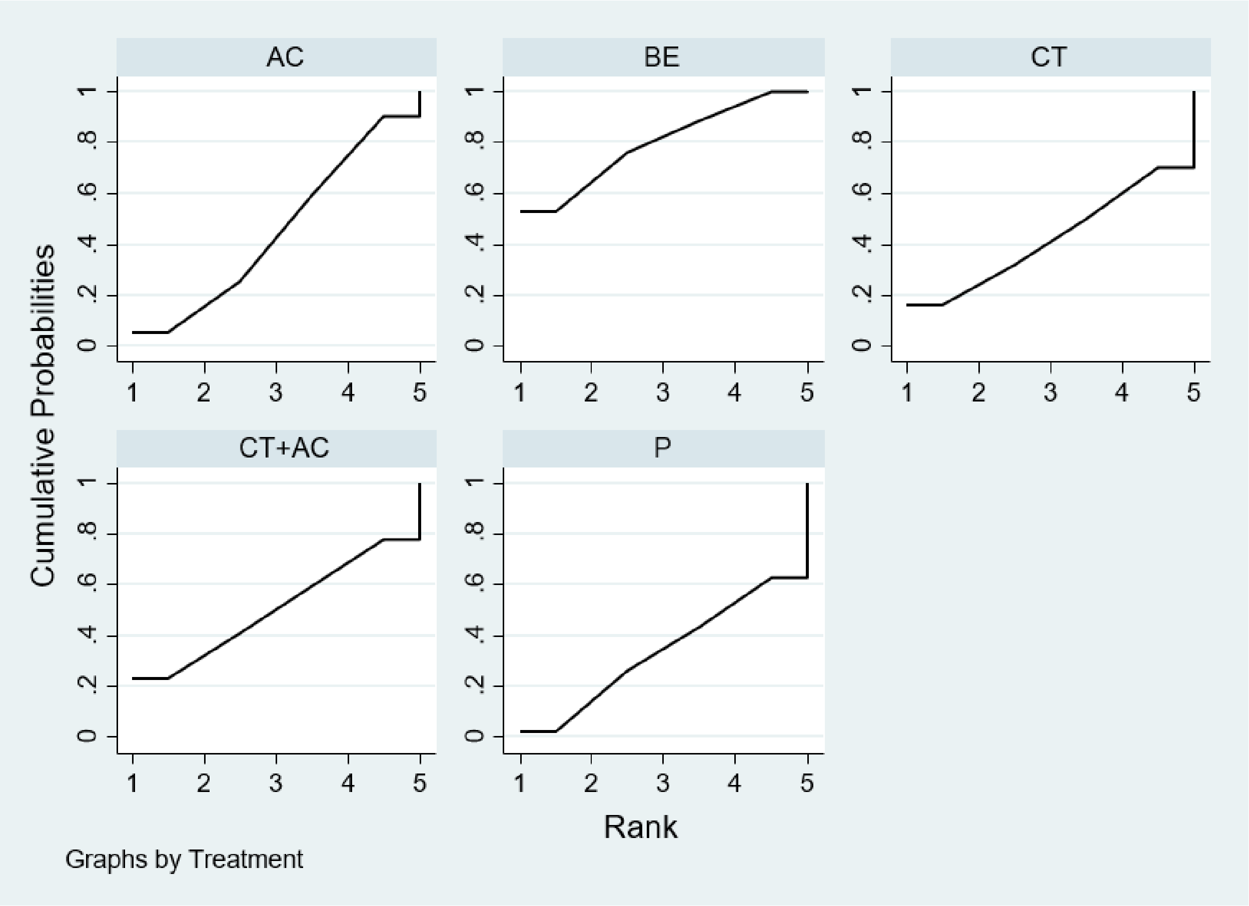

Abbreviation: AC, active drug comparator; BE, bioavailability-enhanced curcuminoid preparations; CT, conventional curcuminoid preparations; CT + AC, conventional curcuminoid preparations + active drug comparator; P, placebo; SUCRA, surface under the cumulative ranking

eFigure I3 SUCRA ranking among interventions on WOMAC function

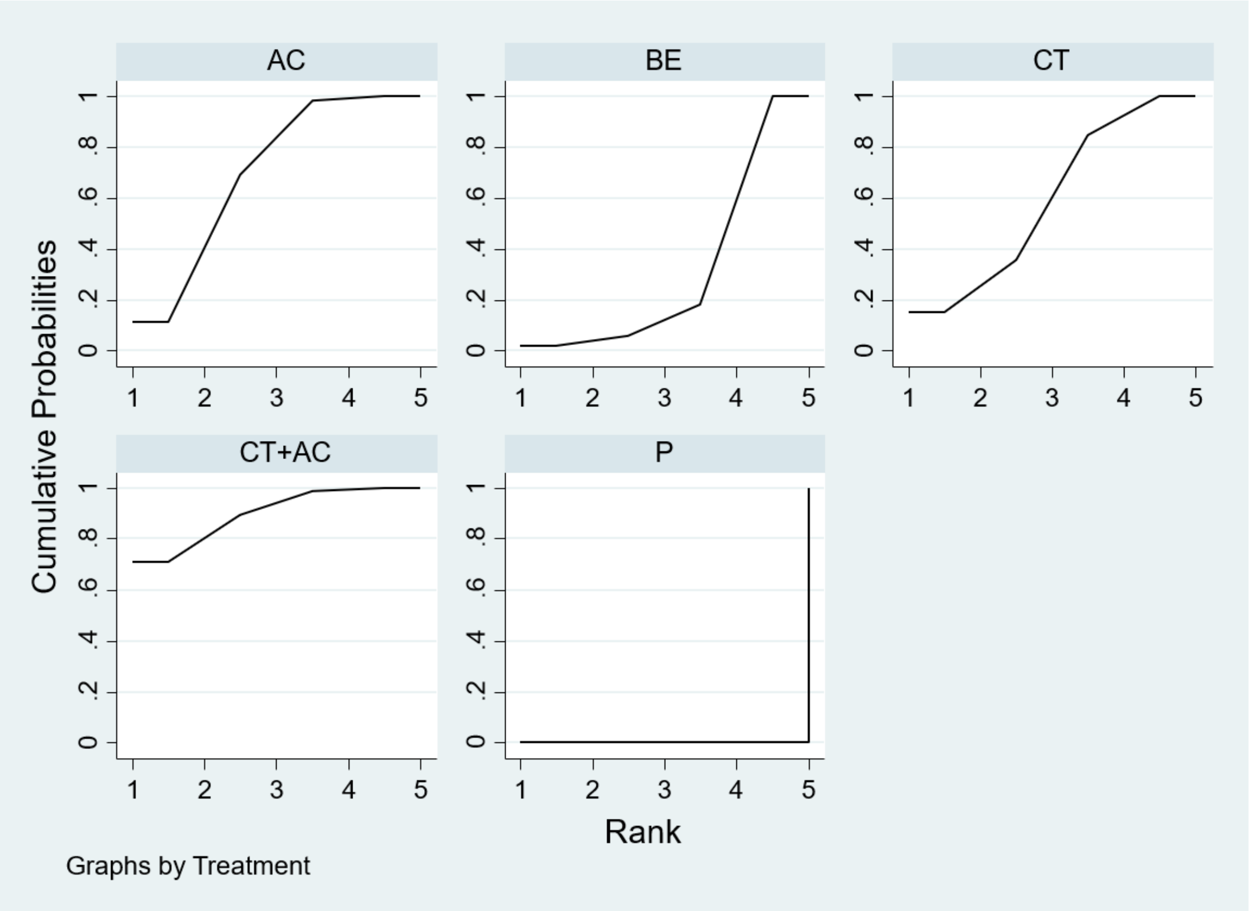

Abbreviation: AC, active drug comparator; BE, bioavailability-enhanced curcuminoid preparations; CT, conventional curcuminoid preparations; CT + AC, conventional curcuminoid preparations + active drug comparator; P, placebo; SUCRA, surface under the cumulative ranking

eFigure I4 SUCRA ranking among interventions on VAS

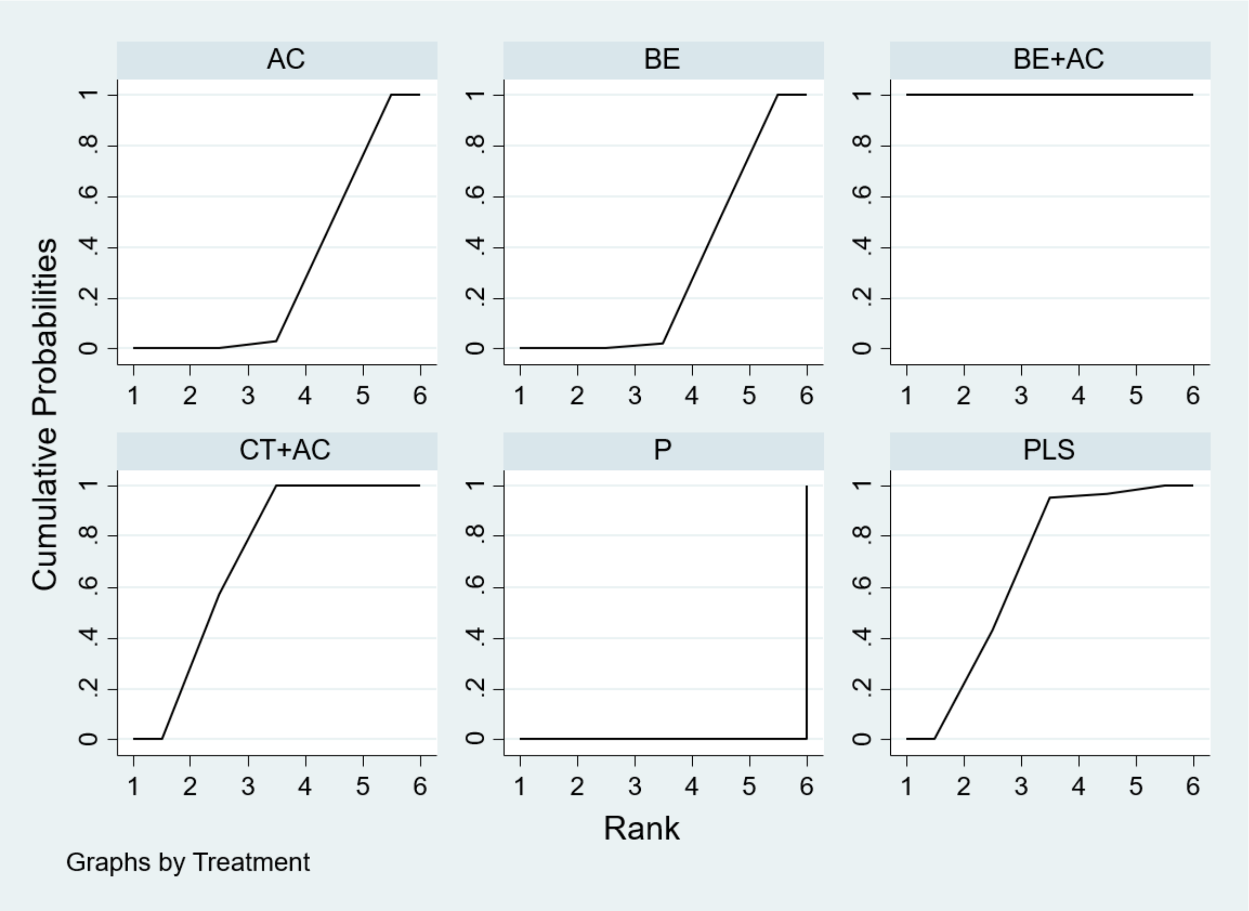

Abbreviation: AC, active drug comparator; BE, bioavailability-enhanced curcuminoid preparations; BE + AC, bioavailability-enhanced curcuminoid preparations + active drug comparator; CT + AC, conventional curcuminoid preparations + active drug comparator; P, placebo; PLS, polysaccharide preparations; SUCRA, surface under the cumulative ranking

Appendix J

Adjusted funnel plots

eFigure J1 Adjusted funnel plot for WOMAC pain

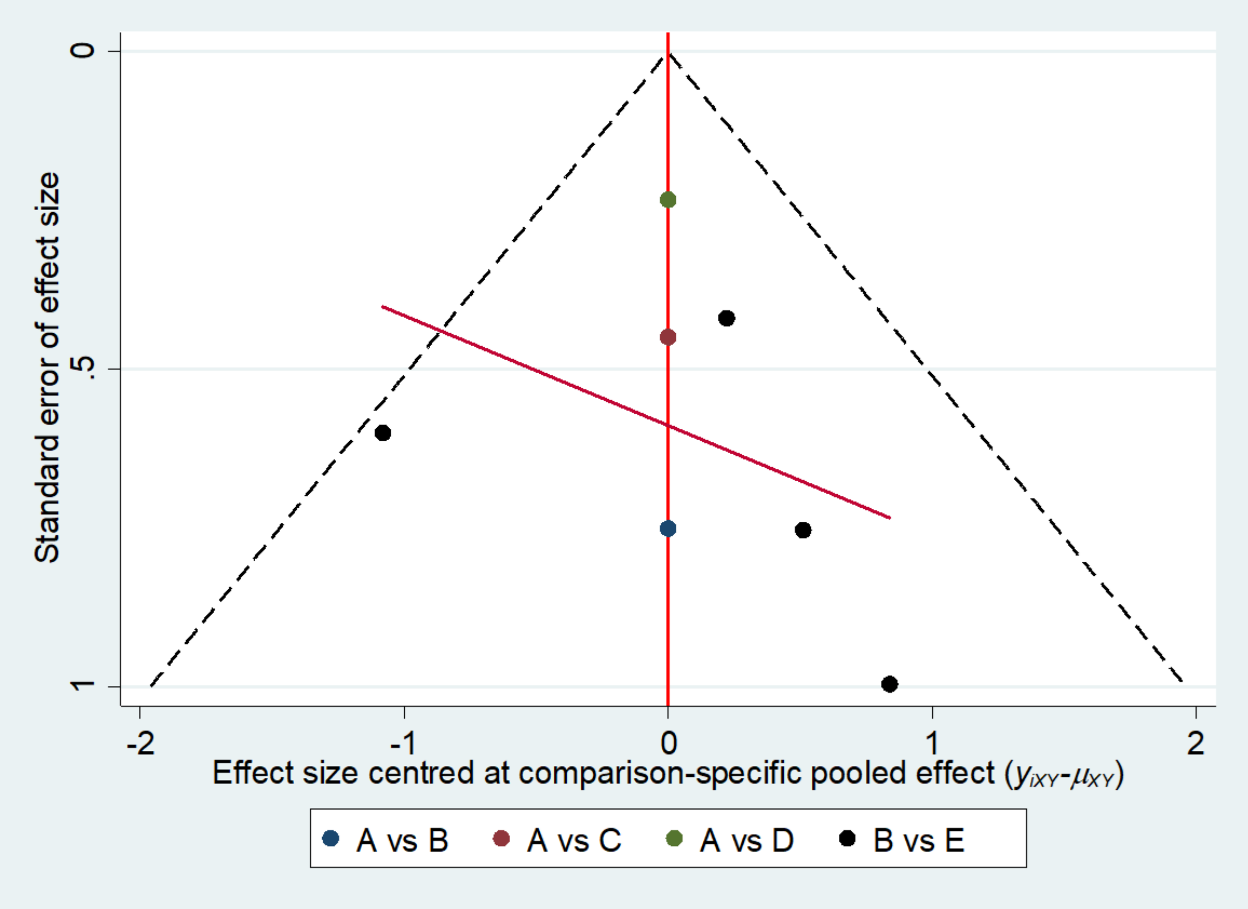

Abbreviation: A = AC, active drug comparator; B = BE, bioavailability-enhanced curcuminoid preparations; C = CT, conventional curcuminoid preparations; D = CT + AC, conventional curcuminoid preparations + active drug comparator; E = P, placebo

eFigure J2 Adjusted funnel plot for WOMAC stiffness

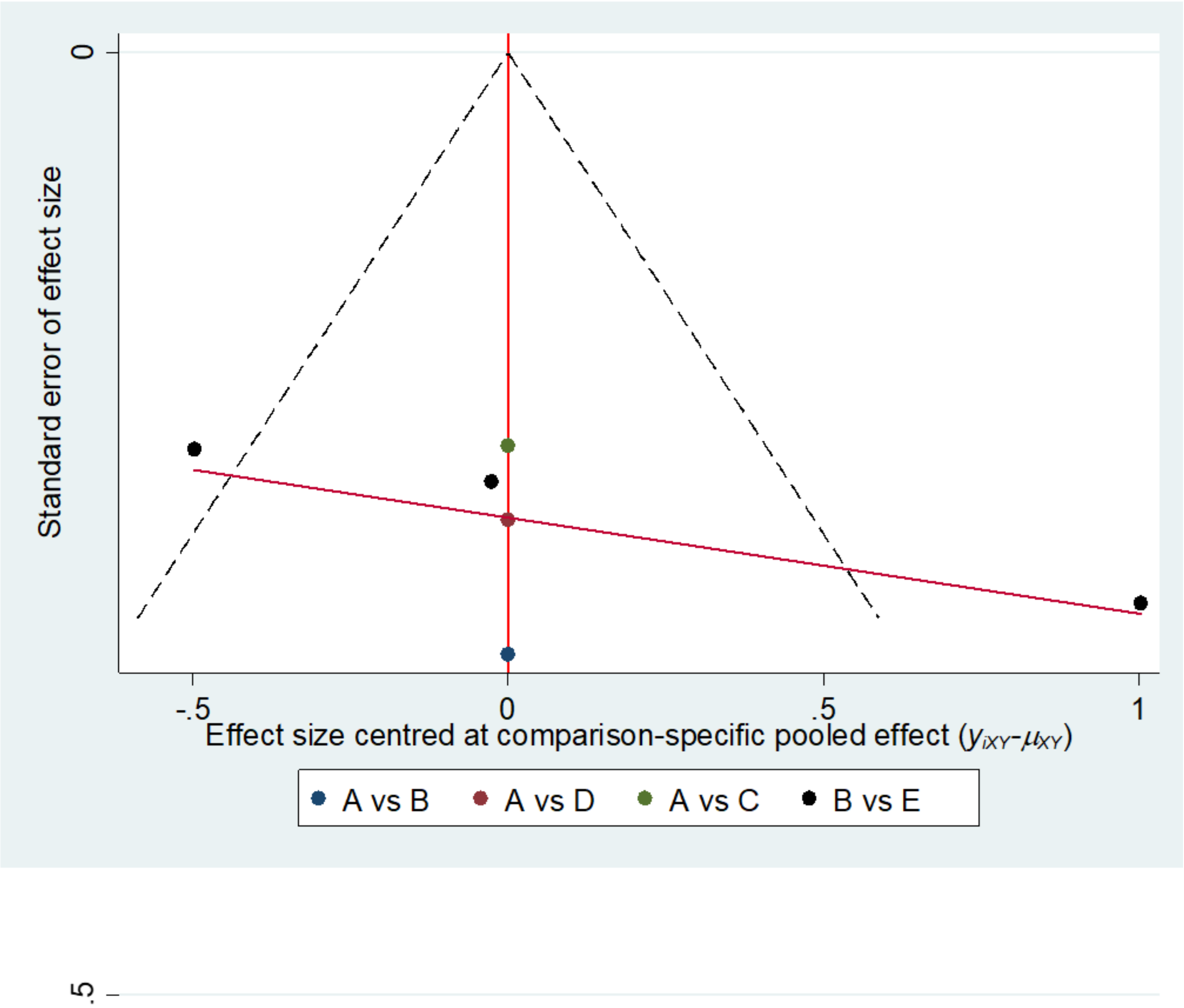

Abbreviation: A = AC, active drug comparator; B = BE, bioavailability-enhanced curcuminoid preparations; C = CT, conventional curcuminoid preparations; D = CT + AC, conventional curcuminoid preparations + active drug comparator; E = P, placebo

eFigure J3 Adjusted funnel plot for WOMAC function

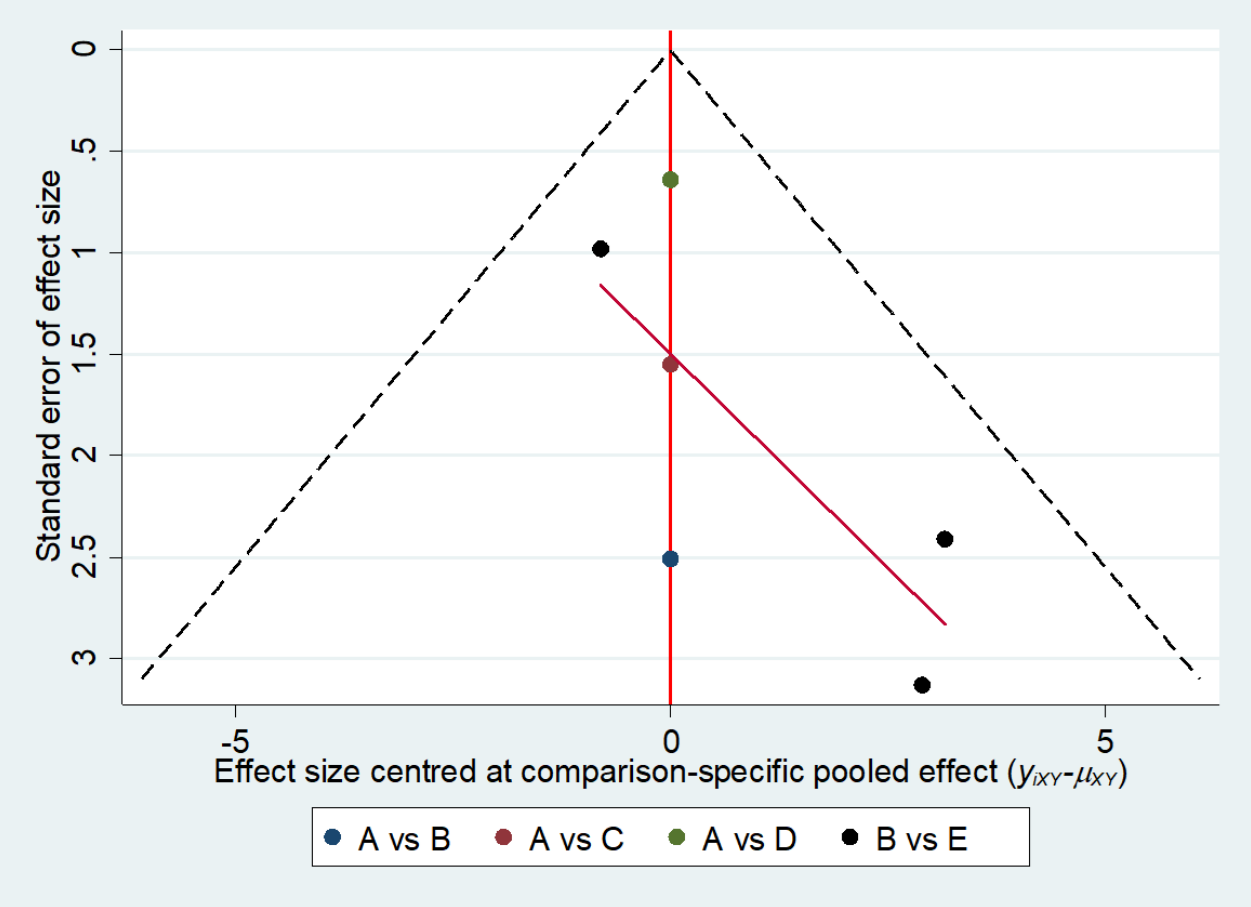

Abbreviation: A = AC, active drug comparator; B = BE, bioavailability-enhanced curcuminoid preparations; C = CT, conventional curcuminoid preparations; D = CT + AC, conventional curcuminoid preparations + active drug comparator; E = P, placebo

eFigure J4 Adjusted funnel plot for VAS

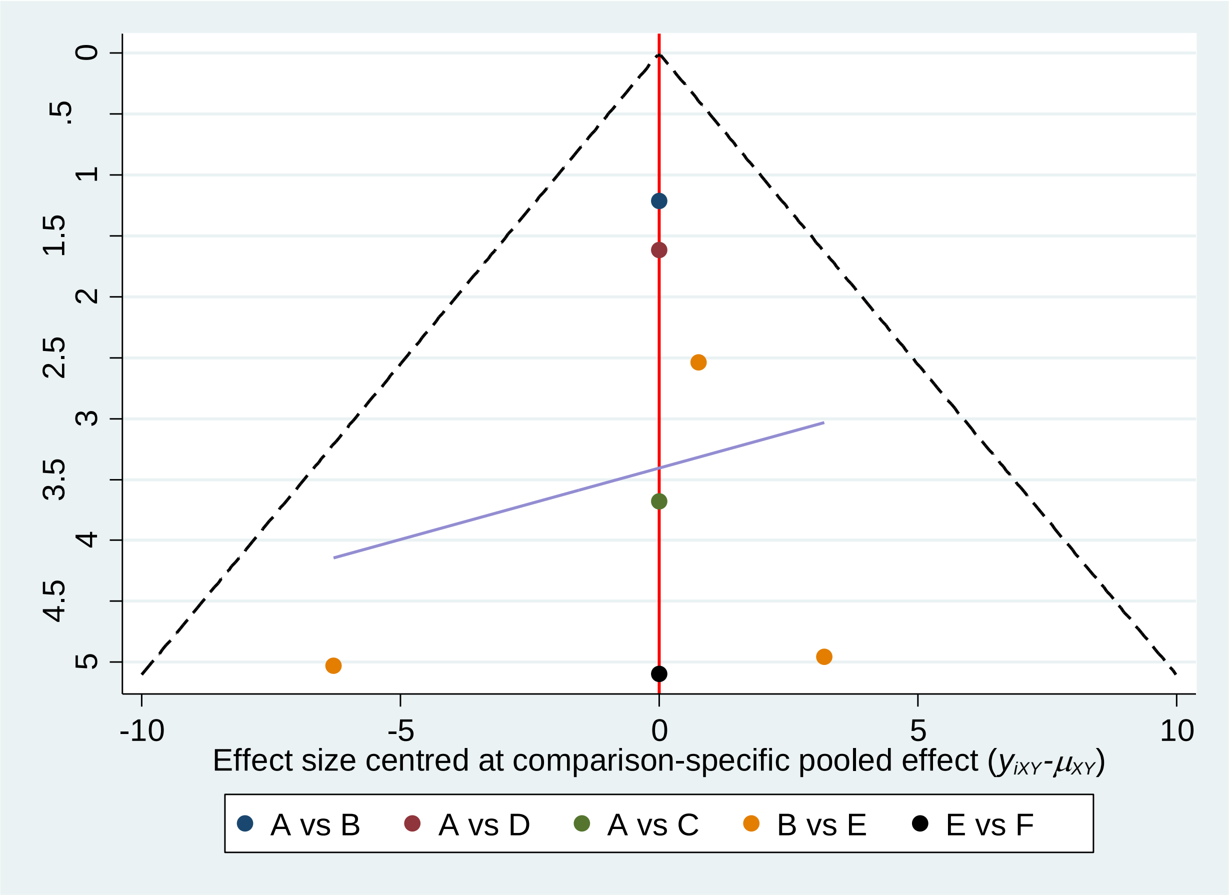

Abbreviation: A = AC, active drug comparator; B = BE, bioavailability-enhanced curcuminoid preparations; C = BE + AC, bioavailability-enhanced curcuminoid preparations + active drug comparator; D = CT + AC, conventional curcuminoid preparations + active drug comparator; E = P, placebo; F = PLS, polysaccharide preparations

Appendix K

Transitivity assessment tables

eTable K1 Descriptive table for the transitivity assessment of potential effect modifiers on WOMAC pain outcome

_Contrast	Studies	Mean age in years (range), SD	Female in percentage (range)	Mean BMI in kg/m^2 (range), SD	Mean duration of baseline knee OA/pain in months (range), SD	Mean baseline WOMAC pain intensity (range), SD	Follow-up period in days (range)
BE vs. P	4	55.57 (53.12 to 57.57), 7.96	85.23 (73.7 to 96.6)	27.65 (24.92 to 29.64), 3.21	23.25 (21.69 to 24.8), 9.23	8.13 (5.85 to 10.5), 2.91	57 (42 to 84)
CT+AC vs. AC	1	50.25 (50.23 to 50.27), 8.36	64.45 (61 to 67.9)	27.86 (27.4 to 28.32), 5.41	NA	15.20 (15.1 to 15.29), 2.55	120
CT vs. AC	1	60.6 (60.3 to 60.9), 6.85	89.35 (86.9 to 91.81)	26.55 (26.5 to 26.6), 3.85	51.65 (51.3 to 52), 52.55	10.7 (10.6 to 10.8), 3.5	28
BE vs. AC	1	51.95 (50.8 to 53.1), 10.4	74.35 (72.6 to 76.1)	NA	NA	NA	42

Abbreviation: AC, active drug comparator; BE, bioavailability-enhanced curcuminoid preparations; CT, conventional curcuminoid preparations; CT + AC, conventional curcuminoid preparations + active drug comparator; P, placebo

eTable K2 Descriptive table for the transitivity assessment of potential effect modifiers on WOMAC stiffness outcome

_Contrast	Studies	Mean age in years (range), SD	Female in percentage (range)	Mean BMI in kg/m^2 (range), SD	Mean duration of baseline knee OA/pain in months (range), SD	Mean baseline WOMAC stiffness intensity (range), SD	Follow-up period in days (range)
BE vs. P	3	55.64 (53.12 to 57.57),7.71	80.98 (73.7 to 88.6)	27.19 (24.92 to 29.64), 3,08	23.25 (21.69 to 24.8), 9.23	2.12 (0.28 to 4.72), 1.20	48 (42 to 60)
CT+AC vs. AC	1	50.25 (50.23 to 50.27), 8.36	64.45 (61 to 67.9)	27.86 (27.4 to 28.82), 5.41	NA	5.43 (5.31 to 5.55), 1.46	120
CT vs. AC	1	60.6 (60.3 to 60.9), 6.85	89.35 (86.9 to 91.81)	26.55 (26.5 to 26.6), 3.85	51.65 (51.3 to 52), 52.55	4.12 (4.08 to 4.16), 2.08	28
BE vs. AC	1	51.95 (50.8 to 53.1), 10.4	74.35 (72.6 to 76.1)	NA	NA	NA	42

Abbreviation: AC, active drug comparator; BE, bioavailability-enhanced curcuminoid preparations; CT, conventional curcuminoid preparations; CT + AC, conventional curcuminoid preparations + active drug comparator; P, placebo

eTable K3 Descriptive table for the transitivity assessment of potential effect modifiers on WOMAC function outcome

_Contrast	Studies	Mean age in years (range), SD	Female in percentage (range)	Mean BMI in kg/m^2 (range), SD	Mean duration of baseline knee OA/pain in months (range), SD	Mean baseline WOMAC function intensity (range), SD	Follow-up period in days (range)
BE vs. P	3	55.64 (53.12 to 57.57),7.71	80.98 (73.7 to 88.6)	27.19 (24.92 to 29.64), 3,08	23.25 (21.69 to 24.8), 9.23	27.28 (24.32 to 32.4), 6.81	48 (42 to 60)
CT+AC vs. AC	1	50.25 (50.23 to 50.27), 8.36	64.45 (61 to 67.9)	27.86 (27.4 to 28.82), 5.41	NA	52.51 (50.99 to 54.03), 6.08	120
CT vs. AC	1	60.6 (60.3 to 60.9), 6.85	89.35 (86.9 to 91.81)	26.55 (26.5 to 26.6), 3.85	51.65 (51.3 to 52), 52.55	35.36 (34.68 to 36.04), 12.92	28
BE vs. AC	1	51.95 (50.8 to 53.1), 10.4	74.35 (72.6 to 76.1)	NA	NA	NA	42

Abbreviation: AC, active drug comparator; BE, bioavailability-enhanced curcuminoid preparations; CT, conventional curcuminoid preparations; CT + AC, conventional curcuminoid preparations + active drug comparator; P, placebo

eTable K4 Descriptive table for the transitivity assessment of potential effect modifiers on VAS outcome

_Contrast	Studies	Mean age in years (range), SD	Female in percentage (range)	Mean BMI in kg/m^2 (range), SD	Mean duration of baseline knee OA/pain in months (range), SD	Mean baseline VAS pain intensity (range), SD	Follow-up period in days (range)
BE vs. P	3	57.95 (53.12 to 63.3), 8.52	78.13 (73.7 to 82.19)	28.01 (24.92 to 29.89), 3.76	87.68 (84.16 to 91.2), 92.57	58.92 (52.37 to 66.32), 11.58	64 (42 to 90)
BE vs. AC	1	52.62 (52.14 to 53.09), 3.97	33.07 (30.43 to 35.71)	NA	7.43 (7.4 to 7.45), 3.34	78.25 (78.1 to 78.4), 6.80	28
BE+AC vs. AC	1	48.70 (48.26 to 49.13), 5.46	100	21.95 (21.9 to 22), 1.45	54.96 (53.52 to 56.4), 30.21	81.95 (79.3 to 84.6), 17.23	90
CT+AC vs. AC	1	50.25 (50.23 to 50.27), 8.36	64.45 (61 to 67.9)	27.86 (27.4 to 28.82), 5.41	NA	78 (76.6 to 79.4), 12.08	120
PLS vs. P	1	56.70 (56.63 to 56.77), 10.28	56.67	27.49 (27.01 to 27.97), 4.41	$\geq$ 6	64 (61.5 to 66.5), 17.39	42

Abbreviation: AC, active drug comparator; BE, bioavailability-enhanced curcuminoid preparations; BE + AC, bioavailability-enhanced curcuminoid preparations + active drug comparator; CT + AC, conventional curcuminoid preparations + active drug comparator; P, placebo; PLS, polysaccharide preparations

Appendix L

Adverse events

Out of the seventeen included studies, five studies were excluded for reporting only the total number of participants experiencing adverse events (AE), without specifying the number of participants for each AE [8, 9, 11, 13, 17]. For the remaining twelve studies, a meta-analysis was not conducted.

eTable L Frequency of adverse events across different treatment groups

**Type of ADR**	**P (N= 236)**	**AC (N=232)**	**BE (N=260)**	**BE + AC (N=15)**	**CT + AC (N=114)**	**PLS (N=66)**
All adverse events	41 (17.36%)	17 (7.32%)	24 (9.22%)	0 (0%)	3 (2.63%)	20 (30.30%)
GI symptoms	12 (5.08%)	8 (3.45%)	17 (6.54%)	0 (0%)	2 (1.75%)	4 (6.06%)
Respiratory	1 (0.42%)	0 (0%)	0 (0%)	0 (0%)	0 (0%)	1 (1.52%)
Neurological and sensory symptoms	2 (0.85%)	4 (1.72%)	5 (1.92%)	0 (0%)	0 (0%)	2 (3.03%)
Dermatological reactions	1 (0.42%)	1 (0.43%)	1 (0.38%)	0 (0%)	1 (0.88%)	0 (0%)
Cardiovascular and edema	1 (0.42%)	0 (0%)	1 (0.38%)	0 (0%)	0 (0%)	0 (0%)
Metabolic effects and weight changes	2 (0.85%)	0 (0%)	0 (0%)	0 (0%)	0 (0%)	0 (0%)
General body pain	5 (2.12%)	0 (0%)	0 (0%)	0 (0%)	0 (0%)	1 (1.52%)
Complications and medical problems	0 (0%)	4 (1.72%)	0 (0%)	0 (0%)	0 (0%)	0 (0%)
Miscellaneous	17 (7.20%)	0 (0%)	0 (0%)	0 (0%)	0 (0%)	12 (18.18%)

Abbreviation: AC, active drug comparator; ADR, adverse drug reaction; BE, bioavailability-enhanced curcuminoid preparations; CT, conventional curcuminoid preparations; GI, gastrointestinal; P, placebo; PLS, polysaccharide preparations

Appendix M
Quality of evidence

eTable M Grading

Comparison	Direct evidence		Indirect evidence		Network meta-analysis	
	Mean difference (95% confidence interval)	Quality of evidence	Mean difference (95% confidence interval)	Quality of evidence	Mean difference (95% confidence interval)	Quality of evidence
**WOMAC Pain**						
BE vs. AC	0.86 (-0.61 to 2.33)	⊕⊕⊕◯[a] MODERATE	-2.80 (-848.71 to 843.11)	⊕⊕◯◯[b] LOW	0.86 (-0.90 to 2.62)	⊕⊕◯◯[a,c] LOW
CT vs. AC	0.16 (-0.72 to 1.04)	⊕⊕⊕◯[a] MODERATE	6.64 (-4578.06 to 4591.35)	⊕⊕◯◯[b] LOW	0.16 (-1.15 to 1.47)	⊕⊕◯◯[a,c] LOW
CT + AC vs. AC	-0.68 (-1.14 to -0.22)	⊕⊕⊕⊕ HIGH	6.64 (-1745.58 to 1758.85)	⊕⊕◯◯[b] LOW	-0.68 (-1.75 to 0.39)	⊕⊕◯◯[a,c] LOW
BE vs. P	-2.47 (-3.25 to -1.68)	⊕⊕◯◯[a,d] LOW	1.08 (-833.42 to 835.59)	⊕⊕◯◯[b] LOW	-2.47 (-3.27 to -1.67)	⊕◯◯◯[a,c,d] VERY LOW
**WOMAC Stiffness**						
BE vs. AC	-0.60 (-1.23 to 0.03)	⊕⊕⊕◯[a] MODERATE	-0.85 (-719.56 to 717.86)	⊕⊕◯◯[b] LOW	-0.60 (-2.13 to 0.93)	⊕⊕⊕◯[a] MODERATE
CT vs. AC	0.10 (-0.31 to 0.50)	⊕⊕⊕⊕ HIGH	0.36 (-2351.42 to 2352.15)	⊕⊕◯◯[b] LOW	0.10 (-1.36 to 1.56)	⊕⊕⊕◯[a] MODERATE
CT + AC vs. AC	-0.08 (-0.57 to 0.41)	⊕⊕⊕⊕ HIGH	0.35 (-1955.96 to 1956.67)	⊕⊕◯◯[b] LOW	-0.08 (-1.56 to 1.40)	⊕⊕◯◯[a,c] LOW
BE vs. P	-0.78 (-1.58 to 0.03)	⊕◯◯◯ [a,d,f] VERY LOW	-0.51 (-639.98 to 638.96)	⊕⊕◯◯[b] LOW	-0.78 (-1.63 to 0.08)	⊕◯◯◯ [a,d,f] VERY LOW

eTable M Grading (cont.)

Comparison	Direct evidence		Indirect evidence		Network meta-analysis	
	**Mean difference (95% confidence interval)**	**Quality of evidence**	**Mean difference (95% confidence interval)**	**Quality of evidence**	**Mean difference (95% confidence interval)**	**Quality of evidence**
**WOMAC Function**						
BE vs. AC	4.81 (-0.11 to 9.73)	⊕⊕⊕◯[a] MODERATE	-10.58 (-2399.80 to 2378.64)	⊕⊕◯◯[b] LOW	4.81 (-1.24 to 10.86)	⊕⊕◯◯[a,c] LOW
CT vs. AC	1.02 (-2.01 to 4.05)	⊕⊕⊕◯[a] MODERATE	28.99 (-16620.51 to 16678.49)	⊕⊕◯◯[b] LOW	1.02 (-3.63 to 5.67)	⊕⊕◯◯[a,c] LOW
CT + AC vs. AC	-1.74 (-3.00 to -0.48)	⊕⊕⊕◯[a] MODERATE	28.97 (-5225.17 to 5283.12)	⊕⊕◯◯[b] LOW	-1.74 (-5.48 to 2.00)	⊕⊕◯◯[a,c] LOW
BE vs. P	-9.62 (-12.47 to -6.76)	⊕⊕◯◯[a,d] LOW	5.35 (-2317.66 to 2328.36)	⊕⊕◯◯[b] LOW	-9.69 (-13.16 to -6.23)	⊕◯◯◯[a,c,d] VERY LOW
**VAS**						
PLS vs. P	-26.55 (-36.53 to -16.57)	⊕⊕◯◯[a,e] LOW	Not estimable	Not estimable	-26.55 (-36.53 to -16.57)	⊕⊕◯◯[a,e] LOW
BE vs. AC	0.00 (-2.38 to 2.38)	⊕⊕⊕◯[a] MODERATE	-33.57 (-3996.87 to 3929.73)	⊕⊕◯◯[b] LOW	0.00 (-2.38 to 2.38)	⊕⊕◯◯[a,c] LOW
BE + AC vs. AC	-57.00 (-64.21 to -49.79)	⊕⊕⊕◯[a] MODERATE	33.61 (-12802.80 to 12870.03)	⊕⊕◯◯[b] LOW	-57.00 (-64.21 to -49.79)	⊕⊕◯◯[a,c] LOW
CT + AC vs. AC	-10.80 (-13.96 to -7.64)	⊕⊕⊕◯[a] MODERATE	33.22 (-11232.34 to 11298.79)	⊕⊕◯◯[b] LOW	-10.80 (-13.96 to -7.64)	⊕⊕◯◯[a,c] LOW

eTable M Grading (cont.)

Comparison	Direct evidence		Indirect evidence		Network meta-analysis	
	**Mean difference (95% confidence interval)**	**Quality of evidence**	**Mean difference (95% confidence interval)**	**Quality of evidence**	**Mean difference (95% confidence interval)**	**Quality of evidence**
BE vs. P	-16.77 (-20.94 to -12.60)	⊕⊕◯◯[a,d] LOW	8.40 (-3156.18 to 3172.99)	⊕⊕◯◯[b] LOW	-16.81 (-20.85 to -12.77)	⊕◯◯◯[a,c,d] VERY LOW

Explanations: [a] Downgraded one level due to serious imprecision, [b] Downgraded two levels due to very serious imprecision, [c] Downgraded one level due to incoherence, [d] Downgraded one level due to publication bias, [e] Downgraded one level due to risk of bias, [f] Downgraded two levels due to very serious inconsistency

Appendix N

Sensitivity analyses

eFigure N1 Sensitivity analysis of follow-up period for WOMAC pain

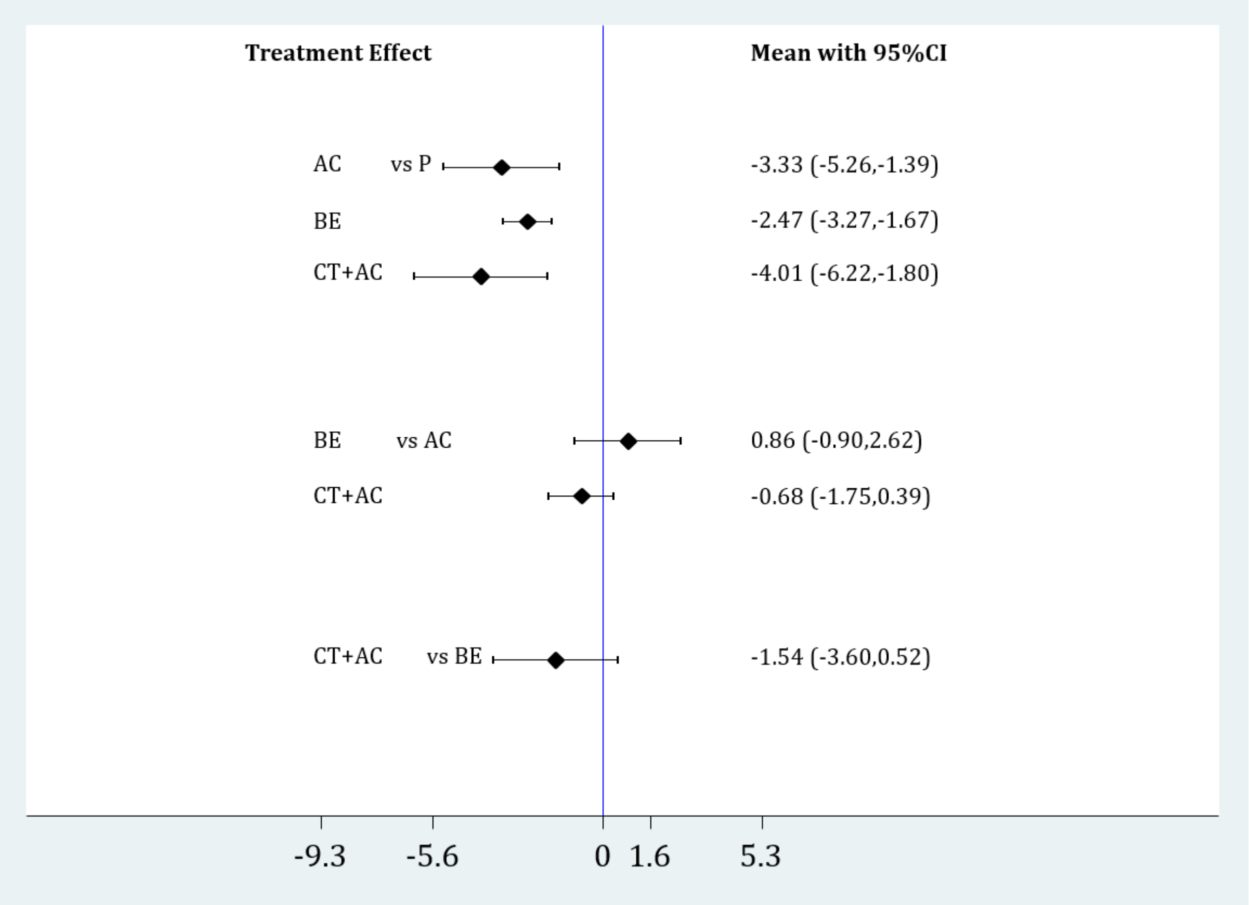

(chi-square for global inconsistency = 36.59, *p* = 0.0000)

Abbreviation: AC, active drug comparator; BE, bioavailability-enhanced curcuminoid preparations; CT, conventional curcuminoid preparations; CT + AC, conventional curcuminoid preparations + active drug comparator; P, placebo

eTable N1 Descriptive table for transitivity assessment of sensitivity analysis on follow-up period for WOMAC pain

_Contrast	Studies	Mean age in years (range), SD	Female in percentage (range)	Mean BMI in kg/m^2 (range), SD	Mean duration of baseline knee OA/pain in months (range), SD	Mean baseline WOMAC pain intensity (range), SD	Follow-up period in days (range)
BE vs. P	4	55.57 (53.12 to 57.57), 7.96	85.23 (73.7 to 96.6)	27.65 (24.92 to 29.64), 3.21	23.25 (21.69 to 24.8), 9.23	8.13 (5.85 to 10.5), 2.91	57 (42 to 84)
CT+AC vs. AC	1	50.25 (50.23 to 50.27), 8.36	64.45 (61 to 67.9)	27.86 (27.4 to 28.32), 5.41	NA	15.20 (15.1 to 15.29), 2.55	120
BE vs. AC	1	51.95 (50.8 to 53.1), 10.4	74.35 (72.6 to 76.1)	NA	NA	NA	42

Abbreviation: AC, active drug comparator; BE, bioavailability-enhanced curcuminoid preparations; CT + AC, conventional curcuminoid preparations + active drug comparator; P, placebo

eFigure N2 Sensitivity analysis of follow-up period for WOMAC stiffness

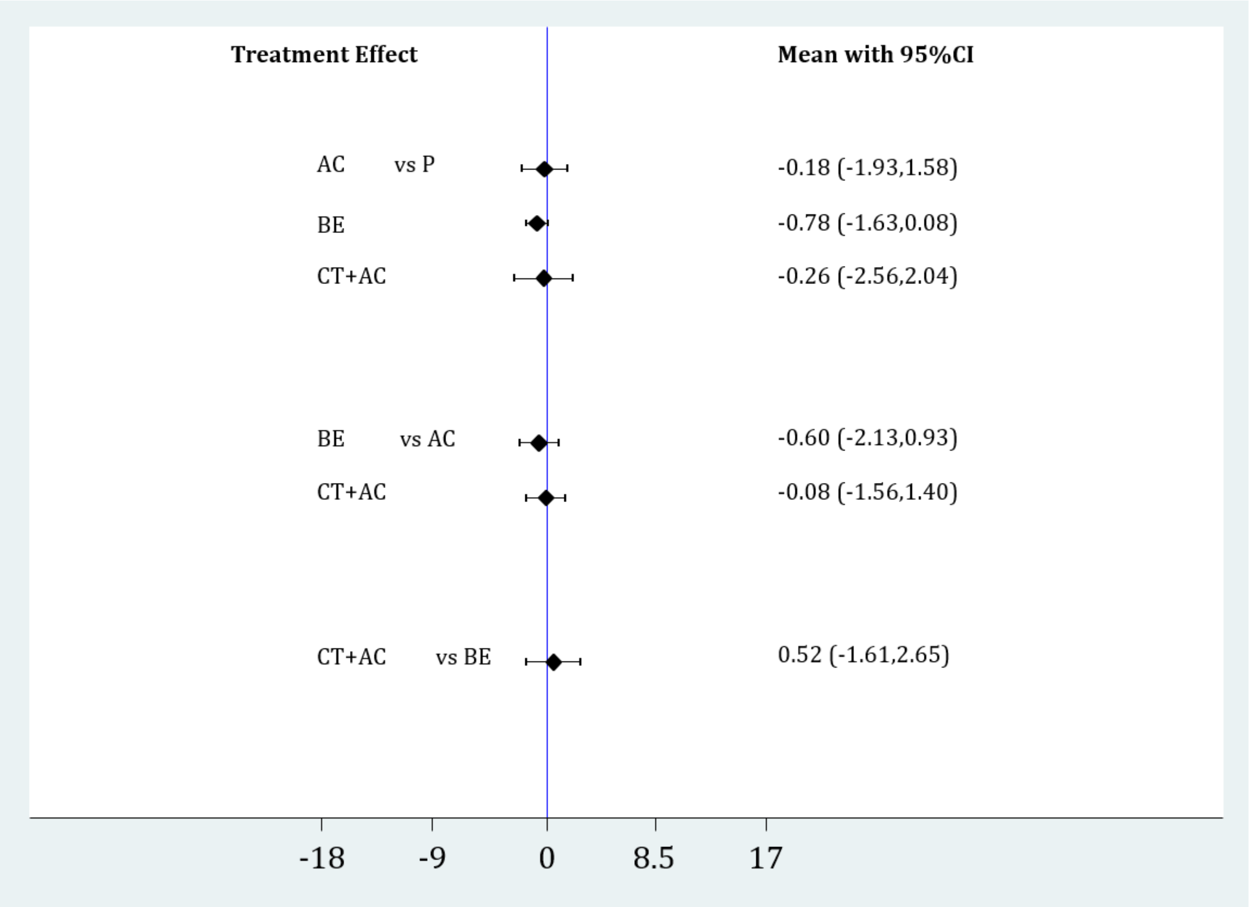

(chi-square for global inconsistency = 3.17, p = 0.0748)

Abbreviation: AC, active drug comparator; BE, bioavailability-enhanced curcuminoid preparations; CT, conventional curcuminoid preparations; CT + AC, conventional curcuminoid preparations + active drug comparator; P, placebo

eTable N2 Descriptive table for transitivity assessment of sensitivity analysis on follow-up period for WOMAC stiffness

_Contrast	Studies	Mean age in years (range), SD	Female in percentage (range)	Mean BMI in kg/m^2 (range), SD	Mean duration of baseline knee OA/pain in months (range), SD	Mean baseline WOMAC stiffness intensity (range), SD	Follow-up period in days (range)
BE vs. P	3	55.64 (53.12 to 57.57),7.71	80.98 (73.7 to 88.6)	27.19 (24.92 to 29.64), 3,08	23.25 (21.69 to 24.8), 9.23	2.12 (0.28 to 4.72), 1.20	48 (42 to 60)
CT+AC vs. AC	1	50.25 (50.23 to 50.27), 8.36	64.45 (61 to 67.9)	27.86 (27.4 to 28.82), 5.41	NA	5.43 (5.31 to 5.55), 1.46	120
BE vs. AC	1	51.95 (50.8 to 53.1), 10.4	74.35 (72.6 to 76.1)	NA	NA	NA	42

Abbreviation: AC, active drug comparator; BE, bioavailability-enhanced curcuminoid preparations; CT + AC, conventional curcuminoid preparations + active drug comparator; P, placebo

eFigure N3 Sensitivity analysis of follow-up period for WOMAC function

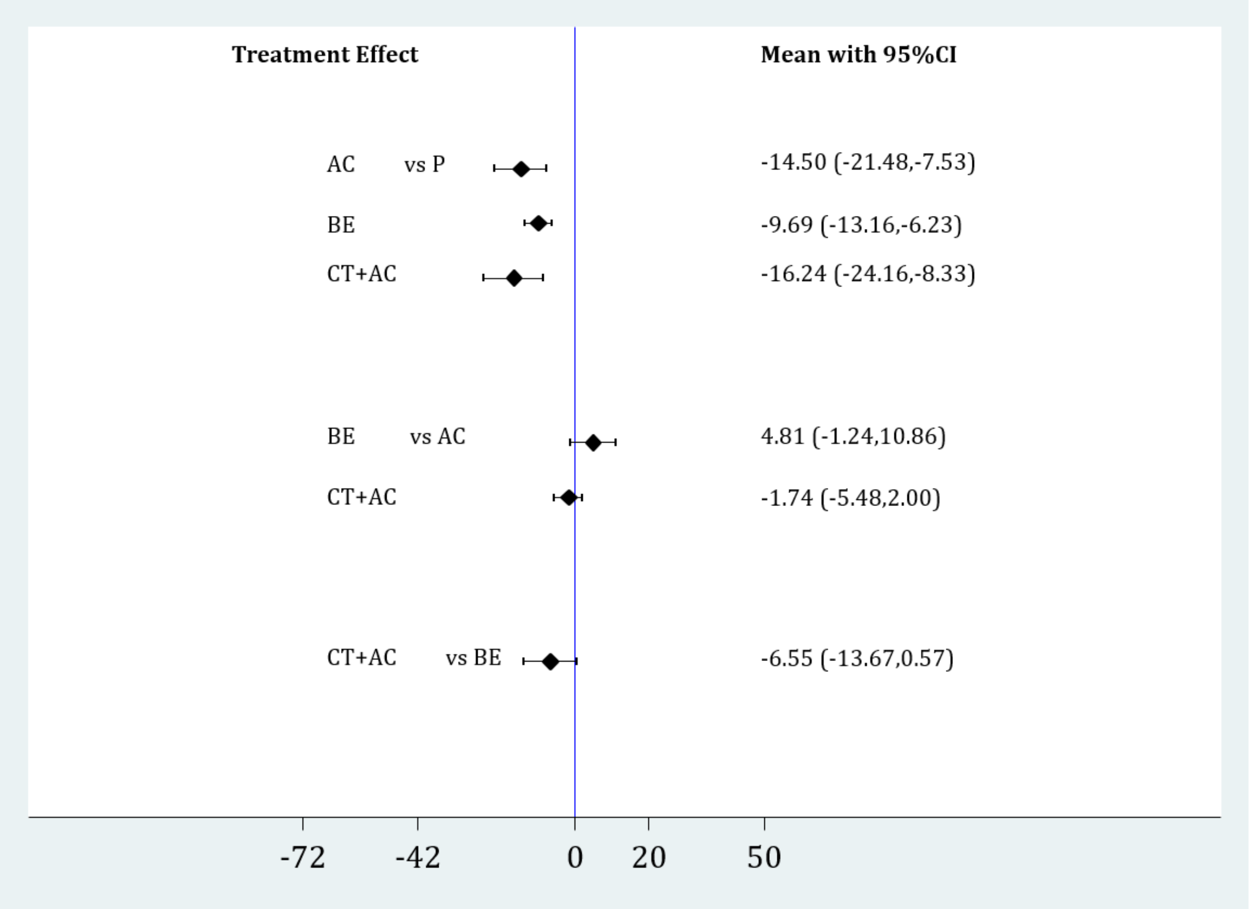

(chi-square for global inconsistency = 30.11, p = 0.0000)

Abbreviation: AC, active drug comparator; BE, bioavailability-enhanced curcuminoid preparations; CT, conventional curcuminoid preparations; CT + AC, conventional curcuminoid preparations + active drug comparator; P, placebo

eTable N3 Descriptive table for transitivity assessment of sensitivity analysis on follow-up period for WOMAC function

_Contrast	Studies	Mean age in years (range), SD	Female in percentage (range)	Mean BMI in kg/m^2 (range), SD	Mean duration of baseline knee OA/pain in months (range), SD	Mean baseline WOMAC function intensity (range), SD	Follow-up period in days (range)
BE vs. P	3	55.64 (53.12 to 57.57),7.71	80.98 (73.7 to 88.6)	27.19 (24.92 to 29.64), 3,08	23.25 (21.69 to 24.8), 9.23	27.28 (24.32 to 32.4), 6.81	48 (42 to 60)
CT+AC vs. AC	1	50.25 (50.23 to 50.27), 8.36	64.45 (61 to 67.9)	27.86 (27.4 to 28.82), 5.41	NA	52.51 (50.99 to 54.03), 6.08	120
BE vs. AC	1	51.95 (50.8 to 53.1), 10.4	74.35 (72.6 to 76.1)	NA	NA	NA	42

Abbreviation: AC, active drug comparator; BE, bioavailability-enhanced curcuminoid preparations; CT + AC, conventional curcuminoid preparations + active drug comparator; P, placebo

eFigure N4 Sensitivity analysis of critical risk of bias for WOMAC pain

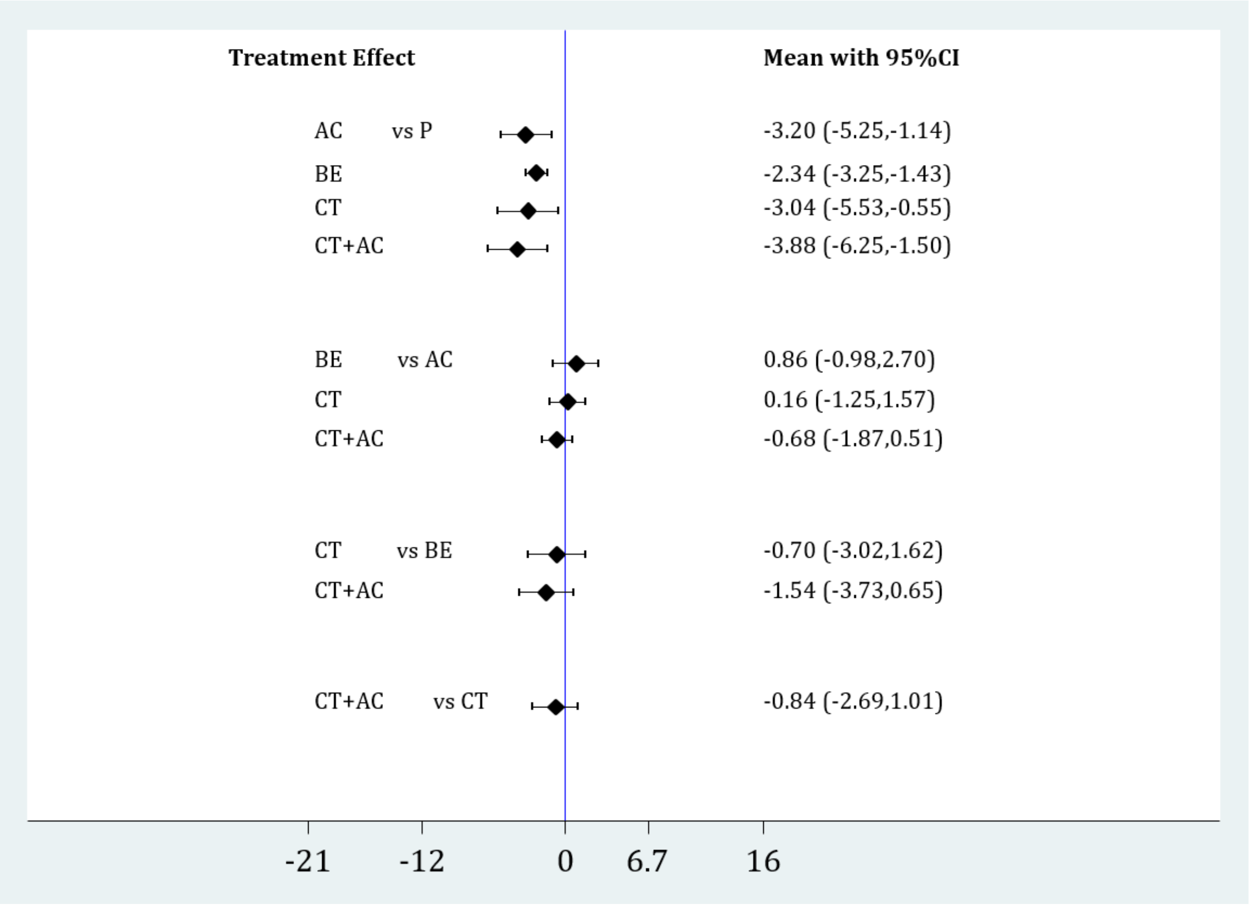

(chi-square for global inconsistency = 25.32, p = 0.0000)

Abbreviation: AC, active drug comparator; BE, bioavailability-enhanced curcuminoid preparations; CT, conventional curcuminoid preparations; CT + AC, conventional curcuminoid preparations + active drug comparator; P, placebo

eTable N4 Descriptive table for transitivity assessment of sensitivity analysis on critical risk of bias for WOMAC pain

_Contrast	Studies	Mean age in years (range), SD	Female in percentage (range)	Mean BMI in kg/m^2 (range), SD	Mean duration of baseline knee OA/pain in months (range), SD	Mean baseline WOMAC pain intensity (range), SD	Follow-up period in days (range)
BE vs. P	3	54.94 (53.12 to 56.54), 7.63	89.18 (80.60 to 96.6)	26.88 (24.92 to 28.81), 2.91	23.25 (21.69 to 24.8), 9.23	7.44 (5.85 to 8.47), 2.53	62 (42 to 84)
CT+AC vs. AC	1	50.25 (50.23 to 50.27), 8.36	64.45 (61 to 67.9)	27.86 (27.4 to 28.32), 5.41	NA	15.20 (15.1 to 15.29), 2.55	120
CT vs. AC	1	60.6 (60.3 to 60.9), 6.85	89.35 (86.9 to 91.81)	26.55 (26.5 to 26.6), 3.85	51.65 (51.3 to 52), 52.55	10.7 (10.6 to 10.8), 3.5	28
BE vs. AC	1	51.95 (50.8 to 53.1), 10.4	74.35 (72.6 to 76.1)	NA	NA	NA	42

Abbreviation: AC, active drug comparator; BE, bioavailability-enhanced curcuminoid preparations; CT, conventional curcuminoid preparations; CT + AC, conventional curcuminoid preparations + active drug comparator; P, placebo

eFigure N5 Sensitivity analysis of critical risk of bias for WOMAC stiffness

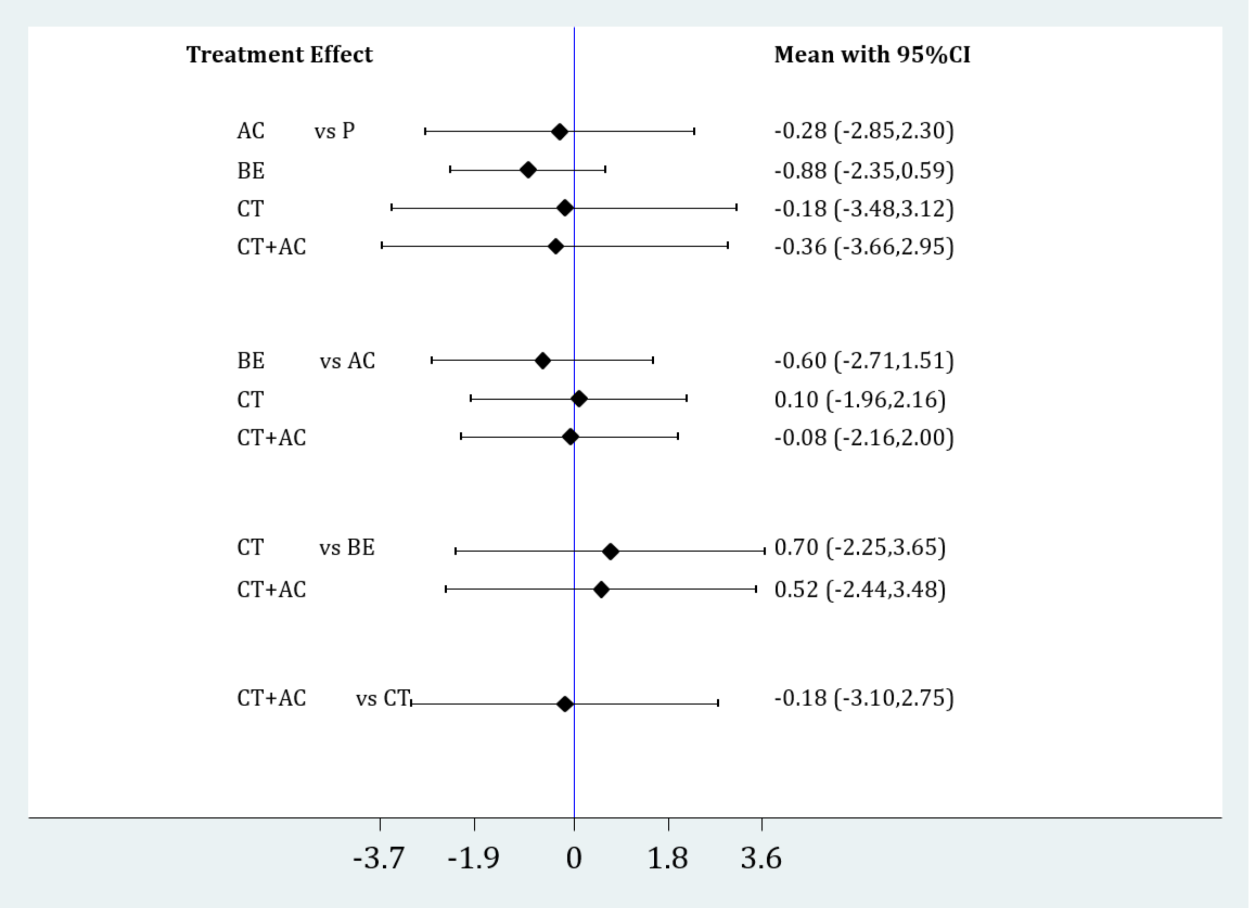

(chi-square for global inconsistency = 1.36, p = 0.2427)

Abbreviation: AC, active drug comparator; BE, bioavailability-enhanced curcuminoid preparations; CT, conventional curcuminoid preparations; CT + AC, conventional curcuminoid preparations + active drug comparator; P, placebo

eTable N5 Descriptive table for transitivity assessment of sensitivity analysis on critical risk of bias for WOMAC stiffness

_Contrast	Studies	Mean age in years (range), SD	Female in percentage (range)	Mean BMI in kg/m^2 (range), SD	Mean duration of baseline knee OA/pain in months (range), SD	Mean baseline WOMAC stiffness intensity (range), SD	Follow-up period in days (range)
BE vs. P	2	54.75 (53.12 to 56.54), 7.10	84.60 (80.6 to 88.6)	25.18 (24.92 to 25.44), 2.34	23.25 (21.69 to 24.8), 9.23	2.43 (0.28 to 4.72), 0.93	51 (42 to 60)
CT+AC vs. AC	1	50.25 (50.23 to 50.27), 8.36	64.45 (61 to 67.9)	27.86 (27.4 to 28.82), 5.41	NA	5.43 (5.31 to 5.55), 1.46	120
CT vs. AC	1	60.6 (60.3 to 60.9), 6.85	89.35 (86.9 to 91.81)	26.55 (26.5 to 26.6), 3.85	51.65 (51.3 to 52), 52.55	4.12 (4.08 to 4.16), 2.08	28
BE vs. AC	1	51.95 (50.8 to 53.1), 10.4	74.35 (72.6 to 76.1)	NA	NA	NA	42

Abbreviation: AC, active drug comparator; BE, bioavailability-enhanced curcuminoid preparations; CT, conventional curcuminoid preparations; CT + AC, conventional curcuminoid preparations + active drug comparator; P, placebo

eFigure N6 Sensitivity analysis of critical risk of bias for WOMAC function

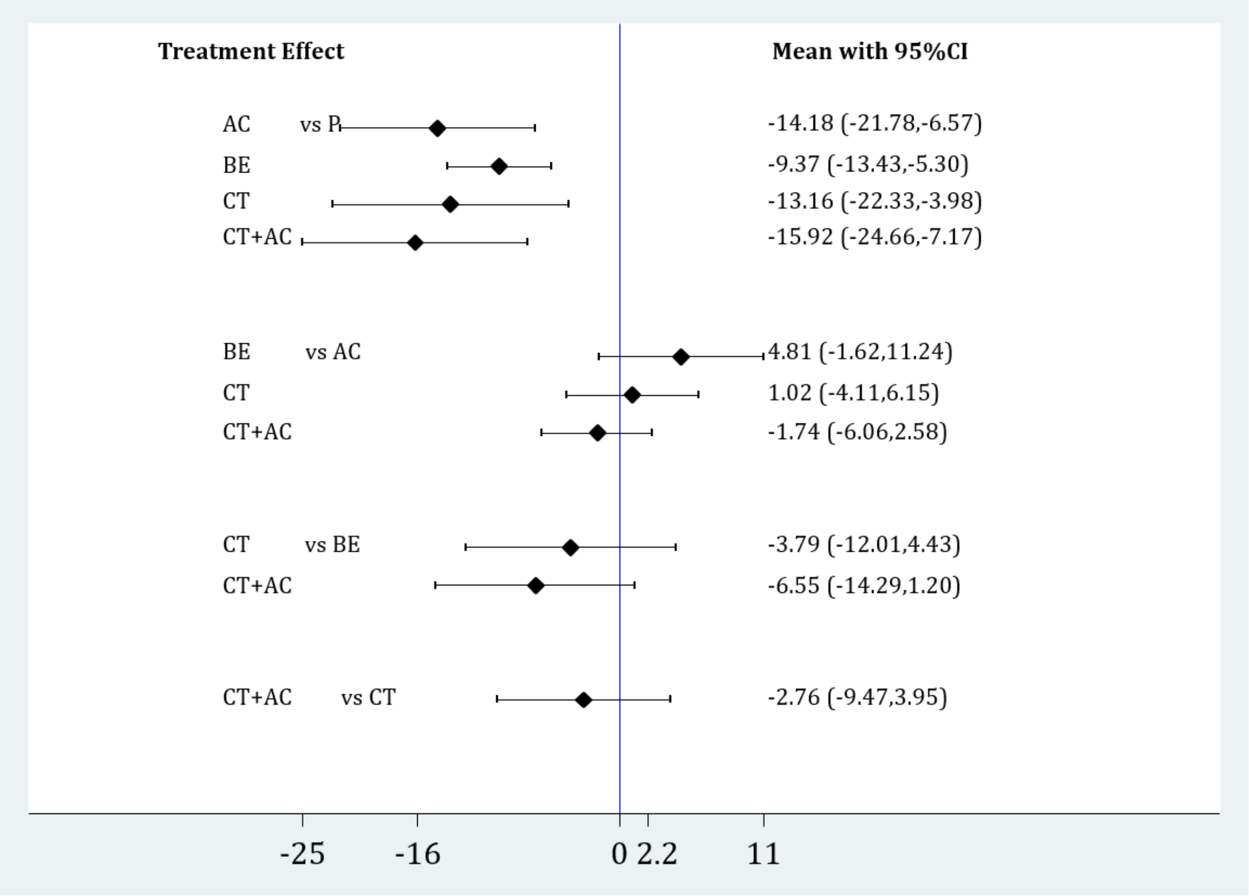

(chi-square for global inconsistency = 20.42, p = 0.0000)

Abbreviation: AC, active drug comparator; BE, bioavailability-enhanced curcuminoid preparations; CT, conventional curcuminoid preparations; CT + AC, conventional curcuminoid preparations + active drug comparator; P, placebo

eTable N6 Descriptive table for transitivity assessment of sensitivity analysis on critical risk of bias for WOMAC function

_Contrast	Studies	Mean age in years (range), SD	Female in percentage (range)	Mean BMI in kg/m^2 (range), SD	Mean duration of baseline knee OA/pain in months (range), SD	Mean baseline WOMAC function intensity (range), SD	Follow-up period in days (range)
BE vs. P	2	54.75 (53.12 to 56.54), 7.10	84.60 (80.6 to 88.6)	25.18 (24.92 to 25.44), 2.34	23.25 (21.69 to 24.8), 9.23	24.87 (24.32 to 25.56), 6.67	51 (42 to 60)
CT+AC vs. AC	1	50.25 (50.23 to 50.27), 8.36	64.45 (61 to 67.9)	27.86 (27.4 to 28.82), 5.41	NA	52.51 (50.99 to 54.03), 6.08	120
CT vs. AC	1	60.6 (60.3 to 60.9), 6.85	89.35 (86.9 to 91.81)	26.55 (26.5 to 26.6), 3.85	51.65 (51.3 to 52), 52.55	35.36 (34.68 to 36.04), 12.92	28
BE vs. AC	1	51.95 (50.8 to 53.1), 10.4	74.35 (72.6 to 76.1)	NA	NA	NA	42

Abbreviation: AC, active drug comparator; BE, bioavailability-enhanced curcuminoid preparations; CT, conventional curcuminoid preparations; CT + AC, conventional curcuminoid preparations + active drug comparator; P, placebo

eFigure N7 Sensitivity analysis of critical risk of bias for VAS

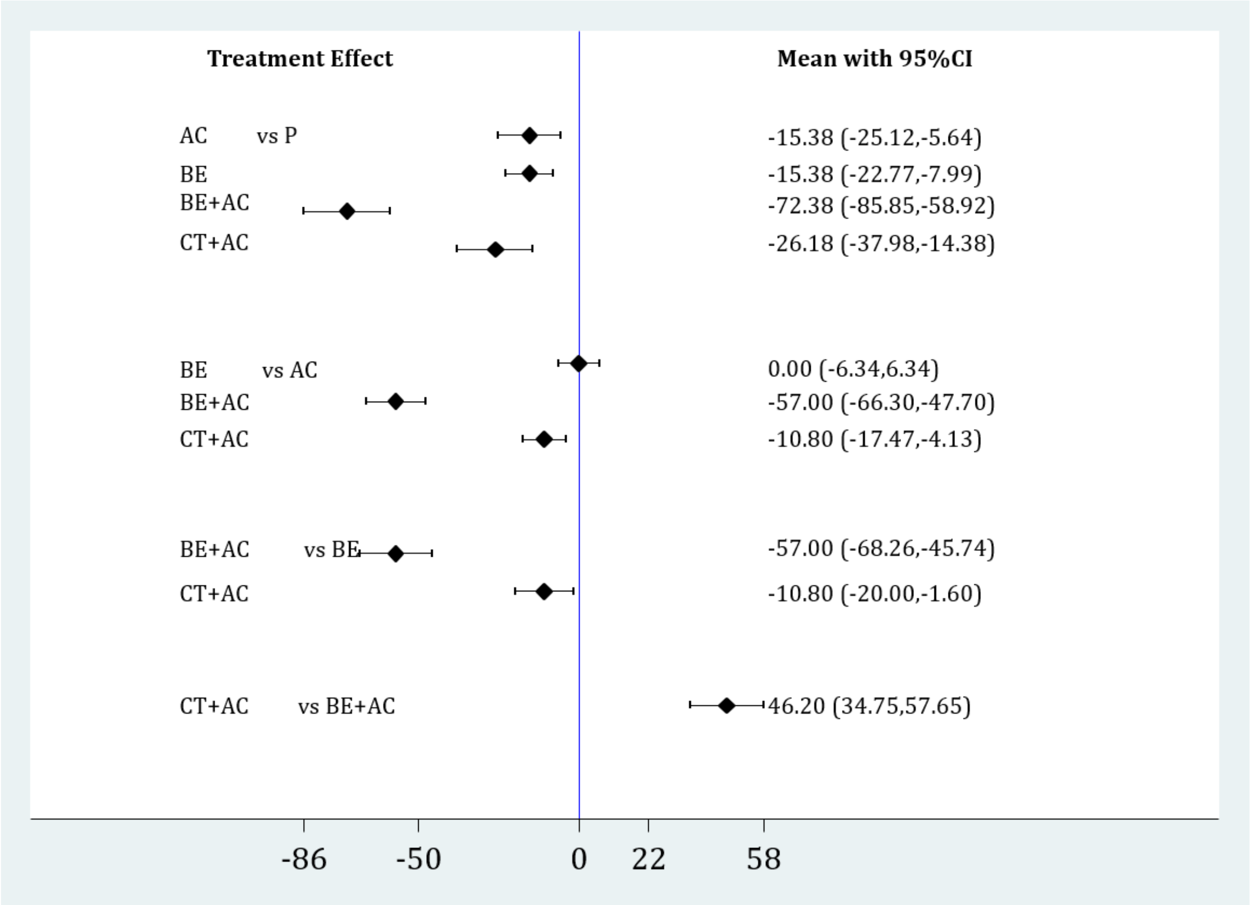

(chi-square for global inconsistency = 16.64, *p* = 0.0000)

Abbreviation: AC, active drug comparator; BE, bioavailability-enhanced curcuminoid preparations; BE + AC, bioavailability-enhanced curcuminoid preparations + active drug comparator; CT + AC, conventional curcuminoid preparations + active drug comparator; P, placebo

eTable N7 Descriptive table for transitivity assessment of sensitivity analysis on critical risk of bias for VAS

_Contrast	Studies	Mean age in years (range), SD	Female in percentage (range)	Mean BMI in kg/m^2 (range), SD	Mean duration of baseline knee OA/pain in months (range), SD	Mean baseline VAS pain intensity (range), SD	Follow-up period in days (range)
BE vs. P	2	58.20 (53.12 to 63.3), 8.28	78.90 (75.6 to 82.19)	27.42 (24.92 to 29.89), 3.73	87.68 (84.16 to 91.2), 92.57	57.04 (52.37 to 63.09), 9.49	75 (60 to 90)
BE vs. AC	1	52.62 (52.14 to 53.09), 3.97	33.07 (30.43 to 35.71)	NA	7.43 (7.4 to 7.45), 3.34	78.25 (78.1 to 78.4), 6.80	28
BE+AC vs. AC	1	48.70 (48.26 to 49.13), 5.46	100	21.95 (21.9 to 22), 1.45	54.96 (53.52 to 56.4), 30.21	81.95 (79.3 to 84.6), 17.23	90
CT+AC vs. AC	1	50.25 (50.23 to 50.27), 8.36	64.45 (61 to 67.9)	27.86 (27.4 to 28.82), 5.41	NA	78 (76.6 to 79.4), 12.08	120

Abbreviation: AC, active drug comparator; BE, bioavailability-enhanced curcuminoid preparations; BE + AC, bioavailability-enhanced curcuminoid preparations + active drug comparator; CT + AC, conventional curcuminoid preparations + active drug comparator; P, placebo

eFigure N8 Sensitivity analysis of small-study effects for WOMAC pain

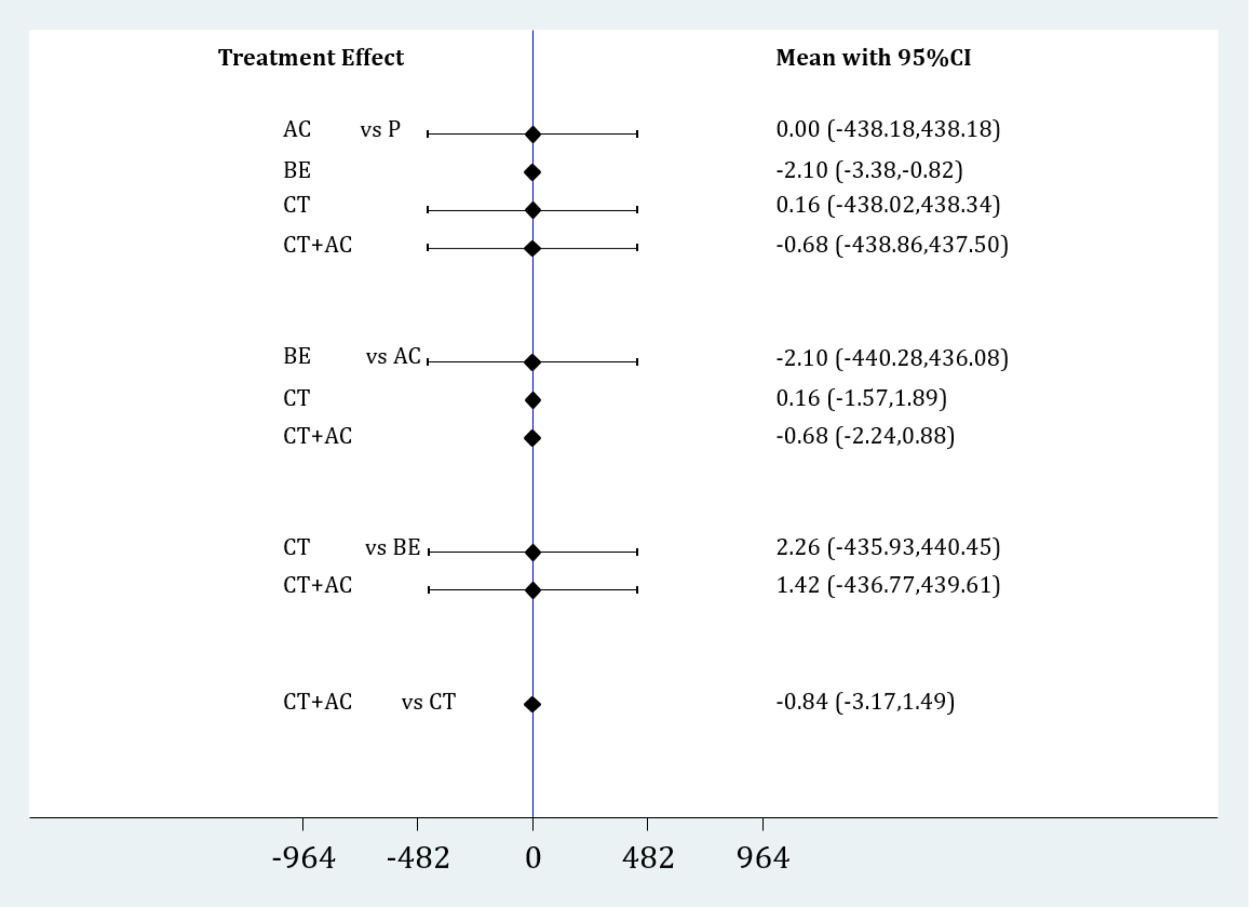

(chi-square for global consistency = 36.59, p = 0.0000)

Abbreviation: AC, active drug comparator; BE, bioavailability-enhanced curcuminoid preparations; CT, conventional curcuminoid preparations; CT + AC, conventional curcuminoid preparations + active drug comparator; P, placebo

eTable N8 Descriptive table for transitivity assessment of sensitivity analysis on small-study effects for WOMAC pain

_Contrast	Studies	Mean age in years (range), SD	Female in percentage (range)	Mean BMI in kg/m^2 (range), SD	Mean duration of baseline knee OA/pain in months (range), SD	Mean baseline WOMAC pain intensity (range), SD	Follow-up period in days (range)
BE vs. P	2	54.76 (53.12 to 56.04), 8.56	93.75 (90.9 to 96.6)	26.88 (24.92 to 28.81), 2.91	NA	7.05 (5.85 to 8.24), 2.2	72 (60 to 84)
CT+AC vs. AC	1	50.25 (50.23 to 50.27), 8.36	64.45 (61 to 67.9)	27.86 (27.4 to 28.32), 5.41	NA	15.20 (15.1 to 15.29), 2.55	120
CT vs. AC	1	60.6 (60.3 to 60.9), 6.85	89.35 (86.9 to 91.81)	26.55 (26.5 to 26.6), 3.85	51.65 (51.3 to 52), 52.55	10.7 (10.6 to 10.8), 3.5	28

Abbreviation: AC, active drug comparator; BE, bioavailability-enhanced curcuminoid preparations; CT, conventional curcuminoid preparations; CT + AC, conventional curcuminoid preparations + active drug comparator; P, placebo

eFigure N9 Sensitivity analysis of small-study effects for WOMAC stiffness

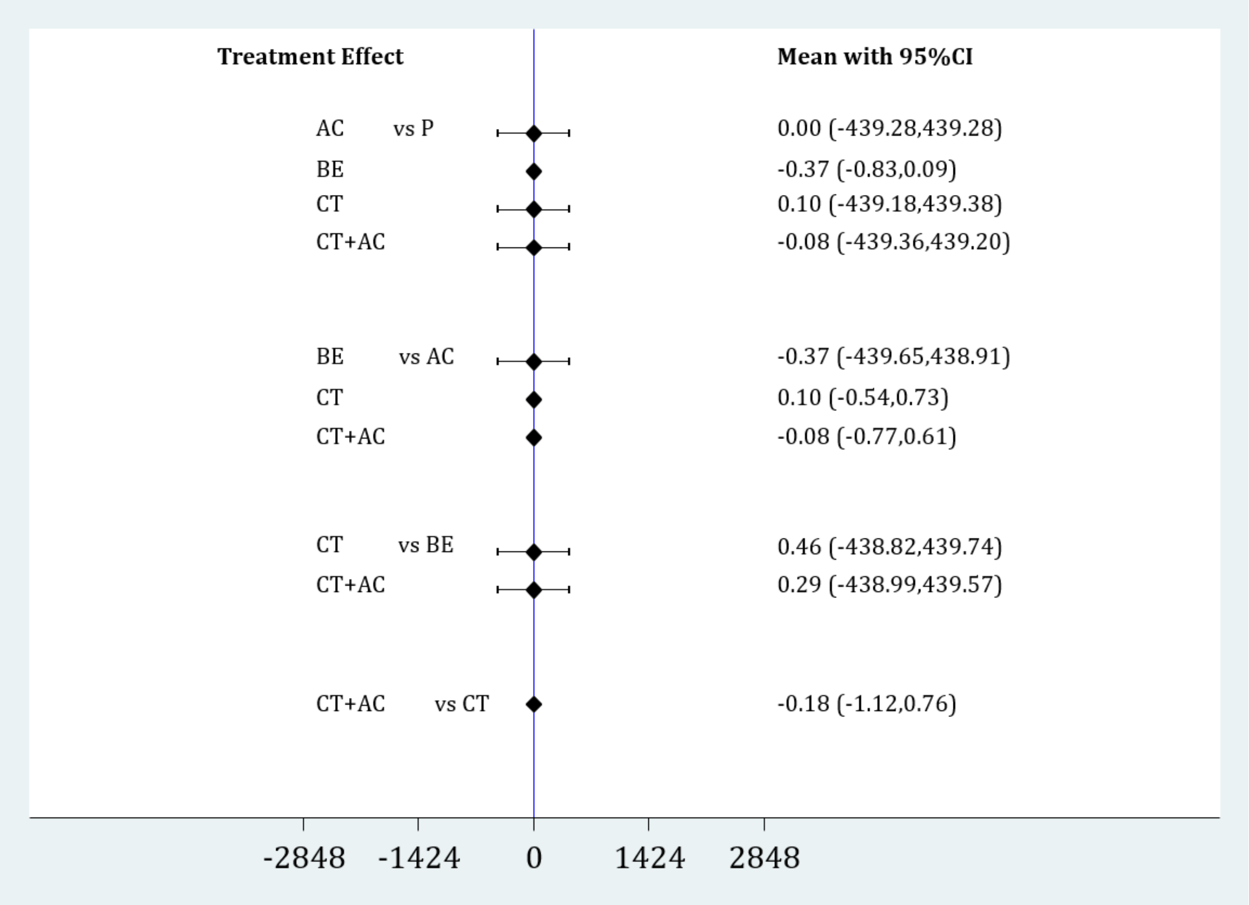

(chi-square for global consistency = 3.17, p = 0.748)

Abbreviation: AC, active drug comparator; BE, bioavailability-enhanced curcuminoid preparations; CT, conventional curcuminoid preparations; CT + AC, conventional curcuminoid preparations + active drug comparator; P, placebo

eTable N9 Descriptive table for transitivity assessment of sensitivity analysis on small-study effects for WOMAC stiffness

_Contrast	Studies	Mean age in years (range), SD	Female in percentage (range)	Mean BMI in kg/m^2 (range), SD	Mean duration of baseline knee OA/pain in months (range), SD	Mean baseline WOMAC stiffness intensity (range), SD	Follow-up period in days (range)
BE vs. P	2	56.39 (54.11 to 57.57), 7.35	80.98 (73.7 to 88.6)	29.20 (28.75 to 29.64), 3.82	23.25 (21.69 to 24.8), 9.23	0.87 (0.28 to 1.7), 1.32	42
CT+AC vs. AC	1	50.25 (50.23 to 50.27), 8.36	64.45 (61 to 67.9)	27.86 (27.4 to 28.82), 5.41	NA	5.43 (5.31 to 5.55), 1.46	120
CT vs. AC	1	60.6 (60.3 to 60.9), 6.85	89.35 (86.9 to 91.81)	26.55 (26.5 to 26.6), 3.85	51.65 (51.3 to 52), 52.55	4.12 (4.08 to 4.16), 2.08	28

Abbreviation: AC, active drug comparator; BE, bioavailability-enhanced curcuminoid preparations; CT, conventional curcuminoid preparations; CT + AC, conventional curcuminoid preparations + active drug comparator; P, placebo

eFigure N10 Sensitivity analysis of small-study effects for WOMAC function

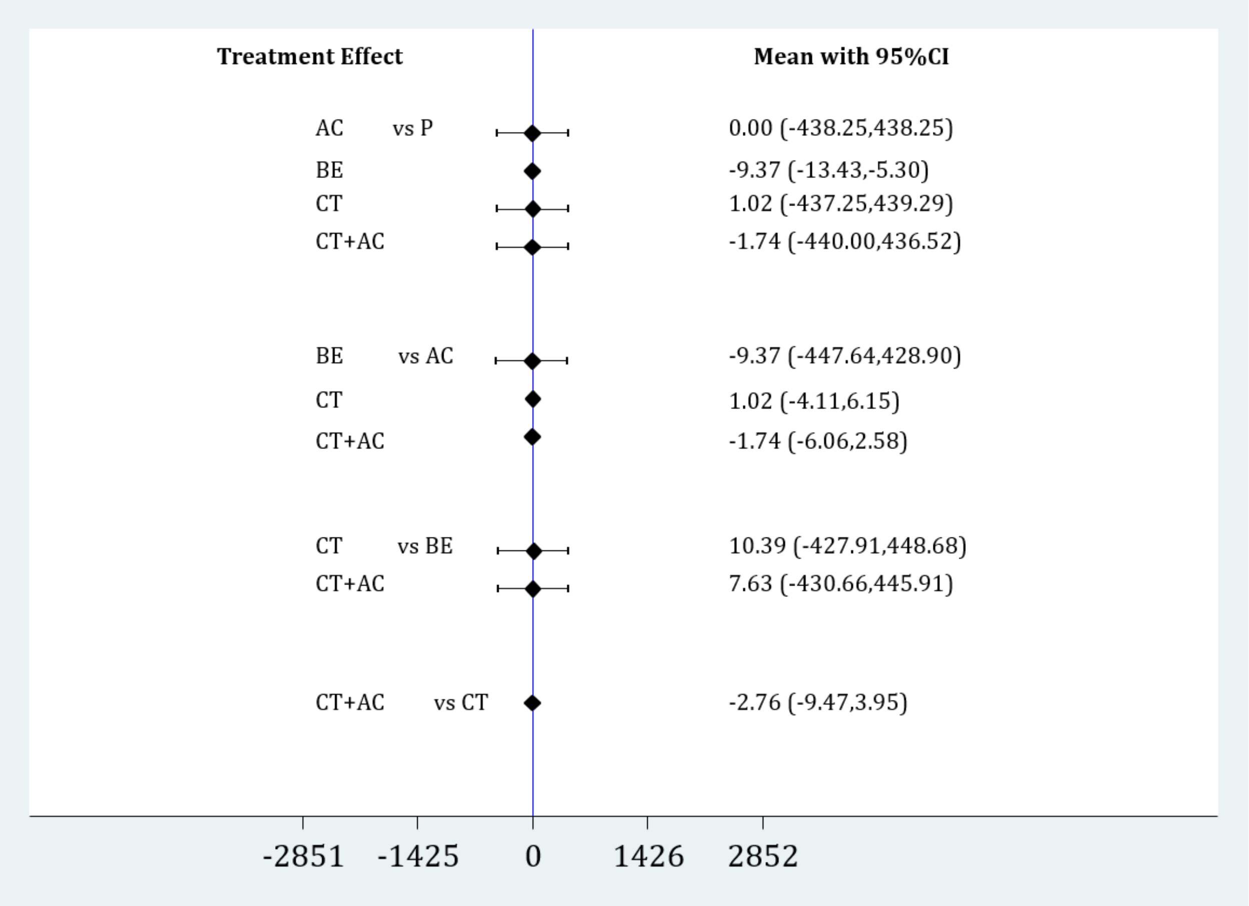

(chi-square for global consistency = 30.11, p = 0.0000)

Abbreviation: AC, active drug comparator; BE, bioavailability-enhanced curcuminoid preparations; CT, conventional curcuminoid preparations; CT + AC, conventional curcuminoid preparations + active drug comparator; P, placebo

eTable N10 Descriptive table for transitivity assessment of sensitivity analysis on small-study effects for WOMAC function

_Contrast	Studies	Mean age in years (range), SD	Female in percentage (range)	Mean BMI in kg/m^2 (range), SD	Mean duration of baseline knee OA/pain in months (range), SD	Mean baseline WOMAC stiffness intensity (range), SD	Follow-up period in days (range)
BE vs. P	2	54.75 (53.12 to 56.54), 7.10	84.60 (80.6 to 88.6)	25.18 (24.92 to 25.44), 2.34	23.25 (21.69 to 24.8), 9.23	24.87 (24.32 to 25.56), 6.67	51 (42 to 60)
CT+AC vs. AC	1	50.25 (50.23 to 50.27), 8.36	64.45 (61 to 67.9)	27.86 (27.4 to 28.82), 5.41	NA	52.51 (50.99 to 54.03), 6.08	120
CT vs. AC	1	60.6 (60.3 to 60.9), 6.85	89.35 (86.9 to 91.81)	26.55 (26.5 to 26.6), 3.85	51.65 (51.3 to 52), 52.55	35.36 (34.68 to 36.04), 12.92	28

Abbreviation: AC, active drug comparator; BE, bioavailability-enhanced curcuminoid preparations; CT, conventional curcuminoid preparations; CT + AC, conventional curcuminoid preparations + active drug comparator; P, placebo

eFigure N11 Sensitivity analysis of small-study effects for VAS

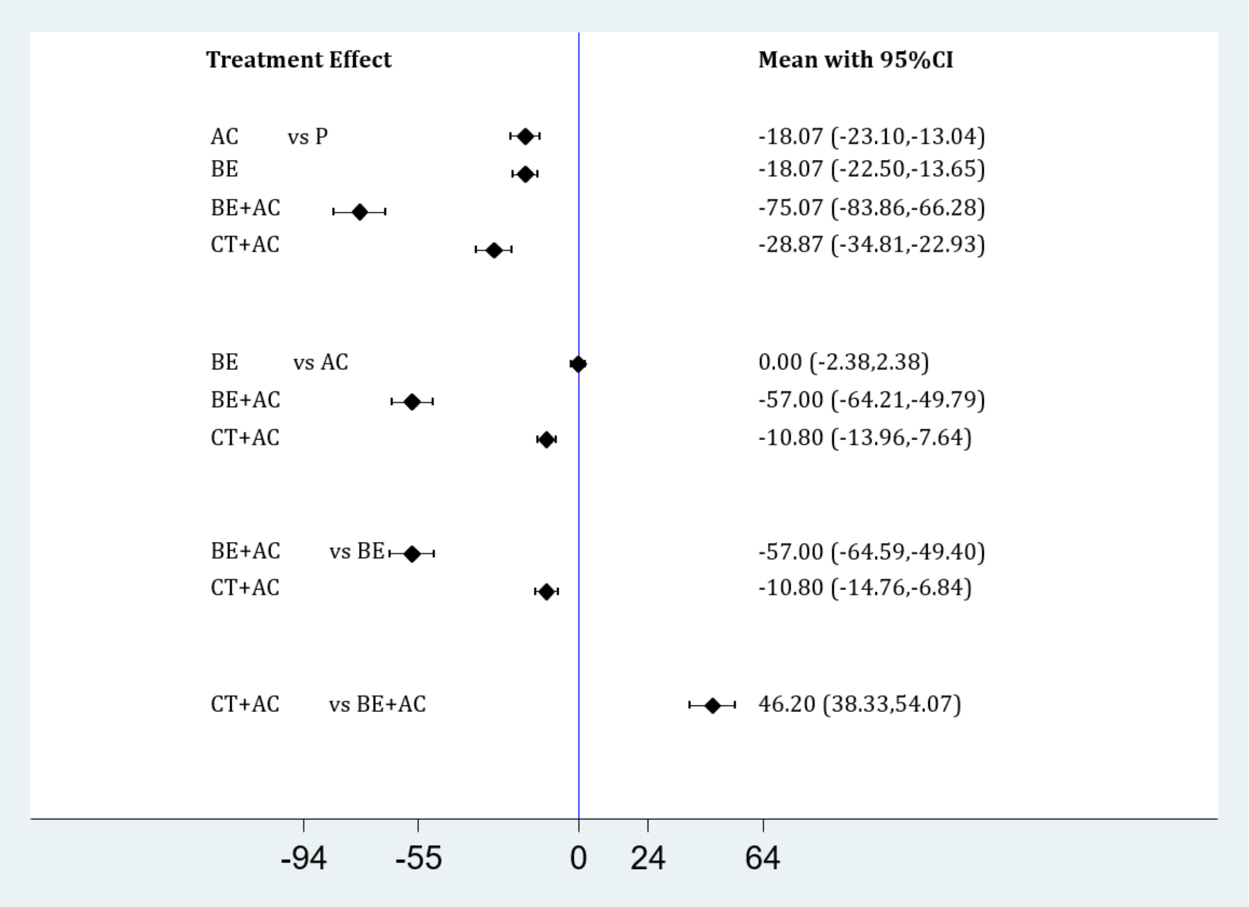

(chi-square for global consistency = 27.16, p = 0.0000)

Abbreviation: AC, active drug comparator; BE, bioavailability-enhanced curcuminoid preparations; BE + AC, bioavailability-enhanced curcuminoid preparations + active drug comparator; CT + AC, conventional curcuminoid preparations + active drug comparator; P, placebo

eTable N11 Descriptive table for transitivity assessment of sensitivity analysis on small-study effects for VAS

_Contrast	Studies	Mean age in years (range), SD	Female in percentage (range)	Mean BMI in kg/m^2 (range), SD	Mean duration of baseline knee OA/pain in months (range), SD	Mean baseline VAS pain intensity (range), SD	Follow-up period in days (range)
BE vs. P	2	55.81 (53.12 to 57.57), 8.65	77.35 (73.7 to 81)	27.19 (24.92 to 29.64), 3.08	NA	57.64 (52.37 to 66.32), 10.60	51 (42 to 60)
BE vs. AC	1	52.62 (52.14 to 53.09), 3.97	33.07 (30.43 to 35.71)	NA	7.43 (7.4 to 7.45), 3.34	78.25 (78.1 to 78.4), 6.80	28
BE+AC vs. AC	1	48.70 (48.26 to 49.13), 5.46	100	21.95 (21.9 to 22), 1.45	54.96 (53.52 to 56.4), 30.21	81.95 (79.3 to 84.6), 17.23	90
CT+AC vs. AC	1	50.25 (50.23 to 50.27), 8.36	64.45 (61 to 67.9)	27.86 (27.4 to 28.82), 5.41	NA	78 (76.6 to 79.4), 12.08	120

Abbreviation: AC, active drug comparator; BE, bioavailability-enhanced curcuminoid preparations; BE + AC, bioavailability-enhanced curcuminoid preparations + active drug comparator; CT + AC, conventional curcuminoid preparations + active drug comparator; P, placebo

eFigure N12 Adjusted funnel plot of small-study effects for WOMAC pain

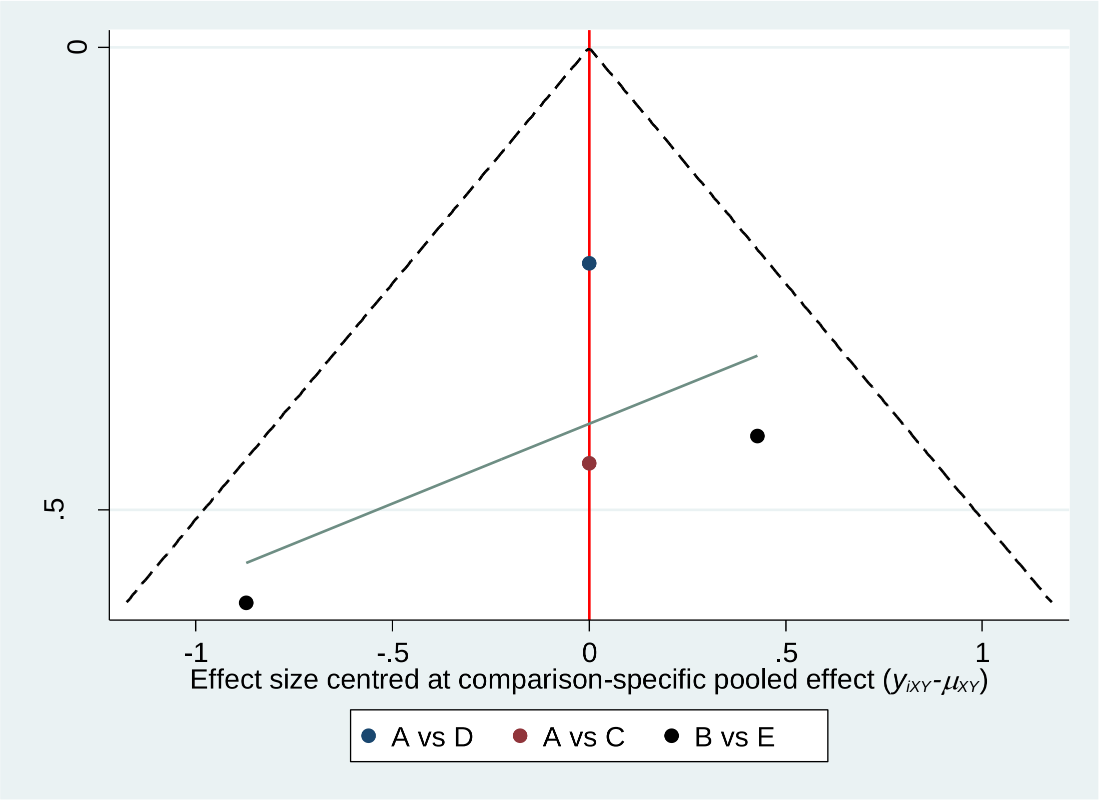

Abbreviation: A = AC, active drug comparator; B = BE, bioavailability-enhanced curcuminoid preparations; C = CT, conventional curcuminoid preparations; D = CT + AC, conventional curcuminoid preparations + active drug comparator; E = P, placebo

eFigure N13 Adjusted funnel plot of small-study effects for WOMAC stiffness

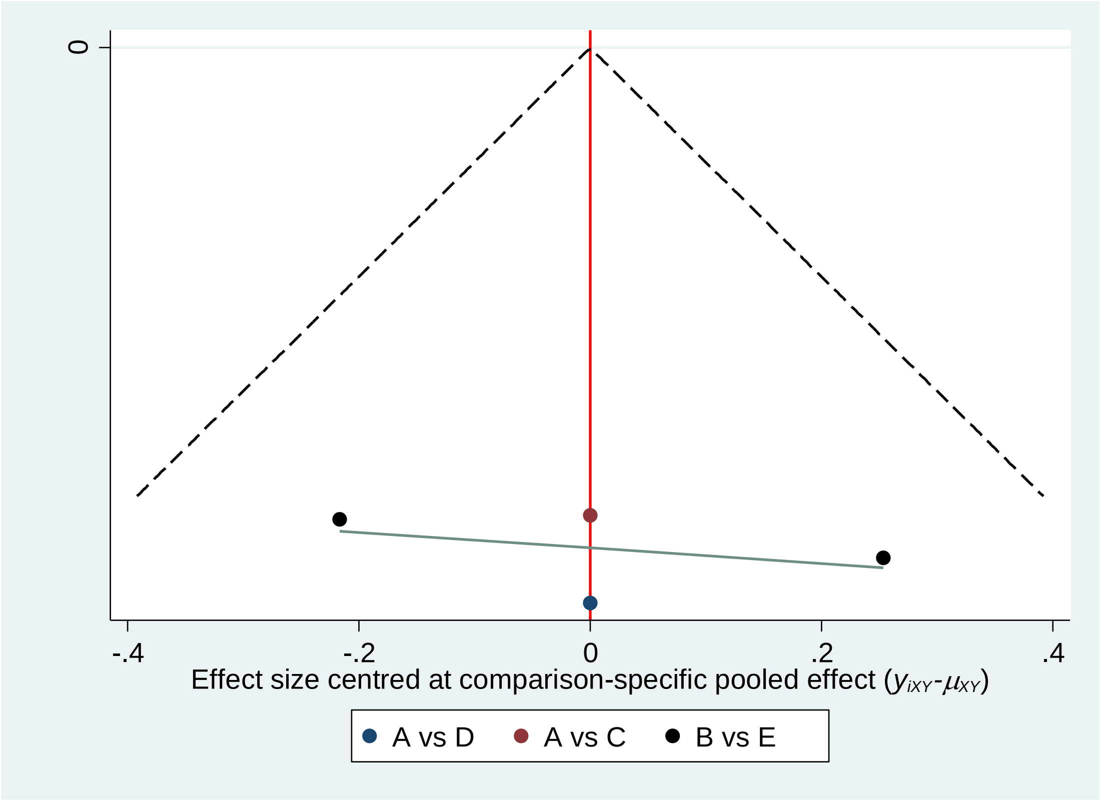

Abbreviation: A = AC, active drug comparator; B = BE, bioavailability-enhanced curcuminoid preparations; C = CT, conventional curcuminoid preparations; D = CT + AC, conventional curcuminoid preparations + active drug comparator; E = P, placebo

eFigure N14 Adjusted funnel plot of small-study effects for WOMAC function

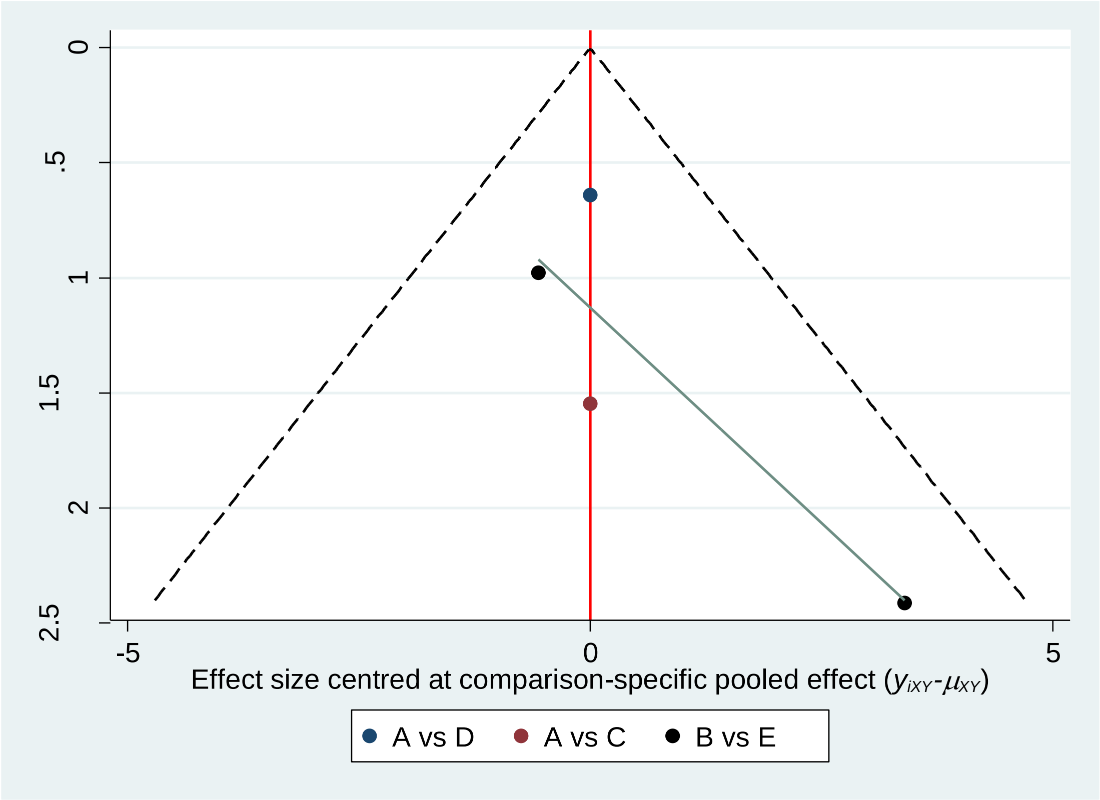

Abbreviation: A = AC, active drug comparator; B = BE, bioavailability-enhanced curcuminoid preparations; C = CT, conventional curcuminoid preparations; D = CT + AC, conventional curcuminoid preparations + active drug comparator; E = P, placebo

eFigure N15 Adjusted funnel plot of small-study effects for VAS

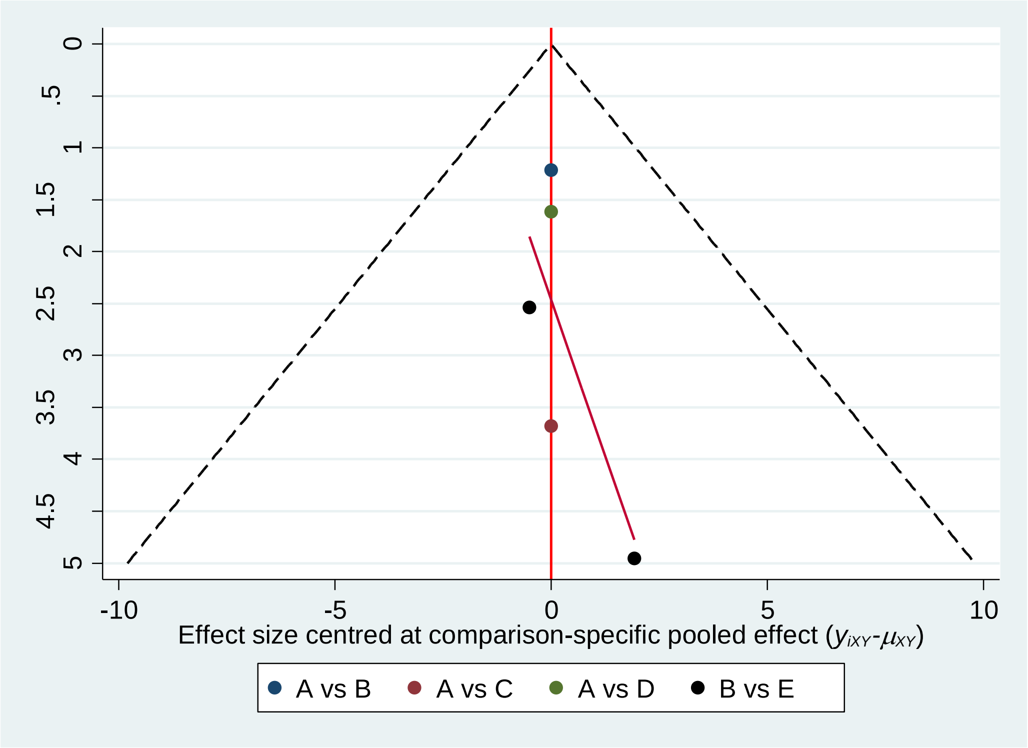

Abbreviation: A = AC, active drug comparator; B = BE, bioavailability-enhanced curcuminoid preparations; C = BE + AC, bioavailability-enhanced curcuminoid preparations + active drug comparator; D = CT + AC, conventional curcuminoid preparations + active drug comparator; E = P, placebo

Appendix O

References

1. Thorlund K, Walter SD, Johnston BC, Furukawa TA, Guyatt GH. Pooling health-related quality of life outcomes in meta-analysis-a tutorial and review of methods for enhancing interpretability. Res Synth Methods. 2011; doi: 10.1002/jrsm.46.

2. Singhal S, Hasan N, Nirmal K, Chawla R, Chawla S, Kalra BS, et al. Bioavailable turmeric extract for knee osteoarthritis: a randomized, non-inferiority trial versus paracetamol. Trials. 2021; doi: 10.1186/s13063-021-05053-7.

3. Haroyan A, Mukuchyan V, Mkrtchyan N, Minasyan N, Gasparyan S, Sargsyan A, et al. Efficacy and safety of curcumin and its combination with boswellic acid in osteoarthritis: a comparative, randomized, double-blind, placebo-controlled study. BMC Complement Altern Med. 2018; doi: 10.1186/s12906-017-2062-z.

4. Hashemzadeh K, Davoudian N, Jaafari MR, Mirfeizi Z. The effect of nanocurcumin in improvement of knee osteoarthritis: a randomized clinical trial. Curr Rheumatol Rev. 2020; doi: 10.2174/1874471013666191223152658.

5. Panda SK, Nirvanashetty S, Parachur VA, Mohanty N, Swain T. A randomized, double blind, placebo controlled, parallel-group study to evaluate the safety and efficacy of Curene® versus placebo in reducing symptoms of knee osteoarthritis. Biomed Res Int. 2018; doi: 10.1155/2018/5291945.

6. Panahi Y, Rahimnia AR, Sharafi M, Alishiri G, Saburi A, Sahebkar A. Curcuminoid treatment for knee osteoarthritis: a randomized double-blind placebo-controlled trial. Phytother Res. 2014; doi: 10.1002/ptr.5174.

7. Srivastava S, Saksena AK, Khattri S, Kumar S, Dagur RS. *Curcuma longa* extract reduces inflammatory and oxidative stress biomarkers in osteoarthritis of knee: a four-month, double-blind, randomized, placebo-controlled trial. Inflammopharmacology. 2016; doi: 10.1007/s10787-016-0289-9.

8. Kuptniratsaikul V, Dajpratham P, Taechaarpornkul W, Buntragulpoontawee M, Lukkanapichonchut P, Chootip C, et al. Efficacy and safety of *Curcuma domestica* extracts compared with ibuprofen in patients with knee osteoarthritis: a multicenter study. Clin Interv Aging. 2014; doi: 10.2147/cia.S58535.

9. Shep D, Khanwelkar C, Gade P, Karad S. Safety and efficacy of curcumin versus diclofenac in knee osteoarthritis: a randomized open-label parallel-arm study. Trials. 2019; doi: 10.1186/s13063-019-3327-2.

10. Atabaki M, Shariati-Sarabi Z, Tavakkol-Afshari J, Mohammadi M. Significant immunomodulatory properties of curcumin in patients with osteoarthritis; a successful clinical trial in Iran. Int Immunopharmacol. 2020; doi: 10.1016/j.intimp.2020.106607.

11. Henrotin Y, Malaise M, Wittoek R, de Vlam K, Brasseur JP, Luyten FP, et al. Bio-optimized *Curcuma longa* extract is efficient on knee osteoarthritis pain: a double-blind multicenter randomized placebo controlled three-arm study. Arthritis Res Ther. 2019; doi: 10.1186/s13075-019-1960-5.

12. Madhu K, Chanda K, Saji MJ. Safety and efficacy of *Curcuma longa* extract in the treatment of painful knee osteoarthritis: a randomized placebo-controlled trial. Inflammopharmacology. 2013; doi: 10.1007/s10787-012-0163-3.

13. Lopresti AL, Smith SJ, Jackson-Michel S, Fairchild T. An investigation into the effects of a curcumin extract (Curcugen(®)) on osteoarthritis pain of the knee: a randomised, double-blind, placebo-controlled study. Nutrients. 2021; doi: 10.3390/nu14010041.

14. Nakagawa Y, Mukai S, Yamada S, Matsuoka M, Tarumi E, Hashimoto T, et al. Short-term effects of highly-bioavailable curcumin for treating knee osteoarthritis: a randomized, double-blind, placebo-controlled prospective study. J Orthop Sci. 2014; doi: 10.1007/s00776-014-0633-0.

15. Gupte PA, Giramkar SA, Harke SM, Kulkarni SK, Deshmukh AP, Hingorani LL, et al. Evaluation of the efficacy and safety of Capsule Longvida(®) optimized curcumin (solid lipid curcumin particles) in knee osteoarthritis: a pilot clinical study. J Inflamm Res. 2019; doi: 10.2147/jir.S205390.

16. Pinsornsak P, Niempoog S. The efficacy of *Curcuma longa* L. extract as an adjuvant therapy in primary knee osteoarthritis: a randomized control trial. J Med Assoc Thai. 2012;95 Suppl 1:S51-S8.

17. Kuptniratsaikul V, Thanakhumtorn S, Chinswangwatanakul P, Wattanamongkonsil L, Thamlikitkul V. Efficacy and safety of *Curcuma domestica* extracts in patients with knee osteoarthritis. J Altern Complement Med. 2009; doi: 10.1089/acm.2008.0186.

18. Wang Z, Jones G, Winzenberg T, Cai G, Laslett LL, Aitken D, et al. Effectiveness of *Curcuma longa* extract for the treatment of symptoms and effusion-synovitis of knee osteoarthritis : a randomized trial. Ann Intern Med. 2020; doi: 10.7326/m20-0990.

19. Jamwal R. Bioavailable curcumin formulations: A review of pharmacokinetic studies in healthy volunteers. Journal of Integrative Medicine. 2018; doi: https://doi.org/10.1016/j.joim.2018.07.001.
