# Supplementary material for: Effect of turmeric products on knee osteoarthritis: a systematic review and network meta-analysis
Source: BMC Complement Med Ther. 2025 Jul 29;25:292. doi: 10.1186/s12906-025-05045-z (PMC12309109; doi:10.1186/s12906-025-05045-z)
Supplement: Supplementary file 2 — Supplementary Material 2 [file 12906_2025_5045_MOESM2_ESM.docx]

**Supplementary Material**

**Online Supplementary Content**

**Contents**

[**Appendix A** 8](#_Toc200707225)

[**PRISMA NMA checklist** 8](#_Toc200707226)

[eTable A1 PRISMA NMA Checklist of Items to Include When Reporting A Systematic Review Involving a Network Meta-analysis 8](#_Toc200707227)

[**Appendix B** 12](#_Toc200707228)

[**Unit standardization** 12](#_Toc200707229)

[eTable B1 Different units standardized to Likert units for WOMAC pain 12](#_Toc200707230)

[eTable B2 Different units standardized to Likert units for WOMAC stiffness 14](#_Toc200707231)

[eTable B3 Different units standardized to Likert units for WOMAC function 15](#_Toc200707232)

[eTable B4 Different units standardized to 0–100 units for VAS 16](#_Toc200707233)

[**Appendix C** 17](#_Toc200707234)

[**Search algorithms** 17](#_Toc200707235)

[eTable C1 PICO domains and search terms 17](#_Toc200707236)

[eTable C2 Search algorithms 18](#_Toc200707237)

[**Appendix D** 20](#_Toc200707238)

[**Additional characteristics of all included studies** 20](#_Toc200707239)

[eTable D1 Regimen characteristics of included studies 20](#_Toc200707240)

[eTable D2 Baseline and follow-up characteristics of all included studies 24](#_Toc200707241)

[eTable D3 Extracted data of outcomes of interest 27](#_Toc200707242)

[eTable D4 Detailed characteristics of curcuminoid preparations included in the analysis 30](#_Toc200707243)

[**Appendix E** 31](#_Toc200707244)

[**Risk of bias assessment** 31](#_Toc200707245)

[eFigure E Risk of bias graph 31](#_Toc200707246)

[eTable E Summarized risk of bias of included studies using ROB2.0 32](#_Toc200707247)

[**Appendix F** 33](#_Toc200707248)

[**Results of meta-analyses of direct comparisons of treatment options** 33](#_Toc200707249)

[eFigure F1 Mean difference (and 95% CI) for WOMAC pain 34](#_Toc200707250)

[eFigure F2 Mean difference (and 95% CI) for WOMAC stiffness 35](#_Toc200707251)

[eFigure F3 Mean difference (and 95% CI) for WOMAC function 36](#_Toc200707252)

[eFigure F4 Mean difference (and 95% CI) for VAS 37](#_Toc200707253)

[**Appendix G** 39](#_Toc200707254)

[**Assessment of global inconsistency for each outcome network in main analysis** 39](#_Toc200707255)

[eTable G Assessment of global inconsistency in networks using the ‘design-by-treatment’ interaction model 39](#_Toc200707256)

[**Appendix H** 40](#_Toc200707257)

[**Interval plots** 40](#_Toc200707258)

[eFigure H1 Interval plot of WOMAC pain 40](#_Toc200707259)

[eFigure H2 Interval plot of WOMAC stiffness 41](#_Toc200707260)

[eFigure H3 Interval plot of WOMAC function 42](#_Toc200707261)

[eFigure H4 Interval plot of VAS 43](#_Toc200707262)

[**Appendix I** 44](#_Toc200707263)

[**SUCRA ranking for WOMAC Pain, Stiffness, Function, and VAS** 44](#_Toc200707264)

[eFigure I1 SUCRA ranking among interventions on WOMAC pain 44](#_Toc200707265)

[eFigure I2 SUCRA ranking among interventions on WOMAC stiffness 45](#_Toc200707266)

[eFigure I3 SUCRA ranking among interventions on WOMAC function 46](#_Toc200707267)

[eFigure I4 SUCRA ranking among interventions on VAS 47](#_Toc200707268)

[**Appendix J** 48](#_Toc200707269)

[**Adjusted funnel plots** 48](#_Toc200707270)

[eFigure J1 Adjusted funnel plot for WOMAC pain 48](#_Toc200707271)

[eFigure J2 Adjusted funnel plot for WOMAC stiffness 49](#_Toc200707272)

[eFigure J3 Adjusted funnel plot for WOMAC function 50](#_Toc200707273)

[eFigure J4 Adjusted funnel plot for VAS 51](#_Toc200707274)

[**Appendix K** 52](#_Toc200707275)

[**Transitivity assessment tables** 52](#_Toc200707276)

[eTable K1 Descriptive table for the transitivity assessment of potential effect modifiers on WOMAC pain outcome 52](#_Toc200707277)

[eTable K2 Descriptive table for the transitivity assessment of potential effect modifiers on WOMAC stiffness outcome 53](#_Toc200707278)

[eTable K3 Descriptive table for the transitivity assessment of potential effect modifiers on WOMAC function outcome 54](#_Toc200707279)

[eTable K4 Descriptive table for the transitivity assessment of potential effect modifiers on VAS outcome 55](#_Toc200707280)

[**Appendix L** 56](#_Toc200707281)

[**Adverse events** 56](#_Toc200707282)

[eTable L Frequency of adverse events across different treatment groups 56](#_Toc200707283)

[**Appendix M** 57](#_Toc200707284)

[**Certainty of evidence** 57](#_Toc200707285)

[eTable M Grading 57](#_Toc200707286)

[**Appendix N** 60](#_Toc200707287)

[**Sensitivity analyses** 60](#_Toc200707288)

[eFigure N1 Sensitivity analysis of follow-up period for WOMAC pain 60](#_Toc200707289)

[eTable N1 Descriptive table for transitivity assessment of sensitivity analysis on follow-up period for WOMAC pain 61](#_Toc200707290)

[eFigure N2 Sensitivity analysis of follow-up period for WOMAC stiffness 62](#_Toc200707291)

[eTable N2 Descriptive table for transitivity assessment of sensitivity analysis on follow-up period for WOMAC stiffness 63](#_Toc200707292)

[eFigure N3 Sensitivity analysis of follow-up period for WOMAC function 64](#_Toc200707293)

[eTable N3 Descriptive table for transitivity assessment of sensitivity analysis on follow-up period for WOMAC function 65](#_Toc200707294)

[eFigure N4 Sensitivity analysis of high risk of bias for WOMAC pain 66](#_Toc200707295)

[eTable N4 Descriptive table for transitivity assessment of sensitivity analysis on high risk of bias for WOMAC pain 67](#_Toc200707296)

[eFigure N5 Sensitivity analysis of high risk of bias for WOMAC stiffness 68](#_Toc200707297)

[eTable N5 Descriptive table for transitivity assessment of sensitivity analysis on high risk of bias for WOMAC stiffness 69](#_Toc200707298)

[eFigure N6 Sensitivity analysis of high risk of bias for WOMAC function 70](#_Toc200707299)

[eTable N6 Descriptive table for transitivity assessment of sensitivity analysis on high risk of bias for WOMAC function 71](#_Toc200707300)

[eFigure N7 Sensitivity analysis of high risk of bias for VAS 72](#_Toc200707301)

[eTable N7 Descriptive table for transitivity assessment of sensitivity analysis on high risk of bias for VAS 73](#_Toc200707302)

[eFigure N8 Sensitivity analysis of small-study effects for WOMAC pain 74](#_Toc200707303)

[eTable N8 Descriptive table for transitivity assessment of sensitivity analysis on small-study effects for WOMAC pain 75](#_Toc200707304)

[eFigure N9 Sensitivity analysis of small-study effects for WOMAC stiffness 76](#_Toc200707305)

[eTable N9 Descriptive table for transitivity assessment of sensitivity analysis on small-study effects for WOMAC stiffness 77](#_Toc200707306)

[eFigure N10 Sensitivity analysis of small-study effects for WOMAC function 78](#_Toc200707307)

[eTable N10 Descriptive table for transitivity assessment of sensitivity analysis on small-study effects for WOMAC function 79](#_Toc200707308)

[eFigure N11 Sensitivity analysis of small-study effects for VAS 80](#_Toc200707309)

[eTable N11 Descriptive table for transitivity assessment of sensitivity analysis on small-study effects for VAS 81](#_Toc200707310)

[eFigure N12 Sensitivity analysis of baseline intensity for WOMAC pain 82](#_Toc200707311)

[eFigure N13 Sensitivity analysis of baseline intensity for WOMAC stiffness 83](#_Toc200707312)

[eFigure N14 Sensitivity analysis of baseline intensity for WOMAC function 84](#_Toc200707313)

[eFigure N15 Sensitivity analysis of baseline intensity for VAS 85](#_Toc200707314)

[eFigure N16 Adjusted funnel plot of small-study effects for WOMAC pain 86](#_Toc200707315)

[eFigure N17 Adjusted funnel plot of small-study effects for WOMAC stiffness 87](#_Toc200707316)

[eFigure N18 Adjusted funnel plot of small-study effects for WOMAC function 88](#_Toc200707317)

[eFigure N19 Adjusted funnel plot of small-study effects for VAS 89](#_Toc200707318)

[**References** 90](#_Toc200707319)

# **Appendix A**

## **PRISMA NMA checklist**

### eTable A1 PRISMA NMA Checklist of Items to Include When Reporting A Systematic Review Involving a Network Meta-analysis

| **Section/Topic** | **Item #** | **Checklist Item** | Reported on Page # |
| --- | --- | --- | --- |
| **TITLE** |  |  |  |
| Title | 1 | Identify the report as a systematic review *incorporating a network meta-analysis (or related form of meta-analysis).* | 1 |
|  |  |  |  |
| **ABSTRACT** |  |  |  |
| Structured summary | 2 | Provide a structured summary including, as applicable:  **Background:** main objectives  **Methods:** data sources; study eligibility criteria, participants, and interventions; study appraisal; and *synthesis methods, such as network meta-analysis.*  **Results:** number of studies and participants identified; summary estimates with corresponding confidence/credible intervals; *treatment rankings may also be discussed. Authors may choose to summarize pairwise comparisons against a chosen treatment included in their analyses for brevity.*  **Discussion/Conclusions:** limitations; conclusions and implications of findings.  **Other:** primary source of funding; systematic review registration number with registry name. | 2 |
|  |  |  |  |
| **INTRODUCTION** |  |  |  |
| Rationale | 3 | Describe the rationale for the review in the context of what is already known*, including mention of why a network meta-analysis has been conducted.* | 4-5 |
| Objectives | 4 | Provide an explicit statement of questions being addressed, with reference to participants, interventions, comparisons, outcomes, and study design (PICOS). | 5 |
|  |  |  |  |
| **METHODS** |  |  |  |
| Protocol and registration | 5 | Indicate whether a review protocol exists and if and where it can be accessed (e.g., Web address); and, if available, provide registration information, including registration number. | 2,5 |
| Eligibility criteria | 6 | Specify study characteristics (e.g., PICOS, length of follow-up) and report characteristics (e.g., years considered, language, publication status) used as criteria for eligibility, giving rationale. *Clearly describe eligible treatments included in the treatment network, and note whether any have been clustered or merged into the same node (with justification).* | 6-7 |
| Information sources | 7 | Describe all information sources (e.g., databases with dates of coverage, contact with study authors to identify additional studies) in the search and date last searched. | 5 |
| Search | 8 | Present full electronic search strategy for at least one database, including any limits used, such that it could be repeated. | 10 |
| Study selection | 9 | State the process for selecting studies (i.e., screening, eligibility, included in systematic review, and, if applicable, included in the meta-analysis). | 5 |
| Data collection process | 10 | Describe method of data extraction from reports (e.g., piloted forms, independently, in duplicate) and any processes for obtaining and confirming data from investigators. | 6 |
| Data items | 11 | List and define all variables for which data were sought (e.g., PICOS, funding sources) and any assumptions and simplifications made. | 10 |
| **Geometry of the network** | **S1** | Describe methods used to explore the geometry of the treatment network under study and potential biases related to it. This should include how the evidence base has been graphically summarized for presentation, and what characteristics were compiled and used to describe the evidence base to readers. | 8 |
| Risk of bias within individual studies | 12 | Describe methods used for assessing risk of bias of individual studies (including specification of whether this was done at the study or outcome level), and how this information is to be used in any data synthesis. | 6 |
| Summary measures | 13 | State the principal summary measures (e.g., risk ratio, difference in means). *Also describe the use of additional summary measures assessed, such as treatment rankings and surface under the cumulative ranking curve (SUCRA) values, as well as modified approaches used to present summary findings from meta-analyses.* | 9 |
| Planned methods of analysis | 14 | Describe the methods of handling data and combining results of studies for each network meta-analysis. This should include, but not be limited to:   - *Handling of multi-arm trials;* - *Selection of variance structure;* - *Selection of prior distributions in Bayesian analyses; and* - *Assessment of model fit.* | 6,8-9 |
| **Assessment of Inconsistency** | **S2** | Describe the statistical methods used to evaluate the agreement of direct and indirect evidence in the treatment network(s) studied. Describe efforts taken to address its presence when found. | 9 |
| Risk of bias across studies | 15 | Specify any assessment of risk of bias that may affect the cumulative evidence (e.g., publication bias, selective reporting within studies). | 9 |
| Additional analyses | 16 | Describe methods of additional analyses if done, indicating which were pre-specified. This may include, but not be limited to, the following:   - Sensitivity or subgroup analyses; - Meta-regression analyses; - *Alternative formulations of the treatment network; and* - *Use of alternative prior distributions for Bayesian analyses (if applicable).* | 9 |
|  |  |  |  |
| **RESULTS†** |  |  |  |
| Study selection | 17 | Give numbers of studies screened, assessed for eligibility, and included in the review, with reasons for exclusions at each stage, ideally with a flow diagram. | 11 |
| **Presentation of network structure** | **S3** | Provide a network graph of the included studies to enable visualization of the geometry of the treatment network. | 14 |
| **Summary of network geometry** | **S4** | Provide a brief overview of characteristics of the treatment network. This may include commentary on the abundance of trials and randomized patients for the different interventions and pairwise comparisons in the network, gaps of evidence in the treatment network, and potential biases reflected by the network structure. | 11-12 |
| Study characteristics | 18 | For each study, present characteristics for which data were extracted (e.g., study size, PICOS, follow-up period) and provide the citations. | 32-33 |
| Risk of bias within studies | 19 | Present data on risk of bias of each study and, if available, any outcome level assessment. | 12 |
| Results of individual studies | 20 | For all outcomes considered (benefits or harms), present, for each study: 1) simple summary data for each intervention group, and 2) effect estimates and confidence intervals. *Modified approaches may be needed to deal with information from larger networks.* | 12 |
| Synthesis of results | 21 | Present results of each meta-analysis done, including confidence/credible intervals. *In larger networks, authors may focus on comparisons versus a particular comparator (e.g. placebo or standard care), with full findings presented in an appendix. League tables and forest plots may be considered to summarize pairwise comparisons.* If additional summary measures were explored (such as treatment rankings), these should also be presented. | 15 |
| **Exploration for inconsistency** | **S5** | Describe results from investigations of inconsistency. This may include such information as measures of model fit to compare consistency and inconsistency models, *P* values from statistical tests, or summary of inconsistency estimates from different parts of the treatment network. | 12-13 |
| Risk of bias across studies | 22 | Present results of any assessment of risk of bias across studies for the evidence base being studied. | 12 |
| Results of additional analyses | 23 | Give results of additional analyses, if done (e.g., sensitivity or subgroup analyses, meta-regression analyses*, alternative network geometries studied, alternative choice of prior distributions for Bayesian analyses,* and so forth). | 18 |
|  |  |  |  |
| **DISCUSSION** |  |  |  |
| Summary of evidence | 24 | Summarize the main findings, including the strength of evidence for each main outcome; consider their relevance to key groups (e.g., healthcare providers, users, and policy-makers). | 18 |
| Limitations | 25 | Discuss limitations at study and outcome level (e.g., risk of bias), and at review level (e.g., incomplete retrieval of identified research, reporting bias). *Comment on the validity of the assumptions, such as transitivity and consistency. Comment on any concerns regarding network geometry (e.g., avoidance of certain comparisons).* | 20 |
| Conclusions | 26 | Provide a general interpretation of the results in the context of other evidence, and implications for future research. | 21 |
|  |  |  |  |
| **FUNDING** |  |  |  |
| Funding | 27 | Describe sources of funding for the systematic review and other support (e.g., supply of data); role of funders for the systematic review. This should also include information regarding whether funding has been received from manufacturers of treatments in the network and/or whether some of the authors are content experts with professional conflicts of interest that could affect use of treatments in the network. | 23 |

# **Appendix B**

## **Unit standardization**

In standardization outcome scales across diverse trials, established conversion formulas were employed to rescale mean responses and SDs from the original instrument (B) to the units of the more familiar instrument (A). The converted mean estimates were derived by adjusting the original mean and SD using the ranges of the respective instruments [1].

mA = (mB – LB) (RA/RB) + LA; RA = UA – LA; RB = UB – LB

### eTable B1 Different units standardized to Likert units for WOMAC pain

| Author, year | Treatment | n | mB | SDB | UB | LB | UA | LA | RA | RB | mA | SDA |
| --- | --- | --- | --- | --- | --- | --- | --- | --- | --- | --- | --- | --- |
| Singhal, et al. 2021 [2] | BE | 73 | 8.78 | 4.87 | 20 | 0 | 20 | 0 | 20 | 20 | 8.78 | 4.87 |
| Singhal, et al. 2021 [2] | AC | 71 | 7.92 | 4.13 | 20 | 0 | 20 | 0 | 20 | 20 | 7.92 | 4.13 |
| Haroyan, et al. 2018 [3] | BE | 58 | 3.84 | 2.88 | 20 | 0 | 20 | 0 | 20 | 20 | 3.84 | 2.88 |
| Haroyan, et al. 2018 [3] | P | 59 | 5.22 | 3.58 | 20 | 0 | 20 | 0 | 20 | 20 | 5.22 | 3.58 |
| Hashemzadeh, et al. 2020 [4] | BE | 36 | 14.53 | 7.18 | 50 | 5 | 20 | 0 | 20 | 45 | 4.24 | 3.19 |
| Hashemzadeh, et al. 2020 [4] | P | 35 | 21.23 | 7.11 | 50 | 5 | 20 | 0 | 20 | 45 | 7.21 | 3.16 |
| Panda, et al. 2018 [5] | BE | 25 | 4.28 | 1.54 | 20 | 0 | 20 | 0 | 20 | 20 | 4.28 | 1.54 |
| Panda, et al. 2018 [5] | P | 25 | 6.96 | 1.43 | 20 | 0 | 20 | 0 | 20 | 20 | 6.96 | 1.43 |
| Panahi, et al. 2014 [6] | BE | 19 | 6.1 | 2.9 | 20 | 0 | 20 | 0 | 20 | 20 | 6.10 | 2.90 |
| Panahi, et al. 2014 [6] | P | 21 | 9.4 | 3.4 | 20 | 0 | 20 | 0 | 20 | 20 | 9.40 | 3.40 |
| Srivastava, et al. 2016 [7] | CT+AC | 78 | 9.48 | 1.50 | 20 | 0 | 20 | 0 | 20 | 20 | 9.48 | 1.50 |
| Srivastava, et al. 2016 [7] | AC | 82 | 10.16 | 1.45 | 20 | 0 | 20 | 0 | 20 | 20 | 10.16 | 1.45 |
| Kuptniratsaikul, et al. 2014 [8] | CT | 171 | 3.25 | 2.11 | 10 | 0 | 20 | 0 | 20 | 10 | 6.50 | 4.22 |
| Kuptniratsaikul, et al. 2014 [8] | AC | 160 | 3.17 | 1.98 | 10 | 0 | 20 | 0 | 20 | 10 | 6.34 | 3.96 |

Abbreviation: A, the desired instrument (Likert) with the range 0–20 for WOMAC pain; B, the original instrument; LA, lower limit of instrument A; LB, lower limit of instrument B; mA, the standardized mean (Likert); mB, the original mean; RA, range of desired instrument (Likert) 0–20; RB, range of original instrument; SDA, the standardized standard deviation (Likert); SDB, the original standard deviation; UA, upper limit of instrument A; UB, lower limit of instrument B

### eTable B2 Different units standardized to Likert units for WOMAC stiffness

| Author, year | Treatment | n | mB | SDB | UB | LB | UA | LA | RA | RB | mA | SDA |
| --- | --- | --- | --- | --- | --- | --- | --- | --- | --- | --- | --- | --- |
| Singhal, et al. 2021 [2] | BE | 73 | 3.01 | 2.05 | 8 | 0 | 8 | 0 | 8 | 8 | 3.01 | 2.05 |
| Singhal, et al. 2021 [2] | AC | 71 | 3.61 | 1.77 | 8 | 0 | 8 | 0 | 8 | 8 | 3.61 | 1.77 |
| Hashemzadeh, et al. 2020 [4] | BE | 36 | 2.64 | 1.93 | 20 | 2 | 8 | 0 | 8 | 18 | 0.28 | 0.86 |
| Hashemzadeh, et al. 2020 [4] | P | 35 | 2.94 | 2.04 | 20 | 2 | 8 | 0 | 8 | 18 | 0.42 | 0.91 |
| Panda, et al. 2018 [5] | BE | 25 | 2.12 | 0.97 | 8 | 0 | 8 | 0 | 8 | 8 | 2.12 | 0.97 |
| Panda, et al. 2018 [5] | P | 25 | 3.76 | 1.09 | 8 | 0 | 8 | 0 | 8 | 8 | 3.76 | 1.09 |
| Panahi, et al. 2014 [6] | BE | 19 | 0.15 | 0.5 | 8 | 0 | 8 | 0 | 8 | 8 | 0.15 | 0.50 |
| Panahi, et al. 2014 [6] | P | 21 | 0.76 | 0.9 | 8 | 0 | 8 | 0 | 8 | 8 | 0.76 | 0.90 |
| Srivastava, et al. 2016 [7] | CT + AC | 78 | 4.08 | 1.50 | 8 | 0 | 8 | 0 | 8 | 8 | 4.08 | 1.50 |
| Srivastava, et al. 2016 [7] | AC | 82 | 4.16 | 1.63 | 8 | 0 | 8 | 0 | 8 | 8 | 4.16 | 1.63 |
| Kuptniratsaikul, et al. 2014 [8] | CT | 171 | 3.28 | 2.38 | 10 | 0 | 8 | 0 | 8 | 10 | 2.62 | 1.90 |
| Kuptniratsaikul, et al. 2014 [8] | AC | 160 | 3.16 | 2.36 | 10 | 0 | 8 | 0 | 8 | 10 | 2.53 | 1.89 |

Abbreviation: A, the desired instrument (Likert) with the range 0–8 for WOMAC stiffness; B, the original instrument; LA, lower limit of instrument A; LB, lower limit of instrument B; mA, the standardized mean (Likert); mB, the original mean; RA, range of desired instrument (Likert) 0–8; RB, range of original instrument; SDA, the standardized standard deviation (Likert); SDB, the original standard deviation; UA, upper limit of instrument A; UB, lower limit of instrument B

### eTable B3 Different units standardized to Likert units for WOMAC function

| Author, year | Treatment | n | mB | SDB | UB | LB | UA | LA | RA | RB | mA | SDA |
| --- | --- | --- | --- | --- | --- | --- | --- | --- | --- | --- | --- | --- |
| Singhal, et al. 2021 [2] | BE | 73 | 31.22 | 16.23 | 68 | 0 | 68 | 0 | 68 | 68 | 31.22 | 16.23 |
| Singhal, et al. 2021 [2] | AC | 71 | 26.41 | 13.82 | 68 | 0 | 68 | 0 | 68 | 68 | 26.41 | 13.82 |
| Hashemzadeh, et al. 2020 [4] | BE | 36 | 42.61 | 16.7 | 170 | 17 | 68 | 0 | 68 | 153 | 11.38 | 7.42 |
| Hashemzadeh, et al. 2020 [4] | P | 35 | 69.51 | 27.56 | 170 | 17 | 68 | 0 | 68 | 153 | 23.34 | 12.25 |
| Panda, et al. 2018 [5] | BE | 25 | 12.04 | 3.12 | 68 | 0 | 68 | 0 | 68 | 68 | 12.04 | 3.12 |
| Panda, et al. 2018 [5] | P | 25 | 20.04 | 3.77 | 68 | 0 | 68 | 0 | 68 | 68 | 20.04 | 3.77 |
| Panahi, et al. 2014 [6] | BE | 19 | 18.7 | 10.3 | 68 | 0 | 68 | 0 | 68 | 68 | 18.70 | 10.30 |
| Panahi, et al. 2014 [6] | P | 21 | 30.4 | 9.4 | 68 | 0 | 68 | 0 | 68 | 68 | 30.40 | 9.40 |
| Srivastava, et al. 2016 [7] | CT + AC | 78 | 32.14 | 3.53 | 68 | 0 | 68 | 0 | 68 | 68 | 32.14 | 3.53 |
| Srivastava, et al. 2016 [7] | AC | 82 | 33.88 | 4.53 | 68 | 0 | 68 | 0 | 68 | 68 | 33.88 | 4.53 |
| Kuptniratsaikul, et al. 2014 [8] | CT | 171 | 3.41 | 2.09 | 10 | 0 | 68 | 0 | 68 | 10 | 23.19 | 14.21 |
| Kuptniratsaikul, et al. 2014 [8] | AC | 160 | 3.26 | 2.05 | 10 | 0 | 68 | 0 | 68 | 10 | 22.17 | 13.94 |

Abbreviation: A, the desired instrument (Likert) with the range 0–68 for WOMAC function; B, the original instrument; LA, lower limit of instrument A; LB, lower limit of instrument B; mA, the standardized mean (Likert); mB, the original mean; RA, range of desired instrument (Likert) 0–68; RB, range of original instrument; SDA, the standardized standard deviation (Likert); SDB, the original standard deviation; UA, upper limit of instrument A; UB, lower limit of instrument B

### eTable B4 Different units standardized to 0–100 units for VAS

| Author, year | Treatment | n | mB | SDB | UB | LB | UA | LA | RA | RB | mA | SDA |
| --- | --- | --- | --- | --- | --- | --- | --- | --- | --- | --- | --- | --- |
| Shep, et al. 2019 [9] | BE | 70 | 2.2 | 0.81 | 10 | 0 | 100 | 0 | 100 | 10 | 22 | 8.10 |
| Shep, et al. 2019 [9] | AC | 69 | 2.2 | 0.61 | 10 | 0 | 100 | 0 | 100 | 10 | 22 | 6.10 |
| Atabaki, et al. 2020 [10] | BE + AC | 15 | 3.4 | 1.05 | 10 | 0 | 100 | 0 | 100 | 10 | 34 | 10.46 |
| Atabaki, et al. 2020 [10] | AC | 15 | 9.1 | 0.97 | 10 | 0 | 100 | 0 | 100 | 10 | 91 | 9.68 |
| Panda, et al. 2018 [5] | BE | 25 | 27.26 | 11.95 | 100 | 0 | 100 | 0 | 100 | 100 | 27.26 | 11.95 |
| Panda, et al. 2018 [5] | P | 25 | 44.83 | 4.27 | 100 | 0 | 100 | 0 | 100 | 100 | 44.83 | 4.27 |
| Panahi, et al. 2014 [6] | BE | 19 | 37 | 17 | 100 | 0 | 100 | 0 | 100 | 100 | 37 | 17.00 |
| Panahi, et al. 2014 [6] | P | 21 | 57 | 14 | 100 | 0 | 100 | 0 | 100 | 100 | 57 | 14.00 |
| ^*^Henrotin, et al. 2019 [11] | BE | 86 | 37.43 | 23.95 | 100 | 0 | 100 | 0 | 100 | 100 | 37.43 | 23.95 |
| Henrotin, et al. 2019 [11] | P | 40 | 47.95 | 27.29 | 100 | 0 | 100 | 0 | 100 | 100 | 47.95 | 27.29 |
| Srivastava, et al. 2016 [7] | CT + AC | 78 | 4.03 | 0.71 | 10 | 0 | 100 | 0 | 100 | 10 | 40.3 | 7.07 |
| Srivastava, et al. 2016 [7] | AC | 82 | 5.11 | 1.27 | 10 | 0 | 100 | 0 | 100 | 10 | 51.1 | 12.68 |
| Madhu, et al. 2013 [12] | PLS | 29 | 19.48 | 17.84 | 100 | 0 | 100 | 0 | 100 | 100 | 19.48 | 17.84 |
| Madhu, et al. 2013 [12] | P | 29 | 46.03 | 20.84 | 100 | 0 | 100 | 0 | 100 | 100 | 46.03 | 20.84 |

Abbreviation: A, the desired instrument with the range 0–100 for VAS; B, the original instrument; LA, lower limit of instrument A; LB, lower limit of instrument B; mA, the standardized mean; mB, the original mean; RA, range of desired instrument 0–100; RB, range of original instrument; SDA, the standardized standard deviation; SDB, the original standard deviation; UA, upper limit of instrument A; UB, lower limit of instrument B

^*^Means and standard deviations of two intervention groups combined into a single intervention group

# **Appendix C**

## **Search algorithms**

The PICO format was used during the searching step.

### eTable C1 PICO domains and search terms

| **Domain** | **Search terms** |
| --- | --- |
| P | Knee osteoarthritis |
| I | Any turmeric preparations |
| C | Active control (Standard pharmacological treatment)  Placebo |
| O | Pain reduction  Stiffness  Function  Adverse events |

### eTable C2 Search algorithms

| **Database** | **Step** | **Search algorithm** | **Items found** |
| --- | --- | --- | --- |
| **PubMed** | #1 | (osteoarthritis) OR (OA) | 137,383 |
|  | #2 | ((((turmeric) OR (curcumin)) OR (curcuma)) OR (turmer*)) OR (curcum*) | 26,004 |
|  | #3 | (Degenerative arthritis) OR (Degenerative joint disease) | 120,395 |
|  | #4 | ((osteoarthritis) OR (OA)) OR ((Degenerative arthritis) OR (Degenerative joint disease)) | 142,894 |
|  | #5 | (((((turmeric) OR (curcumin)) OR (curcuma)) OR (turmer*)) OR (curcum*)) AND (((osteoarthritis) OR (OA)) OR ((Degenerative arthritis) OR (Degenerative joint disease))) | 332 |
|  | #6 | (((((turmeric) OR (curcumin)) OR (curcuma)) OR (turmer*)) OR (curcum*)) AND (((osteoarthritis) OR (OA)) OR ((Degenerative arthritis) OR (Degenerative joint disease))) Filters: Clinical Trial | **48** |
|  |  | New records found after updating search from Oct 2023 to Aug 2024 | **2** |
| **Embase** | #1 | turmeric OR curcumin OR (curcuma AND longa) OR (curcuma AND longa AND extract) OR (turmeric AND oil) OR turmerin | 44,101 |
|  | #2 | 'osteoarthritis'/exp OR osteoarthritis OR oa OR 'degenerative joint disease' OR 'degenerative arthritis' | 233,235 |
|  | #3 | #1 AND #2 | 815 |
|  | #4 | #1 AND #2 AND [randomized controlled trial]/lim | **71** |
|  |  | New records found after updating search from Oct 2023 to Aug 2024 | **4** |
| **Scopus** | #1 | ( ALL ( osteoarthritis ) OR ALL ( OA ) OR ALL ( degenerative AND arthritis ) OR ALL ( degenerative AND joint AND disease ) ) | 715,386 |
|  | #2 | ( ALL ( turmeric ) OR ALL ( curcumin ) OR ALL ( curcuma ) OR ALL ( turmer* ) OR ALL ( curcum* ) ) | 293,810 |
|  | #3 | ( ( ALL ( osteoarthritis ) OR ALL ( oa ) OR ALL ( degenerative AND arthritis ) OR ALL ( degenerative AND joint AND disease ) ) ) AND ( ( ALL ( turmeric ) OR ALL ( curcumin ) OR ALL ( curcuma ) OR ALL ( turmer* ) OR ALL ( curcum* ) ) ) | 14,747 |

eTable C2 Search algorithms (cont.)

| **Database** | **Step** | **Search algorithm** | **Items found** |
| --- | --- | --- | --- |
|  | #4 | ( ( ALL ( osteoarthritis ) OR ALL ( oa ) OR ALL ( degenerative AND arthritis ) OR ALL ( degenerative AND joint AND disease ) ) ) AND ( ( ALL ( turmeric ) OR ALL ( curcumin ) OR ALL ( curcuma ) OR ALL ( turmer* ) OR ALL ( curcum* ) ) )  AND ( EXCLUDE ( DOCTYPE , "re" ) OR EXCLUDE ( DOCTYPE , "sh" ) OR EXCLUDE ( DOCTYPE , "ed" ) OR EXCLUDE ( DOCTYPE , "no" ) OR EXCLUDE ( DOCTYPE , "le" ) OR EXCLUDE ( DOCTYPE , "tb" ) OR EXCLUDE ( DOCTYPE , "er" ) OR EXCLUDE ( DOCTYPE , "dp" ) ) AND ( EXCLUDE ( EXACTKEYWORD , "nonhuman" ) OR EXCLUDE ( EXACTKEYWORD , "animals" ) OR EXCLUDE ( EXACTKEYWORD , "animal" ) OR LIMIT-TO ( EXACTKEYWORD , "osteoarthritis" ) ) | **477** |
|  |  | New records found after updating search from Oct 2023 to Aug 2024 | **84** |
| **ClinicalTrials.gov** | #1 | Condition/disease: Osteoarthritis; Other terms: OA OR Degenerative Arthritis OR Degenerative Joint Disease; Intervention/treatment: Turmeric OR Curcumin OR Curcuminoids OR Curcuma longa OR Curcuma domestica extracts | **9** |
|  |  | New records found after updating search from Oct 2023 to Aug 2024 | **7** |

# **Appendix D**

## **Additional characteristics of all included studies**

### eTable D1 Regimen characteristics of included studies

| **Author, year** | **Location** | **Intervention used** | **Group** | **Dose/capsule (mg)** | **Dose/day (mg)** | **Regimen** | **Add-on dose/day (mg)** | **Add-on name** |
| --- | --- | --- | --- | --- | --- | --- | --- | --- |
| Shep, et al. 2019 [9] | India | BCM-95® | BE | 500 | 1,500 | 1 Capsule TID |  |  |
|  |  | Diclofenac | AC | 50 | 100 | 1 Tablet BID |  |  |
| Singhal, et al. 2021 [2] | India | BCM-95® | BE | 500 | 1,000 | 1 Capsule BID |  |  |
|  |  | Paracetamol | AC | 650 | 1,950 | 1 Capsule TID |  |  |
| Haroyan, et al. 2018 [3] | Armenia | CuraMed® (BCM-95) | BE | 500 | 1,500 | 1 Capsule TID |  |  |
|  |  | Placebo | P | 500 | 1,500 | 1 Capsule TID |  |  |
| Atabaki, et al. 2020 [10] | Iran | Sinacurcumin® + Diclofenac | BE + AC | 80 | 80 | 1 Capsule OD | 50 | Diclofenac |
|  |  | Placebo + Diclofenac | P + AC | - | - | - | 50 | Diclofenac |

eTable D1 Regimen characteristics of included studies (cont.)

| **Author, year** | **Location** | **Intervention used** | **Group** | **Dose/capsule (mg)** | **Dose/day (mg)** | **Regimen** | **Add-on dose/day (mg)** | **Add-on name** |
| --- | --- | --- | --- | --- | --- | --- | --- | --- |
| Hashemzadeh, et al. 2020 [4] | Iran | SinaCurcumin™ | BE | 40 | 80 | 1 Capsules BID |  |  |
|  |  | Placebo | P | - | - | - |  |  |
| Lopresti, et al. 2021 [13] | Australia | Curcugen® | BE | 500 | 1,000 | 1 capsule BID |  |  |
|  |  | Placebo | P | - | - | 1 capsule BID |  |  |
| Nakagawa, et al. 2014 [14] | Japan | Theracurmin® | BE | 30 | 180 | 3 capsules BID |  |  |
|  |  | Placebo | P | - | - | 3 capsules BID |  |  |
| Panda, et al. 2018 [5] | India | Curene® | BE | 500 | 500 | 1 Capsule OD |  |  |
|  |  | Placebo | P | - | - | 1 Capsule OD |  |  |
| Panahi, et al. 2014 [6] | Iran | C3 complex® | BE | 505 | 1,515 | 1 Capsule TID |  |  |
|  |  | Placebo | P | - | - | - |  |  |

eTable D1 Regimen characteristics of included studies (cont.)

| **Author, year** | **Location** | **Intervention used** | **Group** | **Dose/capsule (mg)** | **Dose/day (mg)** | **Regimen** | **Add-on dose/day (mg)** | **Add-on name** |
| --- | --- | --- | --- | --- | --- | --- | --- | --- |
| Gupte, et al. 2019 [15] | India | Longvida® | BE | 400 | 800 | 1 Capsule BID |  |  |
|  |  | Ibuprofen | AC | 400 | 400 | 1 Capsule OD |  |  |
| Henrotin, et al. 2019 [11] | Belgium | FLEXOFYTOL® | BE (high) | 46.67 | 280.02 | 2 Capsules TID |  |  |
|  |  | FLEXOFYTOL® + Placebo | BE (low) + P | 46.67 | 186.68 | 2 Capsules BID + 2 Capsule OD |  |  |
|  |  | Placebo | P | - | - | 2 Capsules TID |  |  |
| Pinsornsak and Niempoog 2012 [16] | Thailand | Curcuminoids capsules + Diclofenac | CT + AC | 250 | 1,000 | 2 Capsules BID | 75 | Diclofenac |
|  |  | Placebo + Diclofenac | P + AC | - | - | 2 Capsules BID | 75 | Diclofenac |
| Srivastava, et al. 2016 [7] | India | *C. longa* extract registered as ‘Haridra’ + Diclofenac | CT + AC | 500 | 1,000 | 1 Capsule BID | 100 | Diclofenac |
|  |  | Placebo + Diclofenac | P + AC | - | - | - | 100 | Diclofenac |

eTable D1 Regimen characteristics of included studies (cont.)

| **Author, year** | **Location** | **Intervention used** | **Group** | **Dose/capsule (mg)** | **Dose/day (mg)** | **Regimen** | **Add-on dose/day (mg)** | **Add-on name** |
| --- | --- | --- | --- | --- | --- | --- | --- | --- |
| Kuptniratsaikul, et al. 2009 [17] | Thailand | *C. domestica* extract | CT | 500 | 2,000 | 1 Capsule QID |  |  |
|  |  | Ibuprofen | AC | 400 | 800 | 1 Capsule BID |  |  |
| Kuptniratsaikul, et al. 2014 [8] | Thailand | *C. domestica* extract | CT | 250 | 1,500 | 2 Capsules TID |  |  |
|  |  | Ibuprofen | AC | 200 | 1,200 | 2 Capsules TID |  |  |
| Madhu, et al. 2013 [12] | India | Turmacin | PLS | 500 | 1,000 | 1 Capsule BID |  |  |
|  |  | Placebo | P | 400 | 800 | 1 Capsule BID |  |  |
| Wang, et al. 2020 [18] | Australia | Turmacin Plus | PLS | 500 | 1,000 | 2 Capsules OD |  |  |
|  |  | Placebo | P | - | - | 2 Capsules OD |  |  |

Abbreviation: A, active drug comparator; BE, bioavailability-enhanced curcuminoid preparations; CT, conventional curcuminoid preparations; P, placebo; PLS, polysaccharide preparations

### eTable D2 Baseline and follow-up characteristics of all included studies

| Author, year | Duration of knee OA/pain, months, mean + SD | | Baseline pain intensity measured by a pain measure, mean ± SD | | Follow-up duration (days) |
| --- | --- | --- | --- | --- | --- |
|  | Intervention | Control | Intervention | Control |  |
| Shep, et al. 2019 [9] | 7.4 +3.53 | 7.45 +3.15 | 78.40 +6.3^a^ | 78.10 +7.3^a^ | 28 |
| Singhal, et al. 2021 [2] | NA | NA | 56.30 +20.50^b^ | 50.20 +19.50^b^ | 42^**^ |
| Haroyan, et al. 2018 [3] | NA | NA | 28.94 +13.20^b^ | 33.37 +15.21^b^ | 84^**^ |
| Atabaki, et al. 2020 [10] | 53.52 +30.21^***^ | 56.4 +30.21^***^ | 79.30 +15.10^a^ | 84.60 +19.36^a^ | 90^**^ |
| Hashemzadeh, et al. 2020 [4] | 21.69 +9.94 | 24.8 +8.52 | 33.44 +14.29^b^ | 34.00 +14.66^b^ | 42 |
| Lopresti, et al. 2021 [13] | NA | NA | 6.22 +1.43^c^ | 5.80 +1.56^c^ | 56^**^ |
| Nakagawa, et al. 2014 [14] | NA | NA | 52 +24^a^ | 42 +25^a^ | 56 |
| Panda, et al. 2018 [5] | NA | NA | 52.37 +6.41^a^ | 52.79 +4.47^a^ | 60 |
| Panahi, et al. 2014 [6] | NA | NA | 66.32 +14.22^a^ | 59.05 +17.29^a^ | 42^**^ |

eTable D2 Baseline and follow-up characteristics of all included studies (cont.)

| Author, year | Duration of knee OA/pain, months, mean + SD | | Baseline pain intensity measured by a pain measure, mean ± SD | | Follow-up duration (days) |
| --- | --- | --- | --- | --- | --- |
|  | **Intervention** | **Control** | **Intervention** | **Control** |  |
| Gupte, et al. 2019 [15] | NA | NA | 80^a,d^ | 85^a,d^ | 90 |
| Henrotin, et al. 2019 [11] | 88.92 +97.52^***^, 79.2 +56.052^***^ | 91.2 +111.6^***^ | 62.9 +13.8^a^, 63.3 +15.8^a^ | 59.9 +12.3^a^ | 90 |
| Pinsornsak and Niempoog 2012 [16] | NA | NA | 55 +NA^a^ | 53.1 +NA^a^ | 90^**^ |
| Srivastava, et al. 2016 [7] | NA | NA | 79.40 +11.48^a^ | 76.60 +12.68^a^ | 120 |
| Kuptniratsaikul, et al. 2009 [17] | 19.1 +19.6 | 22.3 +26.4 | 5.3 +2.3^c^ | 5.0 +1.9^c^ | 42^**^ |
| Kuptniratsaikul, et al. 2014 [8] | 51.3 +53.4 | 52 +51.7 | 50.88 +17.28^b^ | 49.92 +16.32^b^ | 28^**^ |

eTable D2 Baseline and follow-up characteristics of all included studies (cont.)

| Author, year | Duration of knee OA/pain, months, mean + SD | | Baseline pain intensity measured by a pain measure, mean ± SD | | Follow-up duration (days) |
| --- | --- | --- | --- | --- | --- |
|  | **Intervention** | **Control** | **Intervention** | **Control** |  |
| Madhu, et al. 2013 [12] | NA | NA | 66.5 +21.06^a^ | 61.5 +13.71^a^ | 42 |
| Wang, et al. 2020 [18] | NA | NA | 55.6 +16.1^a^ | 54.4 +17.8^a^ | 84^**^ |

Abbreviation: AC, active drug comparator; BE, bioavailability-enhanced curcuminoid preparations; CT, conventional curcuminoid preparations; NA, not available; P, placebo; PLS, polysaccharide preparations, ** converted from week, *** converted from year, ^a^ pain measured on a VAS scale (score standardized to 0–100), ^b^ pain measured on a WOMAC scale (total score standardized to 0–96), ^c^ pain measured on an NRS scale (0–10), ^d^ estimated data obtained from graphical presentation

For each outcome of interest except the adverse events, the final mean (the longest reported follow-up) was used. Data were extracted in mean + standard deviation (SD). In instances where data were not presented in SDs, SDs were computed from the reported standard errors (SEs) or confidence intervals (CIs) as provided.

### eTable D3 Extracted data of outcomes of interest

| **Author, year** | **Treatment** | **n** | **Final mean** | **Final SD** |
| --- | --- | --- | --- | --- |
| **WOMAC pain** | | | | |
| Singhal, et al. 2021 [2] | BE | 73 | 8.78 | 4.87^*^ |
|  | AC | 71 | 7.92 | 4.13^*^ |
| Haroyan, et al. 2018 [3] | BE | 58 | 3.84 | 2.88 |
|  | P | 59 | 5.22 | 3.58 |
| Hashemzadeh, et al. 2020 [4] | BE | 36 | 14.53 | 7.18 |
|  | P | 35 | 21.23 | 7.11 |
| Panda, et al. 2018 [5] | BE | 25 | 4.28 | 1.54 |
|  | P | 25 | 6.96 | 1.43 |
| Panahi, et al. 2014 [6] | BE | 19 | 6.1 | 2.9 |
|  | P | 21 | 9.4 | 3.4 |
| Srivastava, et al. 2016 [7] | CT+AC | 78 | 9.48 | 1.50^*^ |
|  | AC | 82 | 10.16 | 1.45^*^ |
| Kuptniratsaikul, et al. 2014 [8] | CT | 171 | 3.25 | 2.11 |
|  | AC | 160 | 3.17 | 1.98 |
| **WOMAC stiffness** | | | | |
| Singhal, et al. 2021 [2] | BE | 73 | 3.01 | 2.05^*^ |
|  | AC | 71 | 3.61 | 1.77^*^ |
| Hashemzadeh, et al. 2020 [4] | BE | 36 | 2.64 | 1.93 |
|  | P | 35 | 2.94 | 2.04 |
| Panda, et al. 2018 [5] | BE | 25 | 2.12 | 0.97 |
|  | P | 25 | 3.76 | 1.09 |
| Panahi, et al. 2014 [6] | BE | 19 | 0.15 | 0.5 |
|  | P | 21 | 0.76 | 0.9 |
| Srivastava, et al. 2016 [7] | CT + AC | 78 | 4.08 | 1.50^*^ |
|  | AC | 82 | 4.16 | 1.63^*^ |
| Kuptniratsaikul, et al. 2014 [8] | CT | 171 | 3.28 | 2.38^*^ |
|  | AC | 160 | 3.16 | 2.36^*^ |

eTable D3 Extracted data of outcomes of interest (cont.)

| **Author, year** | **Treatment** | **n** | **Final mean** | **Final SD** |
| --- | --- | --- | --- | --- |
| **WOMAC function** | | | | |
| Singhal, et al. 2021 [2] | BE | 73 | 31.22 | 16.23^*^ |
|  | AC | 71 | 26.41 | 13.82^*^ |
| Hashemzadeh, et al. 2020 [4] | BE | 36 | 42.61 | 16.7 |
|  | P | 35 | 69.51 | 27.56 |
| Panda, et al. 2018 [5] | BE | 25 | 12.04 | 3.12 |
|  | P | 25 | 20.04 | 3.77 |
| Panahi, et al. 2014 [6] | BE | 19 | 18.7 | 10.3 |
|  | P | 21 | 30.4 | 9.4 |
| Srivastava, et al. 2016 [7] | CT + AC | 78 | 32.14 | 3.53^*^ |
|  | AC | 82 | 33.88 | 4.53^*^ |
| Kuptniratsaikul, et al. 2014 [8] | CT | 171 | 3.41 | 2.09^*^ |
|  | AC | 160 | 3.26 | 2.05^*^ |
| **VAS** | | | | |
| Shep, et al. 2019 [9] | BE | 70 | 2.2 | 0.81 |
|  | AC | 69 | 2.2 | 0.61 |
| Atabaki, et al. 2020 [10] | BE + AC | 15 | 3.4 | 1.05^*^ |
|  | AC | 15 | 9.1 | 0.97^*^ |
| Panda, et al. 2018 [5] | BE | 25 | 27.26 | 11.95 |
|  | P | 25 | 44.83 | 4.27 |
| Panahi, et al. 2014 [6] | BE | 19 | 37^**^ | 17 |
|  | P | 21 | 57^**^ | 14 |
| Henrotin, et al. 2019 [11] | BE | 86 | 37.43 | 23.95 |
|  | P | 40 | 47.95 | 27.29 |
| Srivastava, et al. 2016 [7] | CT + AC | 78 | 4.03 | 0.71^*^ |
|  | AC | 82 | 5.11 | 1.27^*^ |
| Madhu, et al. 2013 [12] | PLS | 29 | 19.48 | 17.84 |
|  | P | 29 | 46.03 | 20.84 |

Abbreviation: AC, active drug comparator; BE, bioavailability-enhanced curcuminoid preparations; CT, conventional curcuminoid preparations; P, placebo; PLS, polysaccharide preparations; SD, standard deviation; ^*^, Converted from standard error; ^**^, estimated data obtained from graphical presentation


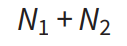
To avoid duplication of control arms in the NMA, the high-dose and low-dose groups from the Henrotin et al. (2019) study were combined into a single intervention group. The combined mean and standard deviation were calculated using the following formulas as stated in the Cochrane Handbook, chapter 6:

Combined sample size =


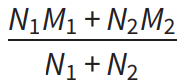


Combined mean =


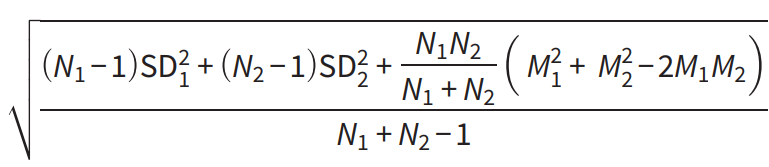


Combined standard deviation =

Where *N_1_* is the sample size of the high-dose arm (48), *M_1_* is the mean of the high-dose arm (37.63), and *SD_1_* is the standard deviation of the high-dose arm (25.63). *N_2_* is the sample size of the low-dose arm (38), *M_2_* is the mean of the low-dose arm (37.18), and *SD_2_* is the standard deviation of the low-dose arm (21.98).

### eTable D4 Detailed characteristics of curcuminoid preparations included in the analysis

| **Author, year** | **Intervention** | **Technique to enhance bioavailability** | **Dose taken per day (mg)** | **Increased absorption (fold)^*^** |
| --- | --- | --- | --- | --- |
| Shep, et al. 2019 [9] | BCM-95® | Addition of turmeric oil | 1,500 | 6.93 |
| Singhal, et al. 2021 [2] |  |  | 1,000 |  |
| Haroyan, et al. 2018 [3] | CuraMed®  (BCM-95) |  | 1,500 |  |
| Panahi, et al. 2014 [6] | C3 complex® | Addition of piperine | 1,515 | 20 |
| Panda, et al. 2018 [5] | Curene® | Formulated with proprietary Aquasome technology – for enhancing bioavailability of curcuminoids | 500 | NA |
| Henrotin, et al. 2019 [11] | FLEXOFYTOL® | Reduction in sample size (emulsifier, polysorbate 80) | 280.02,  186.68 | NA |
| Atabaki, et al. 2020 [10] | Sinacurcumin® | Reduction in particle size (nanomicelle formulation) | 80 | NA |
| Hashemzadeh, et al. 2020 [4] | SinaCurcumin™ |  |  |  |
| Srivastava, et al. 2016 [7] | *C. longa* extract registered as ‘Haridra’ | None | 1,000 | 1 |
| Kuptniratsaikul, et al. 2014 [8] | *C. domestica* extract | None | 1,500 | 1 |

^*^ Data from the literature review [19]

# **Appendix E**

## **Risk of bias assessment**

### eFigure E Risk of bias graph

The review authors’ judgment about each risk of bias item are presented as percentages across all included studies, indicating the distribution of low, some concerns, and high risk of bias.

### eTable E Summarized risk of bias of included studies using ROB2.0

We adhered to the recommended approach for assessing the risk of bias in studies included in Cochrane reviews. This approach encompasses six specific domains, namely the randomization process, intended intervention, missing outcome data, measurement of outcome, reported results, and overall risk of bias. Each domain consists of one or more specific entries in a ‘Risk of Bias’ table. The tool involves assigning a judgment regarding the risk of bias for each entry. This is accomplished by answering a pre-specified question about the adequacy of the study in relation to the entry, resulting in a judgment of low risk of bias, some concerns, or high risk of bias.


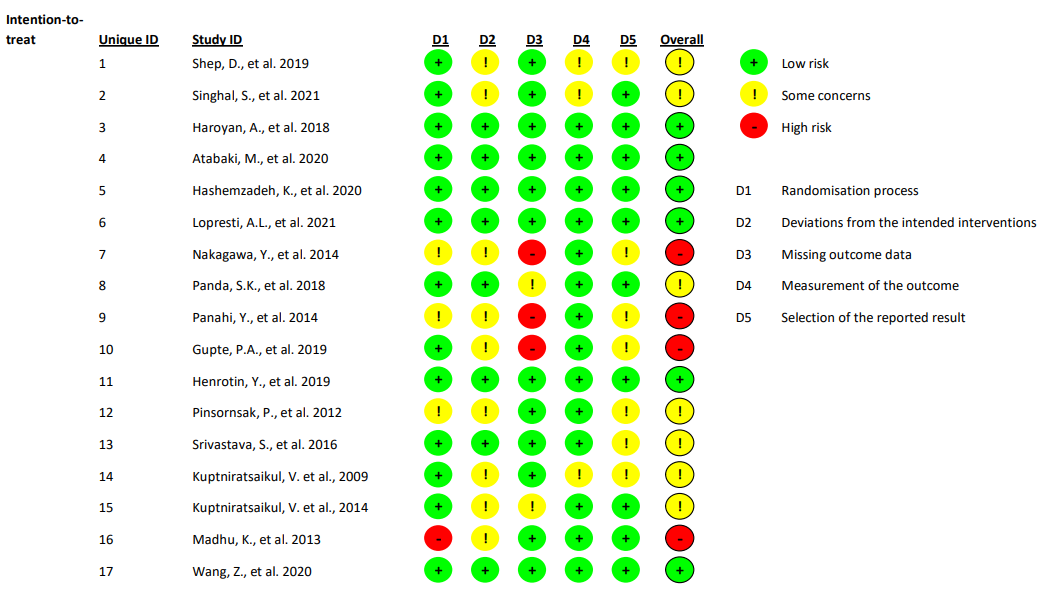


# **Appendix F**

## **Results of meta-analyses of direct comparisons of treatment options**

While eight RCTs reported WOMAC stiffness and WOMAC function outcomes [2-8, 18], Haroyan et al. 2018, and Wang et al. 2020 reported mean change data only, precluding their integration [3, 18]. For WOMAC stiffness and function outcomes, six studies (*N* = 796) were included in the analysis.

### eFigure F1 Mean difference (and 95% CI) for WOMAC pain


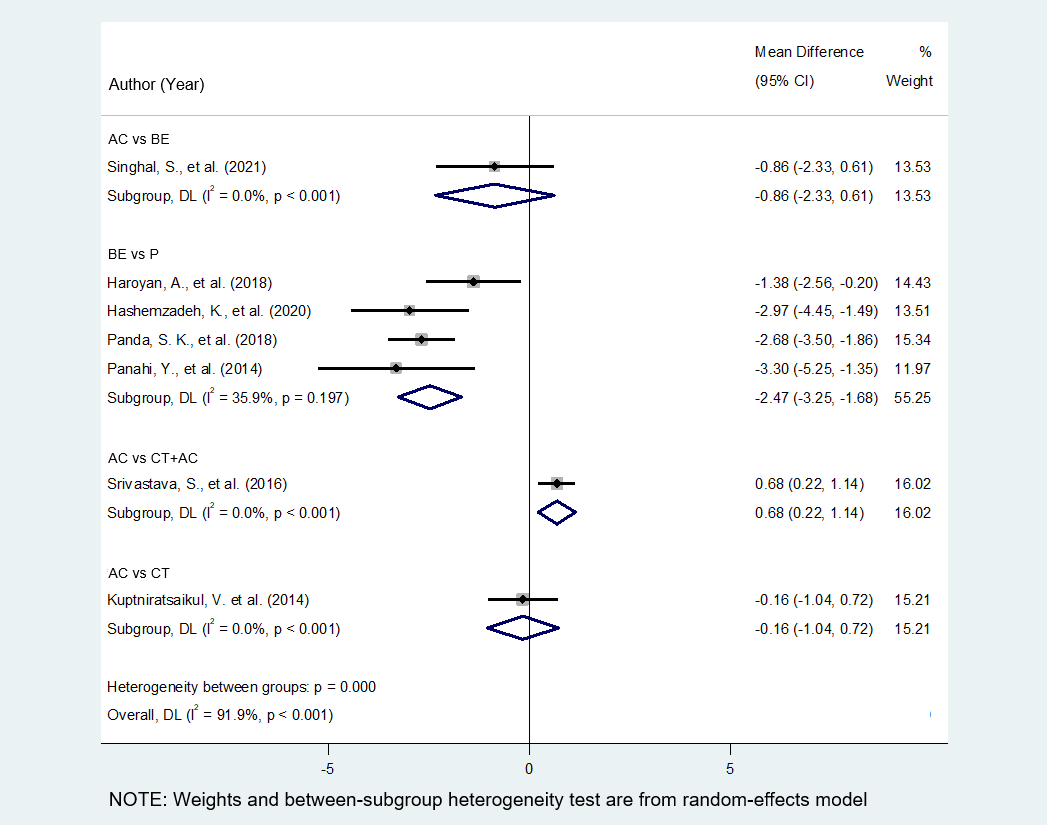


Abbreviation: CI, confidence interval; AC, active drug comparator; BE, bioavailability-enhanced curcuminoid preparations; CT, conventional curcuminoid preparations; CT + AC, conventional curcuminoid preparations + active drug comparator; P, placebo

### eFigure F2 Mean difference (and 95% CI) for WOMAC stiffness


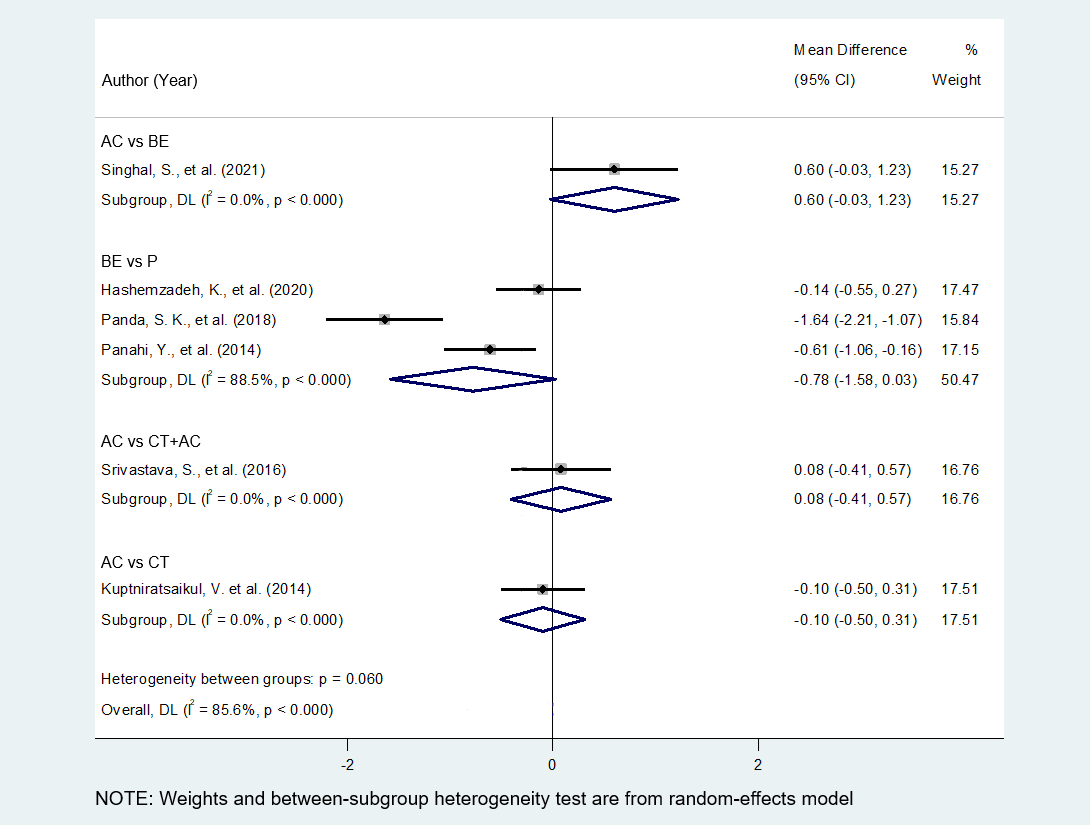


Abbreviation: CI, confidence interval; AC, active drug comparator; BE, bioavailability-enhanced curcuminoid preparations; CT, conventional curcuminoid preparations; CT + AC, conventional curcuminoid preparations + active drug comparator; P, placebo

### eFigure F3 Mean difference (and 95% CI) for WOMAC function


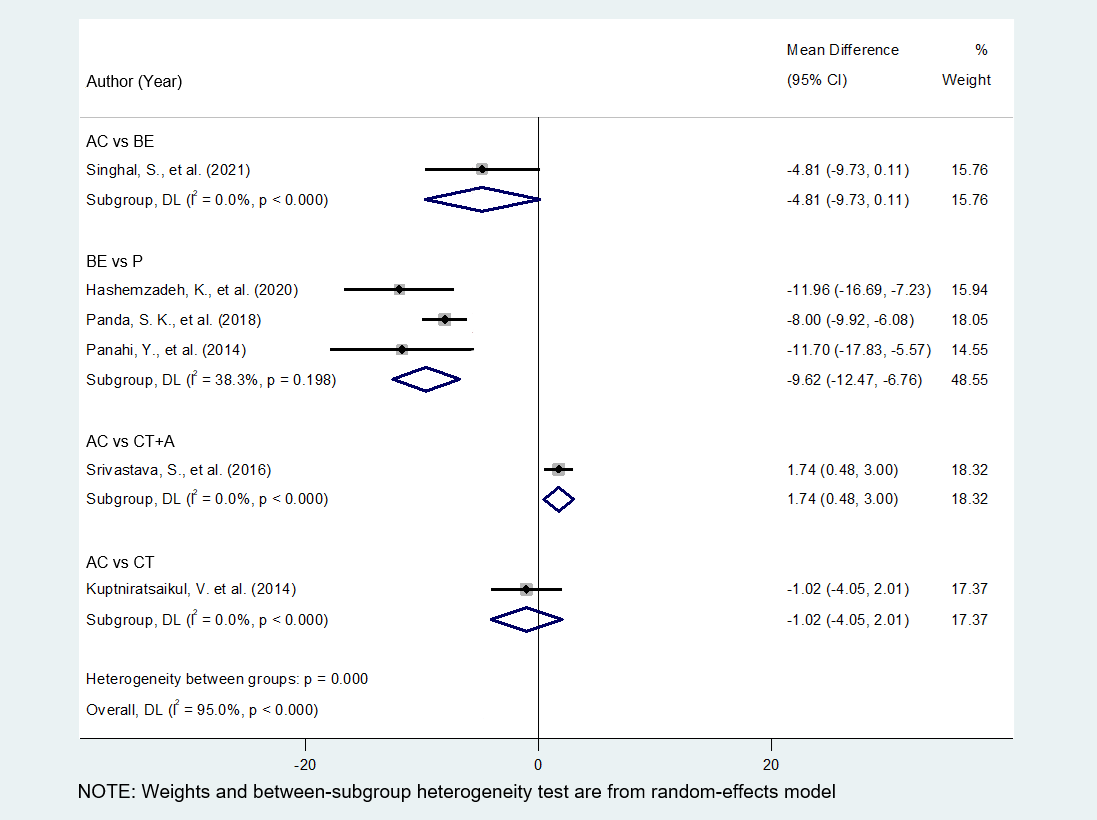


Abbreviation: CI, confidence interval; AC, active drug comparator; BE, bioavailability-enhanced curcuminoid preparations; CT, conventional curcuminoid preparations; CT + AC, conventional curcuminoid preparations + active drug comparator; P, placebo

Eleven RCTs reported VAS outcomes [5-7, 9-12, 14-16, 18]. Nakagawa et al. 2014 and Wang et al. 2020 reported mean change data only, so they were not integrated for analysis [14, 18]. Among the remaining nine RCTs, two studies were excluded: Gupte et al. 2019 reported the data graphically, and Pinsornsak et al. 2012 reported the data in mean only without SD [15, 16]. Finally, seven studies (*N* = 603) were included for VAS pain outcome. Subgroups were formed according to their intervention group and control group: A vs. BE, BE vs. P, A vs. CT + A, P vs. PLS, and A vs. BE + A.

### eFigure F4 Mean difference (and 95% CI) for VAS


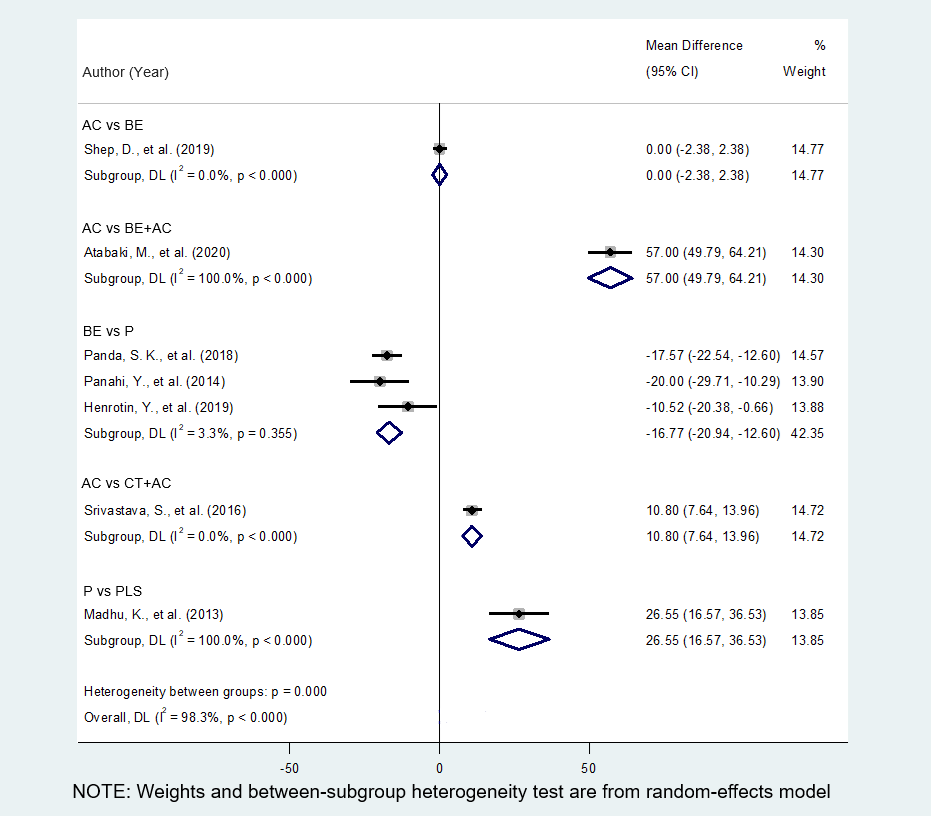
 Abbreviation: CI, confidence interval; AC, active drug comparator; BE, bioavailability-enhanced curcuminoid preparations; BE + AC, bioavailability-enhanced curcuminoid preparations + active drug comparator; CT + AC, conventional curcuminoid preparations + active drug comparator; P, placebo; PLS, polysaccharide preparations

# **Appendix G**

## **Assessment of global inconsistency for each outcome network in main analysis**

### eTable G Assessment of global inconsistency in networks using the ‘design-by-treatment’ interaction model

| **Network outcome** | **Chi-square** | ***p*-value for test of global inconsistency** |
| --- | --- | --- |
| WOMAC pain | 36.59 | 0.0000 |
| WOMAC stiffness | 3.17 | 0.0748 |
| WOMAC function | 30.11 | 0.0000 |
| VAS pain | 27.16 | 0.0000 |

# **Appendix H**

## **Interval plots**

### eFigure H1 Interval plot of WOMAC pain


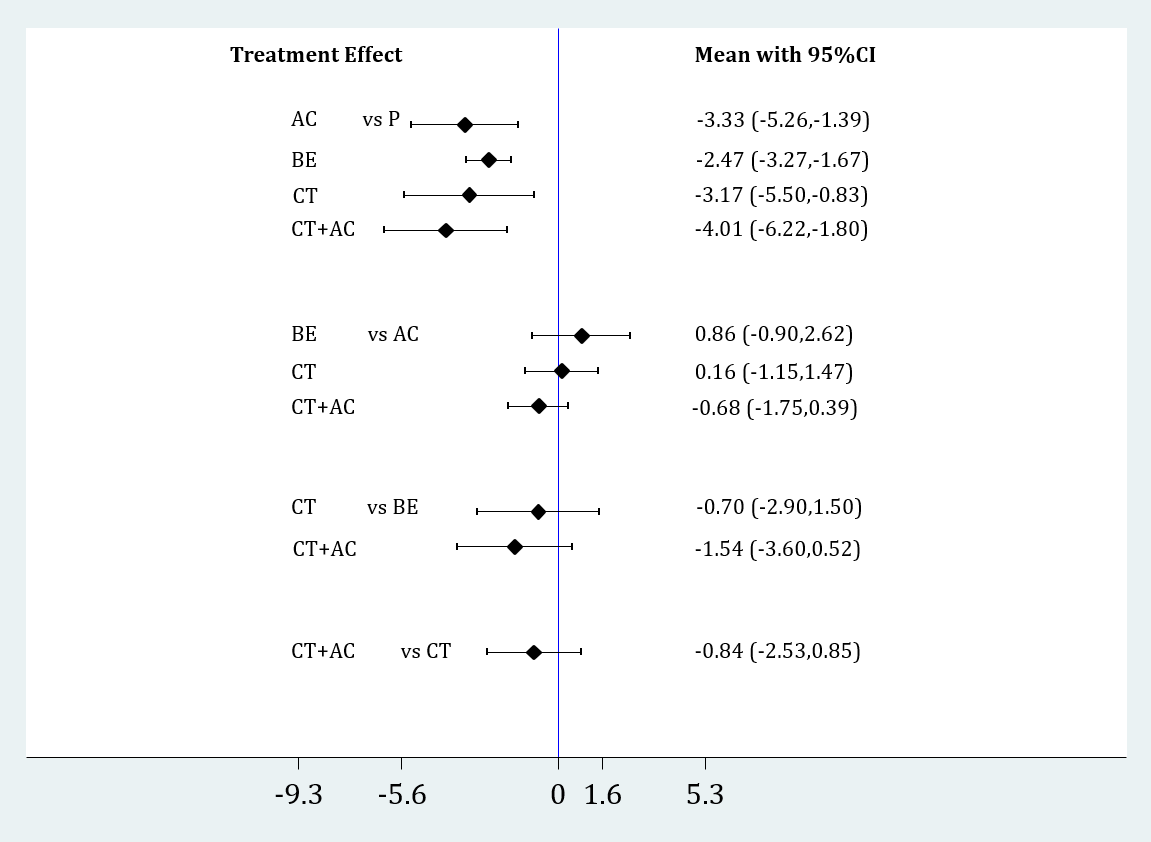
(chi-square for global consistency = 36.59, *p* = 0.0000)

Abbreviation: CI, confidence interval; AC, active drug comparator; BE, bioavailability-enhanced curcuminoid preparations; CT, conventional curcuminoid preparations; CT + AC, conventional curcuminoid preparations + active drug comparator; P, placebo

### eFigure H2 Interval plot of WOMAC stiffness


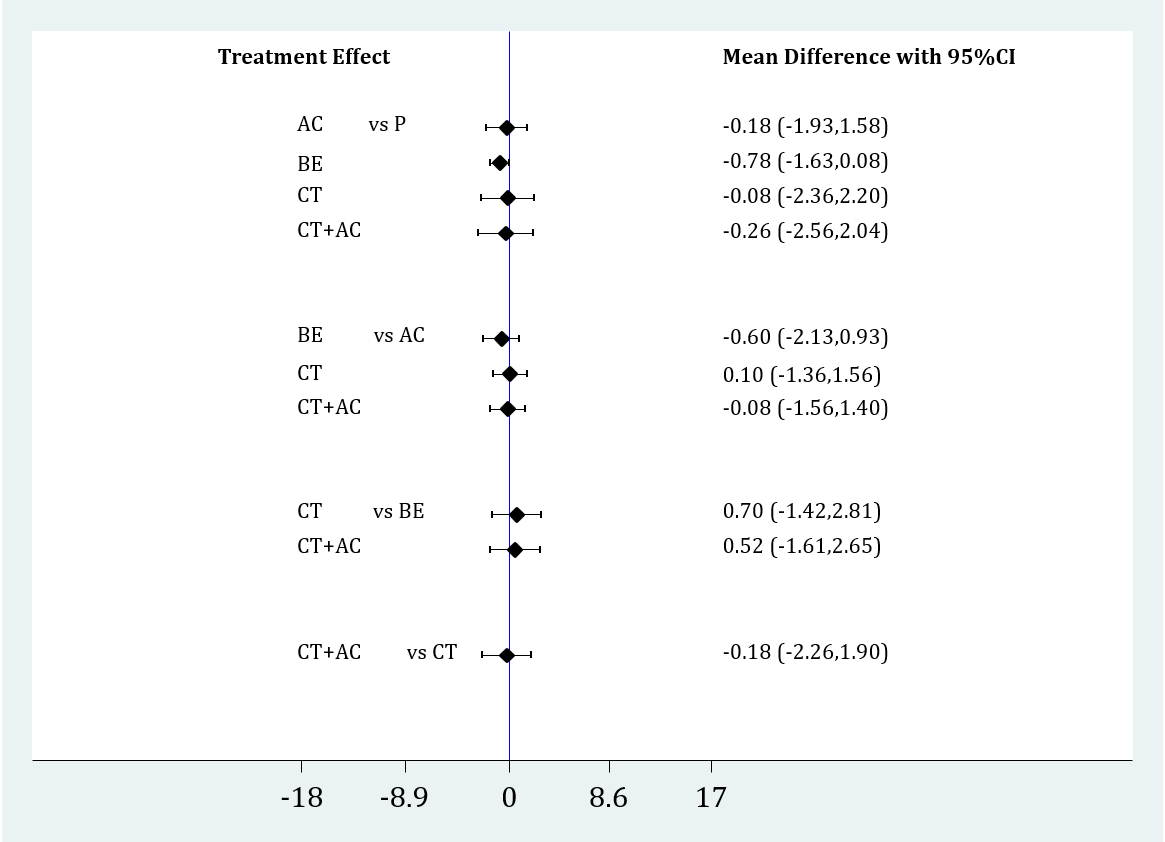


(chi-square for global consistency = 3.17, *p* = 0.0748)

Abbreviation: CI, confidence interval; AC, active drug comparator; BE, bioavailability-enhanced curcuminoid preparations; CT, conventional curcuminoid preparations; CT + AC, conventional curcuminoid preparations + active drug comparator; P, placebo

### eFigure H3 Interval plot of WOMAC function


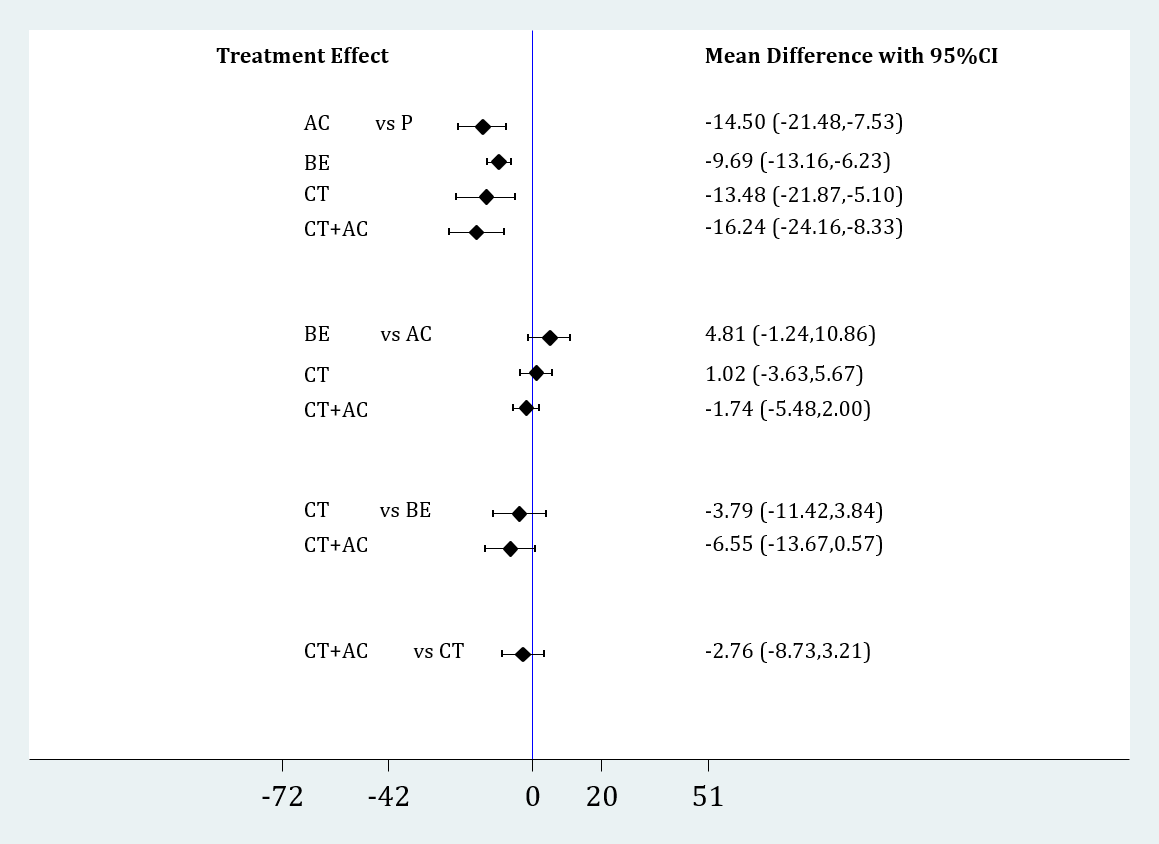


(chi-square for global consistency = 30.11, *p* = 0.0000)

Abbreviation: CI, confidence interval; AC, active drug comparator; BE, bioavailability-enhanced curcuminoid preparations; CT, conventional curcuminoid preparations; CT + AC, conventional curcuminoid preparations + active drug comparator; P, placebo

### eFigure H4 Interval plot of VAS


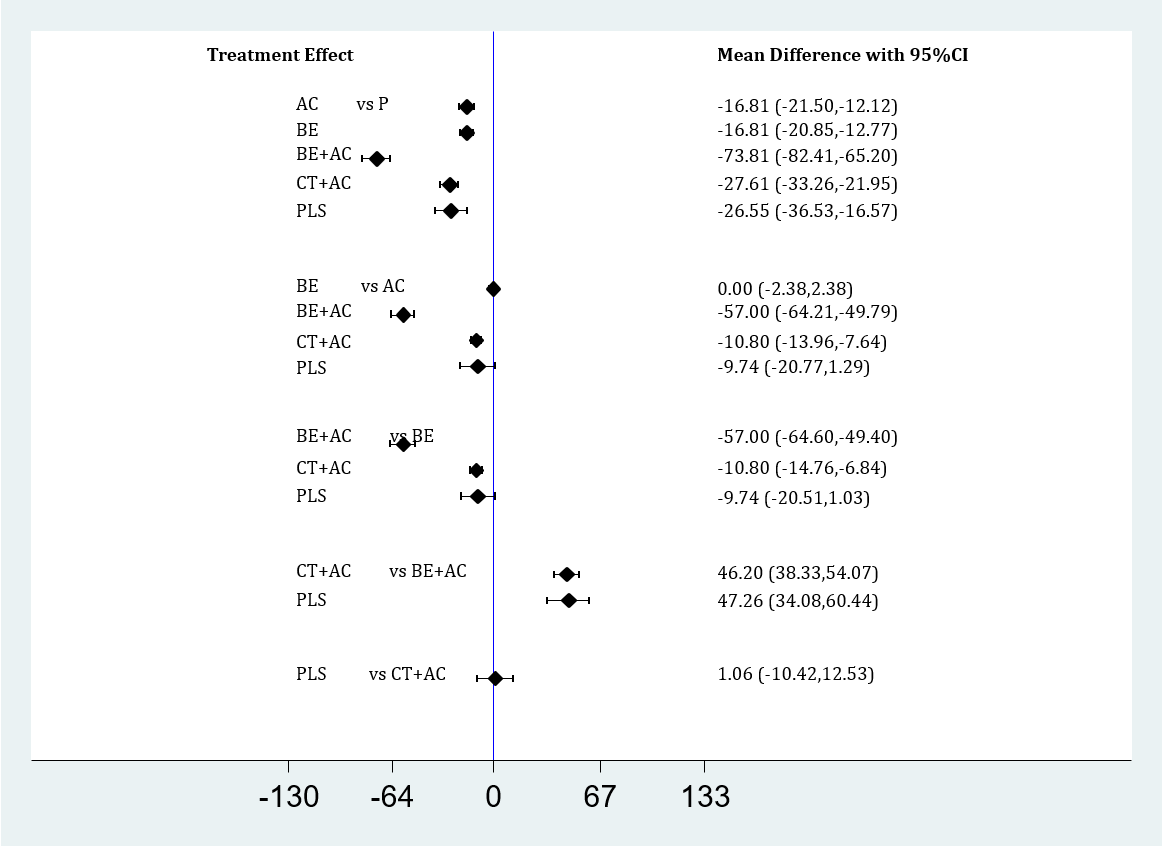


(chi-square for global consistency = 27.16, *p* = 0.0000)

Abbreviation: CI, confidence interval; AC, active drug comparator; BE, bioavailability-enhanced curcuminoid preparations; BE + AC, bioavailability-enhanced curcuminoid preparations + active drug comparator; CT + AC, conventional curcuminoid preparations + active drug comparator; P, placebo; PLS, polysaccharide preparations

# **Appendix I**

## **SUCRA ranking for WOMAC Pain, Stiffness, Function, and VAS**

### eFigure I1 SUCRA ranking among interventions on WOMAC pain


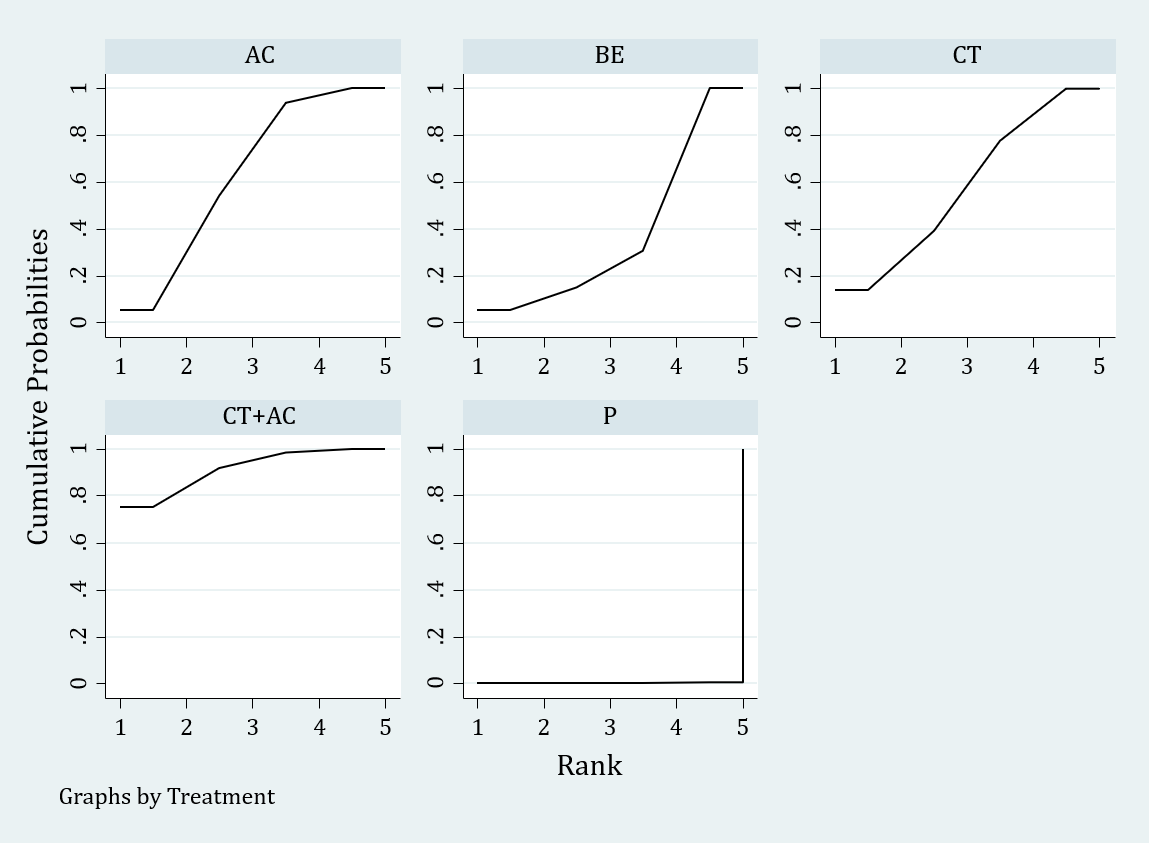


Abbreviation: AC, active drug comparator; BE, bioavailability-enhanced curcuminoid preparations; CT, conventional curcuminoid preparations; CT + AC, conventional curcuminoid preparations + active drug comparator; P, placebo; SUCRA, surface under the cumulative ranking

### eFigure I2 SUCRA ranking among interventions on WOMAC stiffness


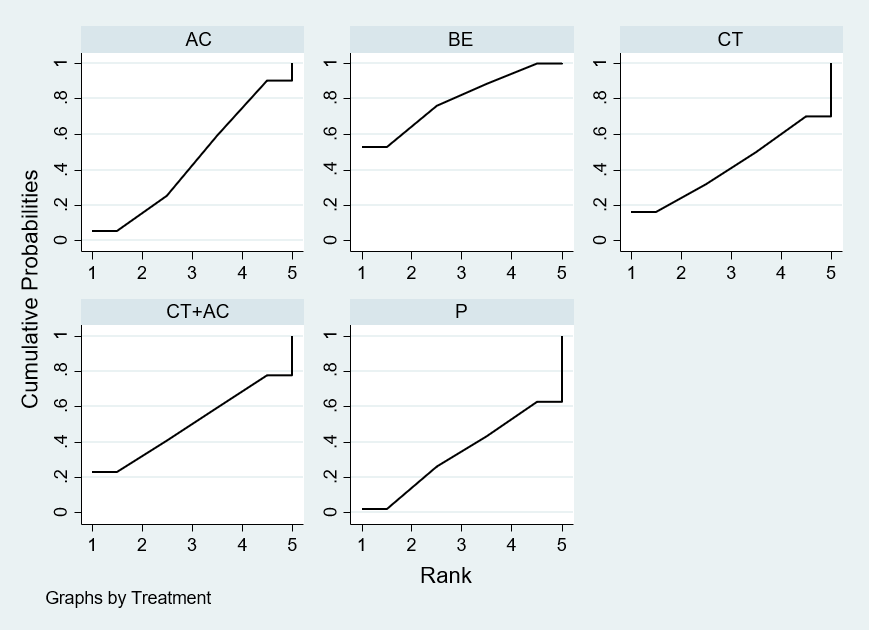


Abbreviation: AC, active drug comparator; BE, bioavailability-enhanced curcuminoid preparations; CT, conventional curcuminoid preparations; CT + AC, conventional curcuminoid preparations + active drug comparator; P, placebo; SUCRA, surface under the cumulative ranking

### eFigure I3 SUCRA ranking among interventions on WOMAC function


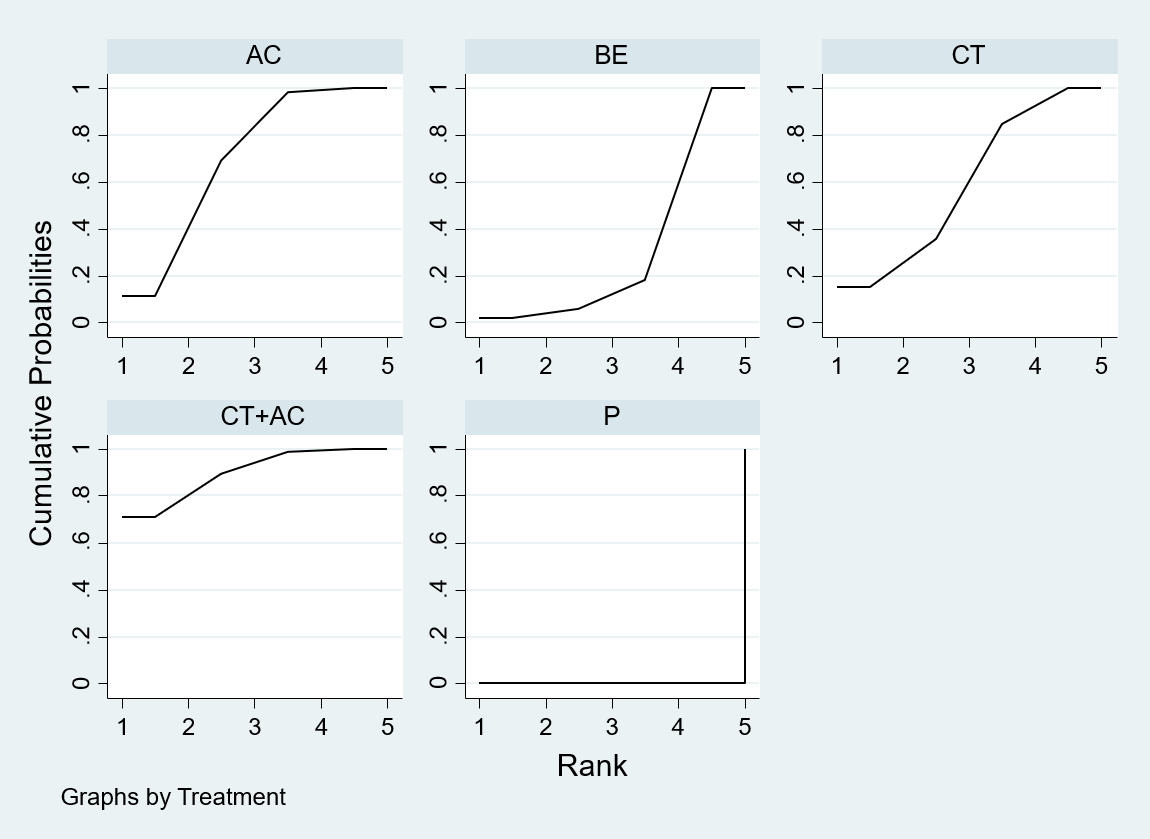


Abbreviation: AC, active drug comparator; BE, bioavailability-enhanced curcuminoid preparations; CT, conventional curcuminoid preparations; CT + AC, conventional curcuminoid preparations + active drug comparator; P, placebo; SUCRA, surface under the cumulative ranking

### eFigure I4 SUCRA ranking among interventions on VAS


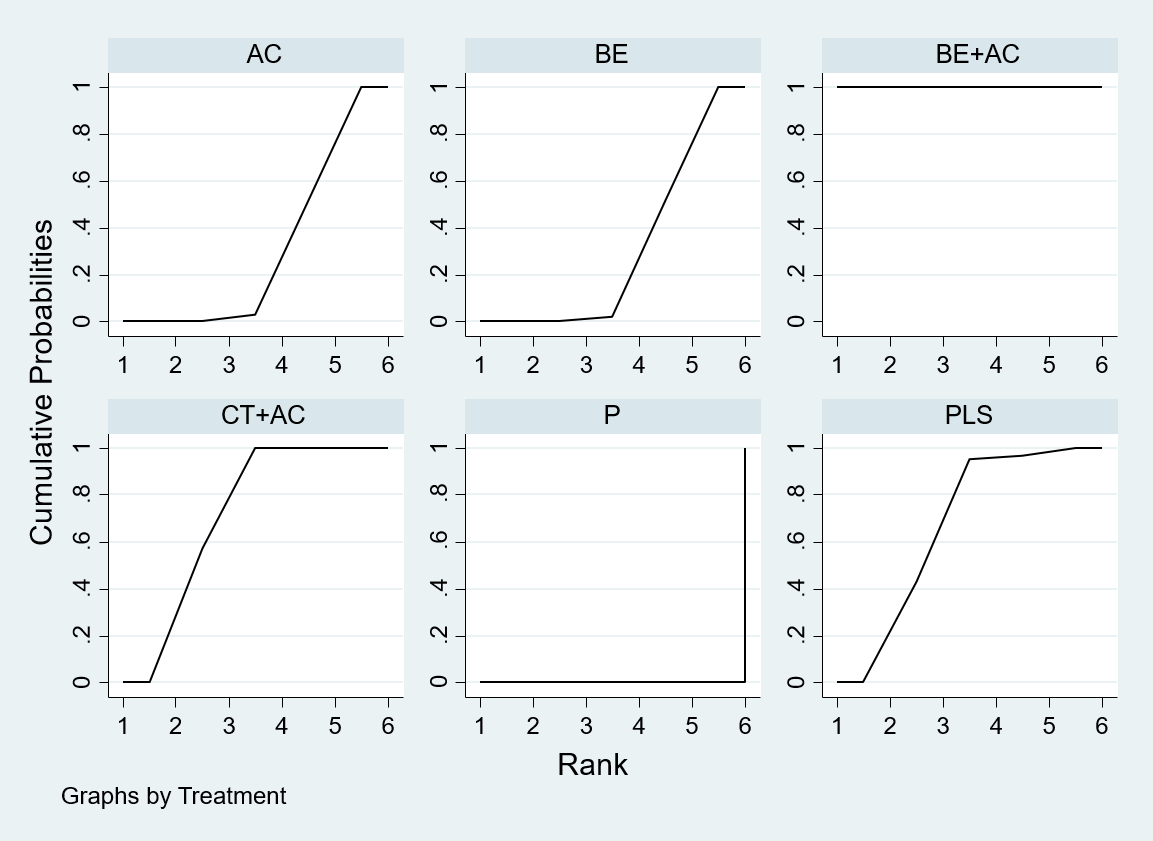


Abbreviation: AC, active drug comparator; BE, bioavailability-enhanced curcuminoid preparations; BE + AC, bioavailability-enhanced curcuminoid preparations + active drug comparator; CT + AC, conventional curcuminoid preparations + active drug comparator; P, placebo; PLS, polysaccharide preparations; SUCRA, surface under the cumulative ranking

# **Appendix J**

## **Adjusted funnel plots**

### eFigure J1 Adjusted funnel plot for WOMAC pain


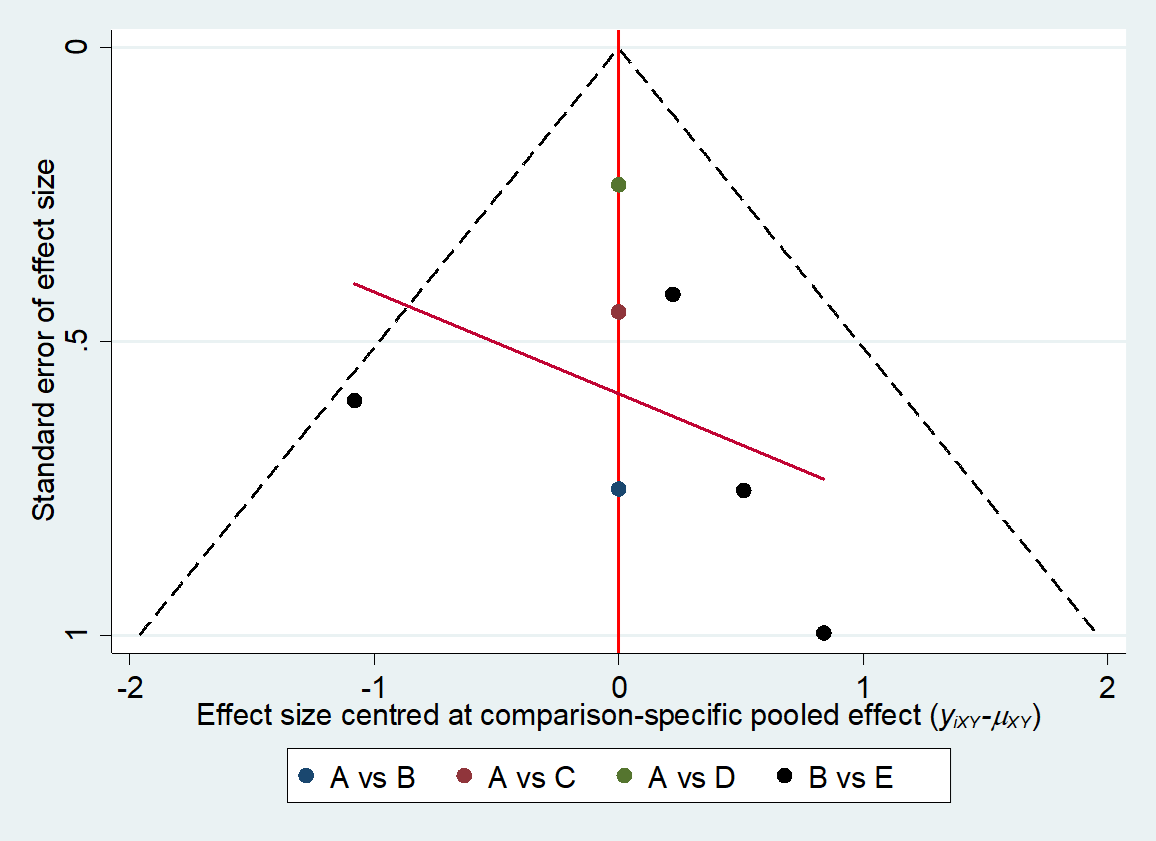


Abbreviation: A = AC, active drug comparator; B = BE, bioavailability-enhanced curcuminoid preparations; C = CT, conventional curcuminoid preparations; D = CT + AC, conventional curcuminoid preparations + active drug comparator; E = P, placebo

### eFigure J2 Adjusted funnel plot for WOMAC stiffness


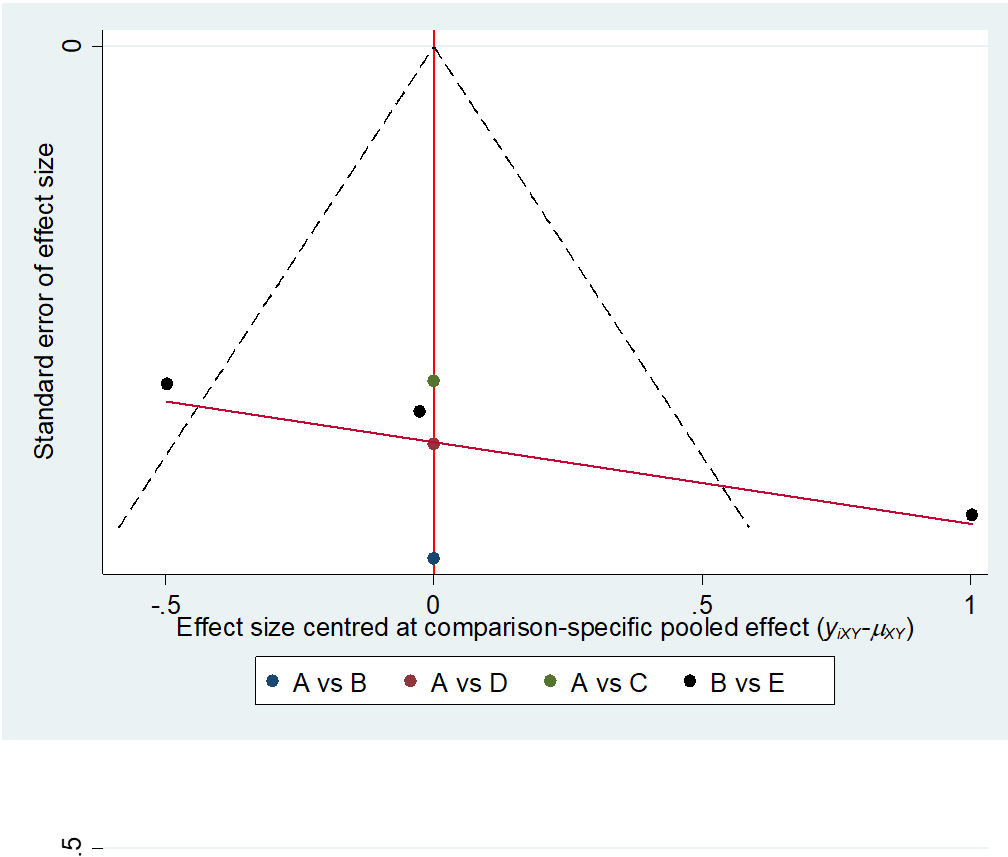


Abbreviation: A = AC, active drug comparator; B = BE, bioavailability-enhanced curcuminoid preparations; C = CT, conventional curcuminoid preparations; D = CT + AC, conventional curcuminoid preparations + active drug comparator; E = P, placebo

### eFigure J3 Adjusted funnel plot for WOMAC function


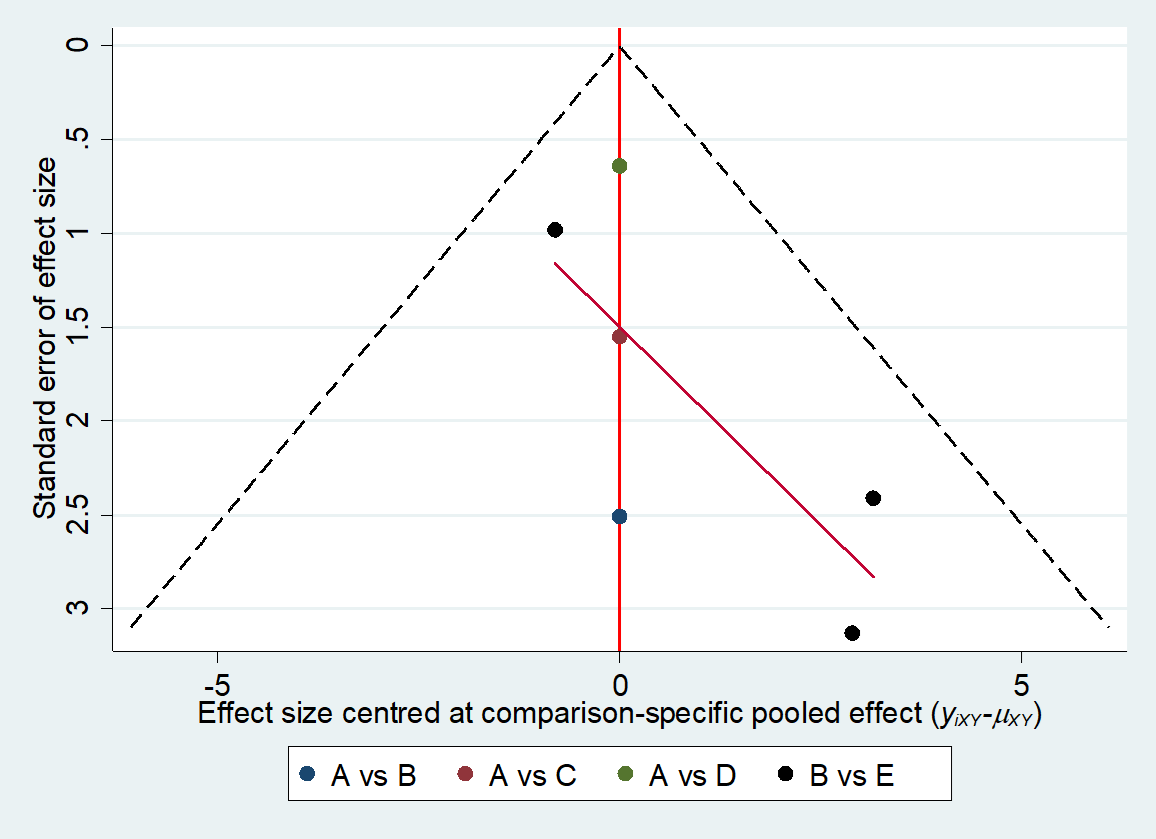


Abbreviation: A = AC, active drug comparator; B = BE, bioavailability-enhanced curcuminoid preparations; C = CT, conventional curcuminoid preparations; D = CT + AC, conventional curcuminoid preparations + active drug comparator; E = P, placebo

### eFigure J4 Adjusted funnel plot for VAS

Abbreviation: A = AC, active drug comparator; B = BE, bioavailability-enhanced curcuminoid preparations; C = BE + AC, bioavailability-enhanced curcuminoid preparations + active drug comparator; D = CT + AC, conventional curcuminoid preparations + active drug comparator; E = P, placebo; F = PLS, polysaccharide preparations

# **Appendix K**

## **Transitivity assessment tables**

### eTable K1 Descriptive table for the transitivity assessment of potential effect modifiers on WOMAC pain outcome

| _Contrast | Studies | Mean age in years (range), SD | Female in percentage (range) | Mean BMI in kg/m^2^ (range), SD | Mean duration of baseline knee OA/pain in months (range), SD | Mean baseline WOMAC pain intensity (range), SD | Follow-up period in days (range) |
| --- | --- | --- | --- | --- | --- | --- | --- |
| BE vs. P | 4 | 55.57 (53.12 to 57.57), 7.96 | 85.23 (73.7 to 96.6) | 27.65 (24.92 to 29.64), 3.21 | 23.25 (21.69 to 24.8), 9.23 | 8.13 (5.85 to 10.5), 2.91 | 57 (42 to 84) |
| CT+AC vs. AC | 1 | 50.25 (50.23 to 50.27), 8.36 | 64.45 (61 to 67.9) | 27.86 (27.4 to 28.32), 5.41 | NA | 15.20 (15.1 to 15.29), 2.55 | 120 |
| CT vs. AC | 1 | 60.6 (60.3 to 60.9), 6.85 | 89.35 (86.9 to 91.81) | 26.55 (26.5 to 26.6), 3.85 | 51.65 (51.3 to 52), 52.55 | 10.7 (10.6 to 10.8), 3.5 | 28 |
| BE vs. AC | 1 | 51.95 (50.8 to 53.1), 10.4 | 74.35 (72.6 to 76.1) | NA | NA | NA | 42 |

Abbreviation: AC, active drug comparator; BE, bioavailability-enhanced curcuminoid preparations; CT, conventional curcuminoid preparations; CT + AC, conventional curcuminoid preparations + active drug comparator; P, placebo

### eTable K2 Descriptive table for the transitivity assessment of potential effect modifiers on WOMAC stiffness outcome

| _Contrast | Studies | Mean age in years (range), SD | Female in percentage (range) | Mean BMI in kg/m^2^ (range), SD | Mean duration of baseline knee OA/pain in months (range), SD | Mean baseline WOMAC stiffness intensity (range), SD | Follow-up period in days (range) |
| --- | --- | --- | --- | --- | --- | --- | --- |
| BE vs. P | 3 | 55.64 (53.12 to 57.57),7.71 | 80.98 (73.7 to 88.6) | 27.19 (24.92 to 29.64), 3,08 | 23.25 (21.69 to 24.8), 9.23 | 2.12 (0.28 to 4.72), 1.20 | 48 (42 to 60) |
| CT+AC vs. AC | 1 | 50.25 (50.23 to 50.27), 8.36 | 64.45 (61 to 67.9) | 27.86 (27.4 to 28.82), 5.41 | NA | 5.43 (5.31 to 5.55), 1.46 | 120 |
| CT vs. AC | 1 | 60.6 (60.3 to 60.9), 6.85 | 89.35 (86.9 to 91.81) | 26.55 (26.5 to 26.6), 3.85 | 51.65 (51.3 to 52), 52.55 | 4.12 (4.08 to 4.16), 2.08 | 28 |
| BE vs. AC | 1 | 51.95 (50.8 to 53.1), 10.4 | 74.35 (72.6 to 76.1) | NA | NA | NA | 42 |

Abbreviation: AC, active drug comparator; BE, bioavailability-enhanced curcuminoid preparations; CT, conventional curcuminoid preparations; CT + AC, conventional curcuminoid preparations + active drug comparator; P, placebo

### eTable K3 Descriptive table for the transitivity assessment of potential effect modifiers on WOMAC function outcome

| _Contrast | Studies | Mean age in years (range), SD | Female in percentage (range) | Mean BMI in kg/m^2^ (range), SD | Mean duration of baseline knee OA/pain in months (range), SD | Mean baseline WOMAC function intensity (range), SD | Follow-up period in days (range) |
| --- | --- | --- | --- | --- | --- | --- | --- |
| BE vs. P | 3 | 55.64 (53.12 to 57.57),7.71 | 80.98 (73.7 to 88.6) | 27.19 (24.92 to 29.64), 3,08 | 23.25 (21.69 to 24.8), 9.23 | 27.28 (24.32 to 32.4), 6.81 | 48 (42 to 60) |
| CT+AC vs. AC | 1 | 50.25 (50.23 to 50.27), 8.36 | 64.45 (61 to 67.9) | 27.86 (27.4 to 28.82), 5.41 | NA | 52.51 (50.99 to 54.03), 6.08 | 120 |
| CT vs. AC | 1 | 60.6 (60.3 to 60.9), 6.85 | 89.35 (86.9 to 91.81) | 26.55 (26.5 to 26.6), 3.85 | 51.65 (51.3 to 52), 52.55 | 35.36 (34.68 to 36.04), 12.92 | 28 |
| BE vs. AC | 1 | 51.95 (50.8 to 53.1), 10.4 | 74.35 (72.6 to 76.1) | NA | NA | NA | 42 |

Abbreviation: AC, active drug comparator; BE, bioavailability-enhanced curcuminoid preparations; CT, conventional curcuminoid preparations; CT + AC, conventional curcuminoid preparations + active drug comparator; P, placebo

### eTable K4 Descriptive table for the transitivity assessment of potential effect modifiers on VAS outcome

| _Contrast | Studies | Mean age in years (range), SD | Female in percentage (range) | Mean BMI in kg/m^2^ (range), SD | Mean duration of baseline knee OA/pain in months (range), SD | Mean baseline VAS pain intensity (range), SD | Follow-up period in days (range) |
| --- | --- | --- | --- | --- | --- | --- | --- |
| BE vs. P | 3 | 57.95 (53.12 to 63.3), 8.52 | 78.13 (73.7 to 82.19) | 28.01 (24.92 to 29.89), 3.76 | 87.68 (84.16 to 91.2), 92.57 | 58.92 (52.37 to 66.32), 11.58 | 64 (42 to 90) |
| BE vs. AC | 1 | 52.62 (52.14 to 53.09), 3.97 | 33.07 (30.43 to 35.71) | NA | 7.43 (7.4 to 7.45), 3.34 | 78.25 (78.1 to 78.4), 6.80 | 28 |
| BE+AC vs. AC | 1 | 48.70 (48.26 to 49.13), 5.46 | 100 | 21.95 (21.9 to 22), 1.45 | 54.96 (53.52 to 56.4), 30.21 | 81.95 (79.3 to 84.6), 17.23 | 90 |
| CT+AC vs. AC | 1 | 50.25 (50.23 to 50.27), 8.36 | 64.45 (61 to 67.9) | 27.86 (27.4 to 28.82), 5.41 | NA | 78 (76.6 to 79.4), 12.08 | 120 |
| PLS vs. P | 1 | 56.70 (56.63 to 56.77), 10.28 | 56.67 | 27.49 (27.01 to 27.97), 4.41 | > 6 | 64 (61.5 to 66.5), 17.39 | 42 |

Abbreviation: AC, active drug comparator; BE, bioavailability-enhanced curcuminoid preparations; BE + AC, bioavailability-enhanced curcuminoid preparations + active drug comparator; CT + AC, conventional curcuminoid preparations + active drug comparator; P, placebo; PLS, polysaccharide preparations

# **Appendix L**

## **Adverse events**

Out of the seventeen included studies, five studies were excluded for reporting only the total number of participants experiencing adverse events (AE), without specifying the number of participants for each AE [8, 9, 11, 13, 17]. For the remaining twelve studies, a meta-analysis was not conducted.

### eTable L Frequency of adverse events across different treatment groups

| Type of ADR | P (N= 236) | AC (N=232) | BE (N=260) | BE + AC (N=15) | CT + AC (N=114) | PLS  (N=66) |
| --- | --- | --- | --- | --- | --- | --- |
| All adverse events | 41 (17.36%) | 17 (7.32%) | 24 (9.22%) | 0 (0%) | 3 (2.63%) | 20 (30.30%) |
| GI symptoms | 12 (5.08%) | 8 (3.45%) | 17 (6.54%) | 0 (0%) | 2 (1.75%) | 4 (6.06%) |
| Respiratory | 1 (0.42%) | 0 (0%) | 0 (0%) | 0 (0%) | 0 (0%) | 1 (1.52%) |
| Neurological and sensory symptoms | 2 (0.85%) | 4 (1.72%) | 5 (1.92%) | 0 (0%) | 0 (0%) | 2 (3.03%) |
| Dermatological reactions | 1 (0.42%) | 1 (0.43%) | 1 (0.38%) | 0 (0%) | 1 (0.88%) | 0 (0%) |
| Cardiovascular and edema | 1 (0.42%) | 0 (0%) | 1 (0.38%) | 0 (0%) | 0 (0%) | 0 (0%) |
| Metabolic effects and weight changes | 2 (0.85%) | 0 (0%) | 0 (0%) | 0 (0%) | 0 (0%) | 0 (0%) |
| General body pain | 5 (2.12%) | 0 (0%) | 0 (0%) | 0 (0%) | 0 (0%) | 1 (1.52%) |
| Complications and medical problems | 0 (0%) | 4 (1.72%) | 0 (0%) | 0 (0%) | 0 (0%) | 0 (0%) |
| Miscellaneous | 17 (7.20%) | 0 (0%) | 0 (0%) | 0 (0%) | 0 (0%) | 12 (18.18%) |

Abbreviation: AC, active drug comparator; ADR, adverse drug reaction; BE, bioavailability-enhanced curcuminoid preparations; CT, conventional curcuminoid preparations; GI, gastrointestinal; P, placebo; PLS, polysaccharide preparations

# **Appendix M**

## **Certainty of evidence**

### eTable M Grading

| **Comparison** | **Direct evidence** | | **Indirect evidence** | | **Network meta-analysis** | |
| --- | --- | --- | --- | --- | --- | --- |
|  | **Mean difference (95% confidence interval)** | **Quality of evidence** | **Mean difference (95% confidence interval)** | **Quality of evidence** | **Mean difference (95% confidence interval)** | **Quality of evidence** |
| **WOMAC Pain** | | | | | | |
| BE vs. AC | 0.86 (-0.61 to 2.33) | ⨁⨁⨁◯^a^  MODERATE | -2.80 (-848.71 to 843.11) | ⨁⨁◯◯^b^  LOW | 0.86 (-0.90 to 2.62) | ⨁⨁◯◯^a,c^  LOW |
| CT vs. AC | 0.16 (-0.72 to 1.04) | ⨁⨁⨁◯^a^  MODERATE | 6.64 (-4578.06 to 4591.35) | ⨁⨁◯◯^b^  LOW | 0.16 (-1.15 to 1.47) | ⨁⨁◯◯^a,c^  LOW |
| CT + AC vs. AC | -0.68 (-1.14 to -0.22) | ⨁⨁⨁⨁  HIGH | 6.64 (-1745.58 to 1758.85) | ⨁⨁◯◯^b^  LOW | -0.68 (-1.75 to 0.39) | ⨁⨁◯◯^a,c^  LOW |
| BE vs. P | -2.47 (-3.25 to -1.68) | ⨁⨁◯◯^a,d^  LOW | 1.08 (-833.42 to 835.59) | ⨁⨁◯◯^b^  LOW | -2.47 (-3.27 to -1.67) | ⨁◯◯◯^a,c,d^  VERY LOW |
| **WOMAC Stiffness** | | | | | | |
| BE vs. AC | -0.60 (-1.23 to 0.03) | ⨁⨁⨁◯^a^  MODERATE | -0.85 (-719.56 to 717.86) | ⨁⨁◯◯^b^  LOW | -0.60 (-2.13 to 0.93) | ⨁⨁⨁◯^a^  MODERATE |
| CT vs. AC | 0.10 (-0.31 to 0.50) | ⨁⨁⨁⨁  HIGH | 0.36 (-2351.42 to 2352.15) | ⨁⨁◯◯^b^  LOW | 0.10 (-1.36 to 1.56) | ⨁⨁⨁◯^a^  MODERATE |
| CT + AC vs. AC | -0.08 (-0.57 to 0.41) | ⨁⨁⨁⨁  HIGH | 0.35 (-1955.96 to 1956.67) | ⨁⨁◯◯^b^  LOW | -0.08 (-1.56 to 1.40) | ⨁⨁◯◯^a,c^  LOW |
| BE vs. P | -0.78 (-1.58 to 0.03) | ⨁◯◯◯ ^a,d,f^  VERY LOW | -0.51 (-639.98 to 638.96) | ⨁⨁◯◯^b^  LOW | -0.78 (-1.63 to 0.08) | ⨁◯◯◯ ^a,d,f^  VERY LOW |

eTable M Grading (cont.)

| **Comparison** | **Direct evidence** | | **Indirect evidence** | | **Network meta-analysis** | |
| --- | --- | --- | --- | --- | --- | --- |
|  | **Mean difference (95% confidence interval)** | **Quality of evidence** | **Mean difference (95% confidence interval)** | **Quality of evidence** | **Mean difference (95% confidence interval)** | **Quality of evidence** |
| **WOMAC Function** | | | | | | |
| BE vs. AC | 4.81 (-0.11 to 9.73) | ⨁⨁⨁◯^a^  MODERATE | -10.58 (-2399.80 to 2378.64) | ⨁⨁◯◯^b^  LOW | 4.81 (-1.24 to 10.86) | ⨁⨁◯◯^a,c^  LOW |
| CT vs. AC | 1.02 (-2.01 to 4.05) | ⨁⨁⨁◯^a^  MODERATE | 28.99 (-16620.51 to 16678.49) | ⨁⨁◯◯^b^  LOW | 1.02 (-3.63 to 5.67) | ⨁⨁◯◯^a,c^  LOW |
| CT + AC vs. AC | -1.74 (-3.00 to -0.48) | ⨁⨁⨁◯^a^  MODERATE | 28.97 (-5225.17 to 5283.12) | ⨁⨁◯◯^b^  LOW | -1.74 (-5.48 to 2.00) | ⨁⨁◯◯^a,c^  LOW |
| BE vs. P | -9.62 (-12.47 to -6.76) | ⨁⨁◯◯^a,d^  LOW | 5.35 (-2317.66 to 2328.36) | ⨁⨁◯◯^b^  LOW | -9.69 (-13.16 to -6.23) | ⨁◯◯◯^a,c,d^  VERY LOW |
| **VAS** | | | | | | |
| PLS vs. P | -26.55 (-36.53 to -16.57) | ⨁⨁◯◯^a,e^  LOW | Not estimable | Not estimable | -26.55 (-36.53 to -16.57) | ⨁⨁◯◯^a,e^  LOW |
| BE vs. AC | 0.00 (-2.38 to 2.38) | ⨁⨁⨁◯^a^  MODERATE | -33.57 (-3996.87 to 3929.73) | ⨁⨁◯◯^b^  LOW | 0.00 (-2.38 to 2.38) | ⨁⨁◯◯^a,c^  LOW |
| BE + AC vs. AC | -57.00 (-64.21 to -49.79) | ⨁⨁⨁◯^a^  MODERATE | 33.61 (-12802.80 to 12870.03) | ⨁⨁◯◯^b^  LOW | -57.00 (-64.21 to -49.79) | ⨁⨁◯◯^a,c^  LOW |
| CT + AC vs. AC | -10.80 (-13.96 to -7.64) | ⨁⨁⨁◯^a^  MODERATE | 33.22 (-11232.34 to 11298.79) | ⨁⨁◯◯^b^  LOW | -10.80 (-13.96 to -7.64) | ⨁⨁◯◯^a,c^  LOW |

eTable M Grading (cont.)

| **Comparison** | **Direct evidence** | | **Indirect evidence** | | **Network meta-analysis** | |
| --- | --- | --- | --- | --- | --- | --- |
|  | **Mean difference (95% confidence interval)** | **Quality of evidence** | **Mean difference (95% confidence interval)** | **Quality of evidence** | **Mean difference (95% confidence interval)** | **Quality of evidence** |
| BE vs. P | -16.77 (-20.94 to -12.60) | ⨁⨁◯◯^a,d^  LOW | 8.40 (-3156.18 to 3172.99) | ⨁⨁◯◯^b^  LOW | -16.81 (-20.85 to -12.77) | ⨁◯◯◯^a,c,d^  VERY LOW |

**Explanations:** ^a^ Downgraded one level due to serious imprecision, ^b^ Downgraded two levels due to very serious imprecision, ^c^ Downgraded one level due to incoherence, ^d^ Downgraded one level due to publication bias, ^e^ Downgraded one level due to risk of bias, ^f^ Downgraded two levels due to very serious inconsistency

# **Appendix N**

## **Sensitivity analyses**

### eFigure N1 Sensitivity analysis of follow-up period for WOMAC pain


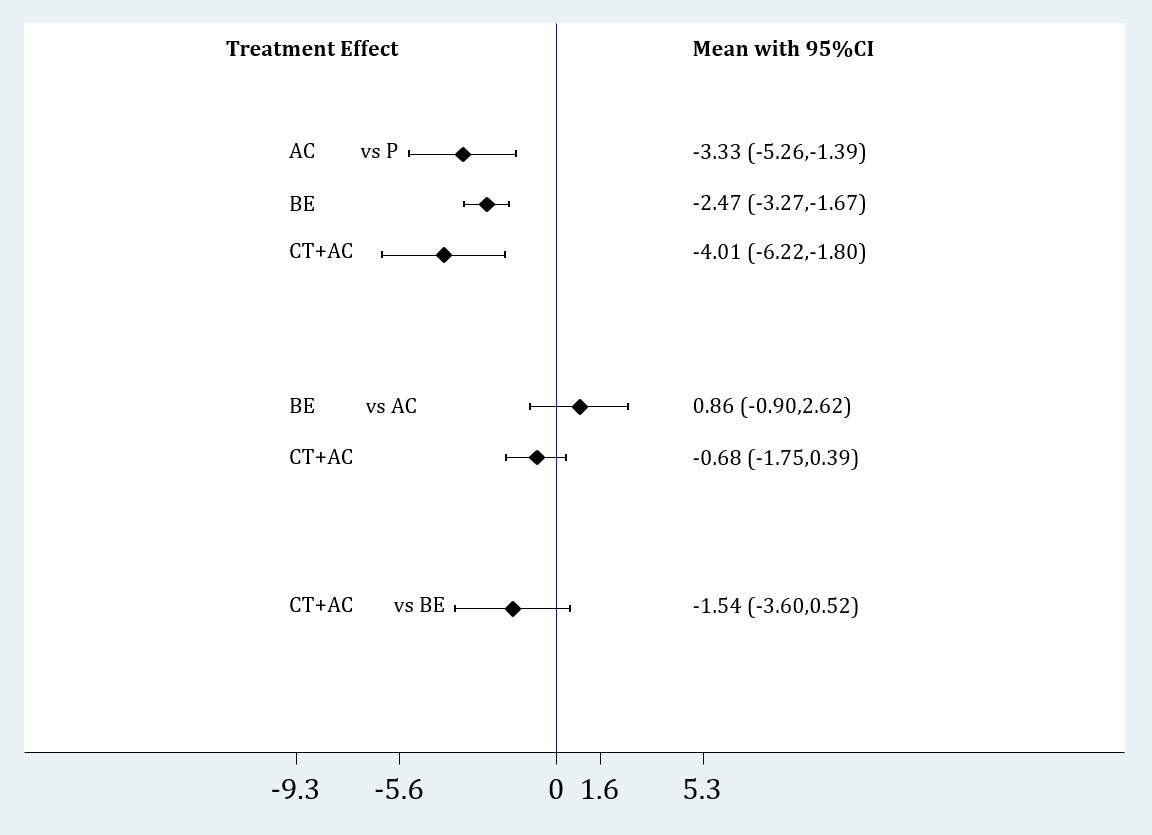
 (chi-square for global inconsistency = 36.59, *p* = 0.0000)

Abbreviation: AC, active drug comparator; BE, bioavailability-enhanced curcuminoid preparations; CT, conventional curcuminoid preparations; CT + AC, conventional curcuminoid preparations + active drug comparator; P, placebo

### eTable N1 Descriptive table for transitivity assessment of sensitivity analysis on follow-up period for WOMAC pain

| _Contrast | Studies | Mean age in years (range), SD | Female in percentage (range) | Mean BMI in kg/m^2^ (range), SD | Mean duration of baseline knee OA/pain in months (range), SD | Mean baseline WOMAC pain intensity (range), SD | Follow-up period in days (range) |
| --- | --- | --- | --- | --- | --- | --- | --- |
| BE vs. P | 4 | 55.57 (53.12 to 57.57), 7.96 | 85.23 (73.7 to 96.6) | 27.65 (24.92 to 29.64), 3.21 | 23.25 (21.69 to 24.8), 9.23 | 8.13 (5.85 to 10.5), 2.91 | 57 (42 to 84) |
| CT+AC vs. AC | 1 | 50.25 (50.23 to 50.27), 8.36 | 64.45 (61 to 67.9) | 27.86 (27.4 to 28.32), 5.41 | NA | 15.20 (15.1 to 15.29), 2.55 | 120 |
| BE vs. AC | 1 | 51.95 (50.8 to 53.1), 10.4 | 74.35 (72.6 to 76.1) | NA | NA | NA | 42 |

Abbreviation: AC, active drug comparator; BE, bioavailability-enhanced curcuminoid preparations; CT + AC, conventional curcuminoid preparations + active drug comparator; P, placebo

### eFigure N2 Sensitivity analysis of follow-up period for WOMAC stiffness


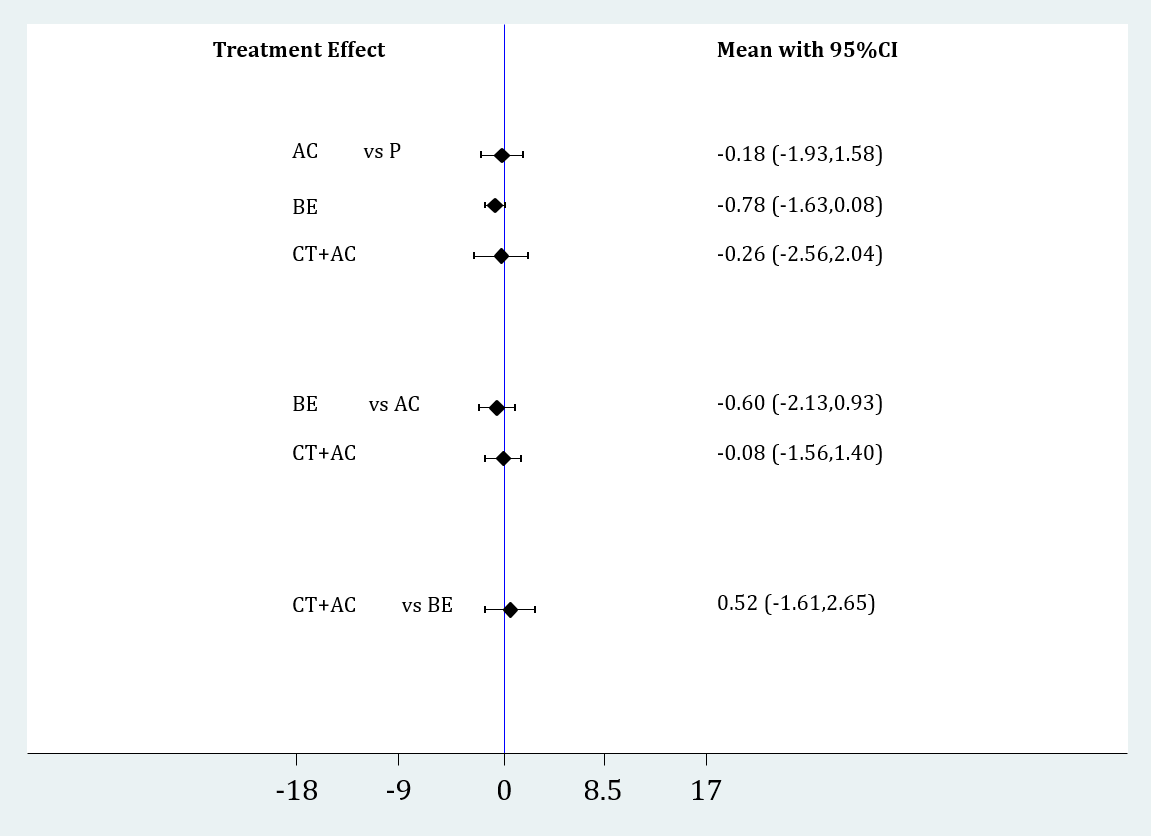


(chi-square for global inconsistency = 3.17, *p* = 0.0748)

Abbreviation: AC, active drug comparator; BE, bioavailability-enhanced curcuminoid preparations; CT, conventional curcuminoid preparations; CT + AC, conventional curcuminoid preparations + active drug comparator; P, placebo

### eTable N2 Descriptive table for transitivity assessment of sensitivity analysis on follow-up period for WOMAC stiffness

| _Contrast | Studies | Mean age in years (range), SD | Female in percentage (range) | Mean BMI in kg/m^2^ (range), SD | Mean duration of baseline knee OA/pain in months (range), SD | Mean baseline WOMAC stiffness intensity (range), SD | Follow-up period in days (range) |
| --- | --- | --- | --- | --- | --- | --- | --- |
| BE vs. P | 3 | 55.64 (53.12 to 57.57),7.71 | 80.98 (73.7 to 88.6) | 27.19 (24.92 to 29.64), 3,08 | 23.25 (21.69 to 24.8), 9.23 | 2.12 (0.28 to 4.72), 1.20 | 48 (42 to 60) |
| CT+AC vs. AC | 1 | 50.25 (50.23 to 50.27), 8.36 | 64.45 (61 to 67.9) | 27.86 (27.4 to 28.82), 5.41 | NA | 5.43 (5.31 to 5.55), 1.46 | 120 |
| BE vs. AC | 1 | 51.95 (50.8 to 53.1), 10.4 | 74.35 (72.6 to 76.1) | NA | NA | NA | 42 |

Abbreviation: AC, active drug comparator; BE, bioavailability-enhanced curcuminoid preparations; CT + AC, conventional curcuminoid preparations + active drug comparator; P, placebo

### eFigure N3 Sensitivity analysis of follow-up period for WOMAC function


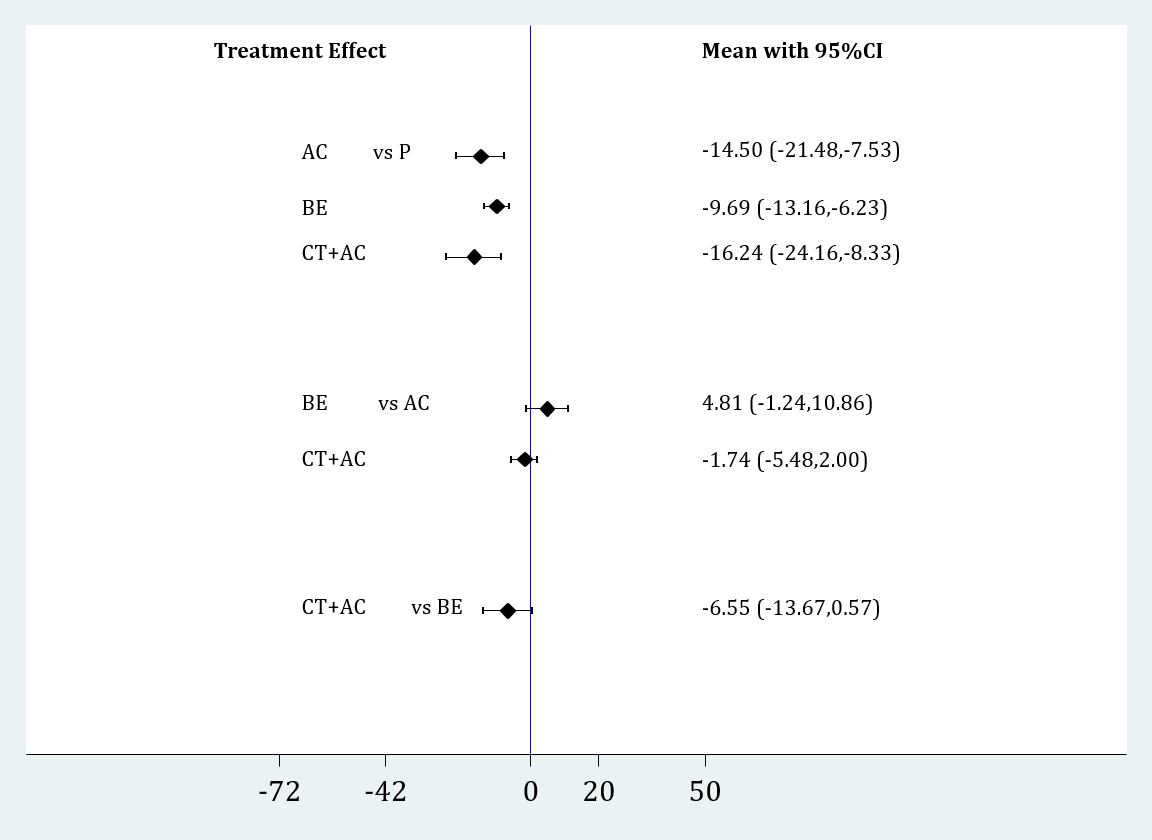


(chi-square for global inconsistency = 30.11, *p* = 0.0000)

Abbreviation: AC, active drug comparator; BE, bioavailability-enhanced curcuminoid preparations; CT, conventional curcuminoid preparations; CT + AC, conventional curcuminoid preparations + active drug comparator; P, placebo

### eTable N3 Descriptive table for transitivity assessment of sensitivity analysis on follow-up period for WOMAC function

| _Contrast | Studies | Mean age in years (range), SD | Female in percentage (range) | Mean BMI in kg/m^2^ (range), SD | Mean duration of baseline knee OA/pain in months (range), SD | Mean baseline WOMAC function intensity (range), SD | Follow-up period in days (range) |
| --- | --- | --- | --- | --- | --- | --- | --- |
| BE vs. P | 3 | 55.64 (53.12 to 57.57),7.71 | 80.98 (73.7 to 88.6) | 27.19 (24.92 to 29.64), 3,08 | 23.25 (21.69 to 24.8), 9.23 | 27.28 (24.32 to 32.4), 6.81 | 48 (42 to 60) |
| CT+AC vs. AC | 1 | 50.25 (50.23 to 50.27), 8.36 | 64.45 (61 to 67.9) | 27.86 (27.4 to 28.82), 5.41 | NA | 52.51 (50.99 to 54.03), 6.08 | 120 |
| BE vs. AC | 1 | 51.95 (50.8 to 53.1), 10.4 | 74.35 (72.6 to 76.1) | NA | NA | NA | 42 |

Abbreviation: AC, active drug comparator; BE, bioavailability-enhanced curcuminoid preparations; CT + AC, conventional curcuminoid preparations + active drug comparator; P, placebo

### eFigure N4 Sensitivity analysis of high risk of bias for WOMAC pain


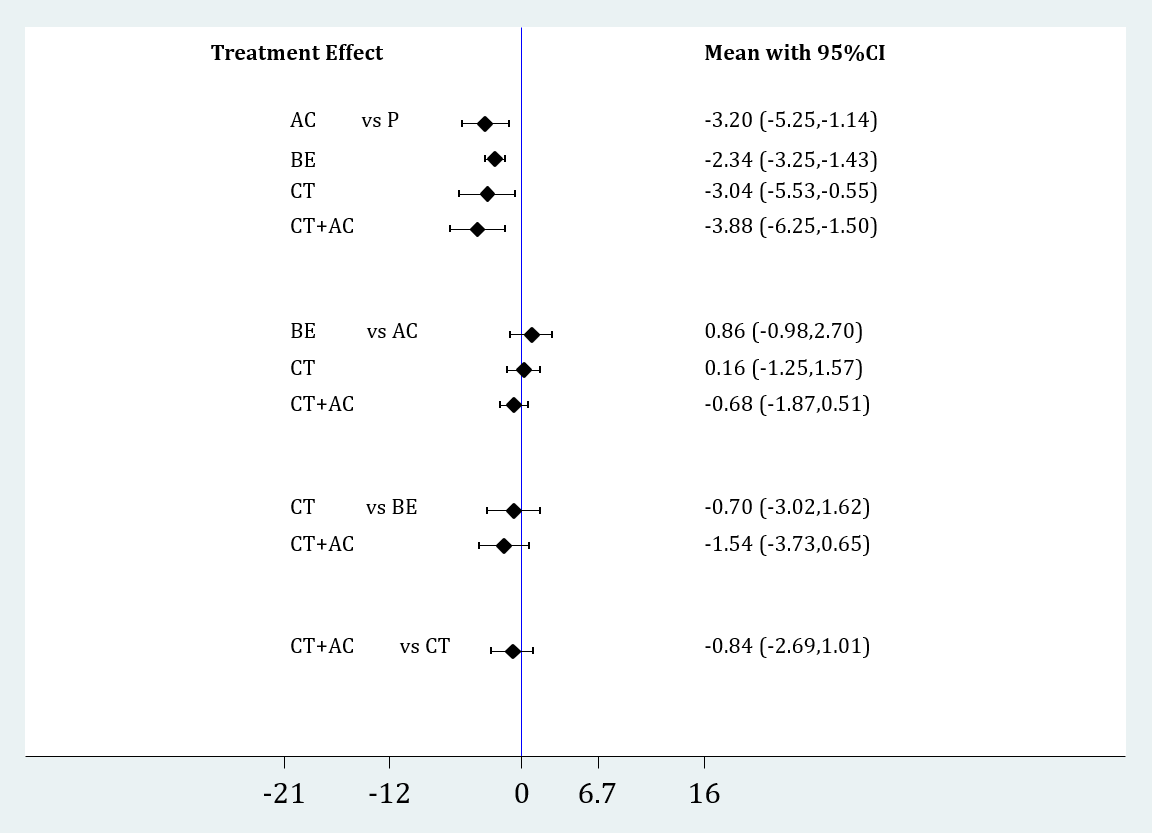


(chi-square for global inconsistency = 25.32, *p* = 0.0000)

Abbreviation: AC, active drug comparator; BE, bioavailability-enhanced curcuminoid preparations; CT, conventional curcuminoid preparations; CT + AC, conventional curcuminoid preparations + active drug comparator; P, placebo

### eTable N4 Descriptive table for transitivity assessment of sensitivity analysis on high risk of bias for WOMAC pain

| _Contrast | Studies | Mean age in years (range), SD | Female in percentage (range) | Mean BMI in kg/m^2^ (range), SD | Mean duration of baseline knee OA/pain in months (range), SD | Mean baseline WOMAC pain intensity (range), SD | Follow-up period in days (range) |
| --- | --- | --- | --- | --- | --- | --- | --- |
| BE vs. P | 3 | 54.94 (53.12 to 56.54), 7.63 | 89.18 (80.60 to 96.6) | 26.88 (24.92 to 28.81), 2.91 | 23.25 (21.69 to 24.8), 9.23 | 7.44 (5.85 to 8.47), 2.53 | 62 (42 to 84) |
| CT+AC vs. AC | 1 | 50.25 (50.23 to 50.27), 8.36 | 64.45 (61 to 67.9) | 27.86 (27.4 to 28.32), 5.41 | NA | 15.20 (15.1 to 15.29), 2.55 | 120 |
| CT vs. AC | 1 | 60.6 (60.3 to 60.9), 6.85 | 89.35 (86.9 to 91.81) | 26.55 (26.5 to 26.6), 3.85 | 51.65 (51.3 to 52), 52.55 | 10.7 (10.6 to 10.8), 3.5 | 28 |
| BE vs. AC | 1 | 51.95 (50.8 to 53.1), 10.4 | 74.35 (72.6 to 76.1) | NA | NA | NA | 42 |

Abbreviation: AC, active drug comparator; BE, bioavailability-enhanced curcuminoid preparations; CT, conventional curcuminoid preparations; CT + AC, conventional curcuminoid preparations + active drug comparator; P, placebo

### eFigure N5 Sensitivity analysis of high risk of bias for WOMAC stiffness


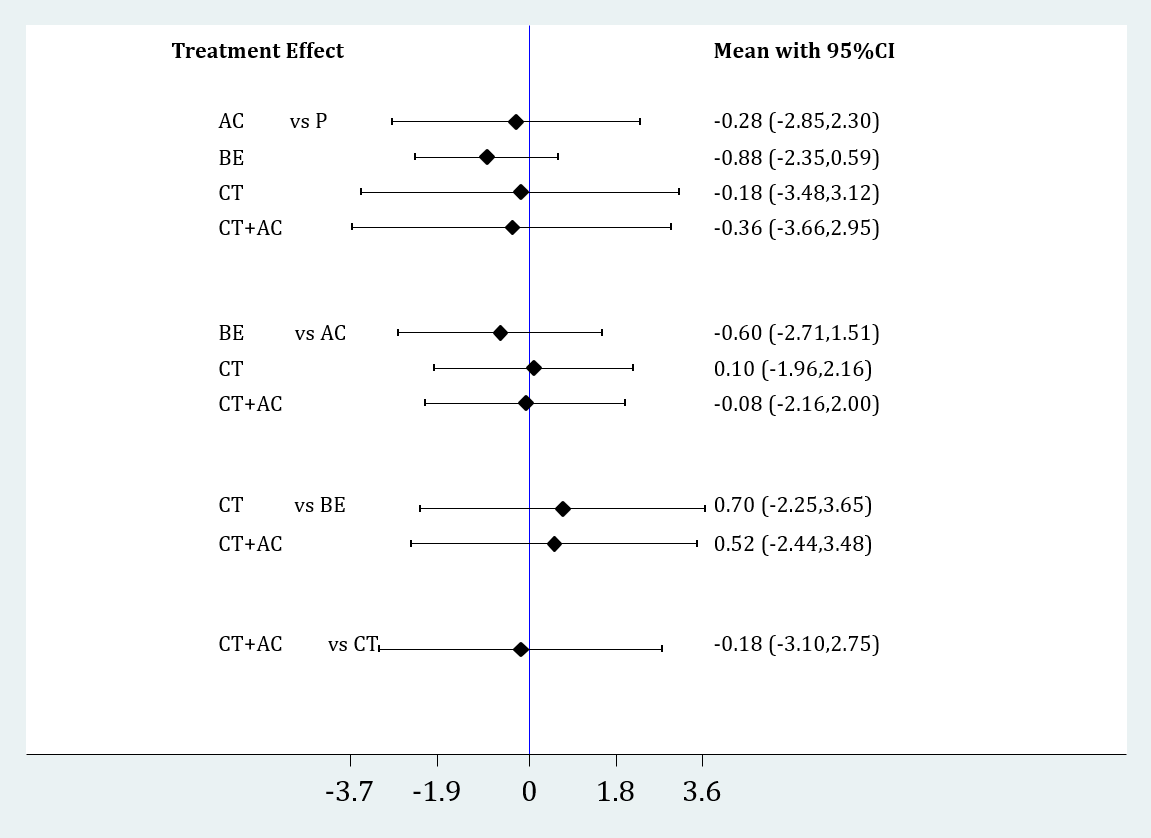


(chi-square for global inconsistency = 1.36, *p* = 0.2427)

Abbreviation: AC, active drug comparator; BE, bioavailability-enhanced curcuminoid preparations; CT, conventional curcuminoid preparations; CT + AC, conventional curcuminoid preparations + active drug comparator; P, placebo

### eTable N5 Descriptive table for transitivity assessment of sensitivity analysis on high risk of bias for WOMAC stiffness

| _Contrast | Studies | Mean age in years (range), SD | Female in percentage (range) | Mean BMI in kg/m^2^ (range), SD | Mean duration of baseline knee OA/pain in months (range), SD | Mean baseline WOMAC stiffness intensity (range), SD | Follow-up period in days (range) |
| --- | --- | --- | --- | --- | --- | --- | --- |
| BE vs. P | 2 | 54.75 (53.12 to 56.54), 7.10 | 84.60 (80.6 to 88.6) | 25.18 (24.92 to 25.44), 2.34 | 23.25 (21.69 to 24.8), 9.23 | 2.43 (0.28 to 4.72), 0.93 | 51 (42 to 60) |
| CT+AC vs. AC | 1 | 50.25 (50.23 to 50.27), 8.36 | 64.45 (61 to 67.9) | 27.86 (27.4 to 28.82), 5.41 | NA | 5.43 (5.31 to 5.55), 1.46 | 120 |
| CT vs. AC | 1 | 60.6 (60.3 to 60.9), 6.85 | 89.35 (86.9 to 91.81) | 26.55 (26.5 to 26.6), 3.85 | 51.65 (51.3 to 52), 52.55 | 4.12 (4.08 to 4.16), 2.08 | 28 |
| BE vs. AC | 1 | 51.95 (50.8 to 53.1), 10.4 | 74.35 (72.6 to 76.1) | NA | NA | NA | 42 |

Abbreviation: AC, active drug comparator; BE, bioavailability-enhanced curcuminoid preparations; CT, conventional curcuminoid preparations; CT + AC, conventional curcuminoid preparations + active drug comparator; P, placebo

### eFigure N6 Sensitivity analysis of high risk of bias for WOMAC function


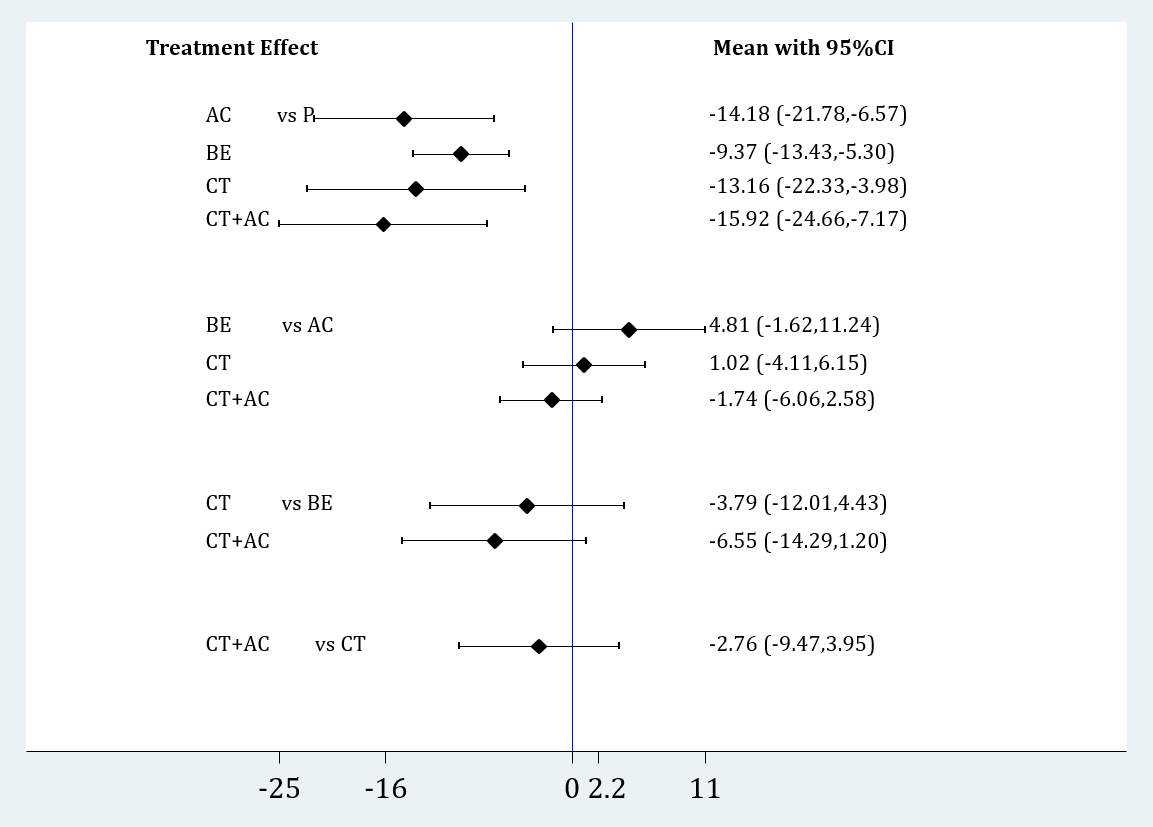


(chi-square for global inconsistency = 20.42, *p* = 0.0000)

Abbreviation: AC, active drug comparator; BE, bioavailability-enhanced curcuminoid preparations; CT, conventional curcuminoid preparations; CT + AC, conventional curcuminoid preparations + active drug comparator; P, placebo

### eTable N6 Descriptive table for transitivity assessment of sensitivity analysis on high risk of bias for WOMAC function

| _Contrast | Studies | Mean age in years (range), SD | Female in percentage (range) | Mean BMI in kg/m^2^ (range), SD | Mean duration of baseline knee OA/pain in months (range), SD | Mean baseline WOMAC function intensity (range), SD | Follow-up period in days (range) |
| --- | --- | --- | --- | --- | --- | --- | --- |
| BE vs. P | 2 | 54.75 (53.12 to 56.54), 7.10 | 84.60 (80.6 to 88.6) | 25.18 (24.92 to 25.44), 2.34 | 23.25 (21.69 to 24.8), 9.23 | 24.87 (24.32 to 25.56), 6.67 | 51 (42 to 60) |
| CT+AC vs. AC | 1 | 50.25 (50.23 to 50.27), 8.36 | 64.45 (61 to 67.9) | 27.86 (27.4 to 28.82), 5.41 | NA | 52.51 (50.99 to 54.03), 6.08 | 120 |
| CT vs. AC | 1 | 60.6 (60.3 to 60.9), 6.85 | 89.35 (86.9 to 91.81) | 26.55 (26.5 to 26.6), 3.85 | 51.65 (51.3 to 52), 52.55 | 35.36 (34.68 to 36.04), 12.92 | 28 |
| BE vs. AC | 1 | 51.95 (50.8 to 53.1), 10.4 | 74.35 (72.6 to 76.1) | NA | NA | NA | 42 |

Abbreviation: AC, active drug comparator; BE, bioavailability-enhanced curcuminoid preparations; CT, conventional curcuminoid preparations; CT + AC, conventional curcuminoid preparations + active drug comparator; P, placebo

### eFigure N7 Sensitivity analysis of high risk of bias for VAS


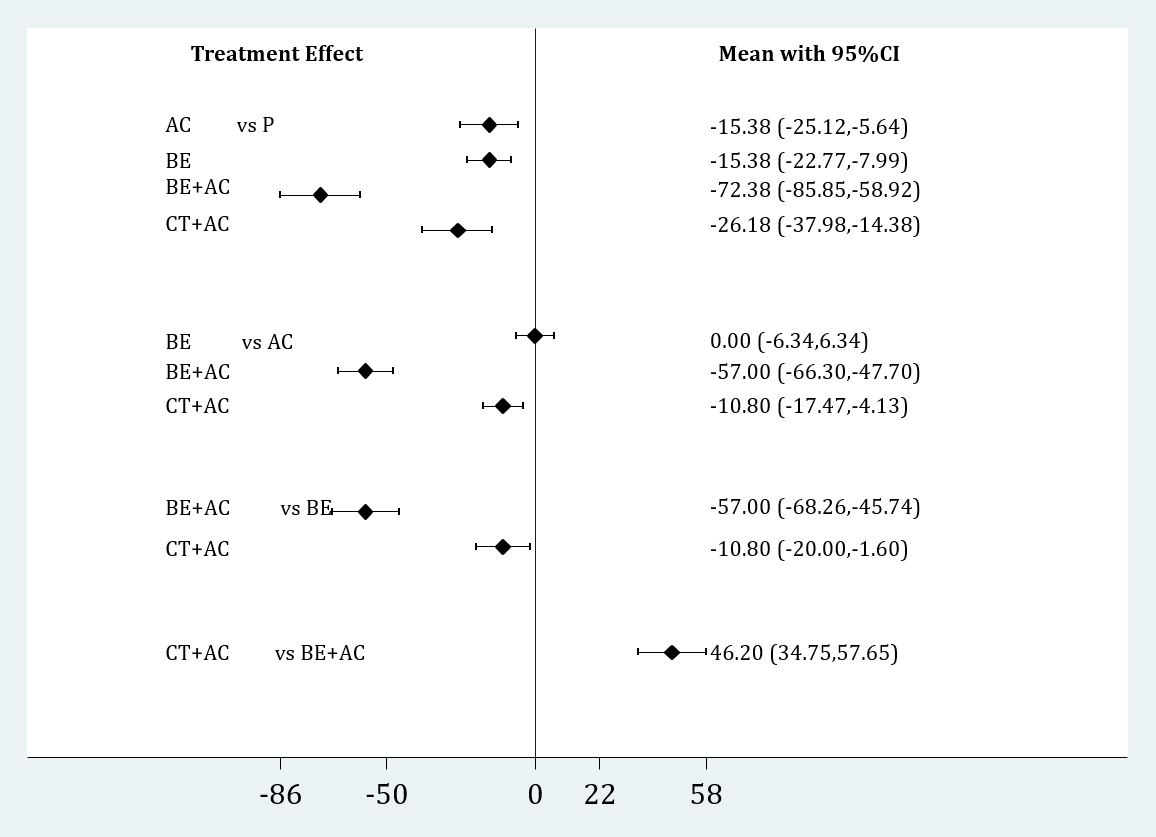


(chi-square for global inconsistency = 16.64, *p* = 0.0000)

Abbreviation: AC, active drug comparator; BE, bioavailability-enhanced curcuminoid preparations; BE + AC, bioavailability-enhanced curcuminoid preparations + active drug comparator; CT + AC, conventional curcuminoid preparations + active drug comparator; P, placebo

### eTable N7 Descriptive table for transitivity assessment of sensitivity analysis on high risk of bias for VAS

| _Contrast | Studies | Mean age in years (range), SD | Female in percentage (range) | Mean BMI in kg/m^2^ (range), SD | Mean duration of baseline knee OA/pain in months (range), SD | Mean baseline VAS pain intensity (range), SD | Follow-up period in days (range) |
| --- | --- | --- | --- | --- | --- | --- | --- |
| BE vs. P | 2 | 58.20 (53.12 to 63.3), 8.28 | 78.90 (75.6 to 82.19) | 27.42 (24.92 to 29.89), 3.73 | 87.68 (84.16 to 91.2), 92.57 | 57.04 (52.37 to 63.09), 9.49 | 75 (60 to 90) |
| BE vs. AC | 1 | 52.62 (52.14 to 53.09), 3.97 | 33.07 (30.43 to 35.71) | NA | 7.43 (7.4 to 7.45), 3.34 | 78.25 (78.1 to 78.4), 6.80 | 28 |
| BE+AC vs. AC | 1 | 48.70 (48.26 to 49.13), 5.46 | 100 | 21.95 (21.9 to 22), 1.45 | 54.96 (53.52 to 56.4), 30.21 | 81.95 (79.3 to 84.6), 17.23 | 90 |
| CT+AC vs. AC | 1 | 50.25 (50.23 to 50.27), 8.36 | 64.45 (61 to 67.9) | 27.86 (27.4 to 28.82), 5.41 | NA | 78 (76.6 to 79.4), 12.08 | 120 |

Abbreviation: AC, active drug comparator; BE, bioavailability-enhanced curcuminoid preparations; BE + AC, bioavailability-enhanced curcuminoid preparations + active drug comparator; CT + AC, conventional curcuminoid preparations + active drug comparator; P, placebo

### eFigure N8 Sensitivity analysis of small-study effects for WOMAC pain


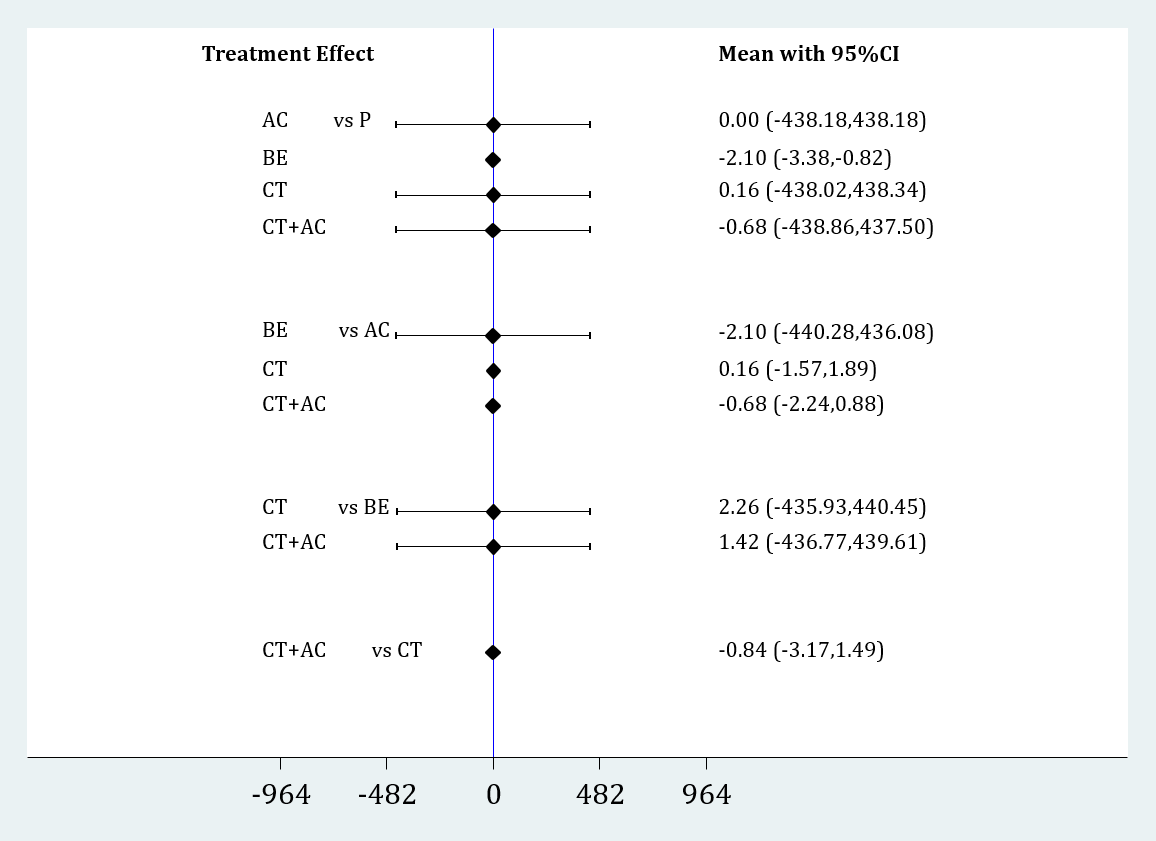


(chi-square for global consistency = 36.59, *p* = 0.0000)

Abbreviation: AC, active drug comparator; BE, bioavailability-enhanced curcuminoid preparations; CT, conventional curcuminoid preparations; CT + AC, conventional curcuminoid preparations + active drug comparator; P, placebo

### eTable N8 Descriptive table for transitivity assessment of sensitivity analysis on small-study effects for WOMAC pain

| _Contrast | Studies | Mean age in years (range), SD | Female in percentage (range) | Mean BMI in kg/m^2^ (range), SD | Mean duration of baseline knee OA/pain in months (range), SD | Mean baseline WOMAC pain intensity (range), SD | Follow-up period in days (range) |
| --- | --- | --- | --- | --- | --- | --- | --- |
| BE vs. P | 2 | 54.76 (53.12 to 56.04), 8.56 | 93.75 (90.9 to 96.6) | 26.88 (24.92 to 28.81), 2.91 | NA | 7.05 (5.85 to 8.24), 2.2 | 72 (60 to 84) |
| CT+AC vs. AC | 1 | 50.25 (50.23 to 50.27), 8.36 | 64.45 (61 to 67.9) | 27.86 (27.4 to 28.32), 5.41 | NA | 15.20 (15.1 to 15.29), 2.55 | 120 |
| CT vs. AC | 1 | 60.6 (60.3 to 60.9), 6.85 | 89.35 (86.9 to 91.81) | 26.55 (26.5 to 26.6), 3.85 | 51.65 (51.3 to 52), 52.55 | 10.7 (10.6 to 10.8), 3.5 | 28 |

Abbreviation: AC, active drug comparator; BE, bioavailability-enhanced curcuminoid preparations; CT, conventional curcuminoid preparations; CT + AC, conventional curcuminoid preparations + active drug comparator; P, placebo

### eFigure N9 Sensitivity analysis of small-study effects for WOMAC stiffness


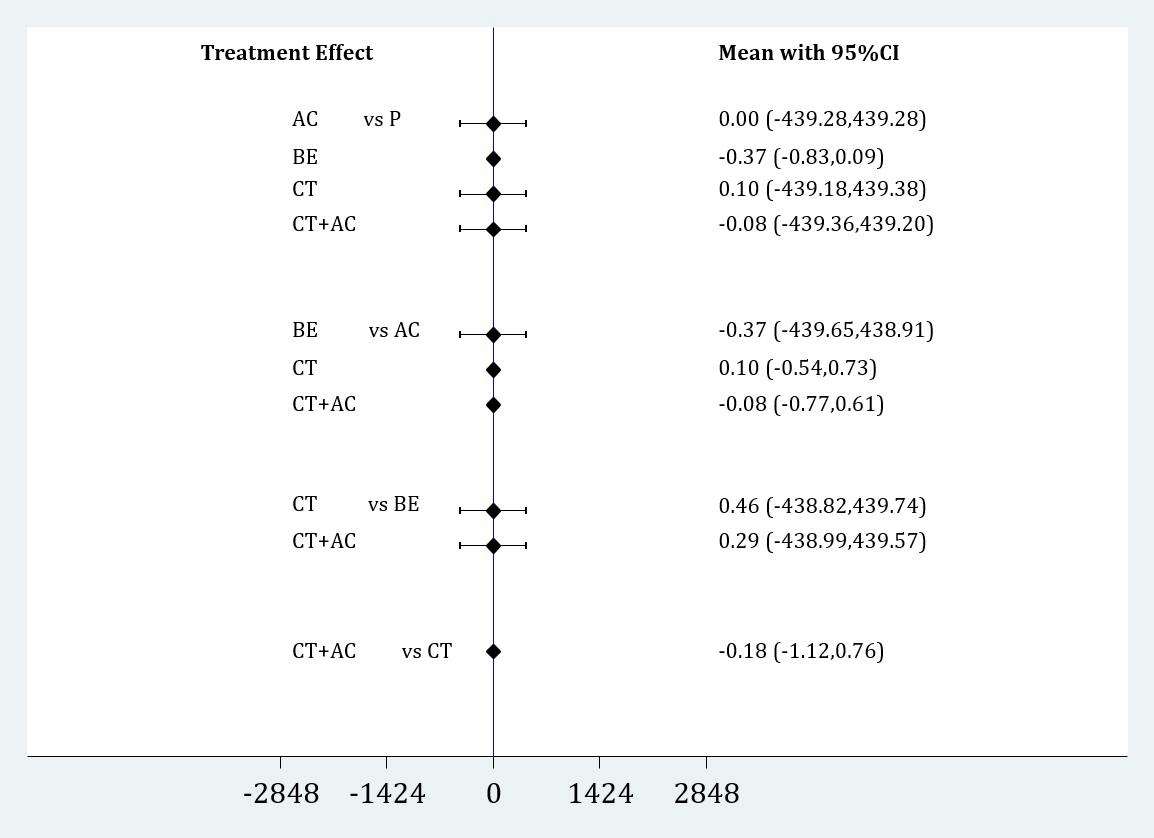


(chi-square for global consistency = 3.17, *p* = 0.748)

Abbreviation: AC, active drug comparator; BE, bioavailability-enhanced curcuminoid preparations; CT, conventional curcuminoid preparations; CT + AC, conventional curcuminoid preparations + active drug comparator; P, placebo

### eTable N9 Descriptive table for transitivity assessment of sensitivity analysis on small-study effects for WOMAC stiffness

| _Contrast | Studies | Mean age in years (range), SD | Female in percentage (range) | Mean BMI in kg/m^2^ (range), SD | Mean duration of baseline knee OA/pain in months (range), SD | Mean baseline WOMAC stiffness intensity (range), SD | Follow-up period in days (range) |
| --- | --- | --- | --- | --- | --- | --- | --- |
| BE vs. P | 2 | 56.39 (54.11 to 57.57), 7.35 | 80.98 (73.7 to 88.6) | 29.20 (28.75 to 29.64), 3.82 | 23.25 (21.69 to 24.8), 9.23 | 0.87 (0.28 to 1.7), 1.32 | 42 |
| CT+AC vs. AC | 1 | 50.25 (50.23 to 50.27), 8.36 | 64.45 (61 to 67.9) | 27.86 (27.4 to 28.82), 5.41 | NA | 5.43 (5.31 to 5.55), 1.46 | 120 |
| CT vs. AC | 1 | 60.6 (60.3 to 60.9), 6.85 | 89.35 (86.9 to 91.81) | 26.55 (26.5 to 26.6), 3.85 | 51.65 (51.3 to 52), 52.55 | 4.12 (4.08 to 4.16), 2.08 | 28 |

Abbreviation: AC, active drug comparator; BE, bioavailability-enhanced curcuminoid preparations; CT, conventional curcuminoid preparations; CT + AC, conventional curcuminoid preparations + active drug comparator; P, placebo

### eFigure N10 Sensitivity analysis of small-study effects for WOMAC function


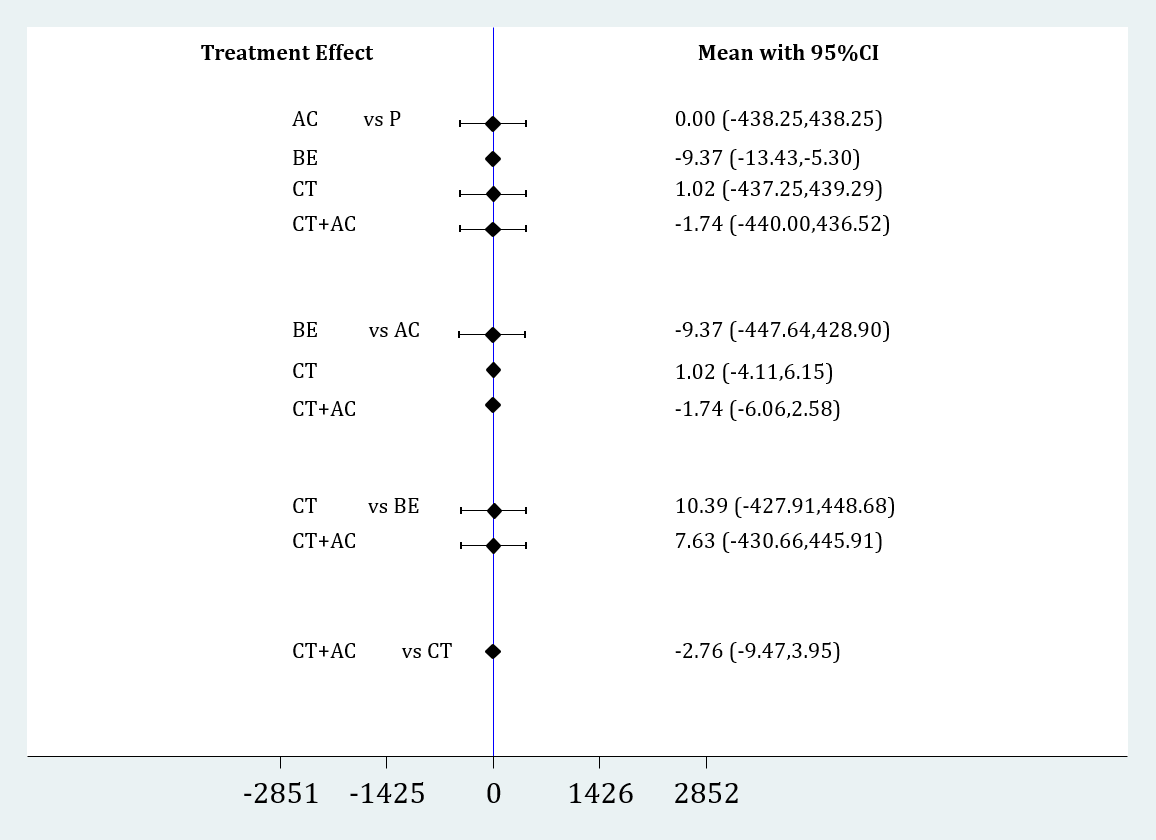


(chi-square for global consistency = 30.11, *p* = 0.0000)

Abbreviation: AC, active drug comparator; BE, bioavailability-enhanced curcuminoid preparations; CT, conventional curcuminoid preparations; CT + AC, conventional curcuminoid preparations + active drug comparator; P, placebo

### eTable N10 Descriptive table for transitivity assessment of sensitivity analysis on small-study effects for WOMAC function

| _Contrast | Studies | Mean age in years (range), SD | Female in percentage (range) | Mean BMI in kg/m^2^ (range), SD | Mean duration of baseline knee OA/pain in months (range), SD | Mean baseline WOMAC stiffness intensity (range), SD | Follow-up period in days (range) |
| --- | --- | --- | --- | --- | --- | --- | --- |
| BE vs. P | 2 | 54.75 (53.12 to 56.54), 7.10 | 84.60 (80.6 to 88.6) | 25.18 (24.92 to 25.44), 2.34 | 23.25 (21.69 to 24.8), 9.23 | 24.87 (24.32 to 25.56), 6.67 | 51 (42 to 60) |
| CT+AC vs. AC | 1 | 50.25 (50.23 to 50.27), 8.36 | 64.45 (61 to 67.9) | 27.86 (27.4 to 28.82), 5.41 | NA | 52.51 (50.99 to 54.03), 6.08 | 120 |
| CT vs. AC | 1 | 60.6 (60.3 to 60.9), 6.85 | 89.35 (86.9 to 91.81) | 26.55 (26.5 to 26.6), 3.85 | 51.65 (51.3 to 52), 52.55 | 35.36 (34.68 to 36.04), 12.92 | 28 |

Abbreviation: AC, active drug comparator; BE, bioavailability-enhanced curcuminoid preparations; CT, conventional curcuminoid preparations; CT + AC, conventional curcuminoid preparations + active drug comparator; P, placebo

### eFigure N11 Sensitivity analysis of small-study effects for VAS


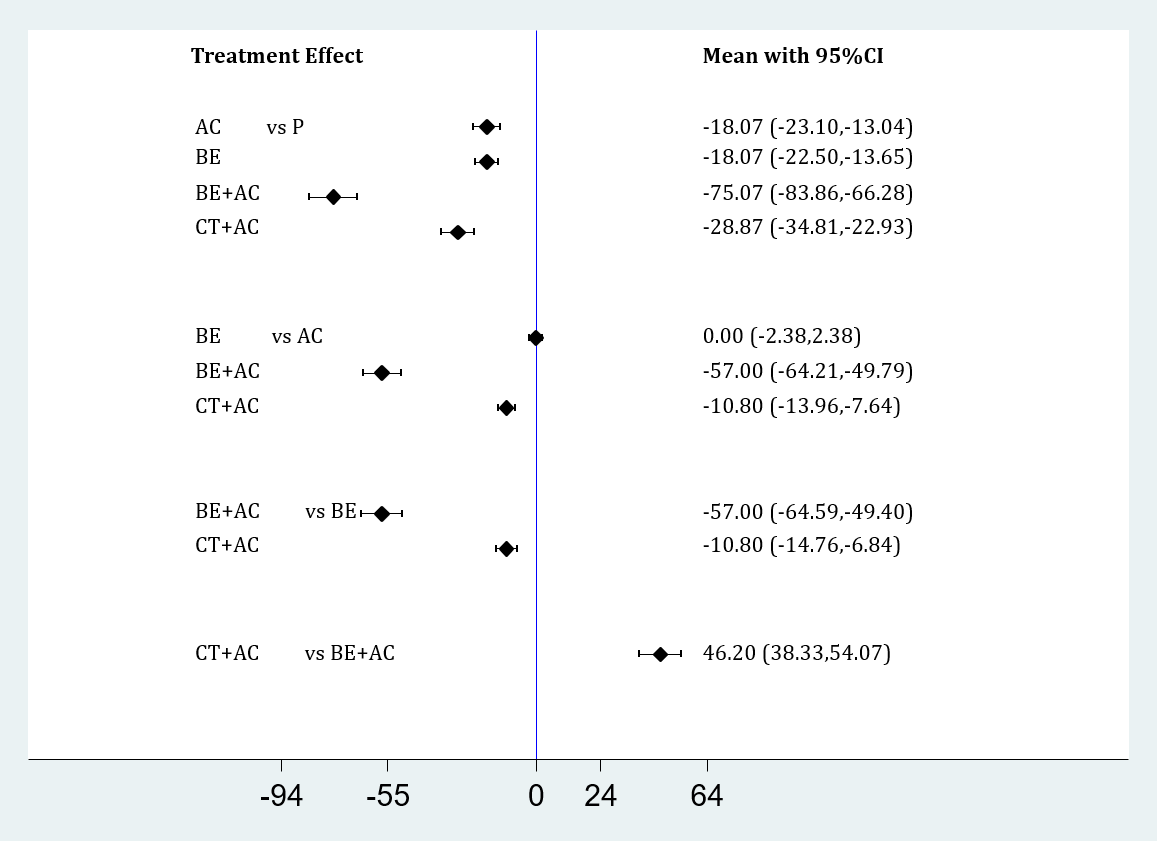


(chi-square for global consistency = 27.16, *p* = 0.0000)

Abbreviation: AC, active drug comparator; BE, bioavailability-enhanced curcuminoid preparations; BE + AC, bioavailability-enhanced curcuminoid preparations + active drug comparator; CT + AC, conventional curcuminoid preparations + active drug comparator; P, placebo

### eTable N11 Descriptive table for transitivity assessment of sensitivity analysis on small-study effects for VAS

| _Contrast | Studies | Mean age in years (range), SD | Female in percentage (range) | Mean BMI in kg/m^2^ (range), SD | Mean duration of baseline knee OA/pain in months (range), SD | Mean baseline VAS pain intensity (range), SD | Follow-up period in days (range) |
| --- | --- | --- | --- | --- | --- | --- | --- |
| BE vs. P | 2 | 55.81 (53.12 to 57.57), 8.65 | 77.35 (73.7 to 81) | 27.19 (24.92 to 29.64), 3.08 | NA | 57.64 (52.37 to 66.32), 10.60 | 51 (42 to 60) |
| BE vs. AC | 1 | 52.62 (52.14 to 53.09), 3.97 | 33.07 (30.43 to 35.71) | NA | 7.43 (7.4 to 7.45), 3.34 | 78.25 (78.1 to 78.4), 6.80 | 28 |
| BE+AC vs. AC | 1 | 48.70 (48.26 to 49.13), 5.46 | 100 | 21.95 (21.9 to 22), 1.45 | 54.96 (53.52 to 56.4), 30.21 | 81.95 (79.3 to 84.6), 17.23 | 90 |
| CT+AC vs. AC | 1 | 50.25 (50.23 to 50.27), 8.36 | 64.45 (61 to 67.9) | 27.86 (27.4 to 28.82), 5.41 | NA | 78 (76.6 to 79.4), 12.08 | 120 |

Abbreviation: AC, active drug comparator; BE, bioavailability-enhanced curcuminoid preparations; BE + AC, bioavailability-enhanced curcuminoid preparations + active drug comparator; CT + AC, conventional curcuminoid preparations + active drug comparator; P, placebo

### eFigure N12 Sensitivity analysis of baseline intensity for WOMAC pain


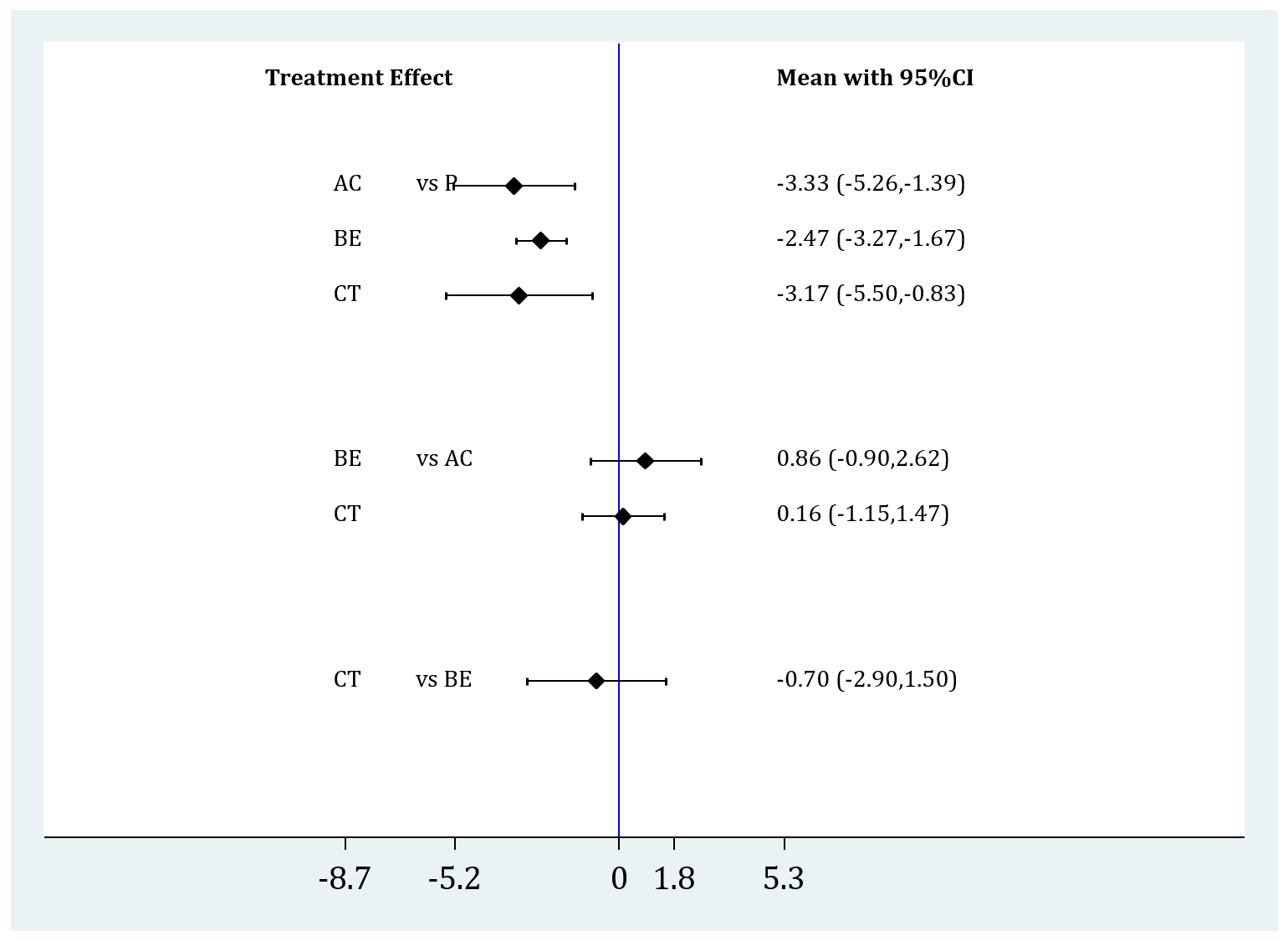


(chi-square for global consistency = 36.59, *p* = 0.0000)

Abbreviation: CI, confidence interval; AC, active drug comparator; BE, bioavailability-enhanced curcuminoid preparations; CT, conventional curcuminoid preparations; P, placebo

### eFigure N13 Sensitivity analysis of baseline intensity for WOMAC stiffness


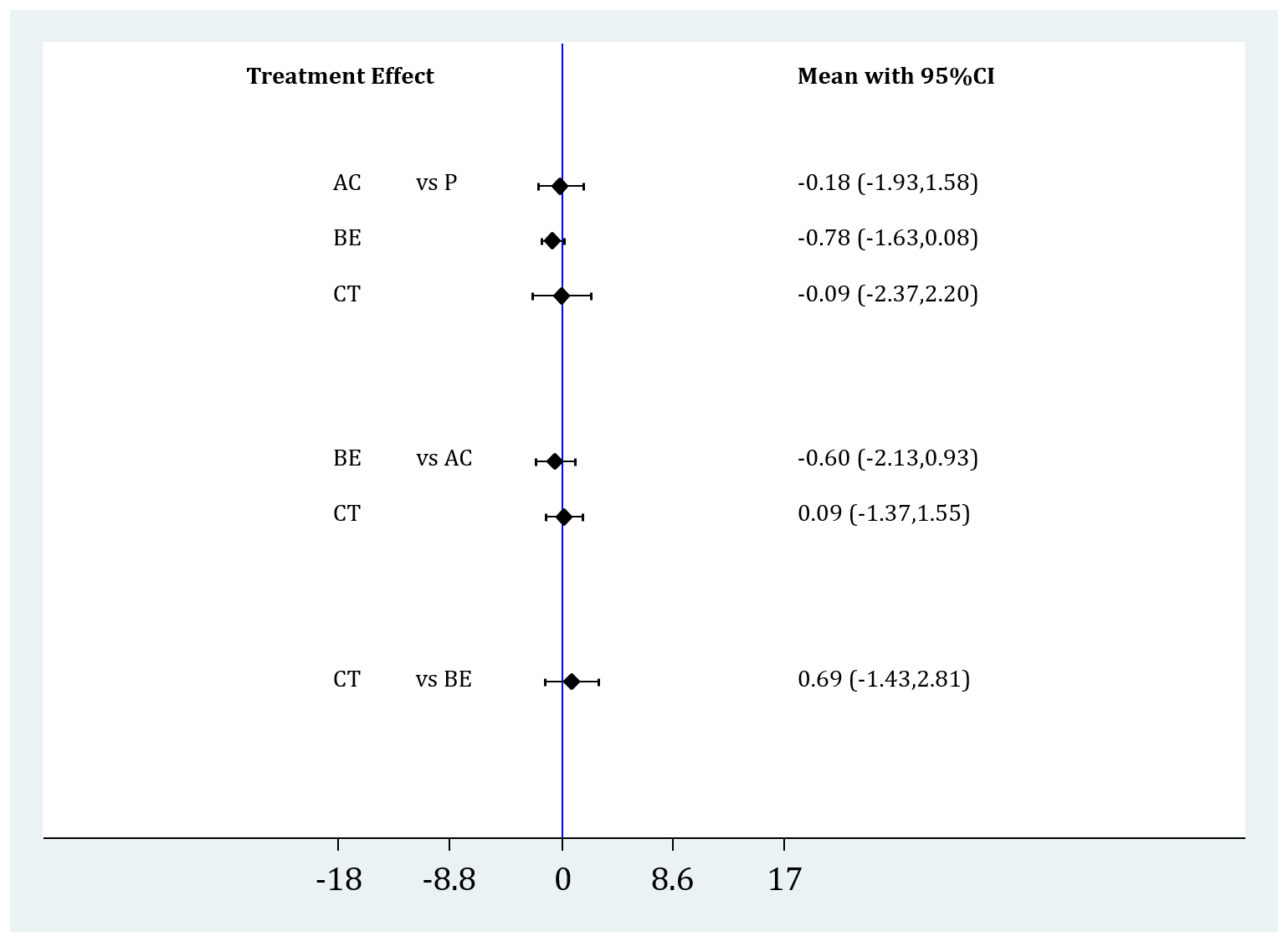


(chi-square for global consistency = 3.17, *p* = 0.0748

Abbreviation: CI, confidence interval; AC, active drug comparator; BE, bioavailability-enhanced curcuminoid preparations; CT, conventional curcuminoid preparations; P, placebo

### eFigure N14 Sensitivity analysis of baseline intensity for WOMAC function


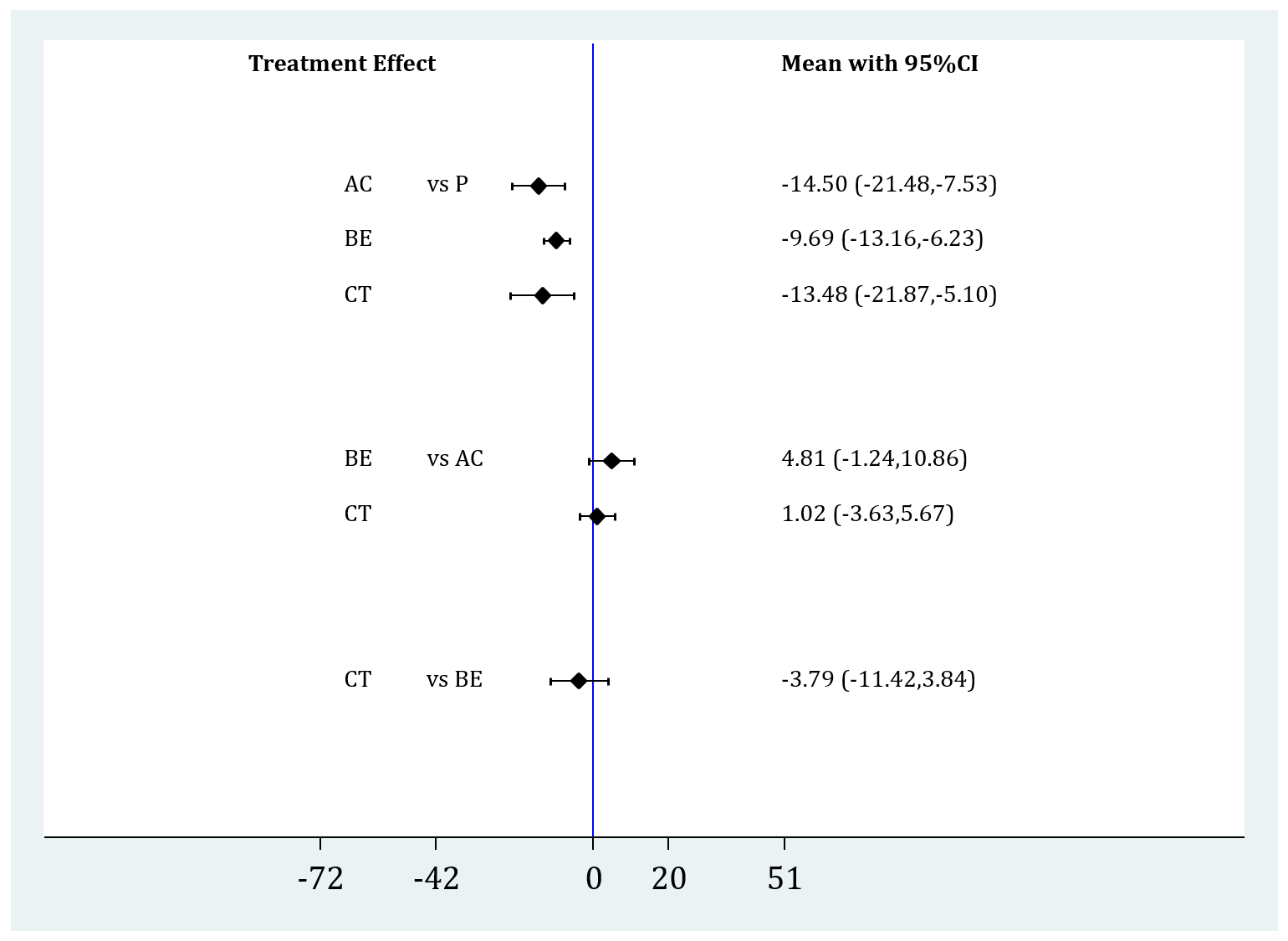


(chi-square for global consistency = 30.12, *p* = 0.0000)

Abbreviation: CI, confidence interval; AC, active drug comparator; BE, bioavailability-enhanced curcuminoid preparations; CT, conventional curcuminoid preparations; P, placebo

### eFigure N15 Sensitivity analysis of baseline intensity for VAS


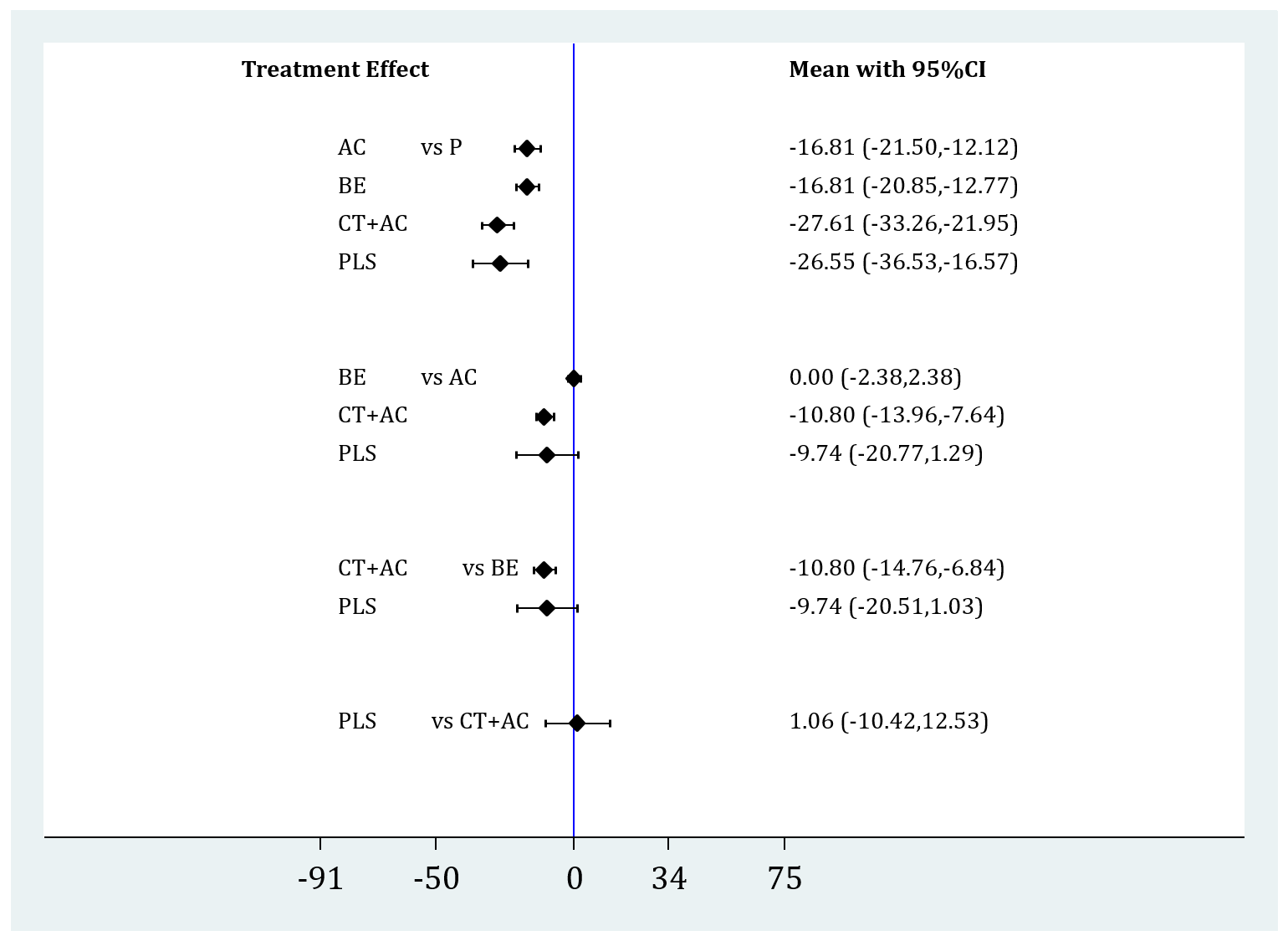


(chi-square for global consistency = 27.16, *p* = 0.0000)

Abbreviation: CI, confidence interval; AC, active drug comparator; BE, bioavailability-enhanced curcuminoid preparations; CT + AC, conventional curcuminoid preparations + active drug comparator; P, placebo; PLS, polysaccharide preparations

### eFigure N16 Adjusted funnel plot of small-study effects for WOMAC pain

Abbreviation: A = AC, active drug comparator; B = BE, bioavailability-enhanced curcuminoid preparations; C = CT, conventional curcuminoid preparations; D = CT + AC, conventional curcuminoid preparations + active drug comparator; E = P, placebo

### eFigure N17 Adjusted funnel plot of small-study effects for WOMAC stiffness

Abbreviation: A = AC, active drug comparator; B = BE, bioavailability-enhanced curcuminoid preparations; C = CT, conventional curcuminoid preparations; D = CT + AC, conventional curcuminoid preparations + active drug comparator; E = P, placebo

### eFigure N18 Adjusted funnel plot of small-study effects for WOMAC function

Abbreviation: A = AC, active drug comparator; B = BE, bioavailability-enhanced curcuminoid preparations; C = CT, conventional curcuminoid preparations; D = CT + AC, conventional curcuminoid preparations + active drug comparator; E = P, placebo

### eFigure N19 Adjusted funnel plot of small-study effects for VAS

Abbreviation: A = AC, active drug comparator; B = BE, bioavailability-enhanced curcuminoid preparations; C = BE + AC, bioavailability-enhanced curcuminoid preparations + active drug comparator; D = CT + AC, conventional curcuminoid preparations + active drug comparator; E = P, placebo

## **References**

1. Thorlund K, Walter SD, Johnston BC, Furukawa TA, Guyatt GH. Pooling health-related quality of life outcomes in meta-analysis-a tutorial and review of methods for enhancing interpretability. Res Synth Methods. 2011; doi: 10.1002/jrsm.46.

2. Singhal S, Hasan N, Nirmal K, Chawla R, Chawla S, Kalra BS, et al. Bioavailable turmeric extract for knee osteoarthritis: a randomized, non-inferiority trial versus paracetamol. Trials. 2021; doi: 10.1186/s13063-021-05053-7.

3. Haroyan A, Mukuchyan V, Mkrtchyan N, Minasyan N, Gasparyan S, Sargsyan A, et al. Efficacy and safety of curcumin and its combination with boswellic acid in osteoarthritis: a comparative, randomized, double-blind, placebo-controlled study. BMC Complement Altern Med. 2018; doi: 10.1186/s12906-017-2062-z.

4. Hashemzadeh K, Davoudian N, Jaafari MR, Mirfeizi Z. The effect of nanocurcumin in improvement of knee osteoarthritis: a randomized clinical trial. Curr Rheumatol Rev. 2020; doi: 10.2174/1874471013666191223152658.

5. Panda SK, Nirvanashetty S, Parachur VA, Mohanty N, Swain T. A randomized, double blind, placebo controlled, parallel-group study to evaluate the safety and efficacy of Curene® versus placebo in reducing symptoms of knee osteoarthritis. Biomed Res Int. 2018; doi: 10.1155/2018/5291945.

6. Panahi Y, Rahimnia AR, Sharafi M, Alishiri G, Saburi A, Sahebkar A. Curcuminoid treatment for knee osteoarthritis: a randomized double-blind placebo-controlled trial. Phytother Res. 2014; doi: 10.1002/ptr.5174.

7. Srivastava S, Saksena AK, Khattri S, Kumar S, Dagur RS. *Curcuma longa* extract reduces inflammatory and oxidative stress biomarkers in osteoarthritis of knee: a four-month, double-blind, randomized, placebo-controlled trial. Inflammopharmacology. 2016; doi: 10.1007/s10787-016-0289-9.

8. Kuptniratsaikul V, Dajpratham P, Taechaarpornkul W, Buntragulpoontawee M, Lukkanapichonchut P, Chootip C, et al. Efficacy and safety of *Curcuma domestica* extracts compared with ibuprofen in patients with knee osteoarthritis: a multicenter study. Clin Interv Aging. 2014; doi: 10.2147/cia.S58535.

9. Shep D, Khanwelkar C, Gade P, Karad S. Safety and efficacy of curcumin versus diclofenac in knee osteoarthritis: a randomized open-label parallel-arm study. Trials. 2019; doi: 10.1186/s13063-019-3327-2.

10. Atabaki M, Shariati-Sarabi Z, Tavakkol-Afshari J, Mohammadi M. Significant immunomodulatory properties of curcumin in patients with osteoarthritis; a successful clinical trial in Iran. Int Immunopharmacol. 2020; doi: 10.1016/j.intimp.2020.106607.

11. Henrotin Y, Malaise M, Wittoek R, de Vlam K, Brasseur JP, Luyten FP, et al. Bio-optimized *Curcuma longa* extract is efficient on knee osteoarthritis pain: a double-blind multicenter randomized placebo controlled three-arm study. Arthritis Res Ther. 2019; doi: 10.1186/s13075-019-1960-5.

12. Madhu K, Chanda K, Saji MJ. Safety and efficacy of *Curcuma longa* extract in the treatment of painful knee osteoarthritis: a randomized placebo-controlled trial. Inflammopharmacology. 2013; doi: 10.1007/s10787-012-0163-3.

13. Lopresti AL, Smith SJ, Jackson-Michel S, Fairchild T. An investigation into the effects of a curcumin extract (Curcugen(®)) on osteoarthritis pain of the knee: a randomised, double-blind, placebo-controlled study. Nutrients. 2021; doi: 10.3390/nu14010041.

14. Nakagawa Y, Mukai S, Yamada S, Matsuoka M, Tarumi E, Hashimoto T, et al. Short-term effects of highly-bioavailable curcumin for treating knee osteoarthritis: a randomized, double-blind, placebo-controlled prospective study. J Orthop Sci. 2014; doi: 10.1007/s00776-014-0633-0.

15. Gupte PA, Giramkar SA, Harke SM, Kulkarni SK, Deshmukh AP, Hingorani LL, et al. Evaluation of the efficacy and safety of Capsule Longvida(®) optimized curcumin (solid lipid curcumin particles) in knee osteoarthritis: a pilot clinical study. J Inflamm Res. 2019; doi: 10.2147/jir.S205390.

16. Pinsornsak P, Niempoog S. The efficacy of *Curcuma longa* L. extract as an adjuvant therapy in primary knee osteoarthritis: a randomized control trial. J Med Assoc Thai. 2012;95 Suppl 1:S51-S8.

17. Kuptniratsaikul V, Thanakhumtorn S, Chinswangwatanakul P, Wattanamongkonsil L, Thamlikitkul V. Efficacy and safety of *Curcuma domestica* extracts in patients with knee osteoarthritis. J Altern Complement Med. 2009; doi: 10.1089/acm.2008.0186.

18. Wang Z, Jones G, Winzenberg T, Cai G, Laslett LL, Aitken D, et al. Effectiveness of *Curcuma longa* extract for the treatment of symptoms and effusion-synovitis of knee osteoarthritis : a randomized trial. Ann Intern Med. 2020; doi: 10.7326/m20-0990.

19. Jamwal R. Bioavailable curcumin formulations: A review of pharmacokinetic studies in healthy volunteers. Journal of Integrative Medicine. 2018; doi: <https://doi.org/10.1016/j.joim.2018.07.001>.
